# Supplementary material for: Exploring thylakoid emergence: evolution of membrane biogenesis and photosystem II assembly in early‐diverging cyanobacteria
Source: New Phytol. 2026 Jun 5;251(3):1329–43. doi: 10.1111/nph.71284 (PMC13326532; doi:10.1111/nph.71284)

New Phytologist Supporting Information
Article title: Exploring Thylakoid Emergence: Evolution of Membrane Biogenesis and Photosystem II assembly in early-diverging Cyanobacteria
Authors: Louise Hambücken, Denis Baurain, Luc Cornet
Article acceptance date: 27 April 2026

Supplementary File

Supplementary Figures

Genome selection and phylogenomic species tree

Supplemental Figure S1: Phylogenomic tree based on ribosomal proteins. (A) Uncollapsed tree and **(B)** collapsed tree by Terrabacteria phyla. Only bootstrap values <100 are shown. The tree was reconstructed from 1867 sequences across 3424 unambiguously aligned positions using IQ-TREE v2.4.0(Minh et al., 2020) with the best-fit substitution model (Q.YEAST+I+R10) and 1,000 ultrafast bootstrap replicates.

Supplemental Figure S2: Genome completeness by Terrabacteria phylum using CheckM2(Chklovski et al., 2023). The boxplot was created using the R package ggplot2 v4.0.0(Wickham et al., 2016) on Rstudio (2025.05.1 Build 513)(Posit team, 2025).

Supplemental Figure S3: Genome contamination by Terrabacteria phylum using CheckM2. The boxplot was created using the R package ggplot2 v4.0.0(Wickham et al., 2016) on Rstudio (2025.05.1 Build 513)(Posit team, 2025).

Supplemental Figure S4: Diversity curve generated using PARNAS v0.1.6(Markin et al., 2023) for the pruning of the ribosomal tree. The curve illustrates the relationship between the number of representative genomes retained and the phylogenetic diversity captured. Based on this analysis, a subset of 950 representative genomes was selected to maximize diversity while reducing redundancy in the dataset.

Identification of the orthologous groups of interest

Figs. S5–S50: Boxplots based on the bitscore distributions of BLASTp(Camacho et al., 2009) hits for each (A) orthologous groups (OG) (B) hierarchical orthologous groups (HOG) from the N0 node inferred. The OGs and HOGs were inferred using OrthoFinder v2.5.5(Emms & Kelly, 2019). The central line represents the median, the box bounds the interquartile range (IQR), whiskers extend to 1.5× IQR, and points beyond this range are shown as outliers. Boxplots were generated using the ggplot2 package in R (Wickham et al., 2016)

Orthologous gene tree inference

Supplemental Figs. S51-S95: Gene trees of each OG of interest generated using IQ-TREE v3.0.0[7] with the best-fit substitution model and 1,000 ultrafast bootstrap replicates. Bitscores were calculated by performing a BLASTp searches (v2.9.0+)(Camacho et al., 2009) of every protein from the 950 proteomes against a database containing reference sequences of known PSII assembly factors and proteins involved in membrane dynamics (**Table S8**).

Supplemental Figs. S96-S136: Gene trees based on the best hits of a HMM search on the Cyanobacterial dataset (343 Cyanobacteriota species) with a HMM profile of the orthologous sequences of the proteins of interest. The HMM search was performed using HMMER v3.4(Finn et al., 2011). The results of the HMM search was filtered using OmpaPa (https://metacpan.org/release/Bio-MUST-Core;D. Baurain).

Supplemental Fig. S137: Additional β-sheet positioned outside of the ring structure formed during oligomerization in the proteins of the basal group I and II. (A) AlphaFold3 predicted 3D structures of the SPFH proteins of the basal group I (BAC89785.1) and basal group II (BAC89786.1) from *Gloeobacter violaceus* with pLDDT scores. (B) AlphaFold3 predicted 3D structures of SPFH proteins from *Gloeobacter violaceus* (basal group I [BAC89785.1], blue, and basal group II [BAC89786.1], green) and two known SPFH proteins present in the same orthogroup from *Synechocystis* sp. PCC 6803 (Slr1106 [P72754], yellow, and Slr1768 [P73049], pink). (C,D) AlphaFold3 predicted 3D structures of the decamerization of BAC89785.1 with pLDDT scores. (E) Position of the extra β-sheet in the decamerization of BAC89785.1. The Predicted Local Distance Difference Test (pLDDT) scores mapped indicate confidence levels of the predicted models.

Supplemental Fig. S138: Interpro Scan predictions(Jones et al., 2014) of functional domains within the closest homologs of Sll0606 in *Gloeobacterales*. Functional protein domains were annotated with InterPro v5.48-83.0. The figure summarizes the domain architecture of each homolog.

Supplemental Figure S139: Alignment of the cyanobacterial Slr0144 and Slr0147 sequences. The alignment was performed using MAFFT v7.471 (Katoh, 2002). Residues that are 100% conserved are indicated by an asterisk (*); residues conserved in more than 50% of the sequences are indicated by a colon (:); and poorly conserved residues are indicated by a dot (·).

Supplemental Figure S140: Alignment of the cyanobacterial YidC sequences. The alignment was performed using MAFFT v7.471 (Katoh, 2002). The periplasmic domain as well as the extra alpha helices are indicated with braces. Residues that are 100% conserved are indicated by an asterisk (*); residues conserved in more than 50% of the sequences are indicated by a colon (:); and poorly conserved residues are indicated by a dot (·).

Supplemental Figure S141: (A) AlphaFold3 predicted 3D structures of SecD from *Gloeobacter violaceus* (GlSecD [BAC92107.1], blue), Thermostichales cyanobacterium GCA_039794545.1 (TSecD, yellow), *Synechocystis* sp. PCC 6803 (SynSecD, pink). **(B)** Additional helices identified in the periplasmic domain of SynSecD (pink). TSecD and GlSecD are shown respectively in blue and yellow. The Predicted Local Distance Difference Test (pLDDT) scores mapped onto the GlSecD, TsecD and SynSecD indicate confidence levels of the predicted models.

Supplemental Figure S142: (A) AlphaFold3 predicted 3D structures of SecF from *Gloeobacter violaceus* (GlSecF [BAC92107.1], blue), Thermostichales cyanobacterium GCA_039794545.1 (TSecF, yellow) cyan, and *Synechocystis* sp. PCC 6803 (SynSecF, pink). **(B)** Additional helices identified in the periplasmic domain of SecF from SynSecF. The Predicted Local Distance Difference Test (pLDDT) scores mapped onto the GlSecF, TsecF and SynSecF indicate confidence levels of the predicted models.

Supplemental Figure S143: Alignment of the cyanobacterial CyanoP sequences. The alignment was performed using MAFFT v7.471 (Katoh, 2002). Residues that are 100% conserved are indicated by an asterisk (*); residues conserved in more than 50% of the sequences are indicated by a colon (:); and poorly conserved residues are indicated by a dot (·).

Supplemental Figure S144: Visualization of the selection of the sequences similar to Slr1106 with Ompa-Pa. Matching sequences were plotted based on their sequence length in function of their -log10(e-value). The blue box corresponds to the selection (Copy per organism: 1 to 10, range of the fraction covering the HMM profile: 0 to 1, range of -log(evalue): 9 to 176, range of sequence length: 140 to 423). Sequences were visualized based on their (A) alignment coverage and (B) number of copies per genome.

Supplemental Figure S145: Visualization of the selection of the sequences similar to Slr1768 with Ompa-Pa. Matching sequences were plotted based on their sequence length in function of their -log10(e-value). The blue box corresponds to the selection (copy per organism: 1 to 10, range of the fraction covering the HMM profile: 0 to 1, range of -log(evalue): 13 to 175, range of sequence length: 149 to 456). Sequences were visualized based on their (A) alignment coverage and (B) number of copies per genome.

Supplemental Figure S146: (A) Preliminary multigenic family tree. The subtree corresponding to sequences similar to Slr1106 is shown in red. The tree was constructed from 11,714 sequences, corresponding to 319 unambiguously aligned positions, using FastTree(Price et al., 2010) with default parameters. The subtree highlighted represents the selected Slr1106 subtree, corresponding to 1380 sequences. **(B) Subtree limited to cyanobacterial sequences similar to Slr1106 (highlighted in red on panel A)).** The node support values are based on the Shimodaira-Hasegawa (SH) test (Gascuel, 1997).

Supplemental Figure S147: (A) Preliminary multigenic family tree. The subtree corresponding to sequences similar to Slr1768 is shown in red. The tree was constructed from 16,475 sequences, corresponding to 325 unambiguously aligned positions, using FastTree(Price et al., 2010) with default parameters. The subtree highlighted in blue represents the selected Slr1768 subtree, corresponding to 169 sequences. **(B) Subtree limited to cyanobacterial sequences similar to Slr1768 (highlighted in red on panel A)).** The node support values are based on the Shimodaira-Hasegawa (SH) test (Gascuel, 1997).s

References

Addlesee, H. A., Fiedor, L., & Hunter, C. N. (2000). Physical Mapping of *bchG* , *orf427* , and *orf177* in the Photosynthesis Gene Cluster of *Rhodobacter sphaeroides*: Functional Assignment of the Bacteriochlorophyll Synthetase Gene. *Journal of Bacteriology*, *182*(11), 3175–3182. https://doi.org/10.1128/JB.182.11.3175-3182.2000

Akulinkina, D. V., Bolychevtseva, Yu. V., Elanskaya, I. V., Karapetyan, N. V., & Yurina, N. P. (2015). Association of high light-inducible HliA/HliB stress proteins with photosystem 1 trimers and monomers of the cyanobacterium Synechocystis PCC 6803. *Biochemistry (Moscow)*, *80*(10), 1254–1261. https://doi.org/10.1134/s0006297915100053

Anbudurai, P. R., Mor, T. S., Ohad, I., Shestakov, S. V., & Pakrasi, H. B. (1994). The ctpA gene encodes the C-terminal processing protease for the D1 protein of the photosystem II reaction center complex. *Proceedings of the National Academy of Sciences*, *91*(17), 8082–8086. https://doi.org/10.1073/pnas.91.17.8082

Armbruster, U., Zühlke, J., Rengstl, B., Kreller, R., Makarenko, E., Rühle, T., Schünemann, D., Jahns, P., Weisshaar, B., Nickelsen, J., & Leister, D. (2010). The *Arabidopsis* Thylakoid Protein PAM68 Is Required for Efficient D1 Biogenesis and Photosystem II Assembly. *The Plant Cell*, *22*(10), 3439–3460. https://doi.org/10.1105/tpc.110.077453

Bečková, M., Gardian, Z., Yu, J., Konik, P., Nixon, P. J., & Komenda, J. (2017). Association of Psb28 and Psb27 Proteins with PSII-PSI Supercomplexes upon Exposure of Synechocystis sp. PCC 6803 to High Light. *Molecular Plant*, *10*(1), 62–72. https://doi.org/10.1016/j.molp.2016.08.001

Bučinská, L., Kiss, E., Konik, P., Knoppova, J., Komenda, J., & Sobotka, R. (2018). The ribosome-bound protein Pam68 promotes insertion of chlorophyll into the CP47 subunit of Photosystem II. *Plant Physiology*, pp.00061.2018. https://doi.org/10.1104/pp.18.00061

Camacho, C., Coulouris, G., Avagyan, V., Ma, N., Papadopoulos, J., Bealer, K., & Madden, T. L. (2009). BLAST+: Architecture and applications. *BMC Bioinformatics*, *10*(1), 421. https://doi.org/10.1186/1471-2105-10-421

Chidgey, J. W., Linhartová, M., Komenda, J., Jackson, P. J., Dickman, M. J., Canniffe, D. P., Koník, P., Pilný, J., Hunter, C. N., & Sobotka, R. (2014a). A Cyanobacterial Chlorophyll Synthase-

HliD Complex Associates with the Ycf39 Protein and the YidC/Alb3 Insertase. *The Plant Cell*, *26*(3), 1267–1279. https://doi.org/10.1105/tpc.114.124495

Chidgey, J. W., Linhartová, M., Komenda, J., Jackson, P. J., Dickman, M. J., Canniffe, D. P., Koník, P., Pilný, J., Hunter, C. N., & Sobotka, R. (2014b). A Cyanobacterial Chlorophyll Synthase-HliD Complex Associates with the Ycf39 Protein and the YidC/Alb3 Insertase. *The Plant Cell*, *26*(3), 1267–1279. https://doi.org/10.1105/tpc.114.124495

Chklovski, A., Parks, D. H., Woodcroft, B. J., & Tyson, G. W. (2023). CheckM2: A rapid, scalable and accurate tool for assessing microbial genome quality using machine learning. *Nature Methods*, *20*(8), 1203–1212. https://doi.org/10.1038/s41592-023-01940-w

Dobáková, M., Sobotka, R., Tichý, M., & Komenda, J. (2009). Psb28 Protein Is Involved in the Biogenesis of the Photosystem II Inner Antenna CP47 (PsbB) in the Cyanobacterium *Synechocystis* sp. PCC 6803. *Plant Physiology*, *149*(2), 1076–1086. https://doi.org/10.1104/pp.108.130039

Emms, D. M., & Kelly, S. (2019). OrthoFinder: Phylogenetic orthology inference for comparative genomics. *Genome Biology*, *20*(1), 238. https://doi.org/10.1186/s13059-019-1832-y

Finn, R. D., Clements, J., & Eddy, S. R. (2011). HMMER web server: Interactive sequence similarity searching. *Nucleic Acids Research*, *39*(suppl), W29–W37. https://doi.org/10.1093/nar/gkr367

Frain, K. M., Gangl, D., Jones, A., Zedler, J. A. Z., & Robinson, C. (2016). Protein translocation and thylakoid biogenesis in cyanobacteria. *Biochimica et Biophysica Acta (BBA) - Bioenergetics*, *1857*(3), 266–273. https://doi.org/10.1016/j.bbabio.2015.08.010

Fu, A., He, Z., Cho, H. S., Lima, A., Buchanan, B. B., & Luan, S. (2007). A chloroplast cyclophilin functions in the assembly and maintenance of photosystem II in *Arabidopsis thaliana*. *Proceedings of the National Academy of Sciences*, *104*(40), 15947–15952. https://doi.org/10.1073/pnas.0707851104

Gascuel, O. (1997). BIONJ: An improved version of the NJ algorithm based on a simple model of sequence data. *Molecular Biology and Evolution*, *14*(7), 685–695. https://doi.org/10.1093/oxfordjournals.molbev.a025808

Gathmann, Sven, Eva Rupprecht, Uwe Kahmann, & Dirk Schneider. (2008). A Conserved Structure and Function of the YidC Homologous Protein Slr1471 from Synechocystis sp. PCC 6803. *Journal of Microbiology and Biotechnology*, *18*(6), 1090–1094.

Jones, P., Binns, D., Chang, H.-Y., Fraser, M., Li, W., McAnulla, C., McWilliam, H., Maslen, J., Mitchell, A., Nuka, G., Pesseat, S., Quinn, A. F., Sangrador-Vegas, A., Scheremetjew, M., Yong, S.-Y., Lopez, R., & Hunter, S. (2014). InterProScan 5: Genome-scale protein function classification. *Bioinformatics*, *30*(9), 1236–1240. https://doi.org/10.1093/bioinformatics/btu031

Karamoko, M., Cline, S., Redding, K., Ruiz, N., & Hamel, P. P. (2011). Lumen Thiol Oxidoreductase1, a Disulfide Bond-Forming Catalyst, Is Required for the Assembly of Photosystem II in*Arabidopsis*. *The Plant Cell*, *23*(12), 4462–4475. https://doi.org/10.1105/tpc.111.089680

Karnauchov, I., Herrmann, R. G., Pakrasi, H. B., & Klösgen, R. B. (1997). Transport of CtpA Protein from the Cyanobacterium *Synechocystis* 6803 Across the Thylakoid Membrane in Chloroplasts. *European Journal of Biochemistry*, *249*(2), 497–504. https://doi.org/10.1111/j.1432-1033.1997.t01-1-00497.x

Kashino, Y., Takahashi, T., Inoue-Kashino, N., Ban, A., Ikeda, Y., Satoh, K., & Sugiura, M. (2007). Ycf12 is a core subunit in the photosystem II complex. *Biochimica et Biophysica Acta (BBA) - Bioenergetics*, *1767*(11), 1269–1275. https://doi.org/10.1016/j.bbabio.2007.08.008

Katoh, K. (2002). MAFFT: A novel method for rapid multiple sequence alignment based on fast Fourier transform. *Nucleic Acids Research*, *30*(14), 3059–3066. https://doi.org/10.1093/nar/gkf436

Keren, N., Ohkawa, H., Welsh, E. A., Liberton, M., & Pakrasi, H. B. (2005). Psb29, a Conserved 22-kD Protein, Functions in the Biogenesis of Photosystem II Complexes in *Synechocystis* and *Arabidopsis*. *The Plant Cell*, *17*(10), 2768–2781. https://doi.org/10.1105/tpc.105.035048

Kiss, É., Knoppová, J., Aznar, G. P., Pilný, J., Yu, J., Halada, P., Nixon, P. J., Sobotka, R., & Komenda, J. (2019). A Photosynthesis-Specific Rubredoxin-Like Protein Is Required for

Efficient Association of the D1 and D2 Proteins during the Initial Steps of Photosystem II Assembly. *The Plant Cell*, *31*(9), 2241–2258. https://doi.org/10.1105/tpc.19.00155

Knoppová, J., Yu, J., Konik, P., Nixon, P. J., & Komenda, J. (2016). CyanoP is Involved in the Early Steps of Photosystem II Assembly in the Cyanobacterium*Synechocystis*sp. PCC 6803. *Plant and Cell Physiology*, *57*(9), 1921–1931. https://doi.org/10.1093/pcp/pcw115

Komenda, J., Knoppová, J., Kopečná, J., Sobotka, R., Halada, P., Yu, J., Nickelsen, J., Boehm, M., & Nixon, P. J. (2012). The Psb27 Assembly Factor Binds to the CP43 Complex of Photosystem II in the Cyanobacterium *Synechocystis* sp. PCC 6803. *Plant Physiology*, *158*(1), 476–486. https://doi.org/10.1104/pp.111.184184

Komenda, J., Nickelsen, J., Tichý, M., Prášil, O., Eichacker, L. A., & Nixon, P. J. (2008). The Cyanobacterial Homologue of HCF136/YCF48 Is a Component of an Early Photosystem II Assembly Complex and Is Important for Both the Efficient Assembly and Repair of Photosystem II in Synechocystis sp. PCC 6803. *Journal of Biological Chemistry*, *283*(33), 22390–22399. https://doi.org/10.1074/jbc.m801917200

Komenda, J., & Sobotka, R. (2016). Cyanobacterial high-light-inducible proteins—Protectors of chlorophyll–protein synthesis and assembly. *Biochimica et Biophysica Acta (BBA) - Bioenergetics*, *1857*(3), 288–295. https://doi.org/10.1016/j.bbabio.2015.08.011

Konert, M. M. (2022). High-light-inducible proteins HliA and HliB: pigment binding and protein–protein interactions. *Photosynthesis Research*, *152*, 317–332.

Kufryk, G. I., & Vermaas, W. F. J. (2001). A Novel Protein Involved in the Functional Assembly of the Oxygen-Evolving Complex of Photosystem II in *Synechocystis* sp. PCC 6803. *Biochemistry*, *40*(31), 9247–9255. https://doi.org/10.1021/bi0026526

Kufryk, G. I., & Vermaas, W. F. J. (2003). Slr2013 Is a Novel Protein Regulating Functional Assembly of Photosystem II in *Synechocystis* sp. Strain PCC 6803. *Journal of Bacteriology*, *185*(22), 6615–6623. https://doi.org/10.1128/jb.185.22.6615-6623.2003

Lima, A., Lima, S., Wong, J. H., Phillips, R. S., Buchanan, B. B., & Luan, S. (2006). A redox-active FKBP-type immunophilin functions in accumulation of the photosystem II supercomplex in *Arabidopsis thaliana*. *Proceedings of the National Academy of Sciences*, *103*(33), 12631–12636. https://doi.org/10.1073/pnas.0605452103

Linhartová, M., Bučinská, L., Halada, P., Ječmen, T., Šetlík, J., Komenda, J., & Sobotka, R. (2014). Accumulation of the Type IV prepilin triggers degradation of SecY and YidC and inhibits synthesis of Photosystem II proteins in the cyanobacterium *Synechocystis* PCC 6803. *Molecular Microbiology*, *93*(6), 1207–1223. https://doi.org/10.1111/mmi.12730

Markin, A., Wagle, S., Grover, S., Vincent Baker, A. L., Eulenstein, O., & Anderson, T. K. (2023). PARNAS: Objectively Selecting the Most Representative Taxa on a Phylogeny. *Systematic Biology*, *72*(5), 1052–1063. https://doi.org/10.1093/sysbio/syad028

Minh, B. Q., Schmidt, H. A., Chernomor, O., Schrempf, D., Woodhams, M. D., Von Haeseler, A., & Lanfear, R. (2020). IQ-TREE 2: New Models and Efficient Methods for Phylogenetic Inference in the Genomic Era. *Molecular Biology and Evolution*, *37*(5), 1530–1534. https://doi.org/10.1093/molbev/msaa015

Nowaczyk, M. M. (2014). Localization of the CyanoP binding site on photosystem II by surface plasmon resonance spectroscopy. *Frontiers in Plant Science*, *5*(595).

Parks, D. H., Chuvochina, M., Rinke, C., Mussig, A. J., Chaumeil, P.-A., & Hugenholtz, P. (2022). GTDB: An ongoing census of bacterial and archaeal diversity through a phylogenetically consistent, rank normalized and complete genome-based taxonomy. *Nucleic Acids Research*, *50*(D1), D785–D794. https://doi.org/10.1093/nar/gkab776

Pascual-Aznar, G., Konert, G., Bečkovo, M., Kotabovo, E., Gardian, Z., Knoppovo, J., Bučinsko, L., Kaňa, R., Sobotka, R., & Komenda, J. (2021). Psb35 Protein Stabilizes the CP47 Assembly Module and Associated High-Light Inducible Proteins during the Biogenesis of Photosystem II in the Cyanobacterium*Synechocystis*sp. PCC6803. *Plant and Cell Physiology*, *62*(1), 178–190. https://doi.org/10.1093/pcp/pcaa148

Plöchinger, M., Schwenkert, S., Von Sydow, L., Schröder, W. P., & Meurer, J. (2016). Functional Update of the Auxiliary Proteins PsbW, PsbY, HCF136, PsbN, TerC and ALB3 in Maintenance and Assembly of PSII. *Frontiers in Plant Science*, *7*. https://doi.org/10.3389/fpls.2016.00423

Posit team. (2025). *RStudio: Integrated development environment for R* [Manual]. Posit Software, PBC. http://www.posit.co/

Price, M. N., Dehal, P. S., & Arkin, A. P. (2010). FastTree 2 – Approximately Maximum-Likelihood Trees for Large Alignments. *PLoS ONE*, *5*(3), e9490. https://doi.org/10.1371/journal.pone.0009490

Proctor, M. S., Chidgey, J. W., Shukla, M. K., Jackson, P. J., Sobotka, R., Hunter, C. N., & Hitchcock, A. (2018). Plant and algal chlorophyll synthases function in *Synechocystis* and interact with the YidC/Alb3 membrane insertase. *FEBS Letters*, *592*(18), 3062–3073. https://doi.org/10.1002/1873-3468.13222

Rahimzadeh-Karvansara, P., Pascual-Aznar, G., Bečková, M., & Komenda, J. (2022). Psb34 protein modulates binding of high-light-inducible proteins to CP47-containing photosystem II assembly intermediates in the cyanobacterium Synechocystis sp. PCC 6803. *Photosynthesis Research*, *152*(3), 333–346. https://doi.org/10.1007/s11120-022-00908-9

Rengstl, B., Knoppová, J., Komenda, J., & Nickelsen, J. (2013). Characterization of a Synechocystis double mutant lacking the photosystem II assembly factors YCF48 and Sll0933. *Planta*, *237*(2), 471–480. https://doi.org/10.1007/s00425-012-1720-0

Rengstl, B., Oster, U., Stengel, A., & Nickelsen, J. (2011). An Intermediate Membrane Subfraction in Cyanobacteria Is Involved in an Assembly Network for Photosystem II Biogenesis. *Journal of Biological Chemistry*, *286*(24), 21944–21951. https://doi.org/10.1074/jbc.m111.237867

Roose, J. L., Kashino, Y., & Pakrasi, H. B. (2007). The PsbQ protein defines cyanobacterial Photosystem II complexes with highest activity and stability. *Proceedings of the National Academy of Sciences*, *104*(7), 2548–2553. https://doi.org/10.1073/pnas.0609337104

Sachelaru, I., Petriman, N. A., Kudva, R., Kuhn, P., Welte, T., Knapp, B., Drepper, F., Warscheid, B., & Koch, H.-G. (2013). YidC Occupies the Lateral Gate of the SecYEG Translocon and Is Sequentially Displaced by a Nascent Membrane Protein. *Journal of Biological Chemistry*, *288*(23), 16295–16307. https://doi.org/10.1074/jbc.m112.446583

Sakata, S., Mizusawa, N., Kubota-Kawai, H., Sakurai, I., & Wada, H. (2013). Psb28 is involved in recovery of photosystem II at high temperature in Synechocystis sp. PCC 6803. *Biochimica et Biophysica Acta (BBA) - Bioenergetics*, *1827*(1), 50–59. https://doi.org/10.1016/j.bbabio.2012.10.004

Schottkowski, M., Ratke, J., Oster, U., Nowaczyk, M., & Nickelsen, J. (2009). Pitt, a Novel Tetratricopeptide Repeat Protein Involved in Light-Dependent Chlorophyll Biosynthesis and Thylakoid Membrane Biogenesis in Synechocystis sp. PCC 6803. *Molecular Plant*, *2*(6), 1289–1297. https://doi.org/10.1093/mp/ssp075

Šesták, Z. (2007). Golbeck, J.H. (ed.): Photosystem I. The Light-Driven Plastocyanin:Ferredoxin Oxidoreductase. *Photosynthetica*, *45*(4), 488–488. https://doi.org/10.1007/s11099-007-0083-4

Shen, G., Antonkine, M. L., Van Der Est, A., Vassiliev, I. R., Brettel, K., Bittl, R., Zech, S. G., Zhao, J., Stehlik, D., Bryant, D. A., & Golbeck, J. H. (2002). Assembly of Photosystem I. *Journal of Biological Chemistry*, *277*(23), 20355–20366. https://doi.org/10.1074/jbc.m201104200

Siebenaller, C., & Schneider, D. (2023). Cyanobacterial membrane dynamics in the light of eukaryotic principles. *Bioscience Reports*, *43*(2), BSR20221269. https://doi.org/10.1042/BSR20221269

Singh, A. K., Li, H., & Sherman, L. A. (2004). Microarray analysis and redox control of gene expression in the cyanobacterium *Synechocystis* sp. PCC 6803. *Physiologia Plantarum*, *120*(1), 27–35. https://doi.org/10.1111/j.0031-9317.2004.0232.x

Sirpiö, S., Khrouchtchova, A., Allahverdiyeva, Y., Hansson, M., Fristedt, R., Vener, A. V., Scheller, H. V., Jensen, P. E., Haldrup, A., & Aro, E. (2008). AtCYP38 ensures early biogenesis, correct assembly and sustenance of photosystem II. *The Plant Journal*, *55*(4), 639–651. https://doi.org/10.1111/j.1365-313x.2008.03532.x

Torabi, S., Umate, P., Manavski, N., Plöchinger, M., Kleinknecht, L., Bogireddi, H., Herrmann, R. G., Wanner, G., Schröder, W. P., & Meurer, J. (2014). PsbN Is Required for Assembly of the Photosystem II Reaction Center in *Nicotiana tabacum*. *The Plant Cell*, *26*(3), 1183–1199. https://doi.org/10.1105/tpc.113.120444

Wegener, K. M., Bennewitz, S., Oelmüller, R., & Pakrasi, H. B. (2011). The Psb32 Protein Aids in Repairing Photodamaged Photosystem II in the Cyanobacterium Synechocystis 6803. *Molecular Plant*, *4*(6), 1052–1061. https://doi.org/10.1093/mp/ssr044

Wegener, K. M., Welsh, E. A., Thornton, L. E., Keren, N., Jacobs, J. M., Hixson, K. K., Monroe, M. E., Camp, D. G., Smith, R. D., & Pakrasi, H. B. (2008). High Sensitivity Proteomics Assisted Discovery of a Novel Operon Involved in the Assembly of Photosystem II, a

Membrane Protein Complex. *Journal of Biological Chemistry*, *283*(41), 27829–27837. https://doi.org/10.1074/jbc.m803918200

Wickham, H., Chang, W., Henry, L., Pedersen, T. L., Takahashi, K., Wilke, C., Woo, K., Yutani, H., Dunnington, D., Brand, T. van den, Posit, & PBC. (2016). *ggplot2: Create Elegant Data Visualisations Using the Grammar of Graphics* (Version 3.5.1) [Computer software]. https://cran.r-project.org/web/packages/ggplot2/index.html

Wong, T. K. F., Nhan Ly-Trong, Huaiyan Ren, Hector Baños, Andrew J. Roger, Edward Susko, Chris Bielow, Nicola De Maio, Nick Goldman, Matthew W. Hahn, Gavin Huttley, Robert Lanfear, & Bui Quang Minh. (2025). IQ-TREE 3: Phylogenomic Inference Software using Complex Evolutionary Models. *EcoEvoRxiv*. https://doi.org/https://doi.org/10.32942/X2P62N

Wysocka, A. (2025). High-light-inducible proteins control associations between chlorophyll synthase and the Photosystem II biogenesis factor Ycf39. *Plant Physiology*, *198*, kiaf213.

Yang, H., Liao, L., Bo, T., Zhao, L., Sun, X., Lu, X., Norling, B., & Huang, F. (2014). Slr0151 in *Synechocystis* sp. PCC 6803 is required for efficient repair of photosystem II under high-light condition. *Journal of Integrative Plant Biology*, *56*(12), 1136–1150. https://doi.org/10.1111/jipb.12275

Zhang, S., Frankel, L. K., & Bricker, T. M. (2010). The Sll0606 Protein Is Required for Photosystem II Assembly/Stability in the Cyanobacterium Synechocystis sp. PCC 6803. *Journal of Biological Chemistry*, *285*(42), 32047–32054. https://doi.org/10.1074/jbc.m110.166983

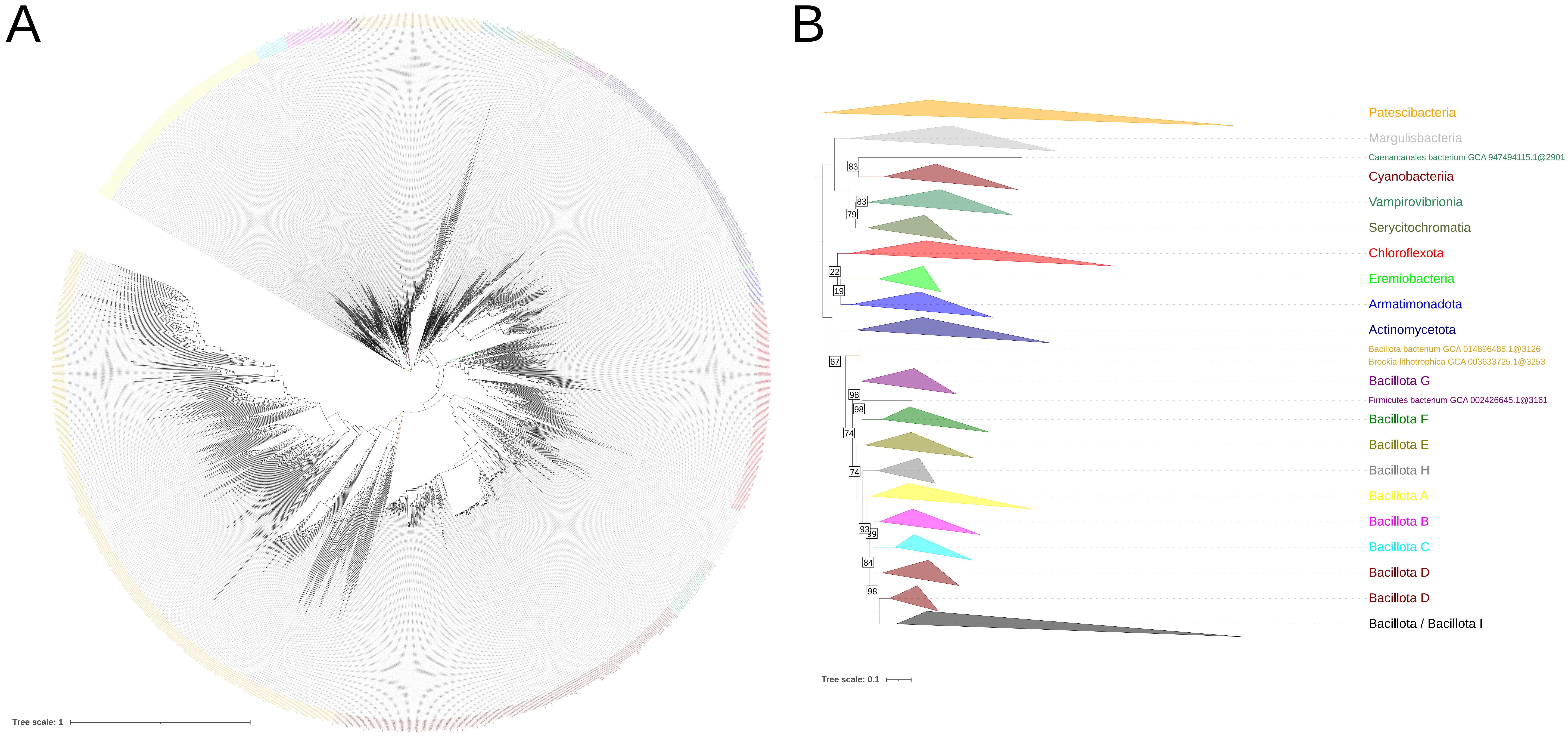
A
B
Tree scale: 1
Tree scale: 0.1
Patescibacteria
Margulisbacteria
Caenarcanales bacterium GCA 947494115.1@2901
Cyanobacteriia
Vampirovibrionia
Serycitochromatia
Chloroflexota
Eremiobacteria
Armatimonadota
Actinomycetota
Bacillota bacterium GCA 014896485.1@3126
Brockia lithotrophica GCA 003633725.1@3253
Bacillota G
Firmicutes bacterium GCA 002426645.1@3161
Bacillota F
Bacillota E
Bacillota H
Bacillota A
Bacillota B
Bacillota C
Bacillota D
Bacillota D
Bacillota / Bacillota I
83
83
79
22
19
67
98
98
74
74
93
99
84
98

Fig. S1

Fig. S2

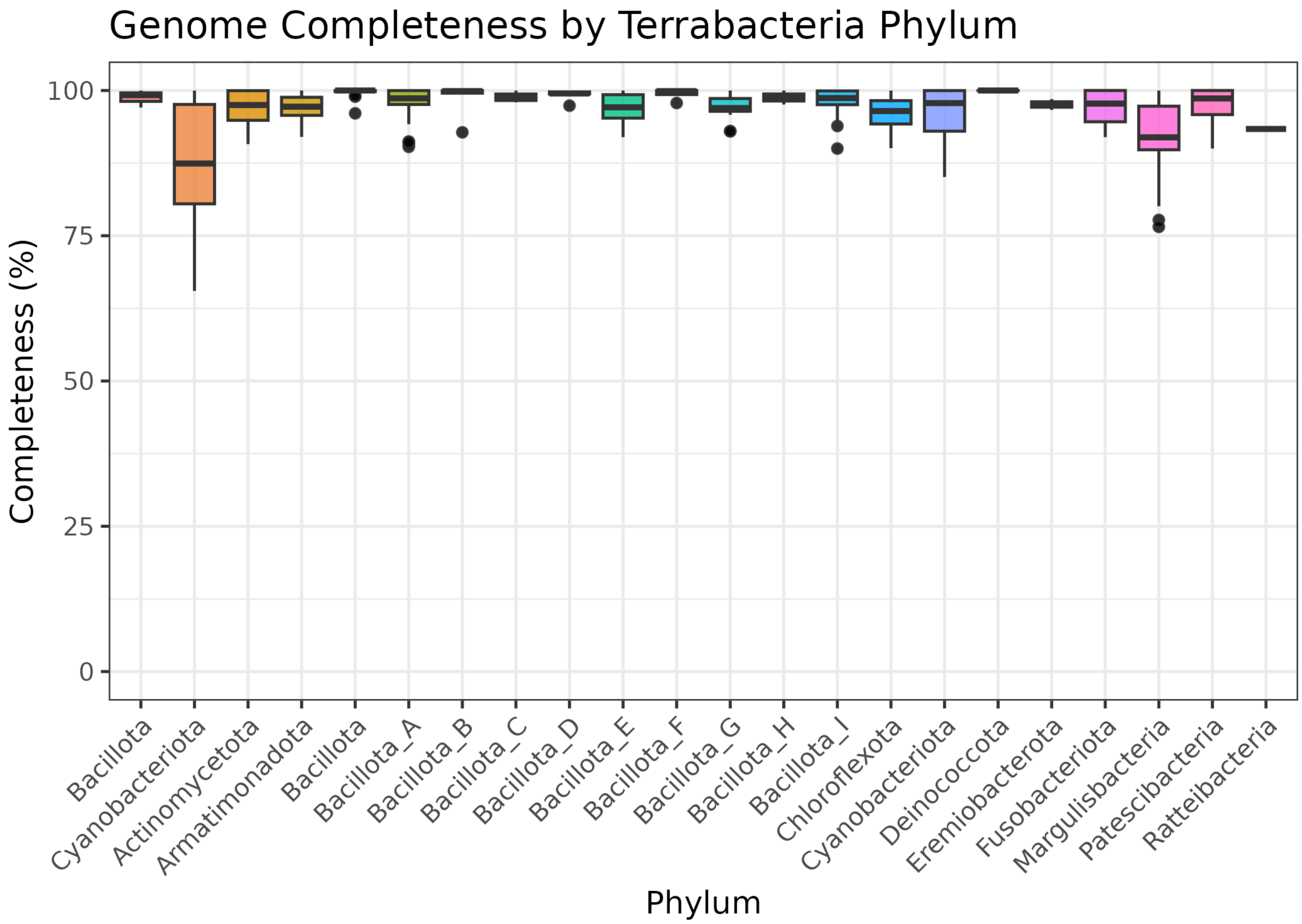

Genome Completeness by Terrabacteria Phylum
Completeness (%)
100
75
50
25
0
Bacillota
Cyanobacteriota
Actinomycetota
Armatimonadota
Bacillota
Bacillota_A
Bacillota_B
Bacillota_C
Bacillota_D
Bacillota_E
Bacillota_F
Bacillota_G
Bacillota_H
Bacillota_I
Chloroflexota
Cyanobacteriota
Deinococcota
Eremiobacterota
Fusobacteriota
Margulisbacteria
Patescibacteria
Ratteibacteria
Phylum

Fig. S3

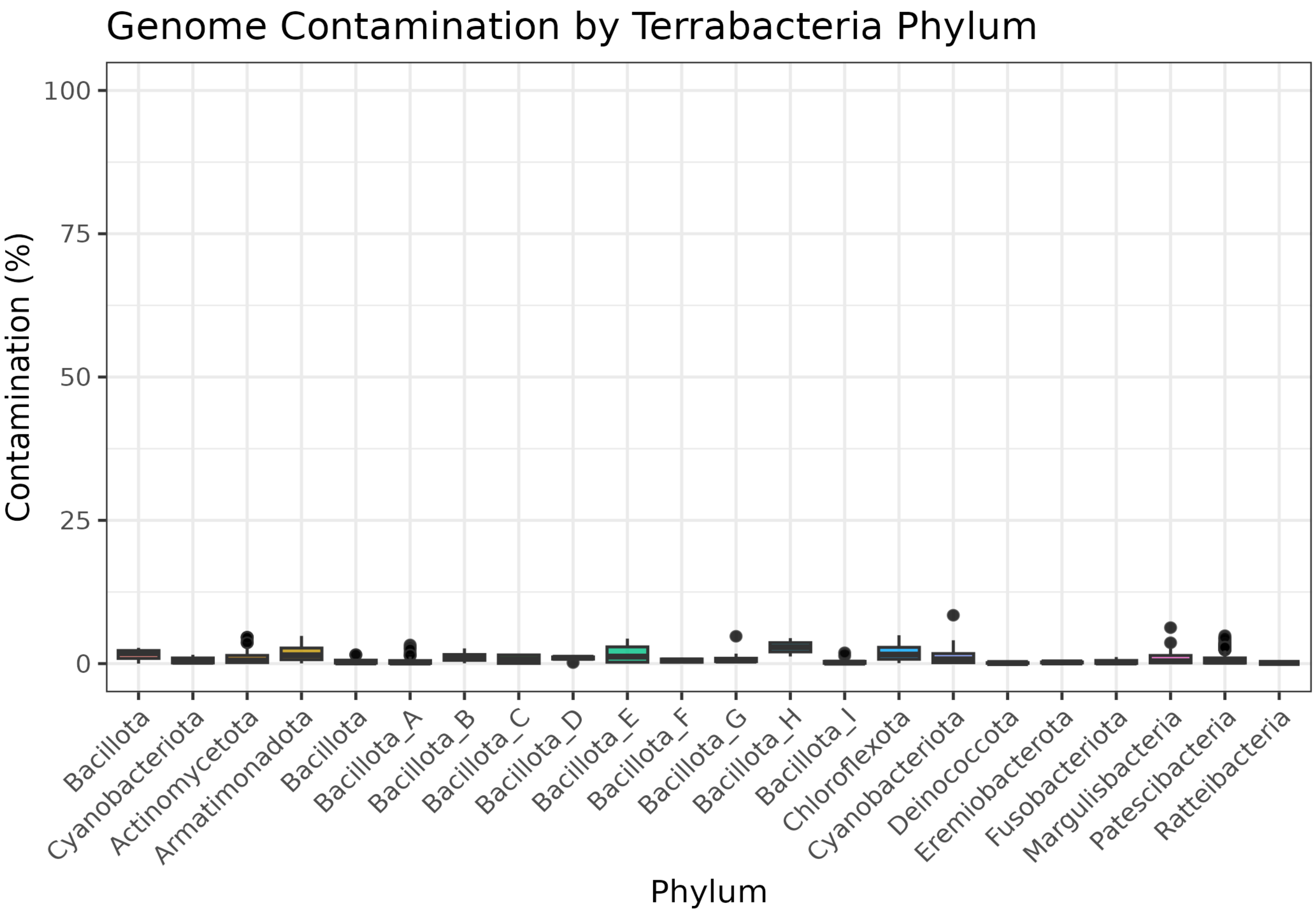

Genome Contamination by Terrabacteria Phylum
Contamination (%)
100
75
50
25
0
Bacillota
Cyanobacteriota
Actinomycetota
Armatimonadota
Bacillota
Bacillota_A
Bacillota_B
Bacillota_C
Bacillota_D
Bacillota_E
Bacillota_F
Bacillota_G
Bacillota_H
Bacillota_I
Chloroflexota
Cyanobacteriota
Deinococcota
Eremiobacterota
Fusobacteriota
Margulisbacteria
Patescibacteria
Ratteibacteria
Phylum

Fig. S4

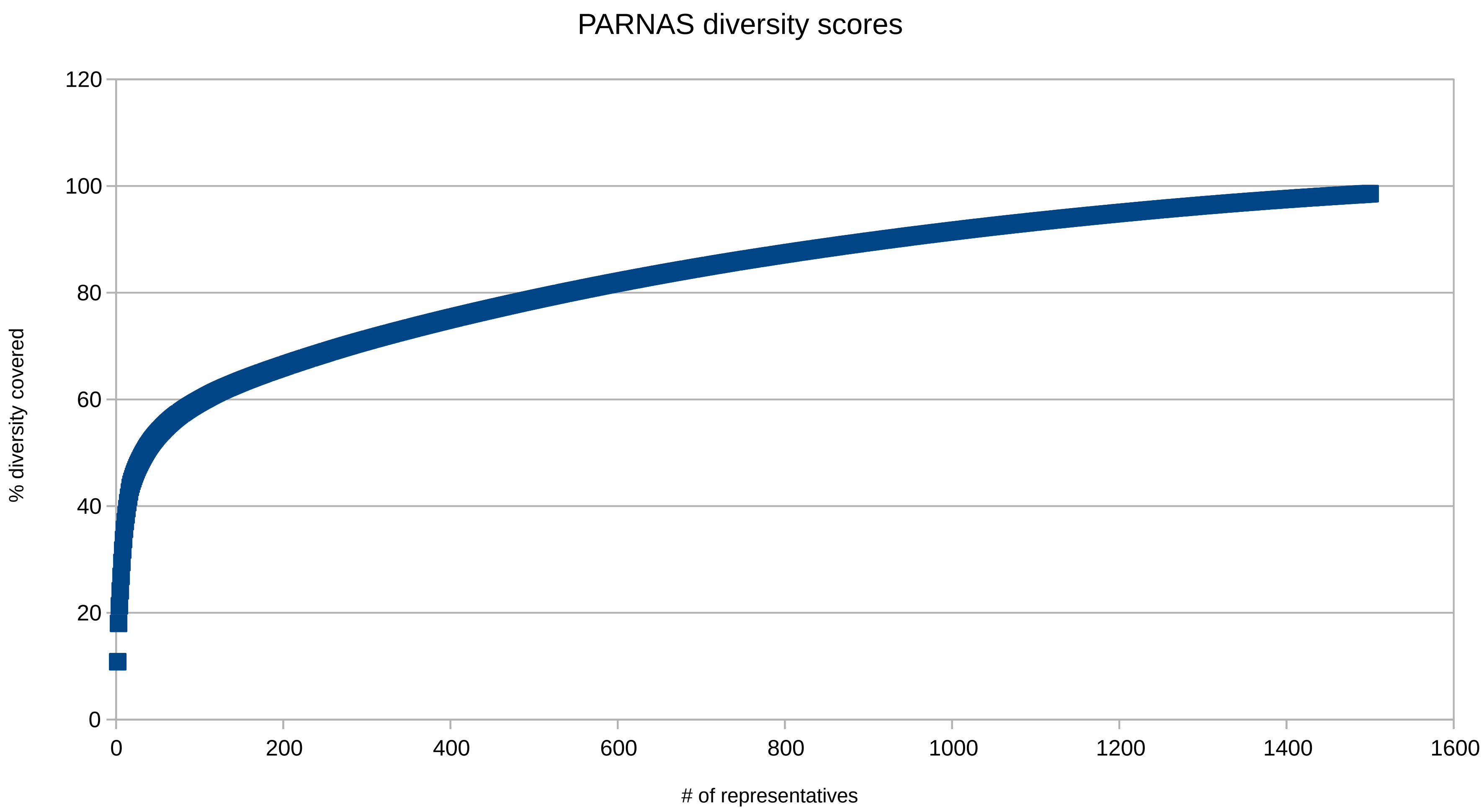
PARNAS diversity scores
% diversity covered
0
20
40
60
80
100
120
# of representatives
0
200
400
600
800
1000
1200
1400
1600

Fig. S5

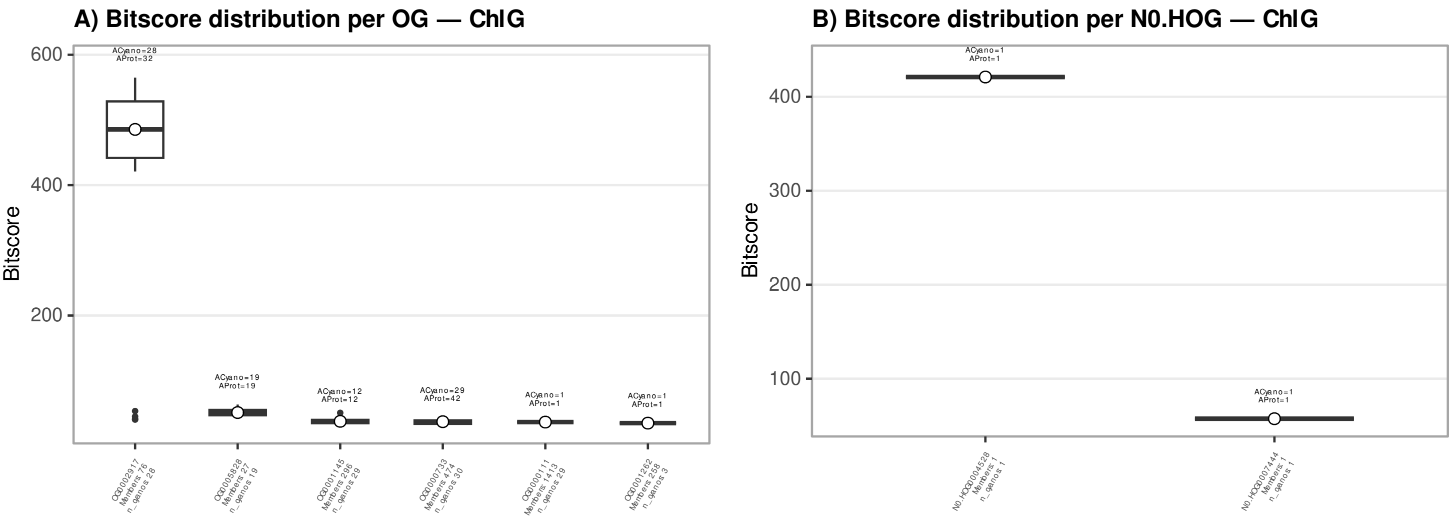

Fig. S6

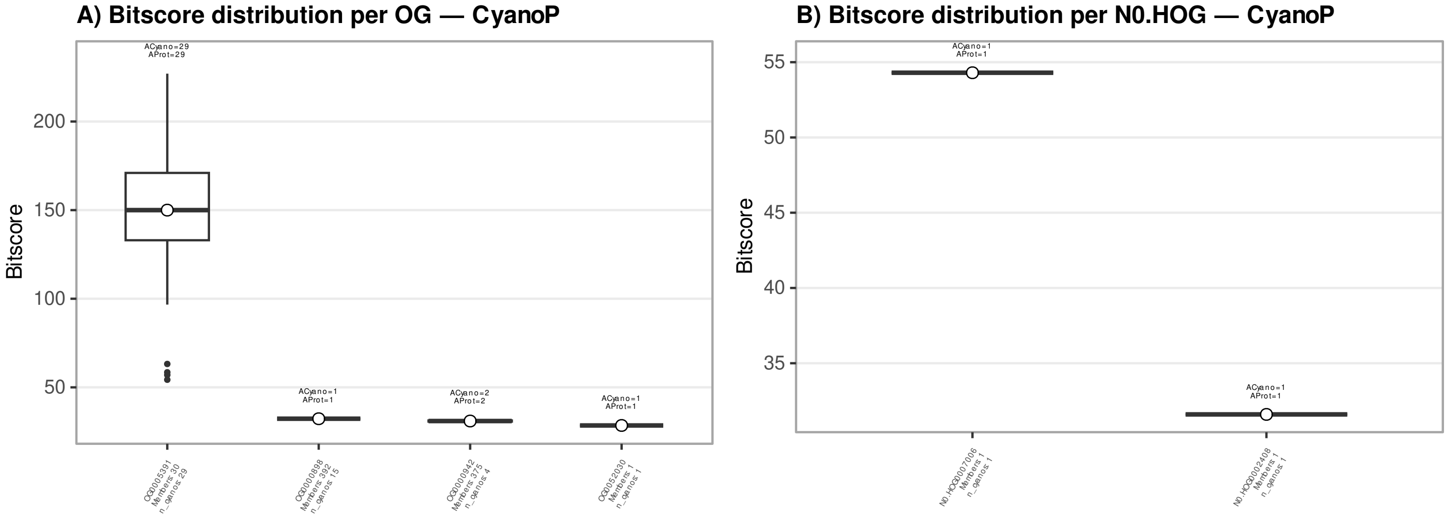

Fig. S7

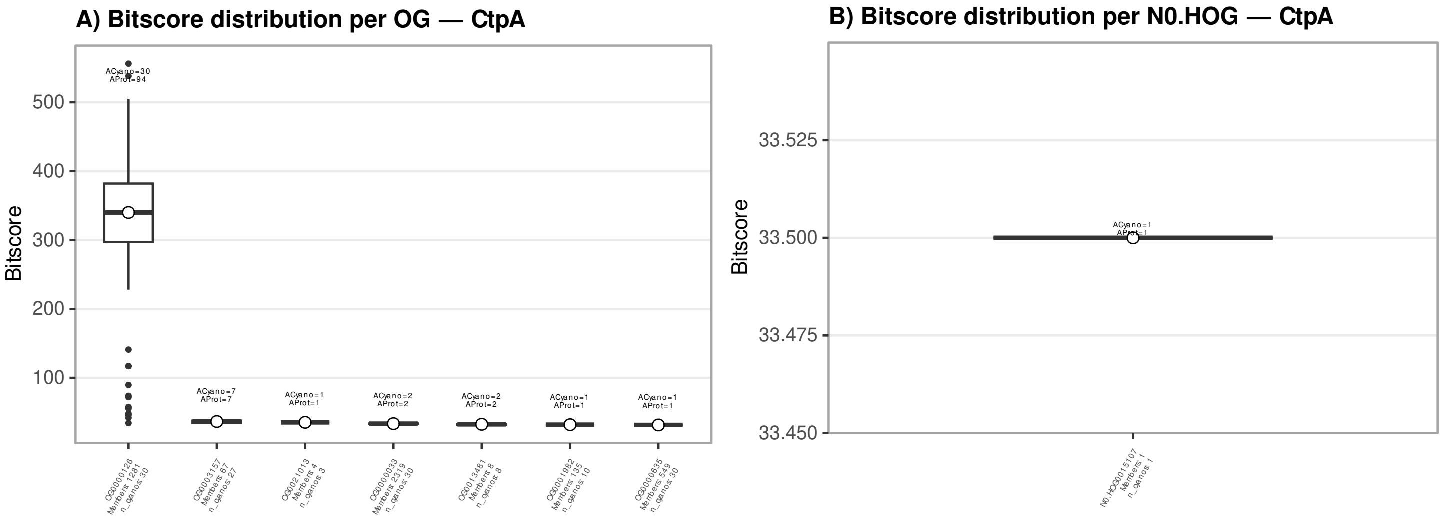

Fig. S8

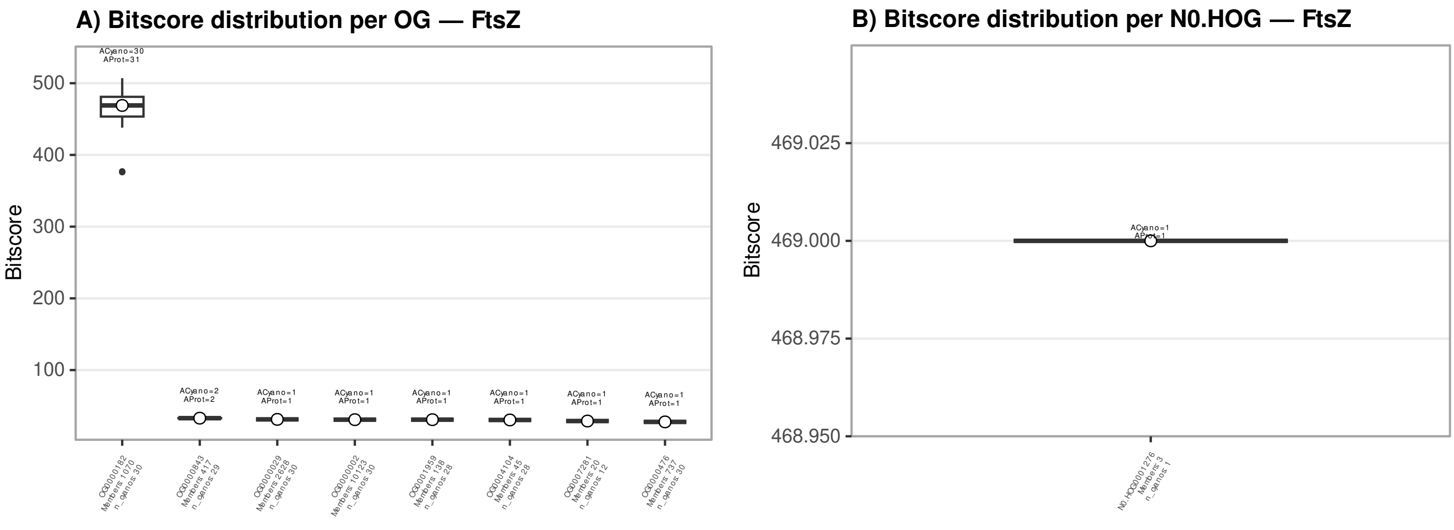

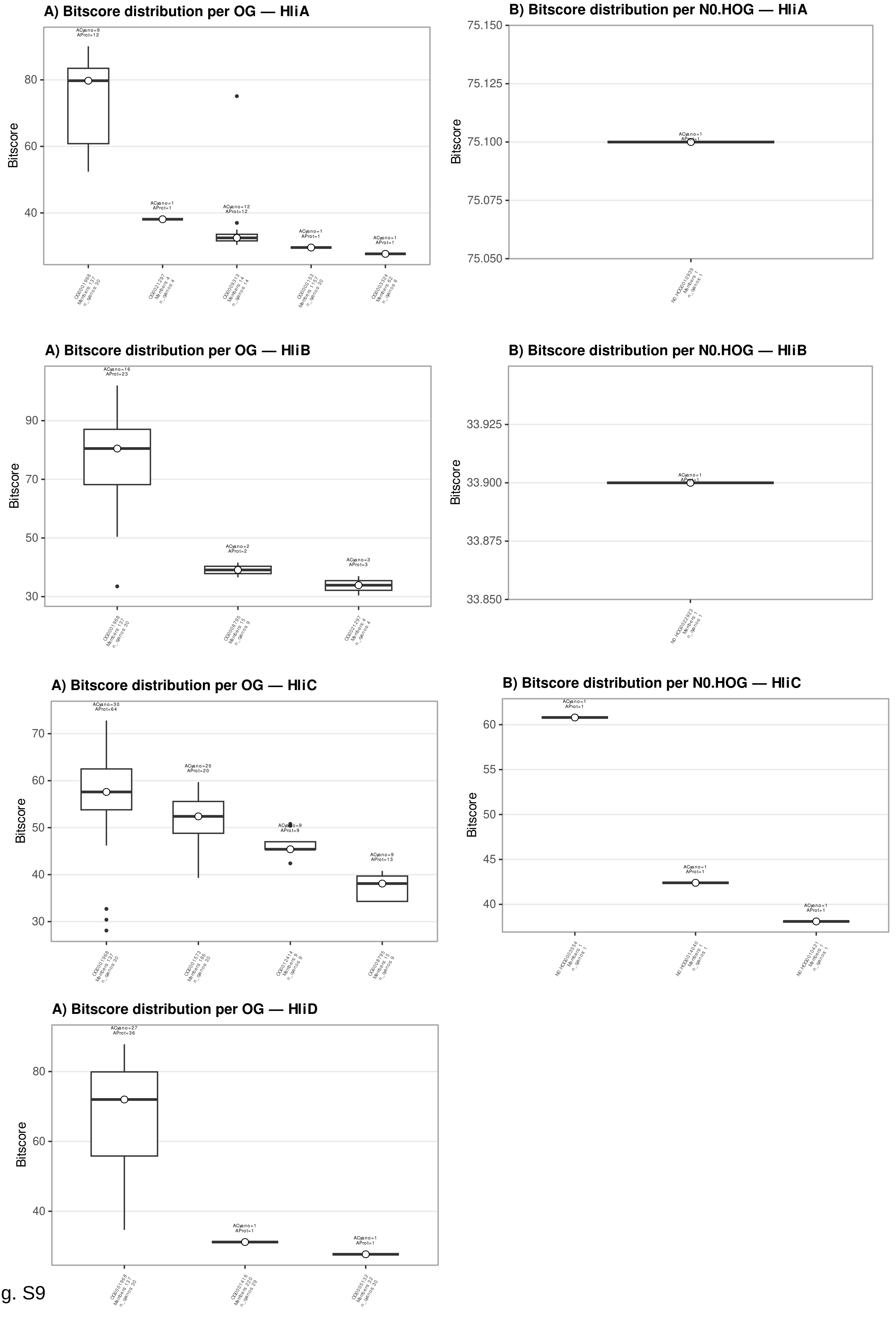

A) Bitscore distribution per OG — HliA
B) Bitscore distribution per N0.HOG — HliA
A) Bitscore distribution per OG — HliB
B) Bitscore distribution per N0.HOG — HliB
A) Bitscore distribution per OG — HliC
B) Bitscore distribution per N0.HOG — HliC
A) Bitscore distribution per OG — HliD
Bitscore

Fig. S9

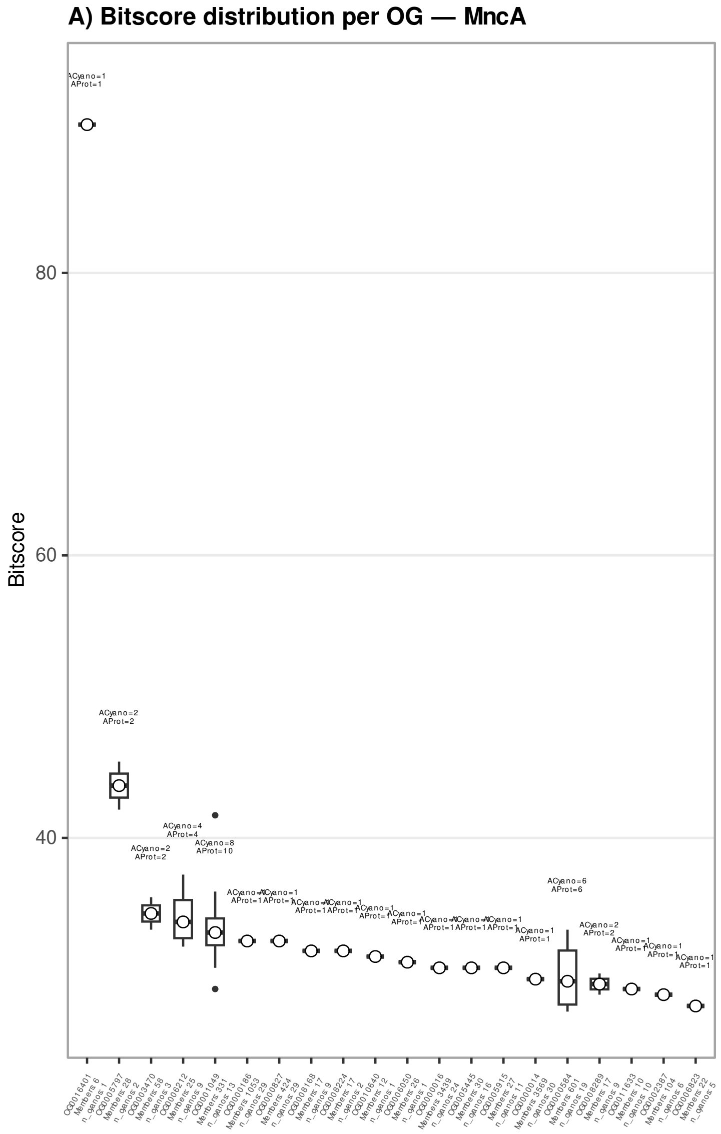

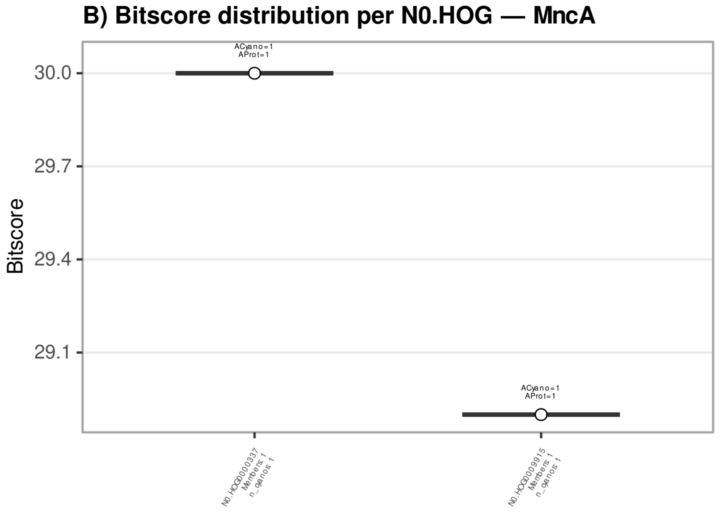

Fig. S9

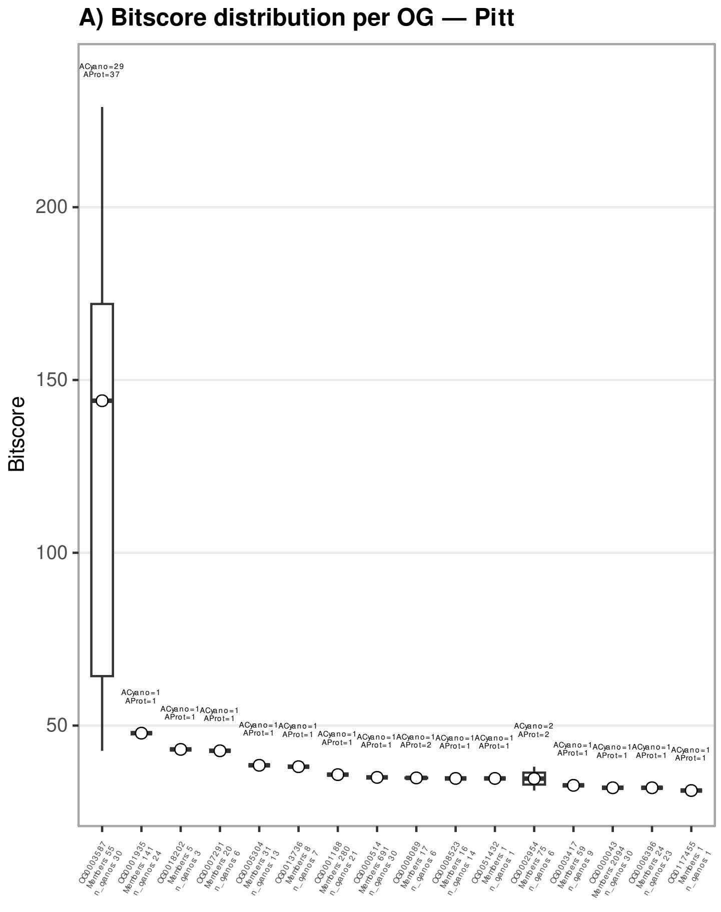

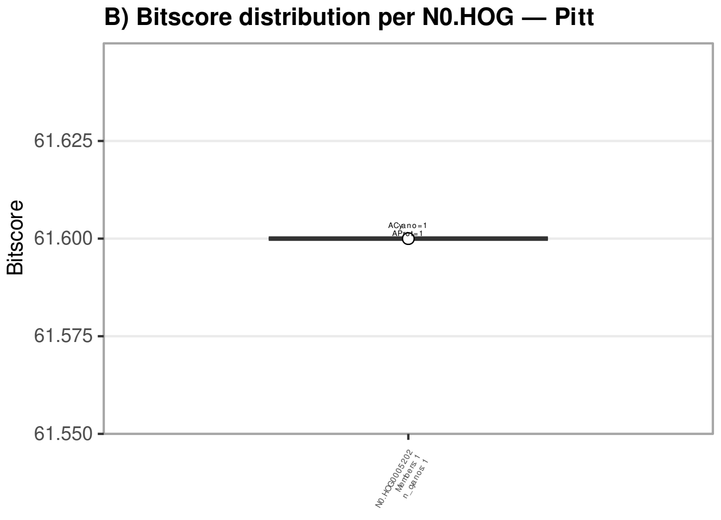

Fig. S10

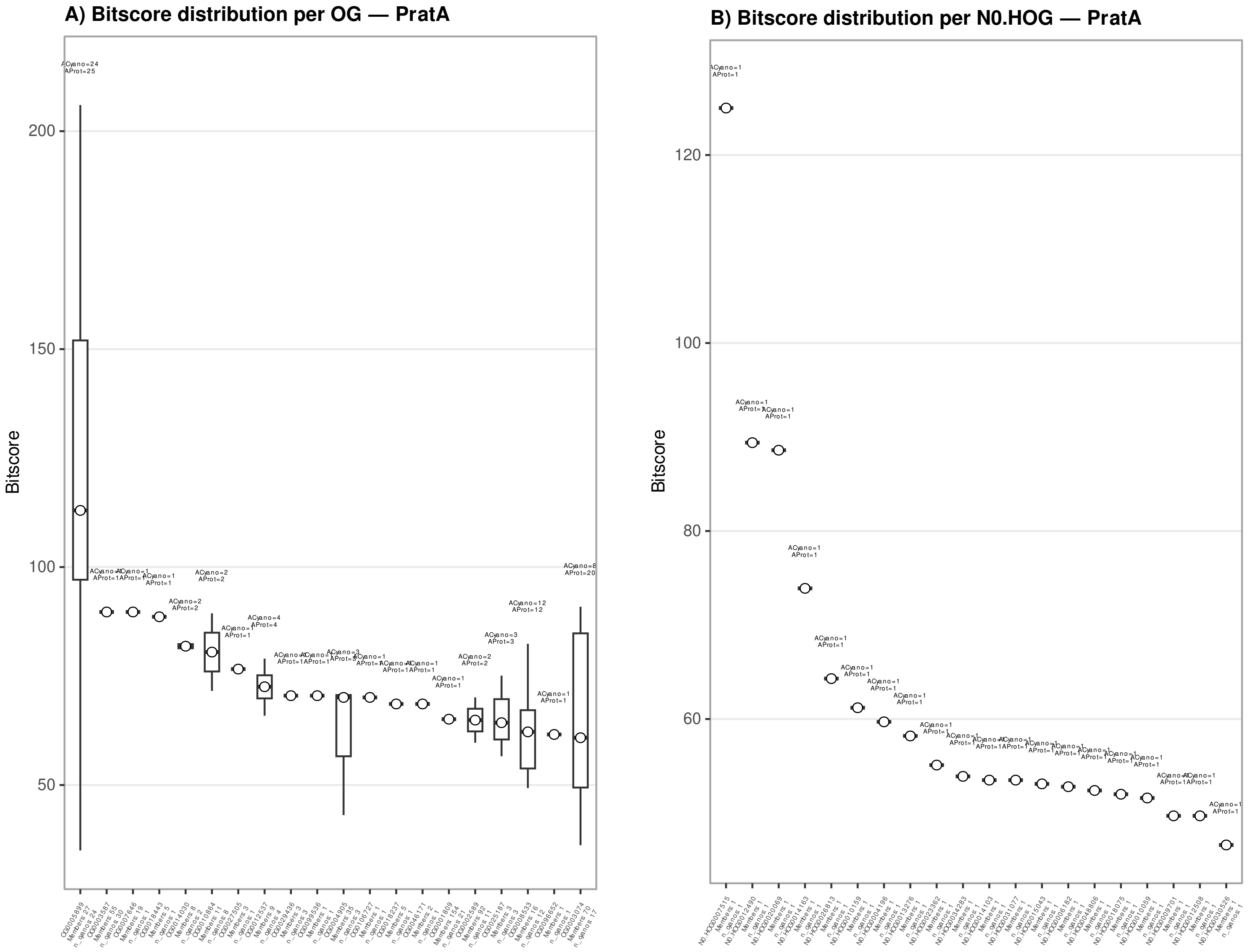

Fig. S11

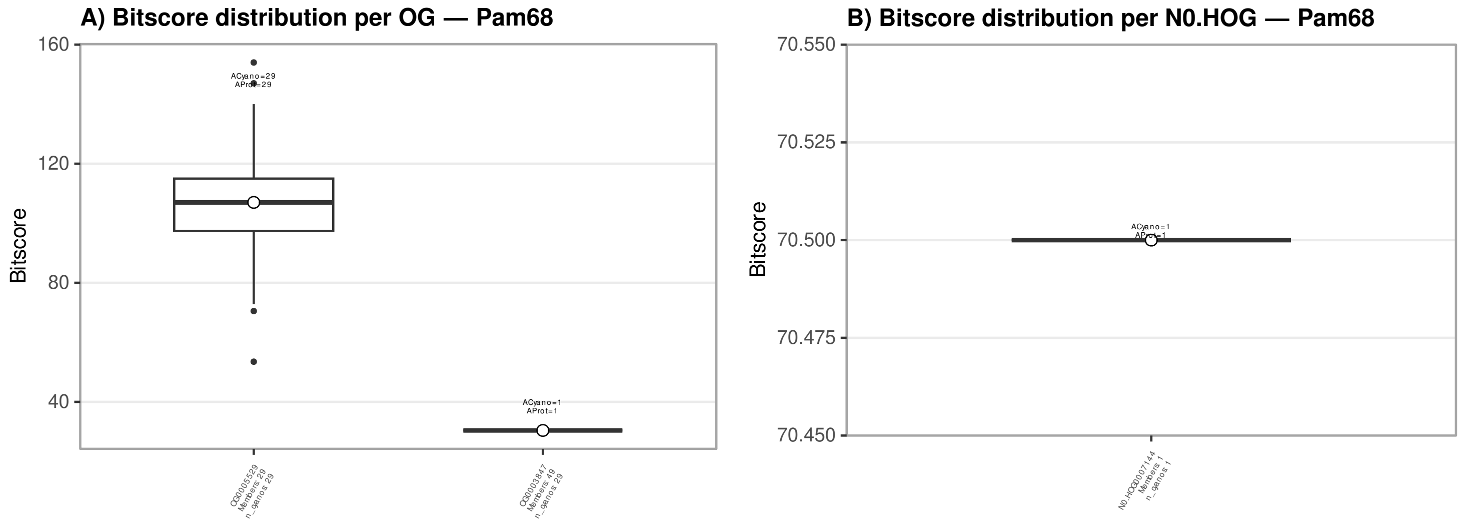

Fig. S12

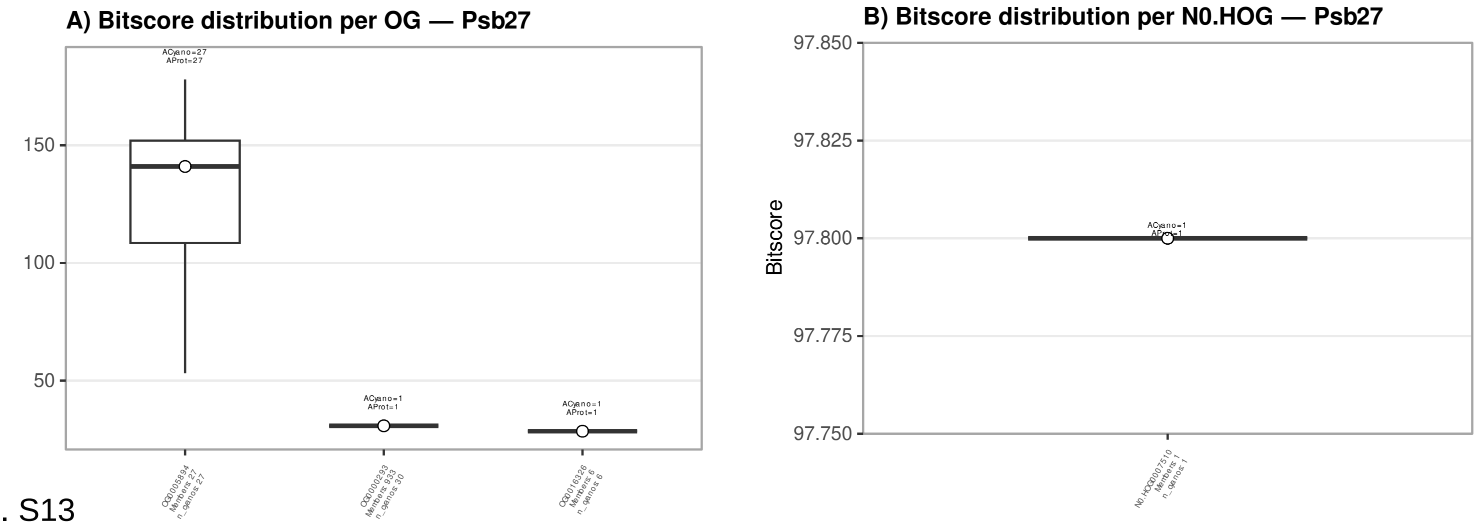

Fig. S13

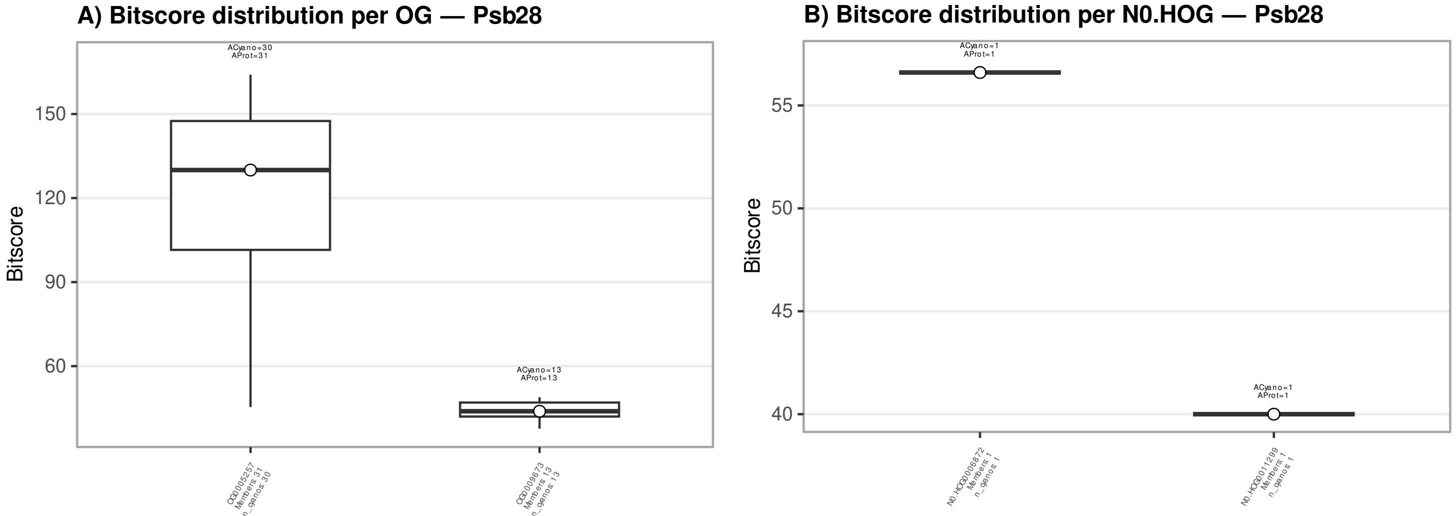

Fig. S14

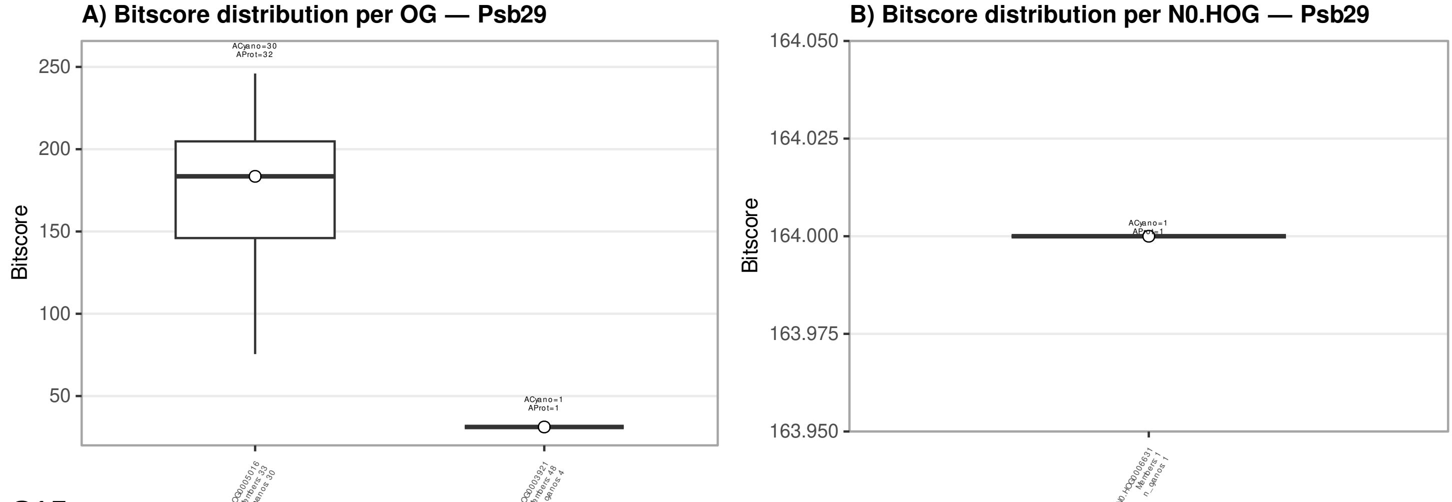

Fig. S15

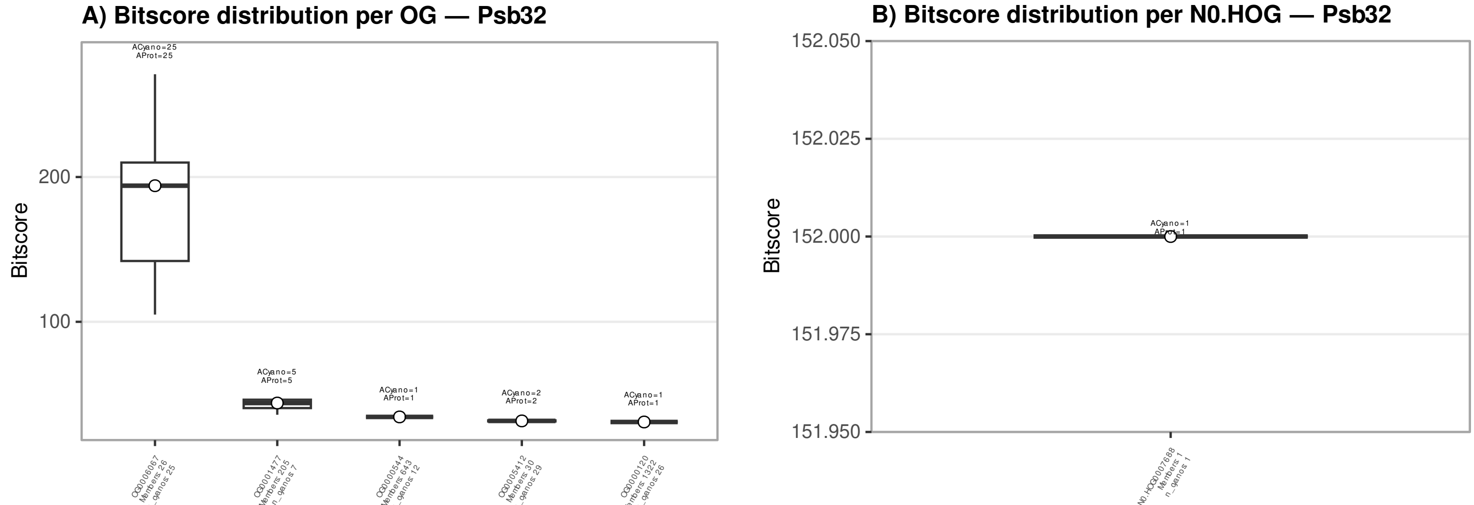

Fig. S16

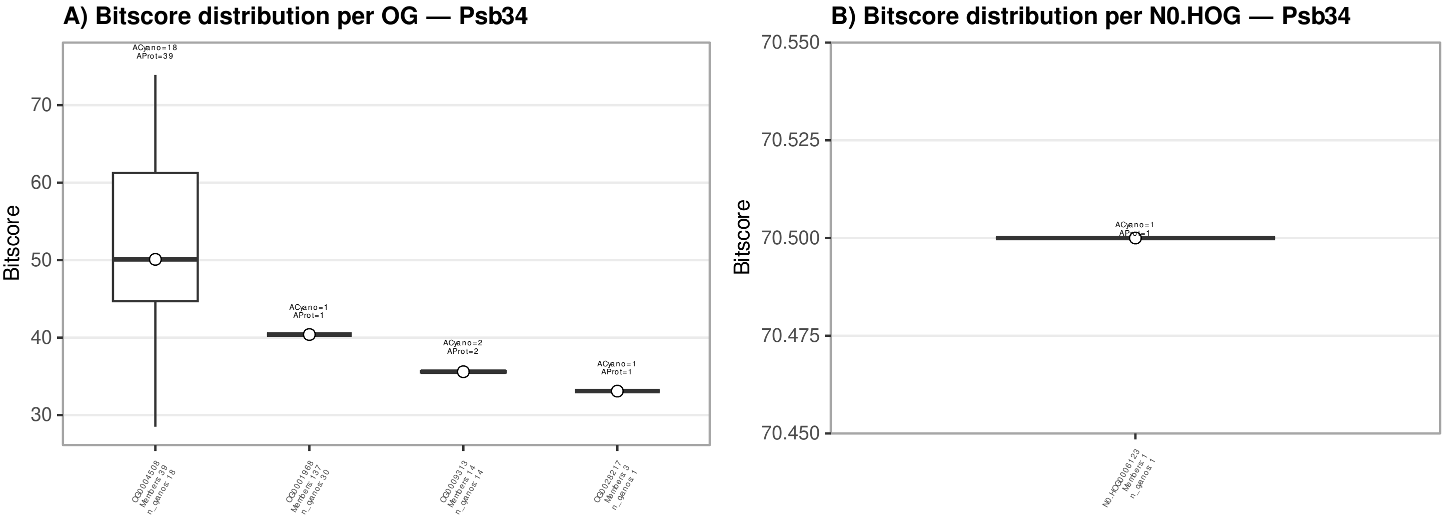

Fig. S17

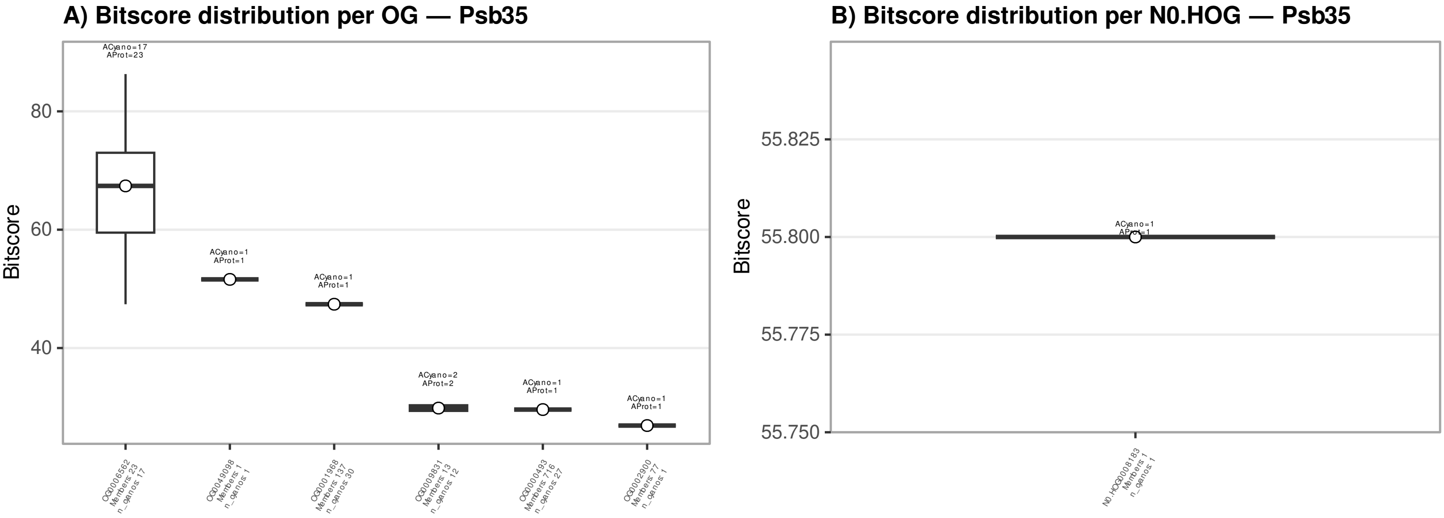

Fig. S18

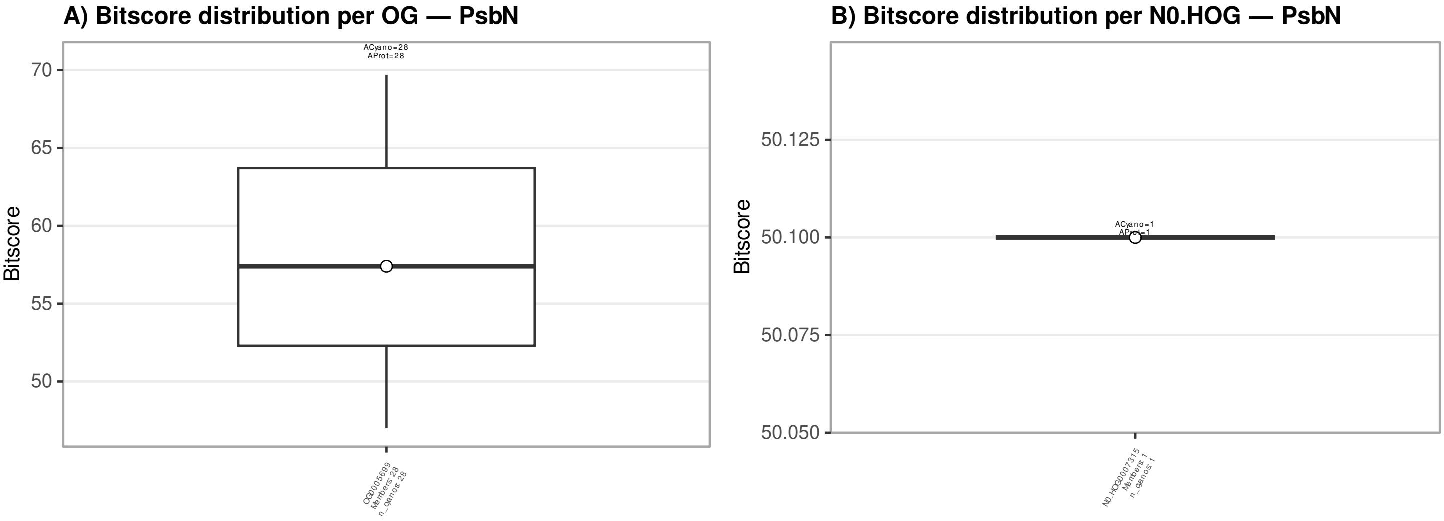

Fig. S19

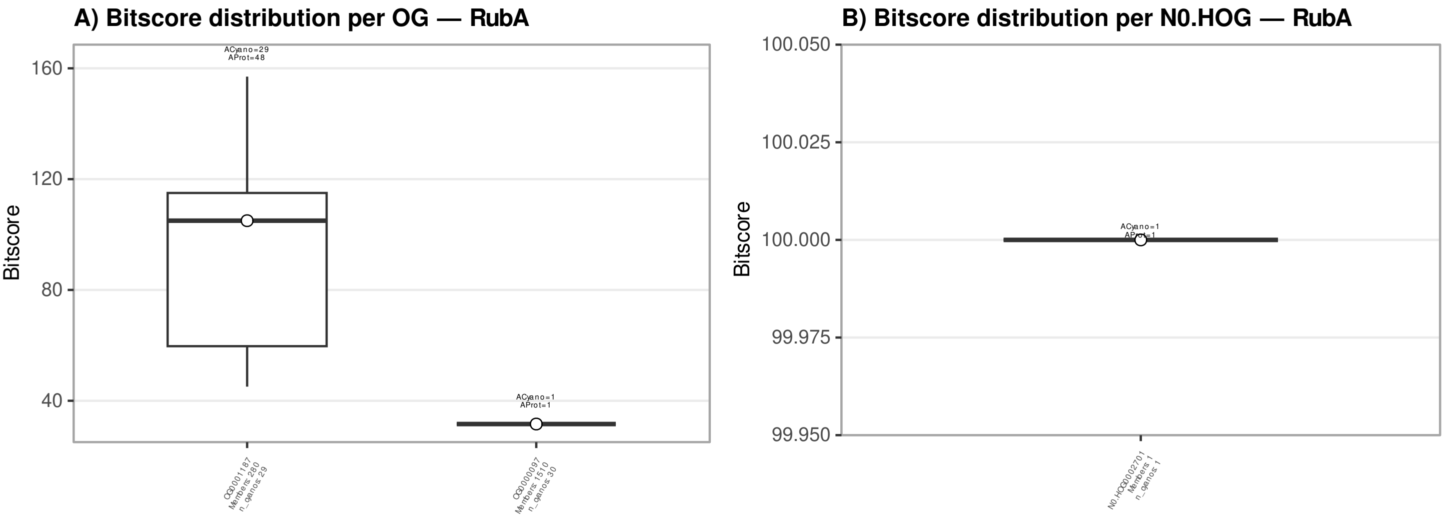

Fig. S20

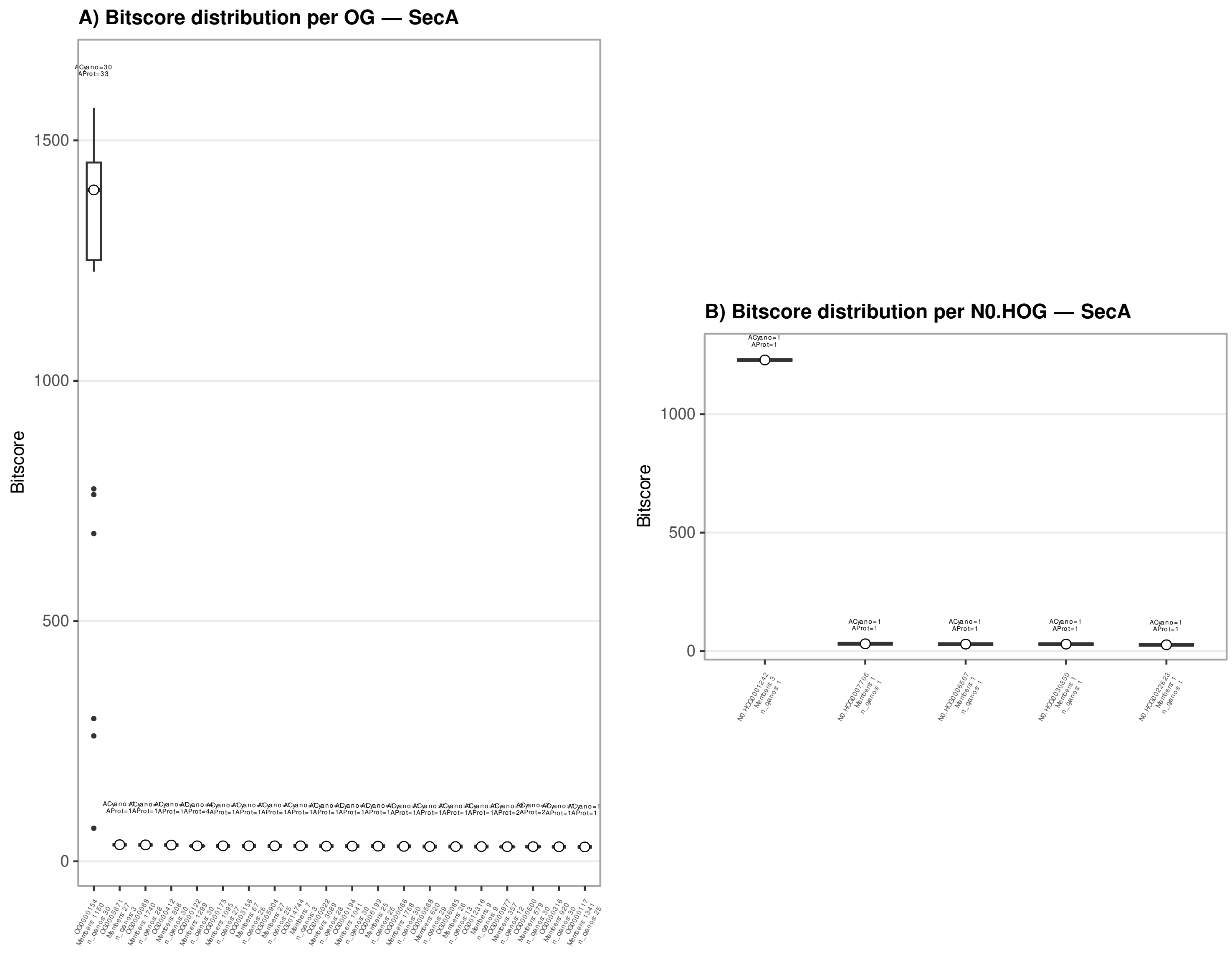

Fig. S21

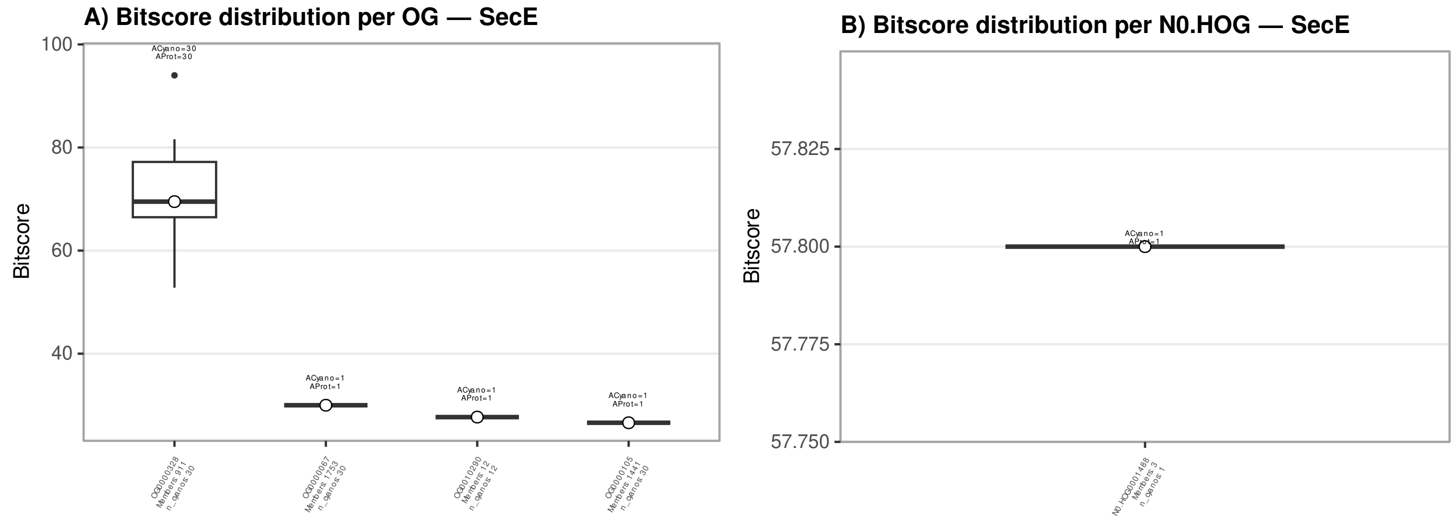

Fig. S22

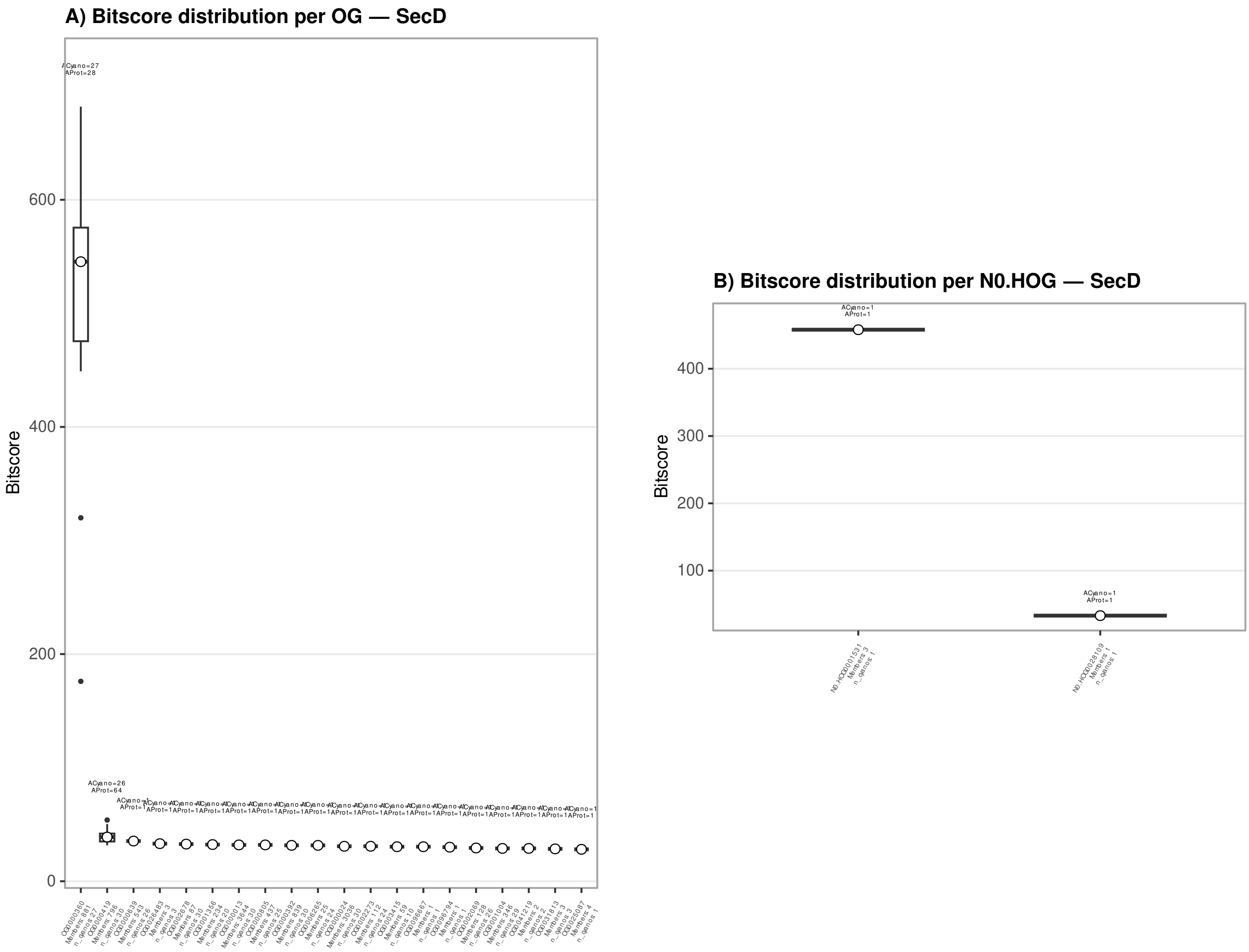

Fig. S23

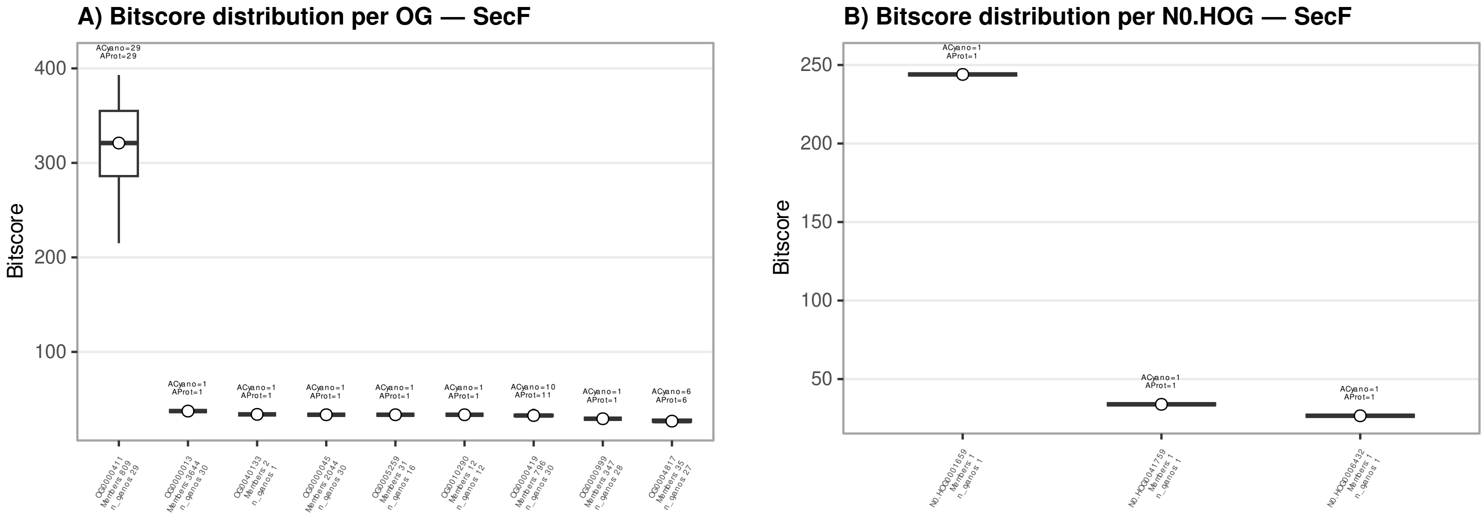

Fig. S24

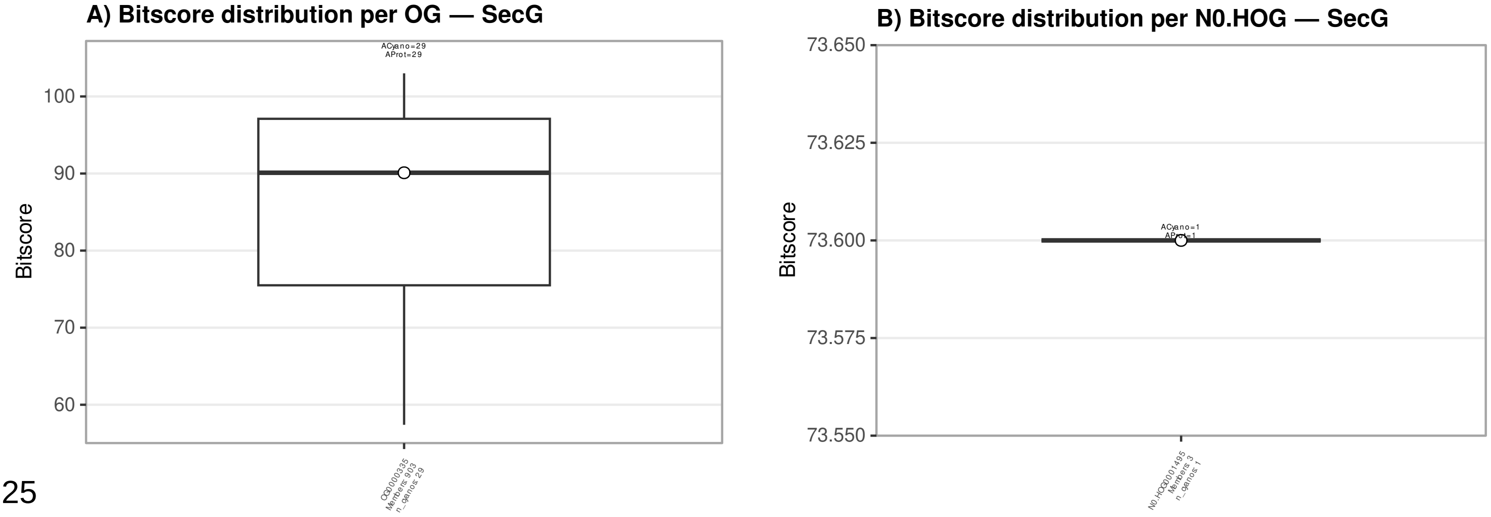

Fig. S25

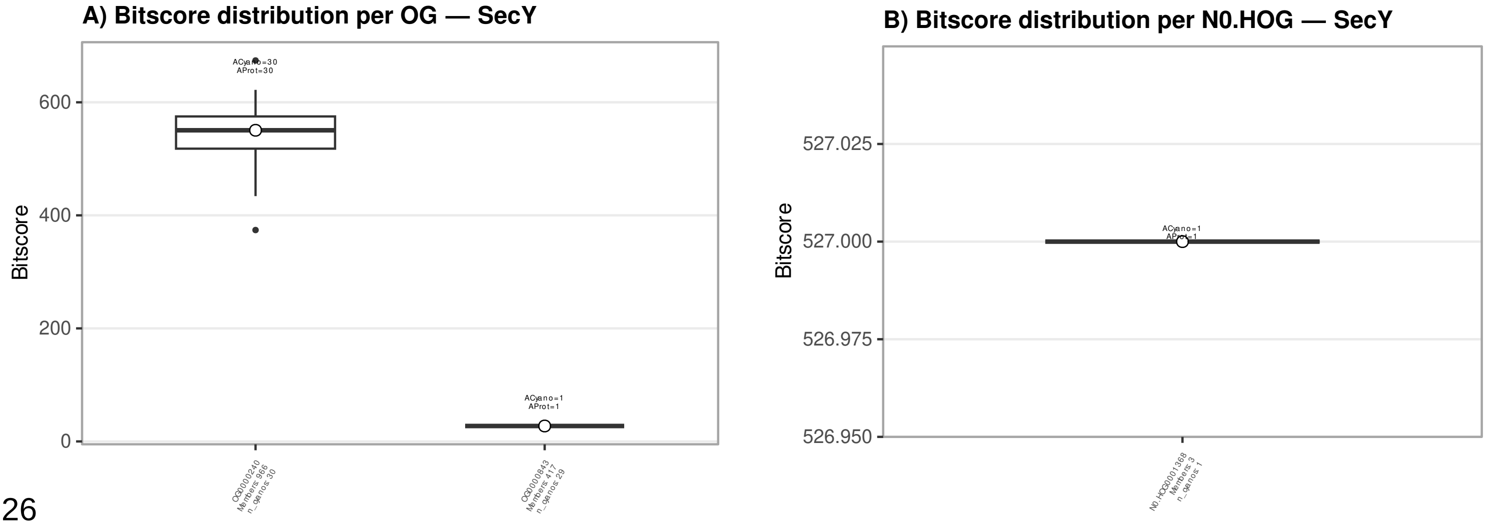

Fig. S26

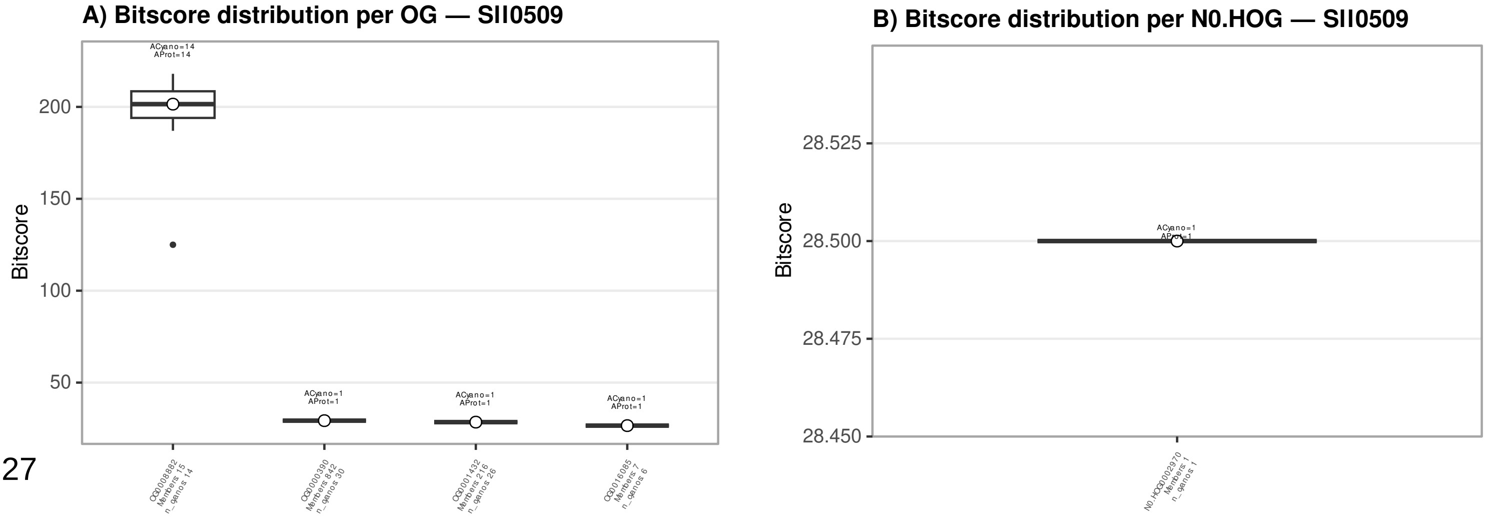

Fig. S27

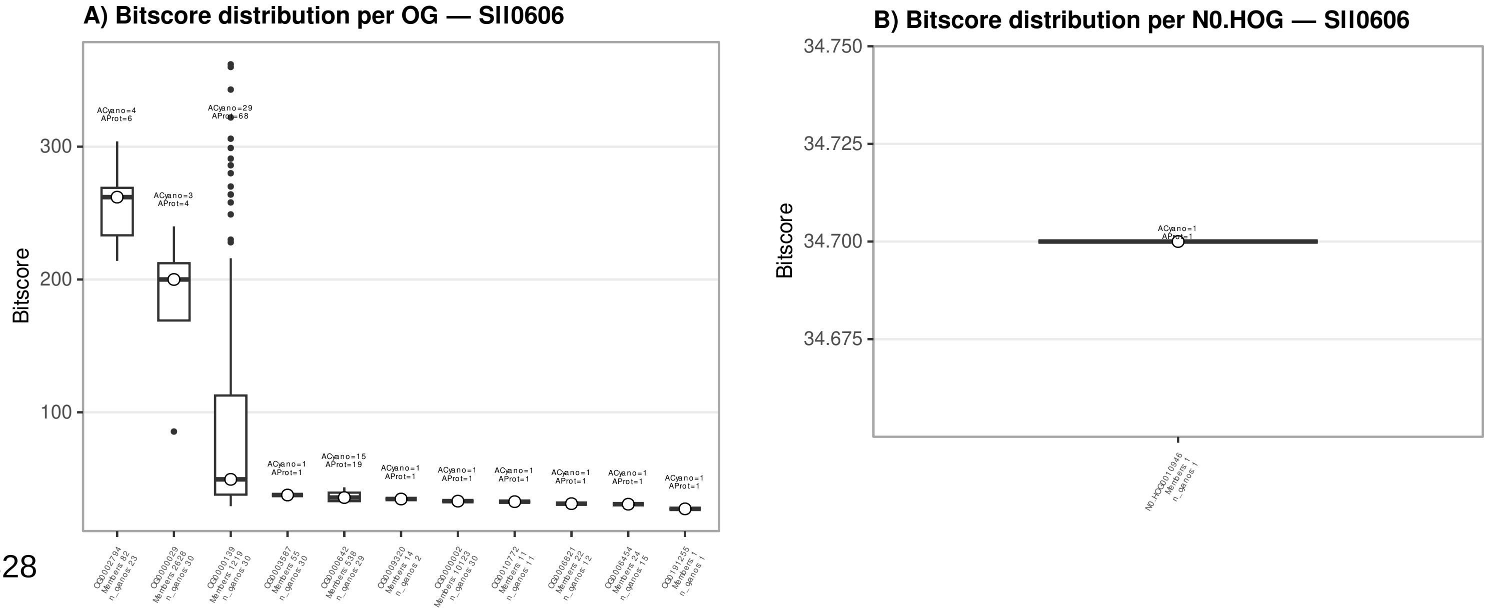

Fig. S28

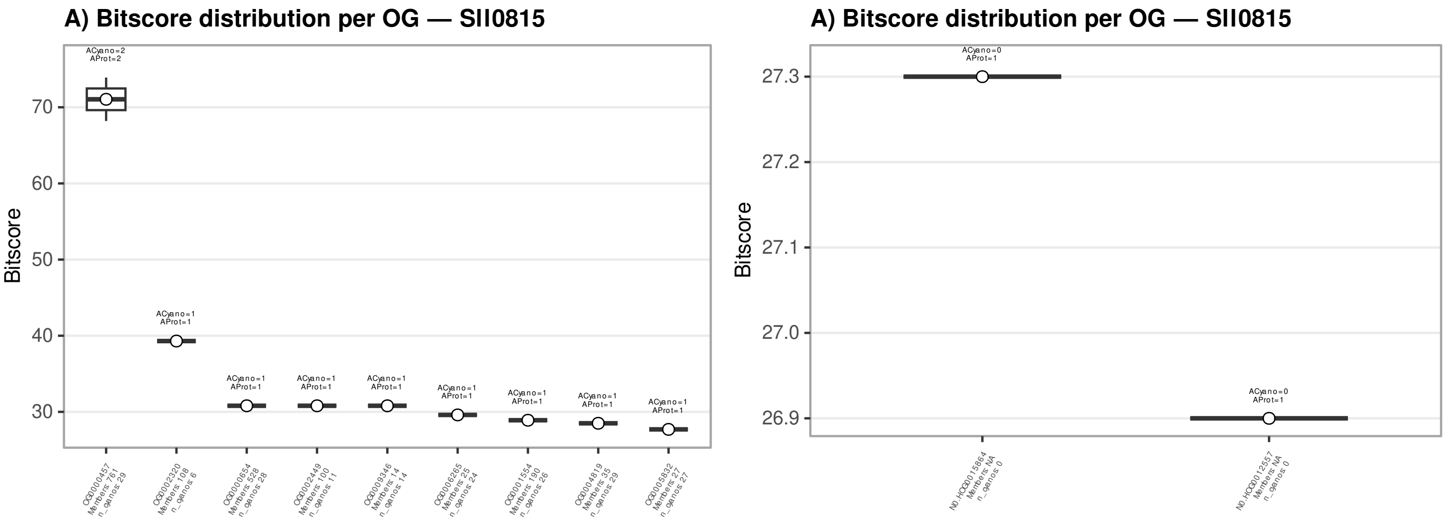

Fig. S29

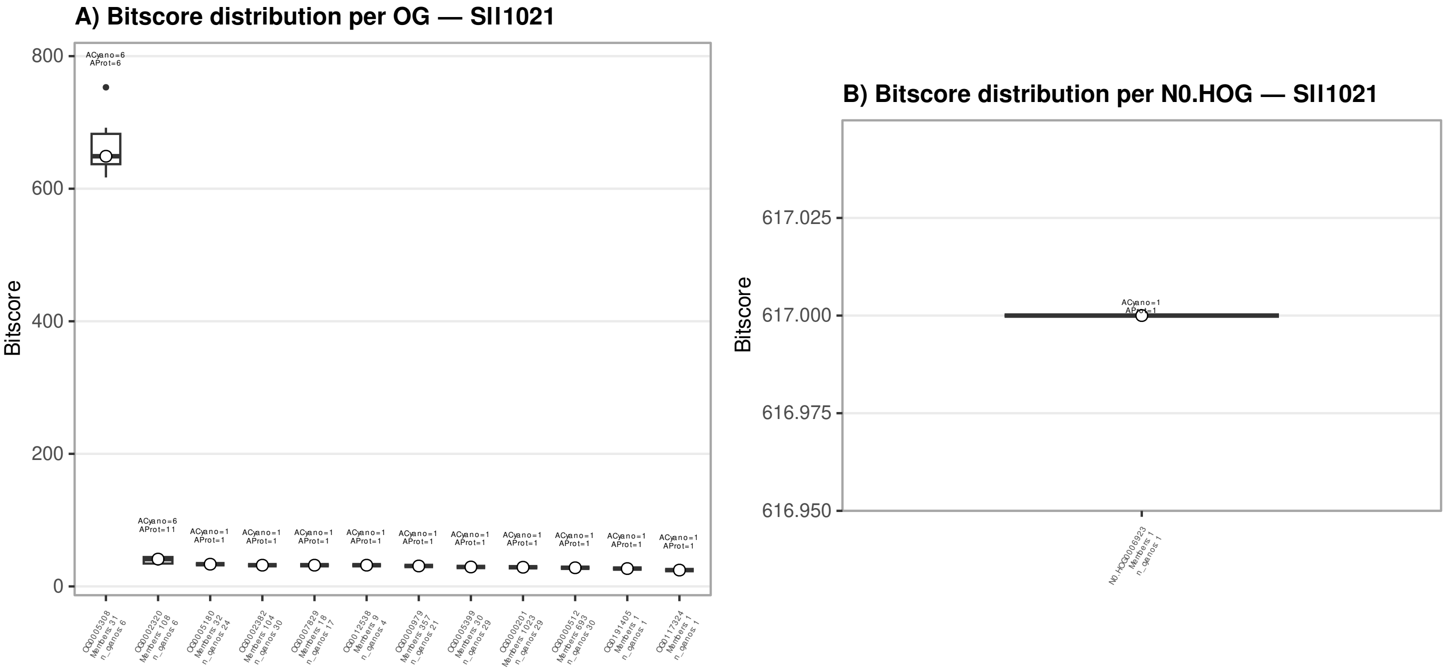

Fig. S30

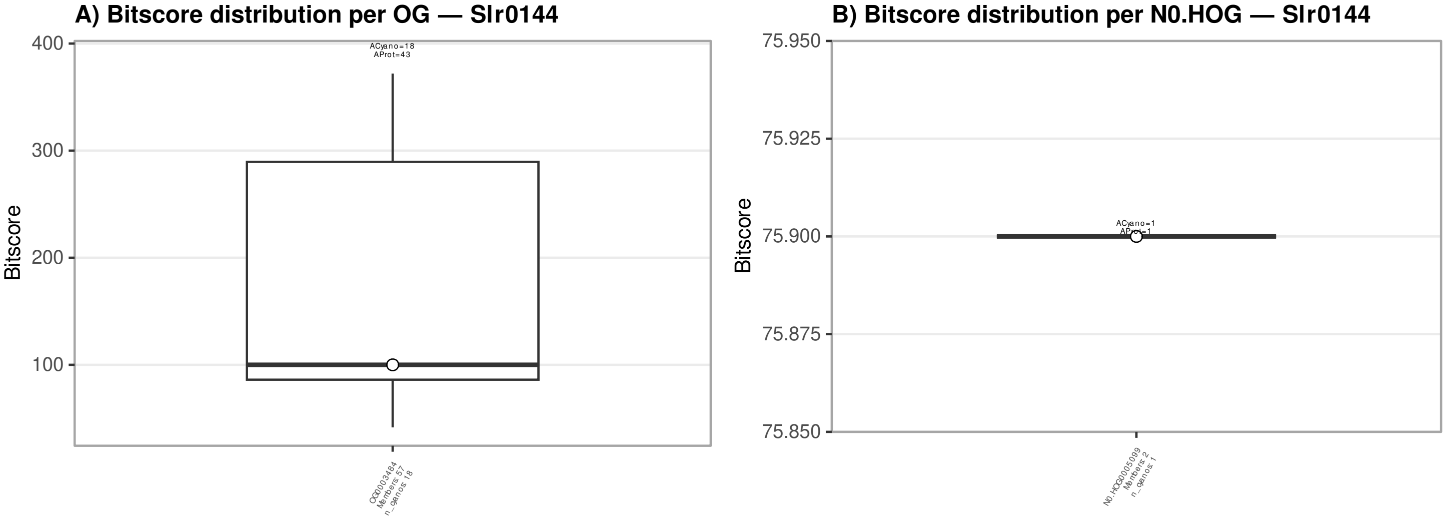

Fig. S31

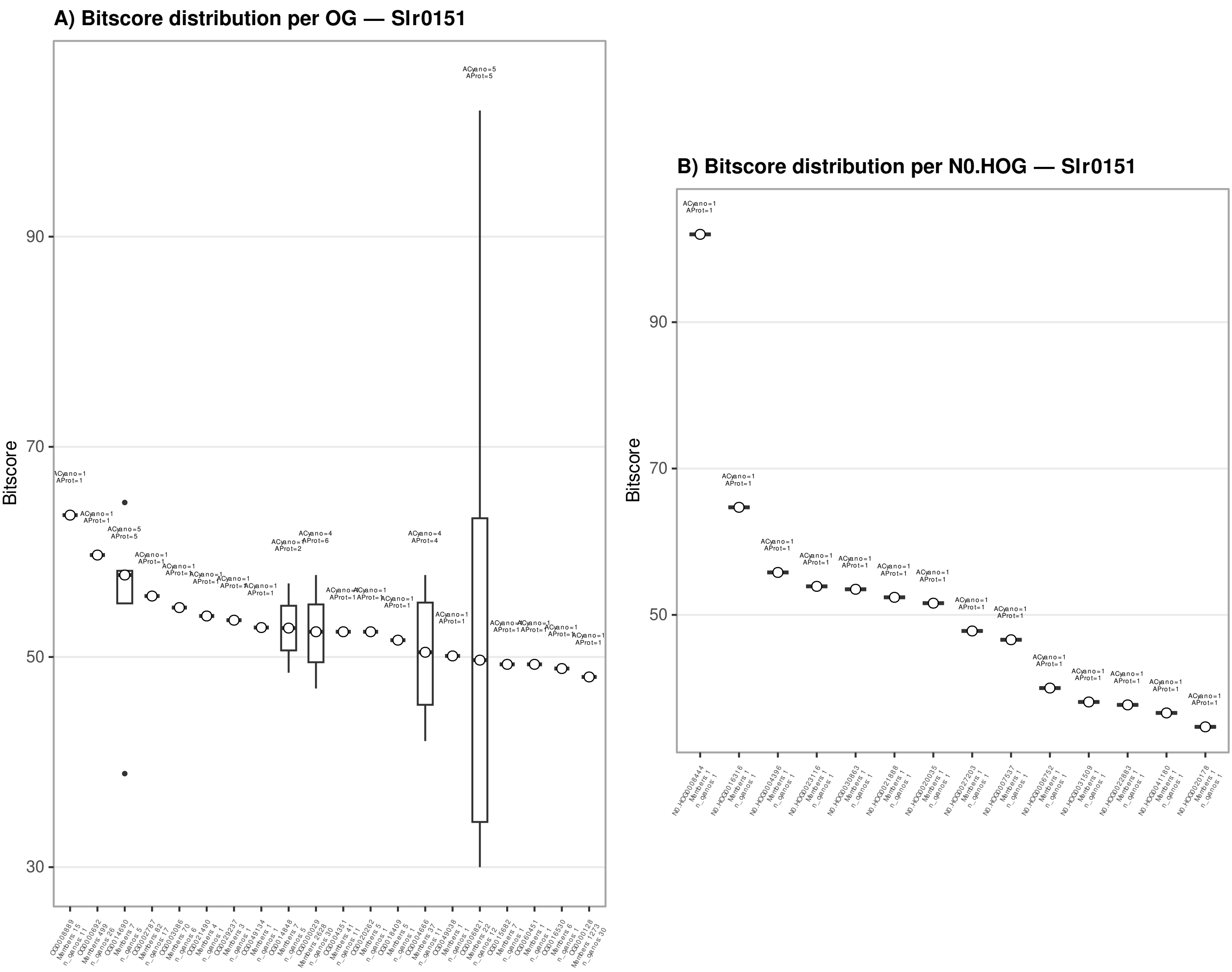

Fig. S32

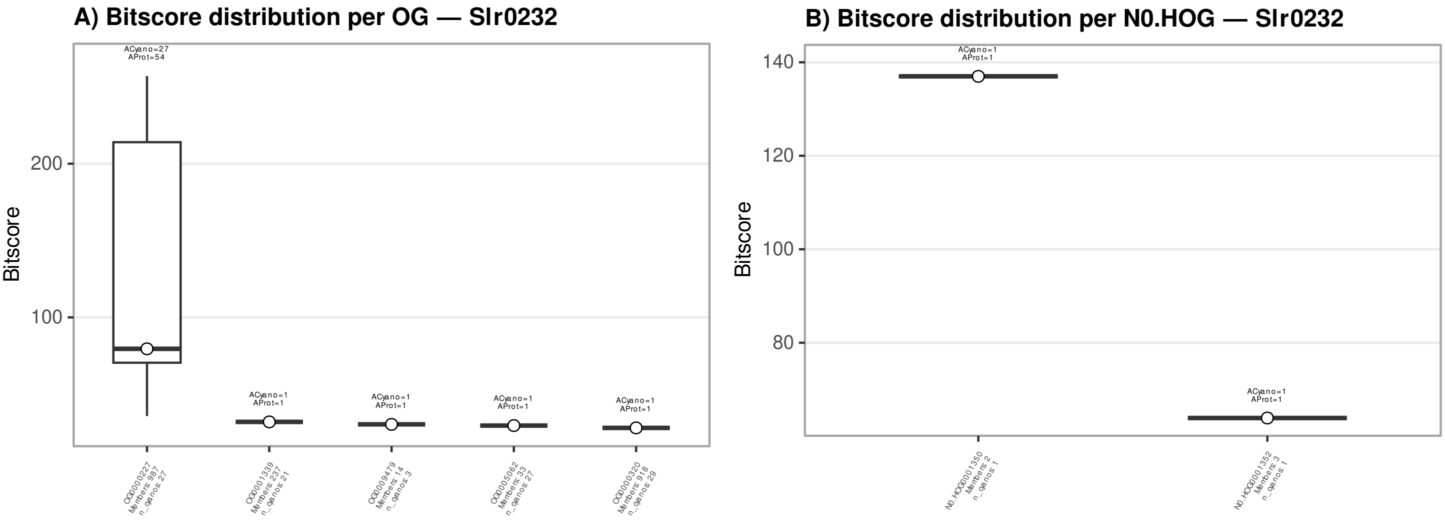

Fig. S33

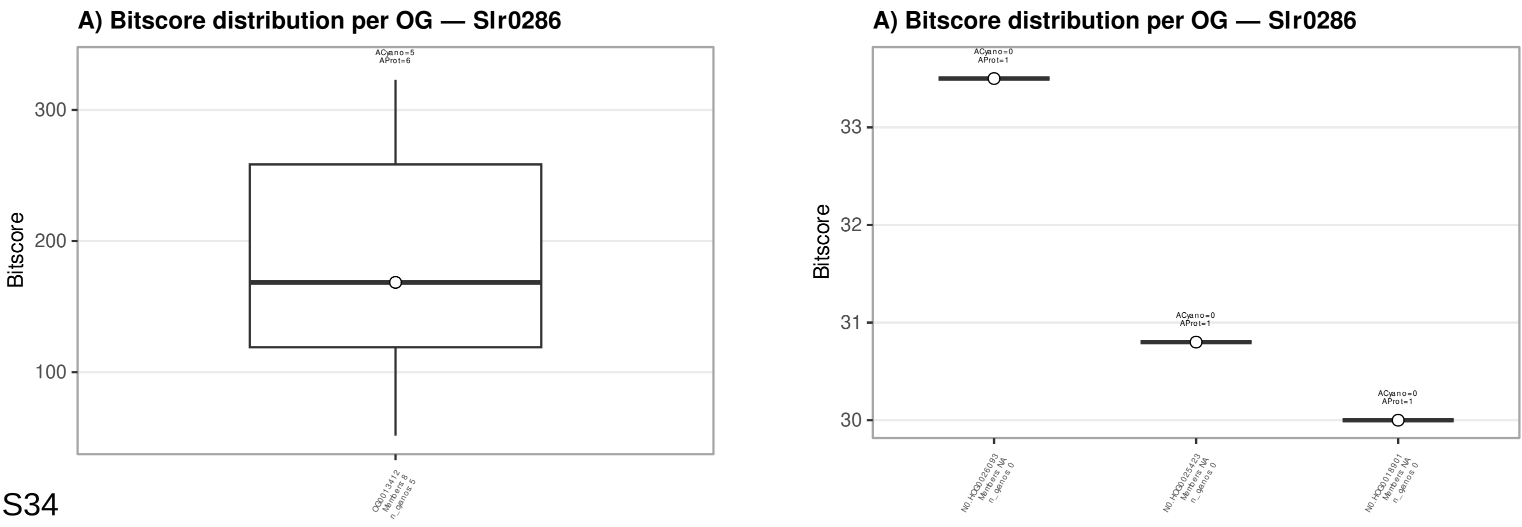

Fig. S34

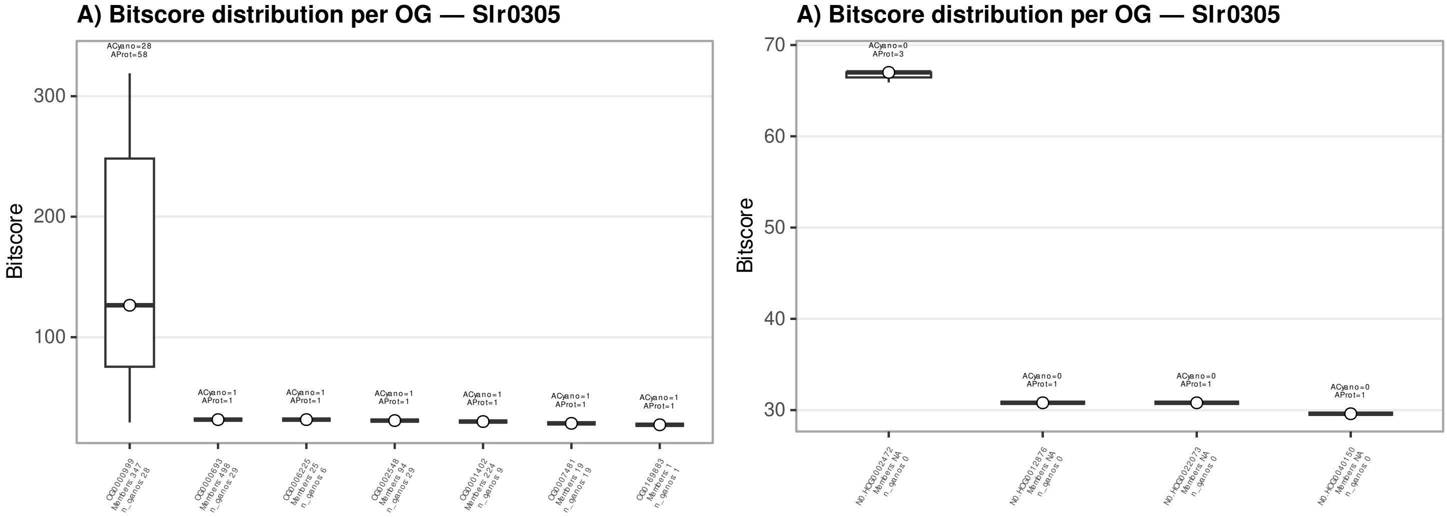

Fig. S35

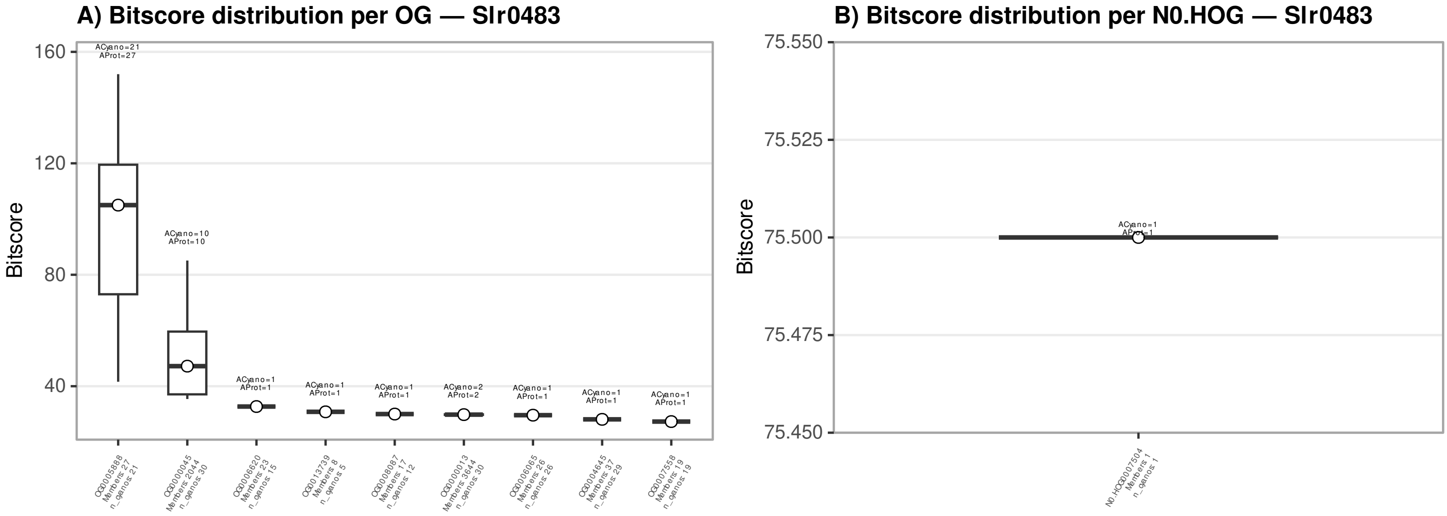

Fig. S36

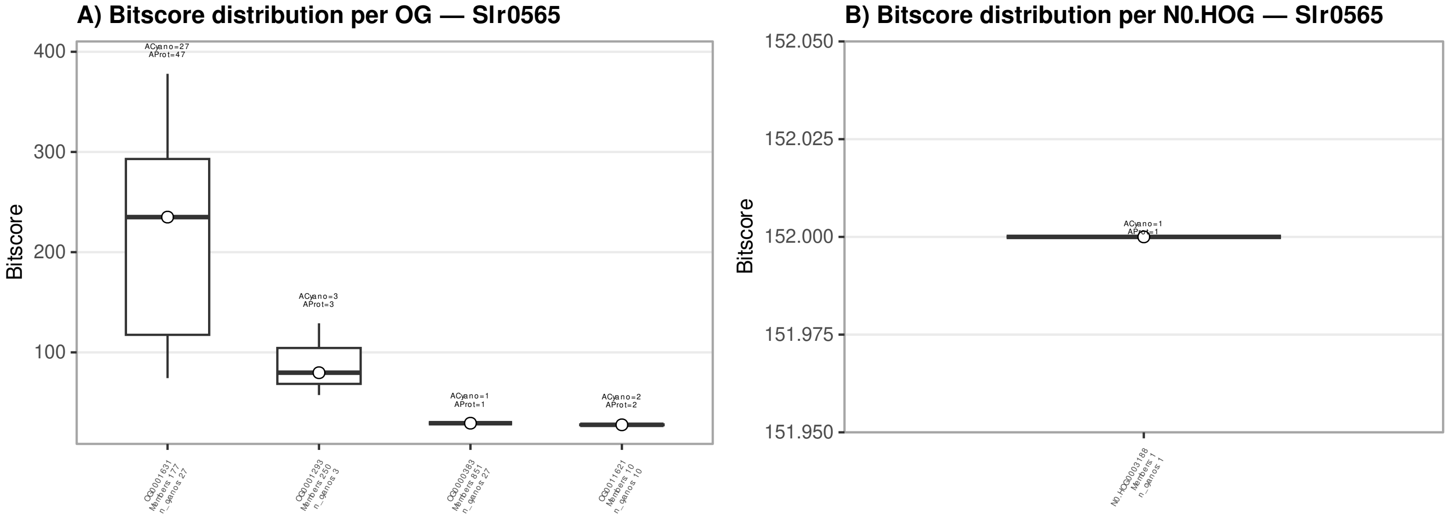

Fig. S37

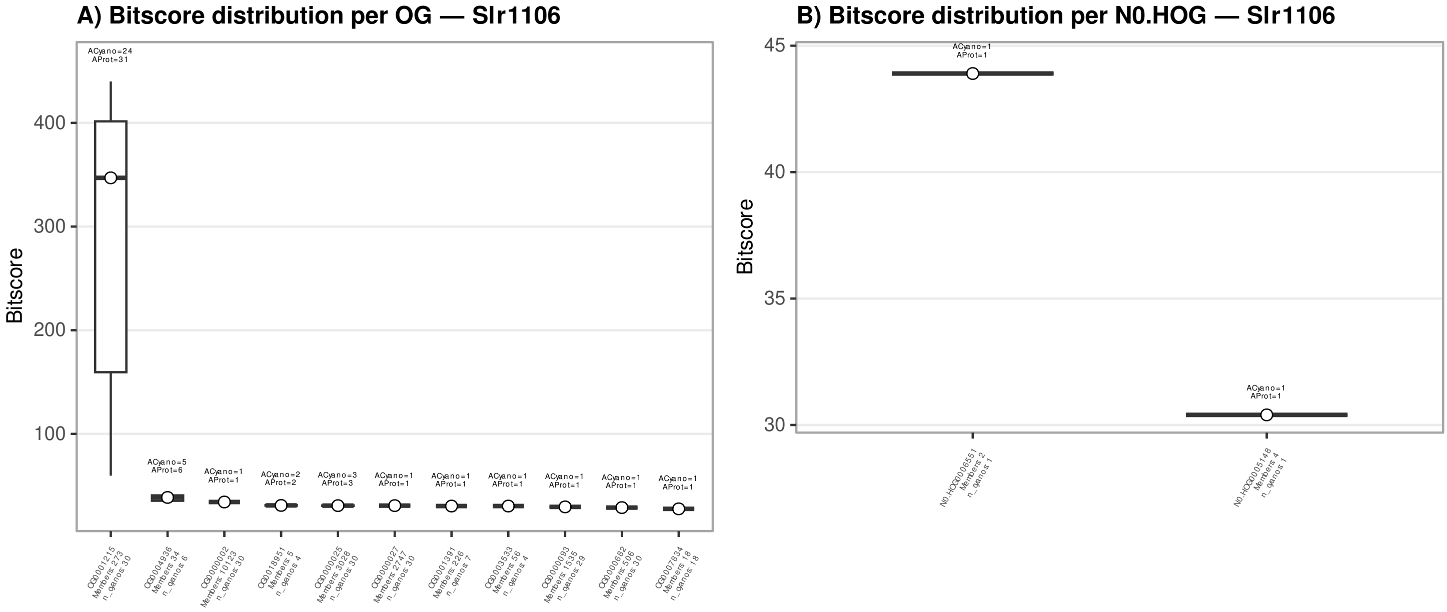

Fig. S38

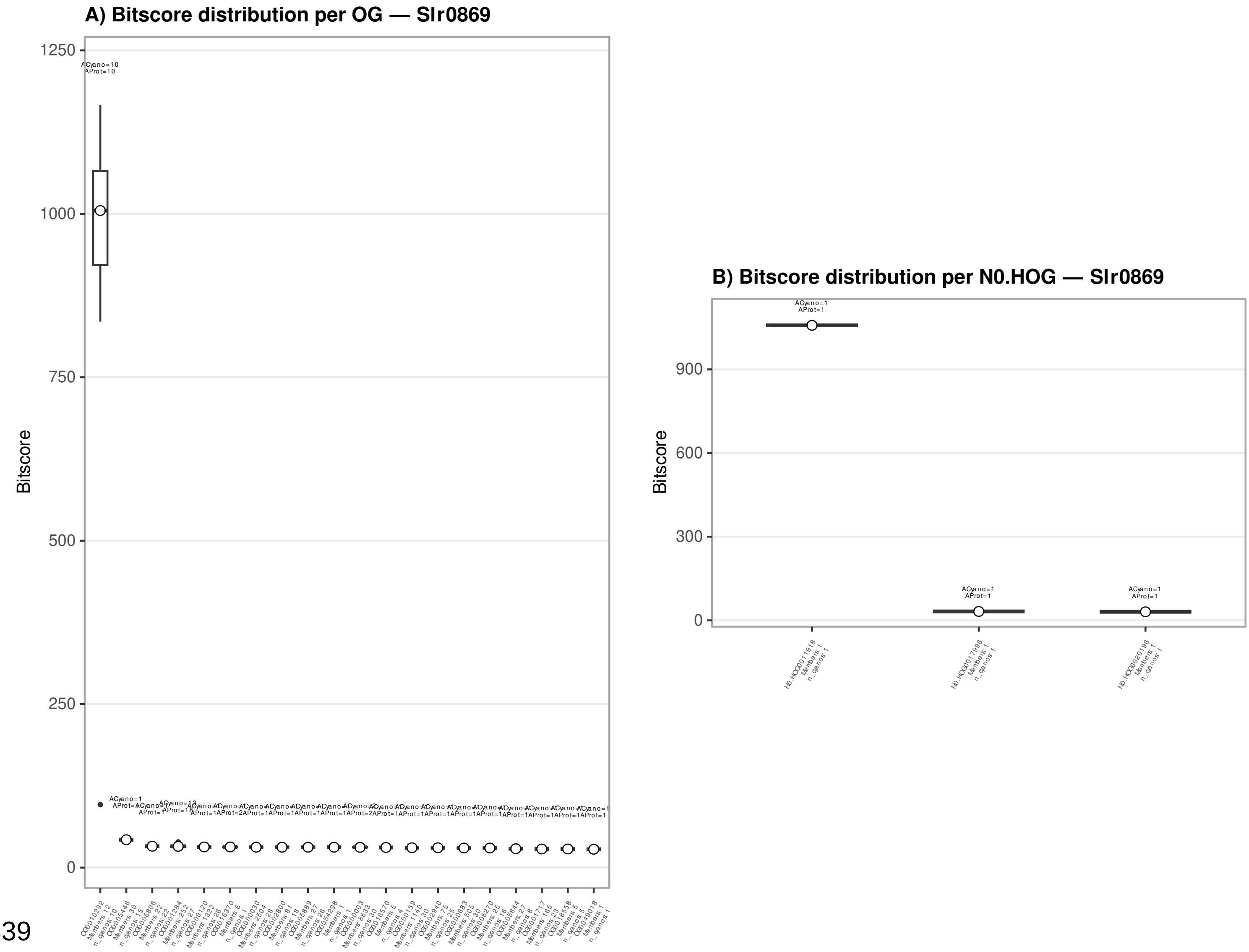

Fig. S39

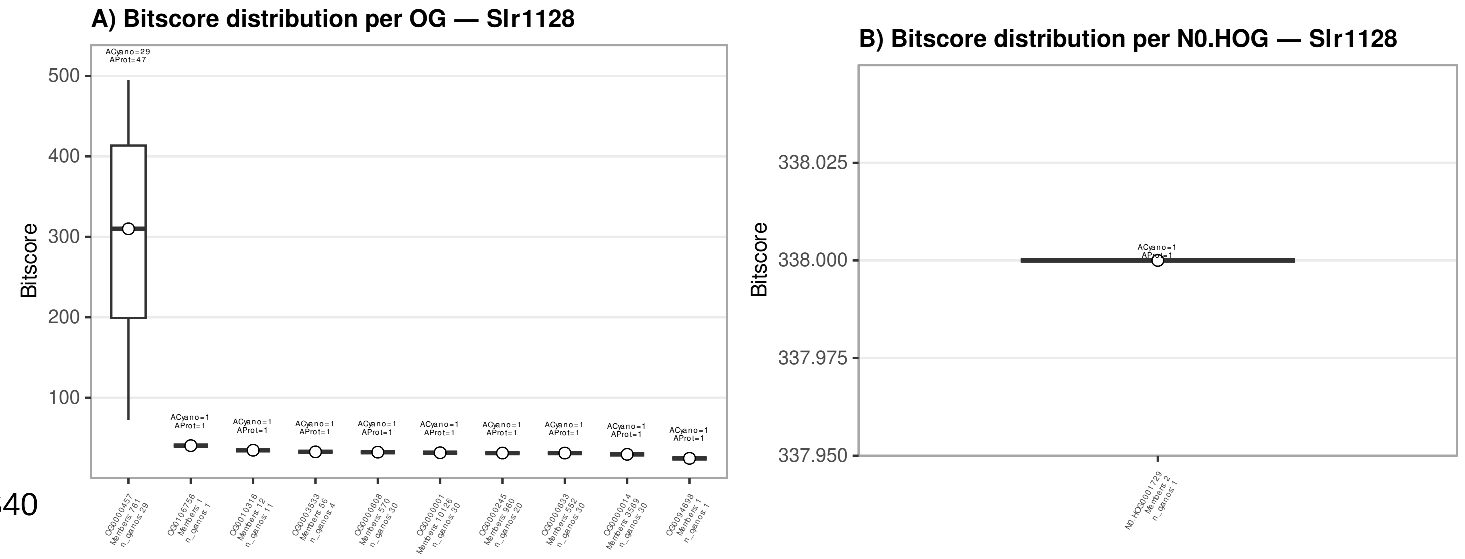

Fig. S40

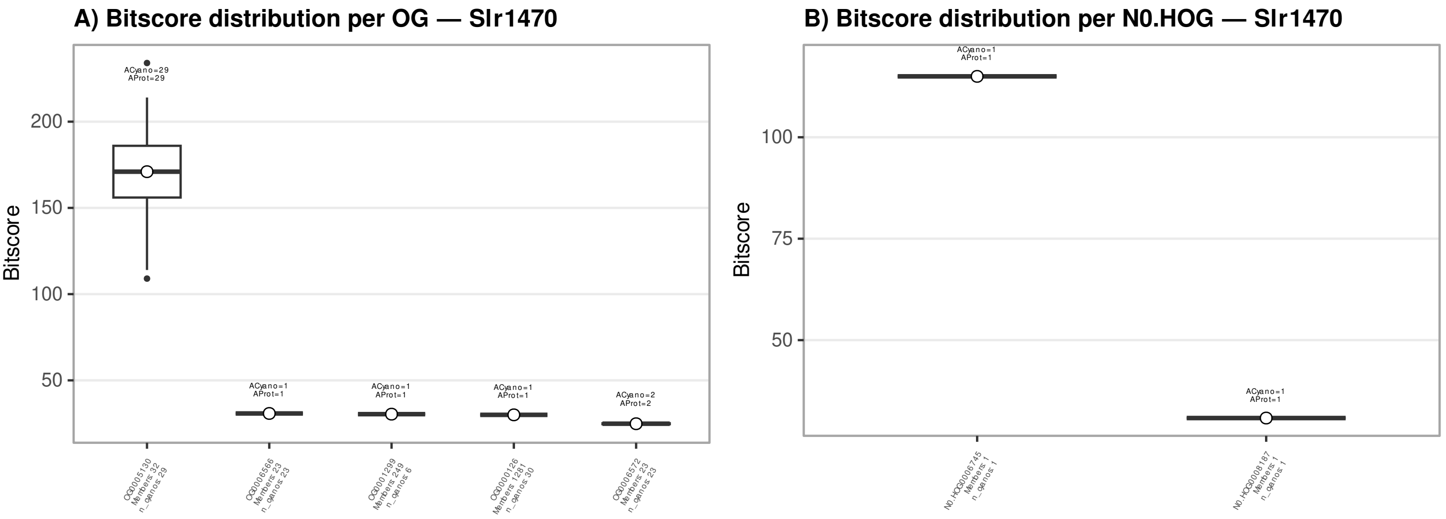

Fig. S41

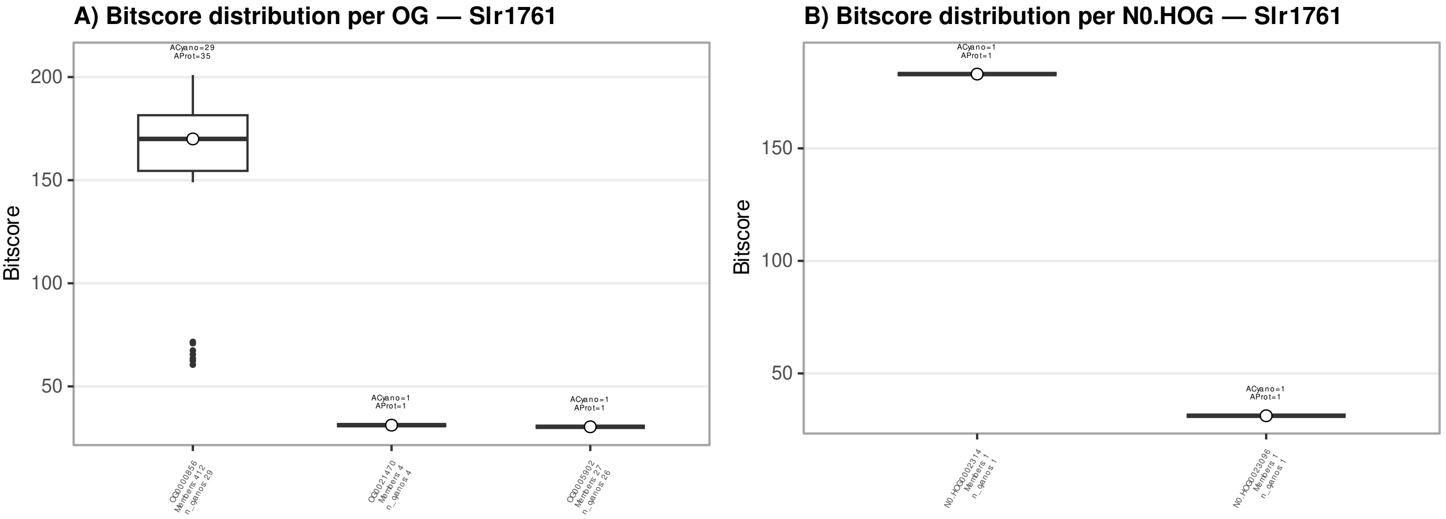

Fig. S42

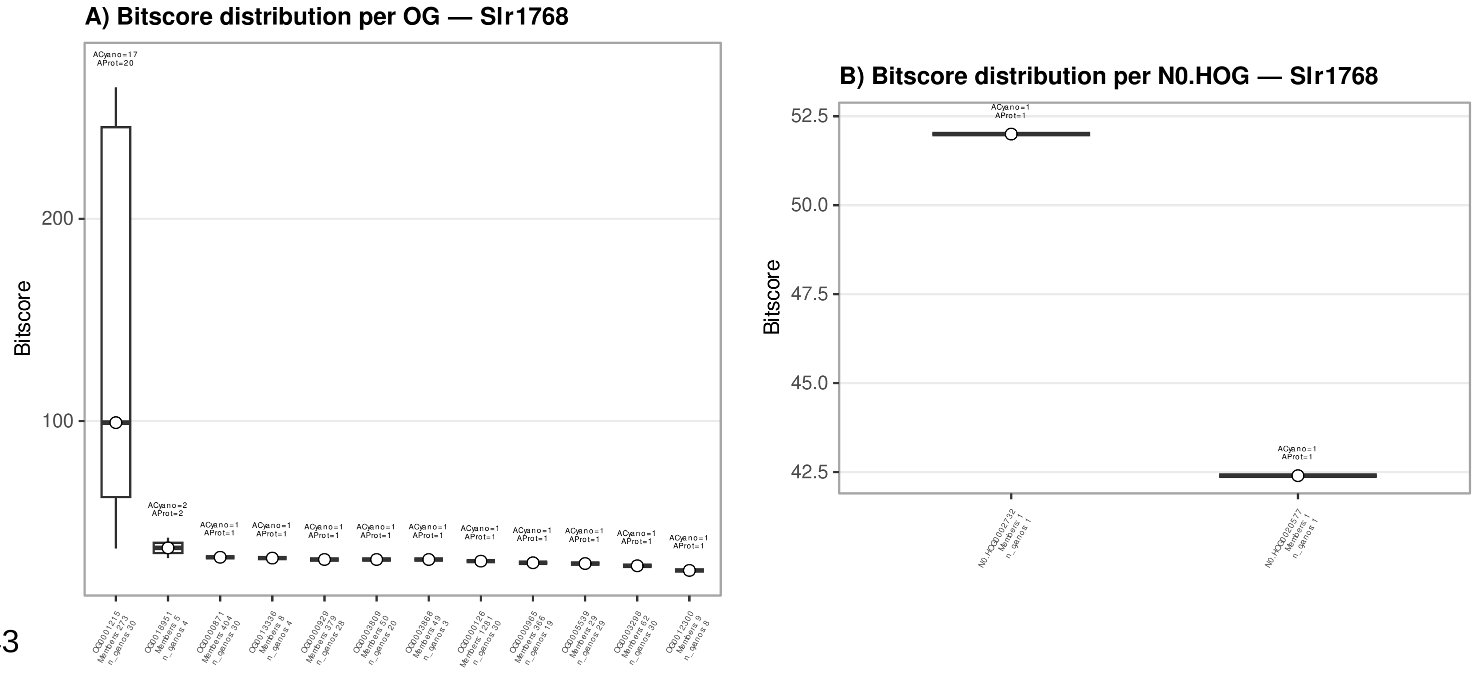

Fig. S43

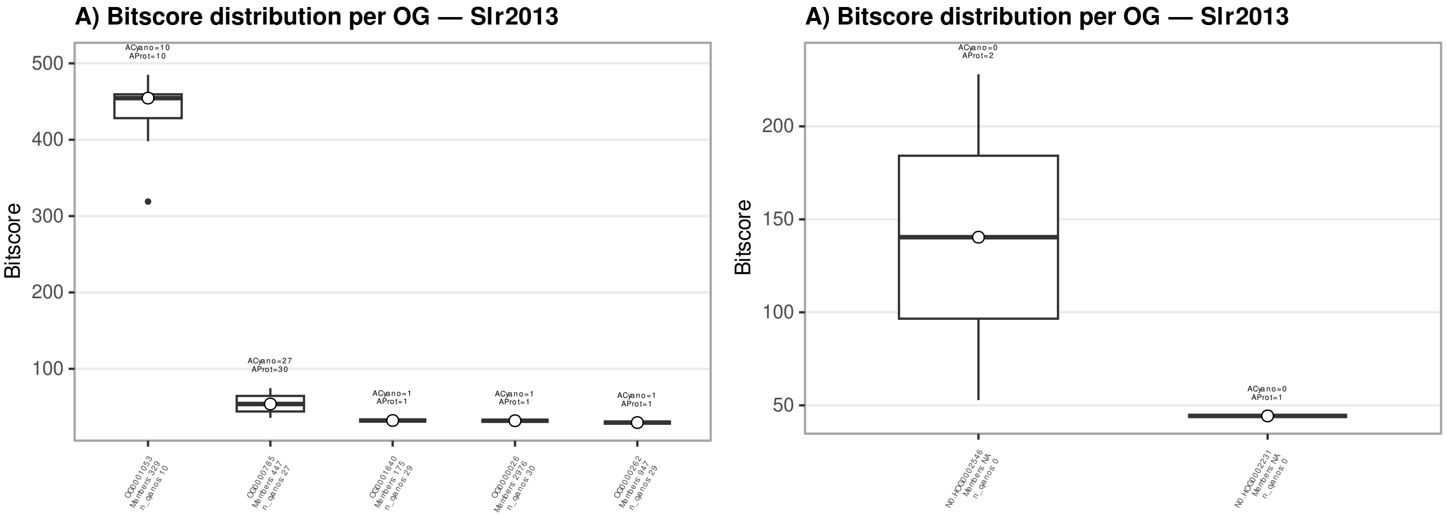

Fig. S44

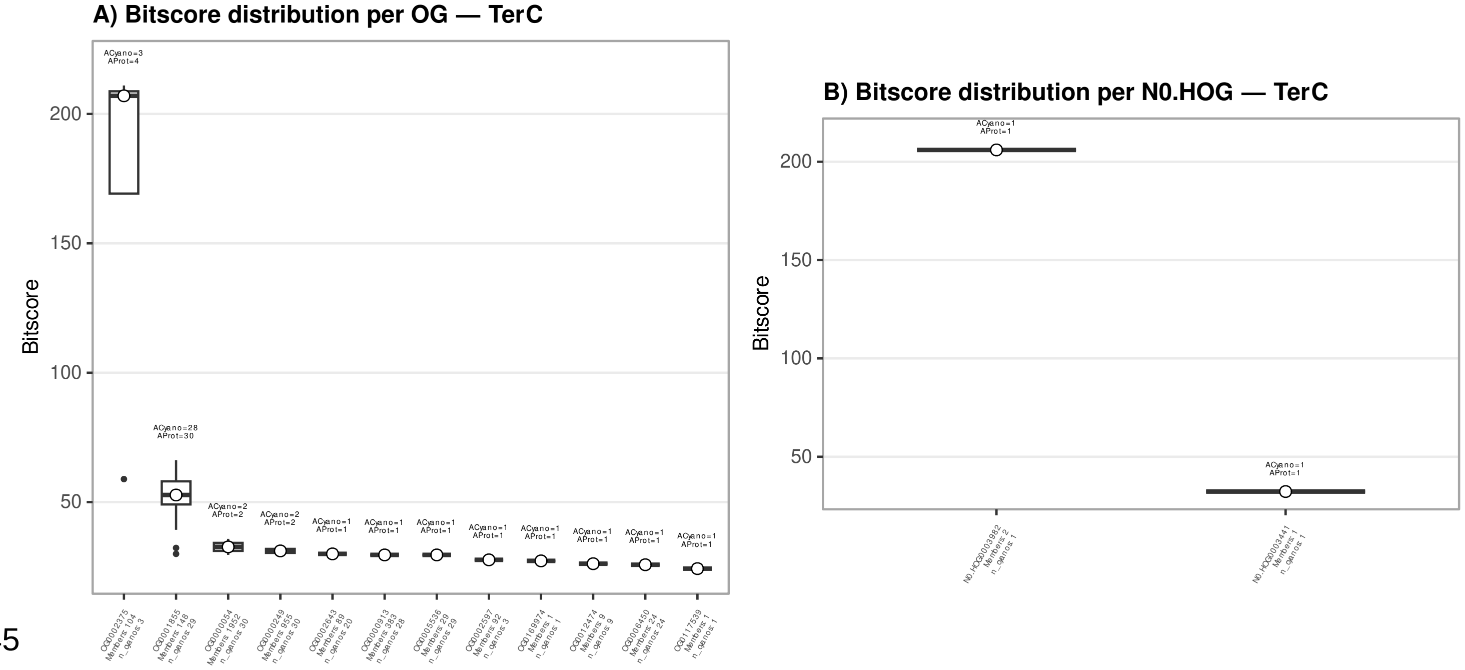

Fig. S45

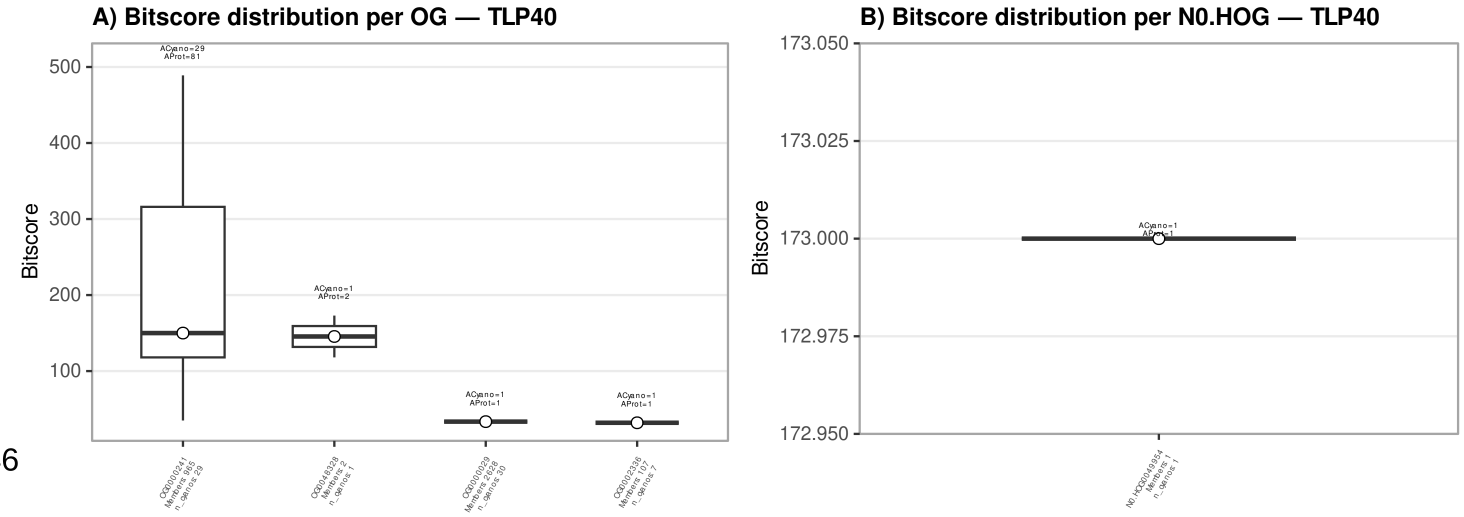

Fig. S46

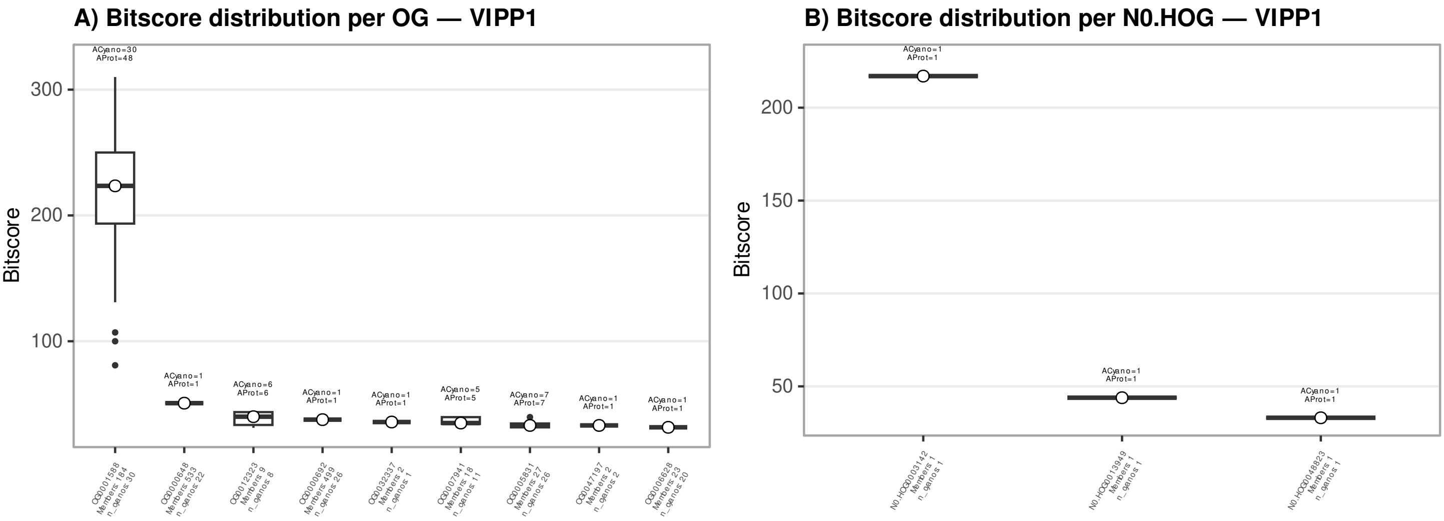

Fig. S47

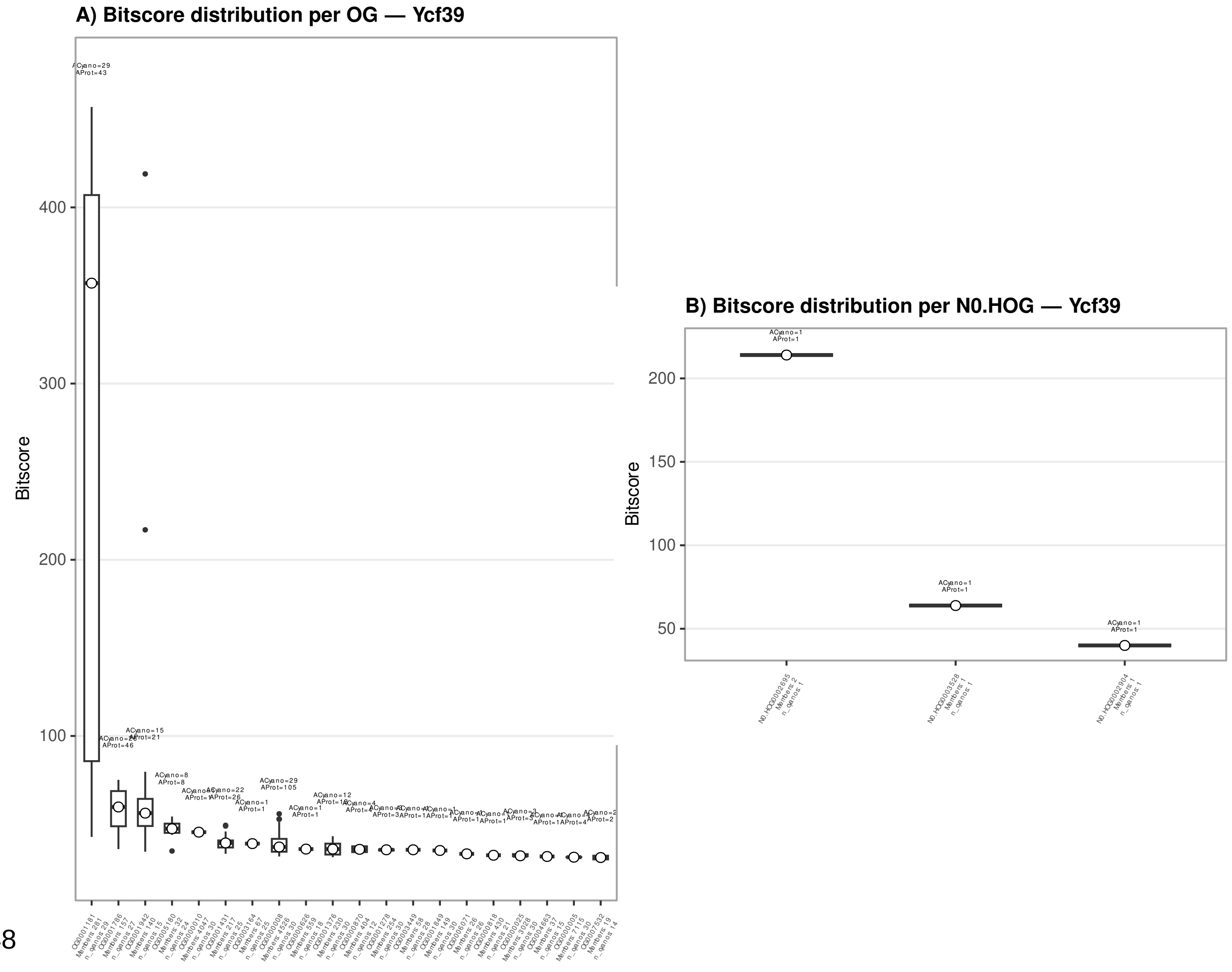

Fig. S48

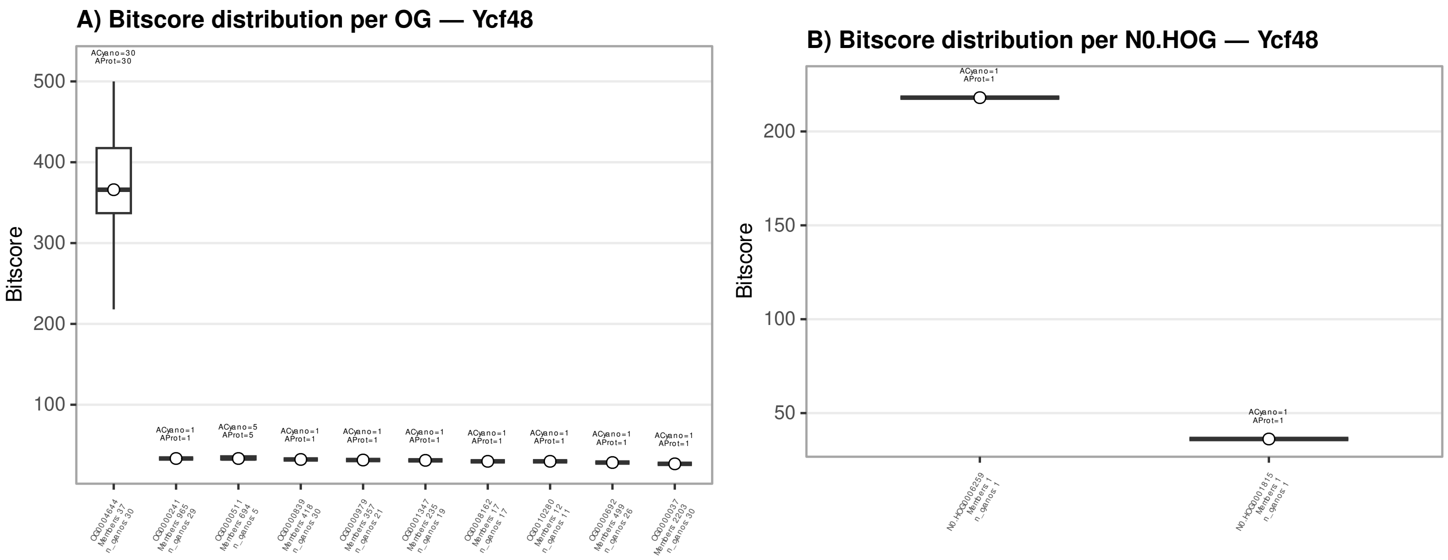

Fig. S49

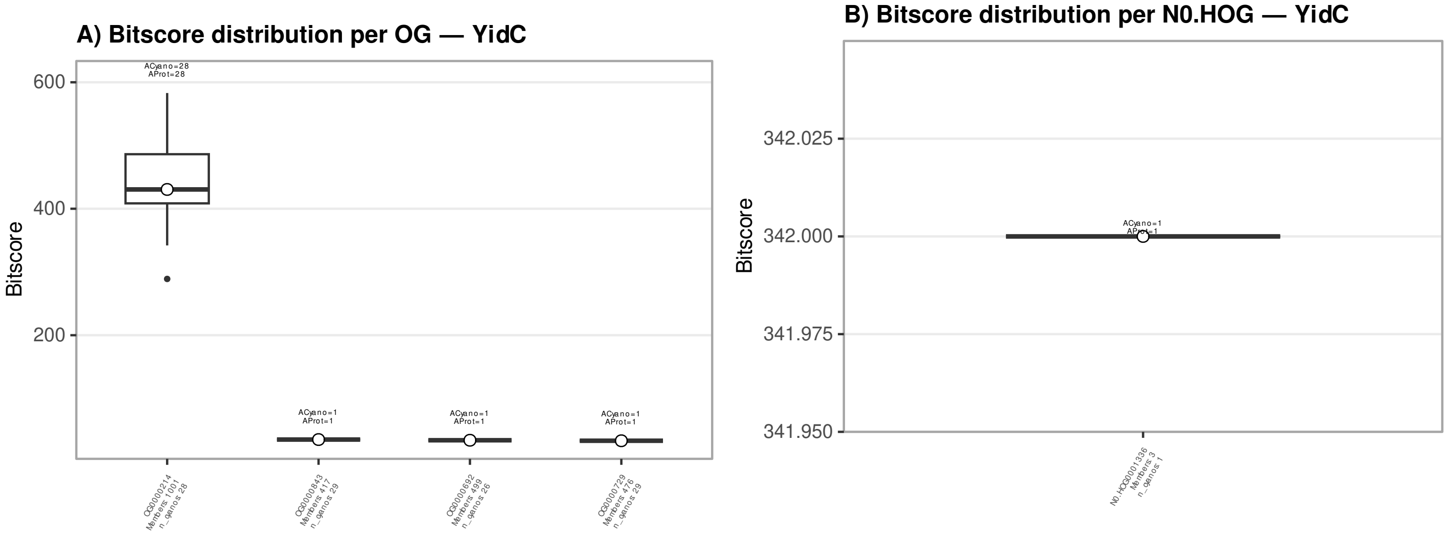

Fig. S50

Fig. S51 - ChlG

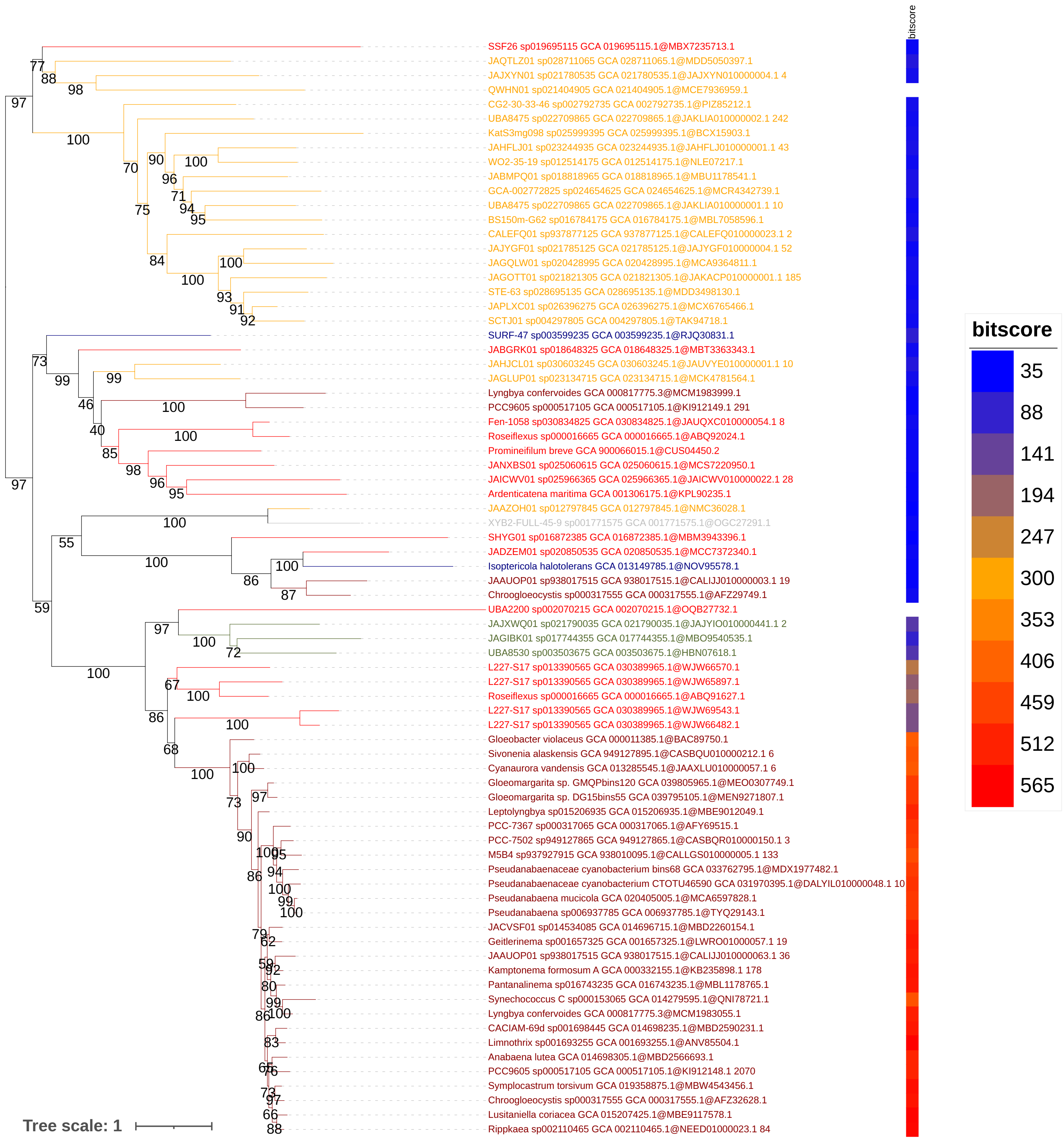
SSF26 sp019695115 GCA 019695115.1@MBX7235713.1
JAQTLZ01 sp028711065 GCA 028711065.1@MDD5050397.1
JAJXYN01 sp021780535 GCA 021780535.1@JAJXYN010000004.1 4
QWHN01 sp021404905 GCA 021404905.1@MCE7936959.1
CG2-30-33-46 sp002792735 GCA 002792735.1@PIZ85212.1
UBA8475 sp022709865 GCA 022709865.1@JAKLIA010000002.1 242
KatS3mg098 sp025999395 GCA 025999395.1@BCX15903.1
JAHFLJ01 sp023244935 GCA 023244935.1@JAHFLJ010000001.1 43
WO2-35-19 sp012514175 GCA 012514175.1@NLE07217.1
JABMPQ01 sp018818965 GCA 018818965.1@MBU1178541.1
GCA-002772825 sp024654625 GCA 024654625.1@MCR4342739.1
UBA8475 sp022709865 GCA 022709865.1@JAKLIA010000001.1 10
BS150m-G62 sp016784175 GCA 016784175.1@MBL7058596.1
CALEFQ01 sp937877125 GCA 937877125.1@CALEFQ010000023.1 2
JAJYGF01 sp021785125 GCA 021785125.1@JAJYGF010000004.1 52
JAGQLW01 sp020428995 GCA 020428995.1@MCA9364811.1
JAGOTT01 sp021821305 GCA 021821305.1@JAKACP010000001.1 185
STE-63 sp028695135 GCA 028695135.1@MDD3498130.1
JAPLXC01 sp026396275 GCA 026396275.1@MCX6765466.1
SCTJ01 sp004297805 GCA 004297805.1@TAK94718.1
SURF-47 sp003599235 GCA 003599235.1@RJQ30831.1
JABGRK01 sp018648325 GCA 018648325.1@MBT3363343.1
JAHJCL01 sp030603245 GCA 030603245.1@JAUVYE010000001.1 10
JAGLUP01 sp023134715 GCA 023134715.1@MCK4781564.1
Lyngbya confervoides GCA 000817775.3@MCM1983999.1
PCC9605 sp000517105 GCA 000517105.1@KI912149.1 291
Fen-1058 sp030834825 GCA 030834825.1@JAUQXC010000054.1 8
Roseiflexus sp000016665 GCA 000016665.1@ABQ92024.1
Promineifilum breve GCA 900066015.1@CUS04450.2
JANXBS01 sp025060615 GCA 025060615.1@MCS7220950.1
JAICWV01 sp025966365 GCA 025966365.1@JAICWV010000022.1 28
Ardenticatena maritima GCA 001306175.1@KPL90235.1
JAAZOH01 sp012797845 GCA 012797845.1@NMC36028.1
XYB2-FULL-45-9 sp001771575 GCA 001771575.1@OGC27291.1
SHYG01 sp016872385 GCA 016872385.1@MBM3943396.1
JADZEM01 sp020850535 GCA 020850535.1@MCC7372340.1
Isopericola halotolerans GCA 013149785.1@NOV95578.1
JAAUOP01 sp938017515 GCA 938017515.1@CALIJJ010000003.1 19
Chroogloeocystis sp000317555 GCA 000317555.1@AFZ29749.1
UBA2200 sp002070215 GCA 002070215.1@OQB27732.1
JAJXWQ01 sp021790035 GCA 021790035.1@JAJYIO010000441.1 2
JAGIBK01 sp017744355 GCA 017744355.1@MBO9540535.1
UBA8530 sp003503675 GCA 003503675.1@HBN07618.1
L227-S17 sp013390565 GCA 030389965.1@WJW66570.1
L227-S17 sp013390565 GCA 030389965.1@WJW65897.1
Roseiflexus sp000016665 GCA 000016665.1@ABQ91627.1
L227-S17 sp013390565 GCA 030389965.1@WJW69543.1
L227-S17 sp013390565 GCA 030389965.1@WJW66482.1
Gloeobacter violaceus GCA 000011385.1@BAC89750.1
Sivonenia alaskensis GCA 949127895.1@CASBQU010000212.1 6
Cyanaurora vandensis GCA 013285545.1@JAAXLU010000057.1 6
Gloeomargarita sp. GMQPbins120 GCA 039805965.1@MEO0307749.1
Gloeomargarita sp. DG15bins55 GCA 039795105.1@MEN9271807.1
Leptolyngbya sp015206935 GCA 015206935.1@MBE9012049.1
PCC-7367 sp000317065 GCA 000317065.1@AFY69515.1
PCC-7502 sp949127865 GCA 949127865.1@CASBQR010000150.1 3
M5B4 sp937927915 GCA 938010095.1@CALLGS010000005.1 133
Pseudanabaenaceae cyanobacterium bins68 GCA 033762795.1@MDX1977482.1
Pseudanabaenaceae cyanobacterium CTOTU46590 GCA 031970395.1@DALYIL010000048.1 10
Pseudanabaena mucicola GCA 020405005.1@MCA6597828.1
Pseudanabaena sp006937785 GCA 006937785.1@TYQ29143.1
JACVSF01 sp014534085 GCA 014696715.1@MBD2260154.1
Geitlerinema sp001657325 GCA 001657325.1@LWRO01000057.1 19
JAAUOP01 sp938017515 GCA 938017515.1@CALIJJ010000063.1 36
Kamptonema formosum A GCA 000332155.1@KB235898.1 178
Pantanalinema sp016743235 GCA 016743235.1@MBL1178765.1
Synechococcus C sp000153065 GCA 014279595.1@QNI78721.1
Lyngbya confervoides GCA 000817775.3@MCM1983055.1
CACIAM-69d sp001698445 GCA 014698235.1@MBD2590231.1
Limnothrix sp001693255 GCA 001693255.1@ANV85504.1
Anabaena lutea GCA 014698305.1@MBD2566693.1
PCC9605 sp000517105 GCA 000517105.1@KI912148.1 2070
Symplocastrum torsivum GCA 019358875.1@MBW4543456.1
Chroogloeocystis sp000317555 GCA 000317555.1@AFZ32628.1
Lusitaniella coriacea GCA 015207425.1@MBE9117578.1
Rippkaea sp002110465 GCA 002110465.1@NEED01000023.1 84
bitscore
bitscore
35
88
141
194
247
300
353
406
459
512
565
Tree scale: 1

Fig. S52 - CtpA

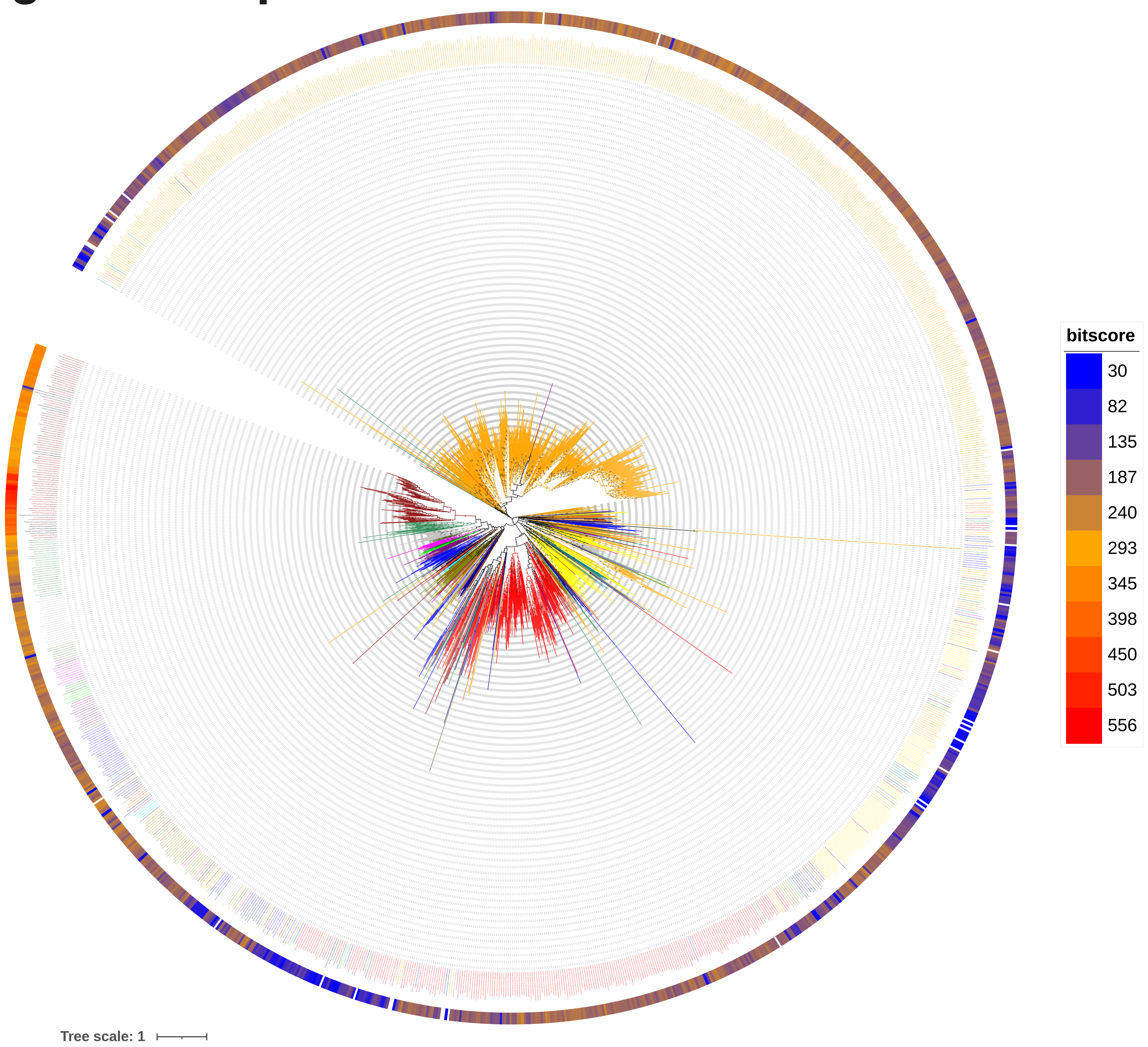
bitscore
30
82
135
187
240
293
345
398
450
503
556
Tree scale: 1

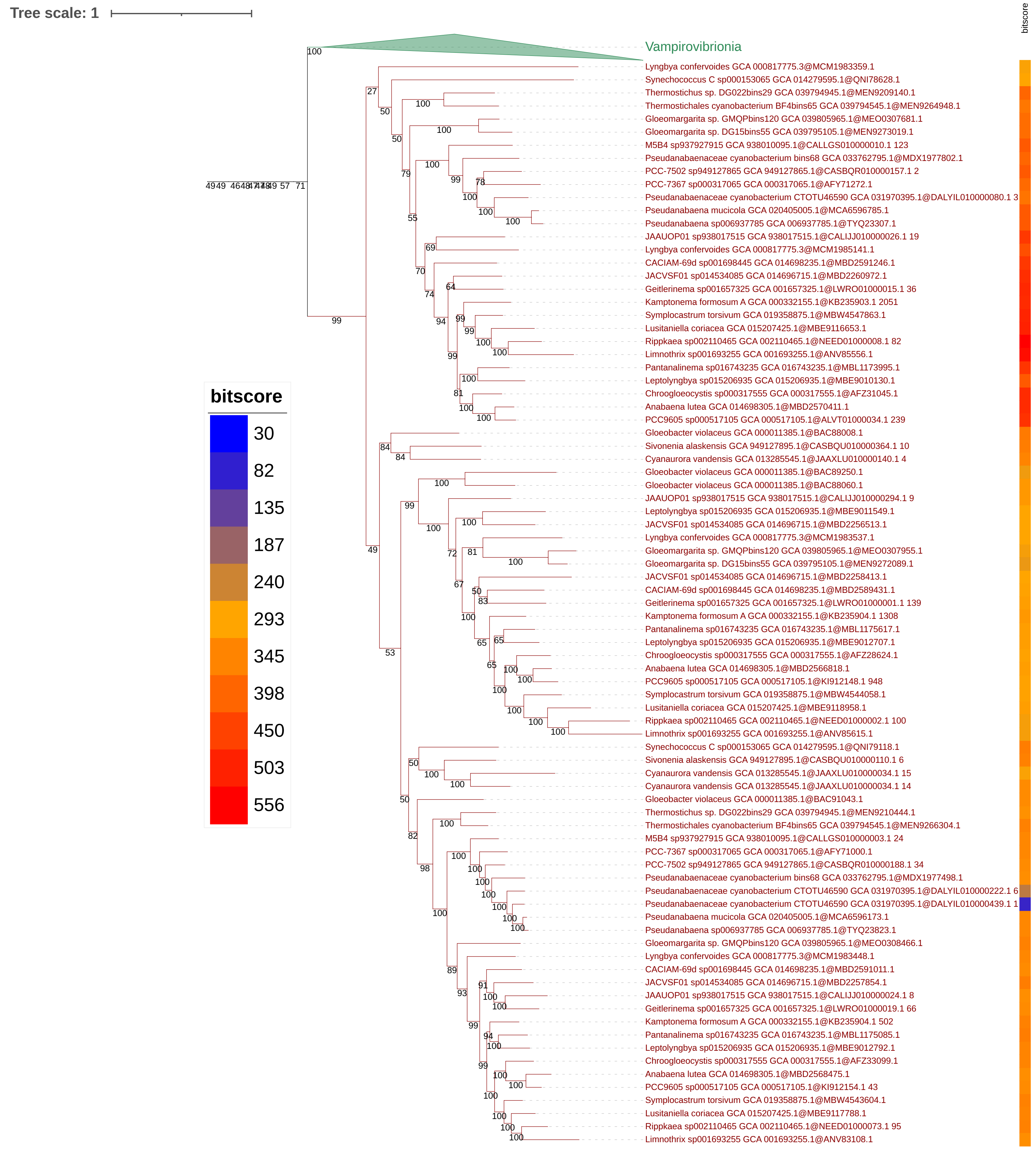
Tree scale: 1
bitscore
bitscore
30
82
135
187
240
293
345
398
450
503
556
Vampirovibrionia
Lyngbya confervoides GCA 000817775.3@MCM1983359.1
Synechococcus C sp000153065 GCA 014279595.1@QNI78628.1
Thermostichus sp. DG022bins29 GCA 039794945.1@MEN9209140.1
Thermostichales cyanobacterium BF4bins65 GCA 039794545.1@MEN9264948.1
Gloeomargarita sp. GMQPbins120 GCA 039805965.1@MEO0307681.1
Gloeomargarita sp. DG15bins55 GCA 039795105.1@MEN9273019.1
M5B4 sp937927915 GCA 938010095.1@CALLGS010000010.1 123
Pseudanabaenaceae cyanobacterium bins68 GCA 033762795.1@MDX1977802.1
PCC-7502 sp949127865 GCA 949127865.1@CASBQR010000157.1 2
PCC-7367 sp000317065 GCA 000317065.1@AFY71272.1
Pseudanabaenaceae cyanobacterium CTOTU46590 GCA 031970395.1@DALYIL010000080.1 3
Pseudanabaena mucicola GCA 020405005.1@MCA6596785.1
Pseudanabaena sp006937785 GCA 006937785.1@TYQ23307.1
JAAUOP01 sp938017515 GCA 938017515.1@CALIJJ010000026.1 19
Lyngbya confervoides GCA 000817775.3@MCM1985141.1
CACIAM-69d sp001698445 GCA 014698235.1@MBD2591246.1
JACVSF01 sp014534085 GCA 014696715.1@MBD2260972.1
Geitlerinema sp001657325 GCA 001657325.1@LWRO01000015.1 36
Kamptonema formosum A GCA 000332155.1@KB235903.1 2051
Symplocastrum torsivum GCA 019358875.1@MBW4547863.1
Lusitaniella coriacea GCA 015207425.1@MBE9116653.1
Rippkaea sp002110465 GCA 002110465.1@NEED01000008.1 82
Limnothrix sp001693255 GCA 001693255.1@ANV85556.1
Pantanalinema sp016743235 GCA 016743235.1@MBL1173995.1
Leptolyngbya sp015206935 GCA 015206935.1@MBE9010130.1
Chroogloeocystis sp000317555 GCA 000317555.1@AFZ31045.1
Anabaena lutea GCA 014698305.1@MBD2570411.1
PCC9605 sp000517105 GCA 000517105.1@ALVT01000034.1 239
Gloeobacter violaceus GCA 000011385.1@BAC88008.1
Sivonenia alaskensis GCA 949127895.1@CASBQU010000364.1 10
Cyanaurora vandensis GCA 013285545.1@JAAXLU010000140.1 4
Gloeobacter violaceus GCA 000011385.1@BAC89250.1
Gloeobacter violaceus GCA 000011385.1@BAC88060.1
JAAUOP01 sp938017515 GCA 938017515.1@CALIJJ010000294.1 9
Leptolyngbya sp015206935 GCA 015206935.1@MBE9011549.1
JACVSF01 sp014534085 GCA 014696715.1@MBD2256513.1
Lyngbya confervoides GCA 000817775.3@MCM1983537.1
Gloeomargarita sp. GMQPbins120 GCA 039805965.1@MEO0307955.1
Gloeomargarita sp. DG15bins55 GCA 039795105.1@MEN9272089.1
JACVSF01 sp014534085 GCA 014696715.1@MBD2258413.1
CACIAM-69d sp001698445 GCA 014698235.1@MBD2589431.1
Geitlerinema sp001657325 GCA 001657325.1@LWRO01000001.1 139
Kamptonema formosum A GCA 000332155.1@KB235904.1 1308
Pantanalinema sp016743235 GCA 016743235.1@MBL1175617.1
Leptolyngbya sp015206935 GCA 015206935.1@MBE9012707.1
Chroogloeocystis sp000317555 GCA 000317555.1@AFZ28624.1
Anabaena lutea GCA 014698305.1@MBD2566818.1
PCC9605 sp000517105 GCA 000517105.1@KI912148.1 948
Symplocastrum torsivum GCA 019358875.1@MBW4544058.1
Lusitaniella coriacea GCA 015207425.1@MBE9118958.1
Rippkaea sp002110465 GCA 002110465.1@NEED01000002.1 100
Limnothrix sp001693255 GCA 001693255.1@ANV85615.1
Synechococcus C sp000153065 GCA 014279595.1@QNI79118.1
Sivonenia alaskensis GCA 949127895.1@CASBQU010000110.1 6
Cyanaurora vandensis GCA 013285545.1@JAAXLU010000034.1 15
Cyanaurora vandensis GCA 013285545.1@JAAXLU010000034.1 14
Gloeobacter violaceus GCA 000011385.1@BAC91043.1
Thermostichus sp. DG022bins29 GCA 039794945.1@MEN9210444.1
Thermostichales cyanobacterium BF4bins65 GCA 039794545.1@MEN9266304.1
M5B4 sp937927915 GCA 938010095.1@CALLGS010000003.1 24
PCC-7367 sp000317065 GCA 000317065.1@AFY71000.1
PCC-7502 sp949127865 GCA 949127865.1@CASBQR010000188.1 34
Pseudanabaenaceae cyanobacterium bins68 GCA 033762795.1@MDX1977498.1
Pseudanabaenaceae cyanobacterium CTOTU46590 GCA 031970395.1@DALYIL010000222.1 6
Pseudanabaenaceae cyanobacterium CTOTU46590 GCA 031970395.1@DALYIL010000439.1 1
Pseudanabaena mucicola GCA 020405005.1@MCA6596173.1
Pseudanabaena sp006937785 GCA 006937785.1@TYQ23823.1
Gloeomargarita sp. GMQPbins120 GCA 039805965.1@MEO0308466.1
Lyngbya confervoides GCA 000817775.3@MCM1983448.1
CACIAM-69d sp001698445 GCA 014698235.1@MBD2591011.1
JACVSF01 sp014534085 GCA 014696715.1@MBD2257854.1
JAAUOP01 sp938017515 GCA 938017515.1@CALIJJ010000024.1 8
Geitlerinema sp001657325 GCA 001657325.1@LWRO01000019.1 66
Kamptonema formosum A GCA 000332155.1@KB235904.1 502
Pantanalinema sp016743235 GCA 016743235.1@MBL1175085.1
Leptolyngbya sp015206935 GCA 015206935.1@MBE9012792.1
Chroogloeocystis sp000317555 GCA 000317555.1@AFZ33099.1
Anabaena lutea GCA 014698305.1@MBD2568475.1
PCC9605 sp000517105 GCA 000517105.1@KI912154.1 43
Symplocastrum torsivum GCA 019358875.1@MBW4543604.1
Lusitaniella coriacea GCA 015207425.1@MBE9117788.1
Rippkaea sp002110465 GCA 002110465.1@NEED01000073.1 95
Limnothrix sp001693255 GCA 001693255.1@ANV83108.1

Fig. S53 - CyanoP (PsbP-like)

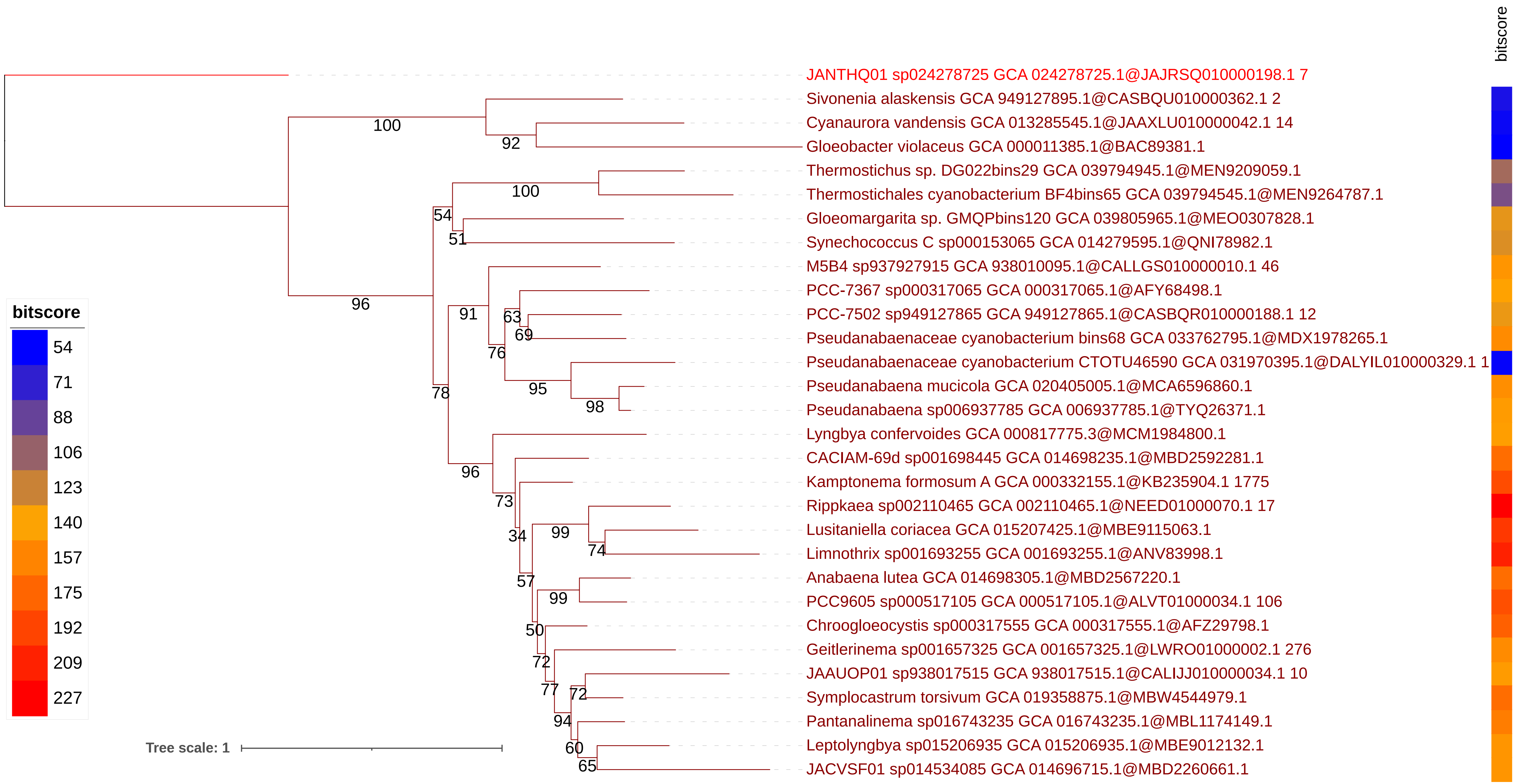

Fig. S54 - FtsZ

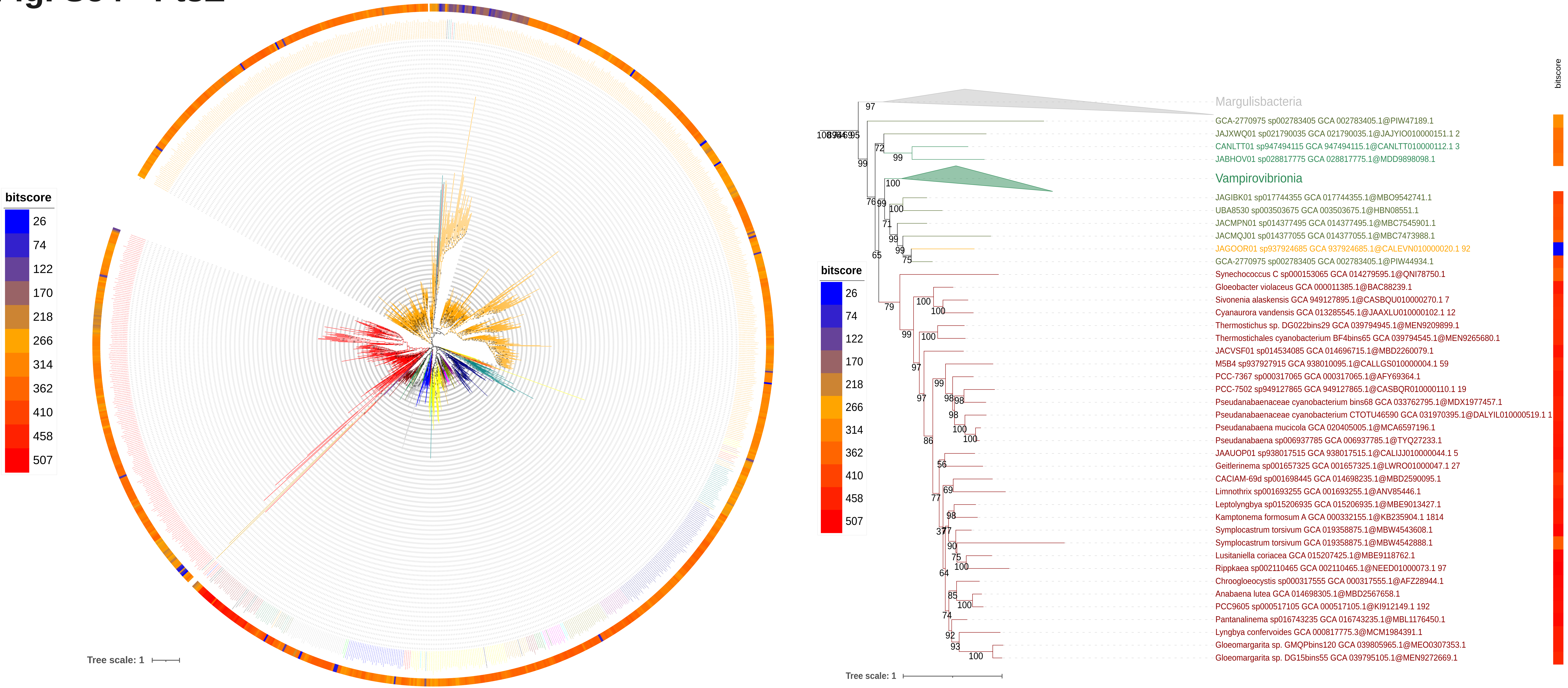
bitscore
26
74
122
170
218
266
314
362
410
458
507
Tree scale: 1
Margulisbacteria
GCA-2770975 sp002783405 GCA 002783405.1@PIW47189.1
JAJXWQ01 sp021790035 GCA 021790035.1@JAJYIO010000151.1 2
CANLTT01 sp947494115 GCA 947494115.1@CANLTT010000112.1 3
JABHOV01 sp028817775 GCA 028817775.1@MDD9898098.1
Vampirovibrionia
JAGIBK01 sp017744355 GCA 017744355.1@MBO9542741.1
UBA8530 sp003503675 GCA 003503675.1@HBN08551.1
JACMPN01 sp014377495 GCA 014377495.1@MBC7545901.1
JACMQJ01 sp014377055 GCA 014377055.1@MBC7473988.1
JAGOOR01 sp937924685 GCA 937924685.1@CALEVN010000020.1 92
GCA-2770975 sp002783405 GCA 002783405.1@PIW44934.1
Synechococcus C sp000153065 GCA 014279595.1@QNI78750.1
Gloeobacter violaceus GCA 000011385.1@BAC88239.1
Sivonenia alaskensis GCA 949127895.1@CASBQU010000270.1 7
Cyanaurora vandensis GCA 013285545.1@JAAXLU010000102.1 12
Thermostichus sp. DG022bins29 GCA 039794945.1@MEN9209899.1
Thermostichales cyanobacterium BF4bins65 GCA 039794545.1@MEN9265680.1
JACVSF01 sp014534085 GCA 014696715.1@MBD2260079.1
M5B4 sp937927915 GCA 938010095.1@CALLGS010000004.1 59
PCC-7367 sp000317065 GCA 000317065.1@AFY69364.1
PCC-7502 sp949127865 GCA 949127865.1@CASBQR010000110.1 19
Pseudanabaenaceae cyanobacterium bins68 GCA 033762795.1@MDX1977457.1
Pseudanabaenaceae cyanobacterium CTOTU46590 GCA 031970395.1@DALYIL010000519.1 1
Pseudanabaena mucicola GCA 020405005.1@MCA6597196.1
Pseudanabaena sp006937785 GCA 006937785.1@TYQ27233.1
JAAUOP01 sp938017515 GCA 938017515.1@CALIJJ010000044.1 5
Geitlerinema sp001657325 GCA 001657325.1@LWRO01000047.1 27
CACIAM-69d sp001698445 GCA 014698235.1@MBD2590095.1
Limnothrix sp001693255 GCA 001693255.1@ANV85446.1
Leptolyngbya sp015206935 GCA 015206935.1@MBE9013427.1
Kamptonema formosum A GCA 000332155.1@KB235904.1 1814
Symplocastrum torsivum GCA 019358875.1@MBW4543608.1
Symplocastrum torsivum GCA 019358875.1@MBW4542888.1
Lusitaniella coriacea GCA 015207425.1@MBE9118762.1
Rippkaea sp002110465 GCA 002110465.1@NEED01000073.1 97
Chroogloeocystis sp000317555 GCA 000317555.1@AFZ28944.1
Anabaena lutea GCA 014698305.1@MBD2567658.1
PCC9605 sp000517105 GCA 000517105.1@KI912149.1 192
Pantanalinema sp016743235 GCA 016743235.1@MBL1176450.1
Lyngbya confervoides GCA 000817775.3@MCM1984391.1
Gloeomargarita sp. GMQPbins120 GCA 039805965.1@MEO0307353.1
Gloeomargarita sp. DG15bins55 GCA 039795105.1@MEN9272669.1

Fig. S55 - HliABCD

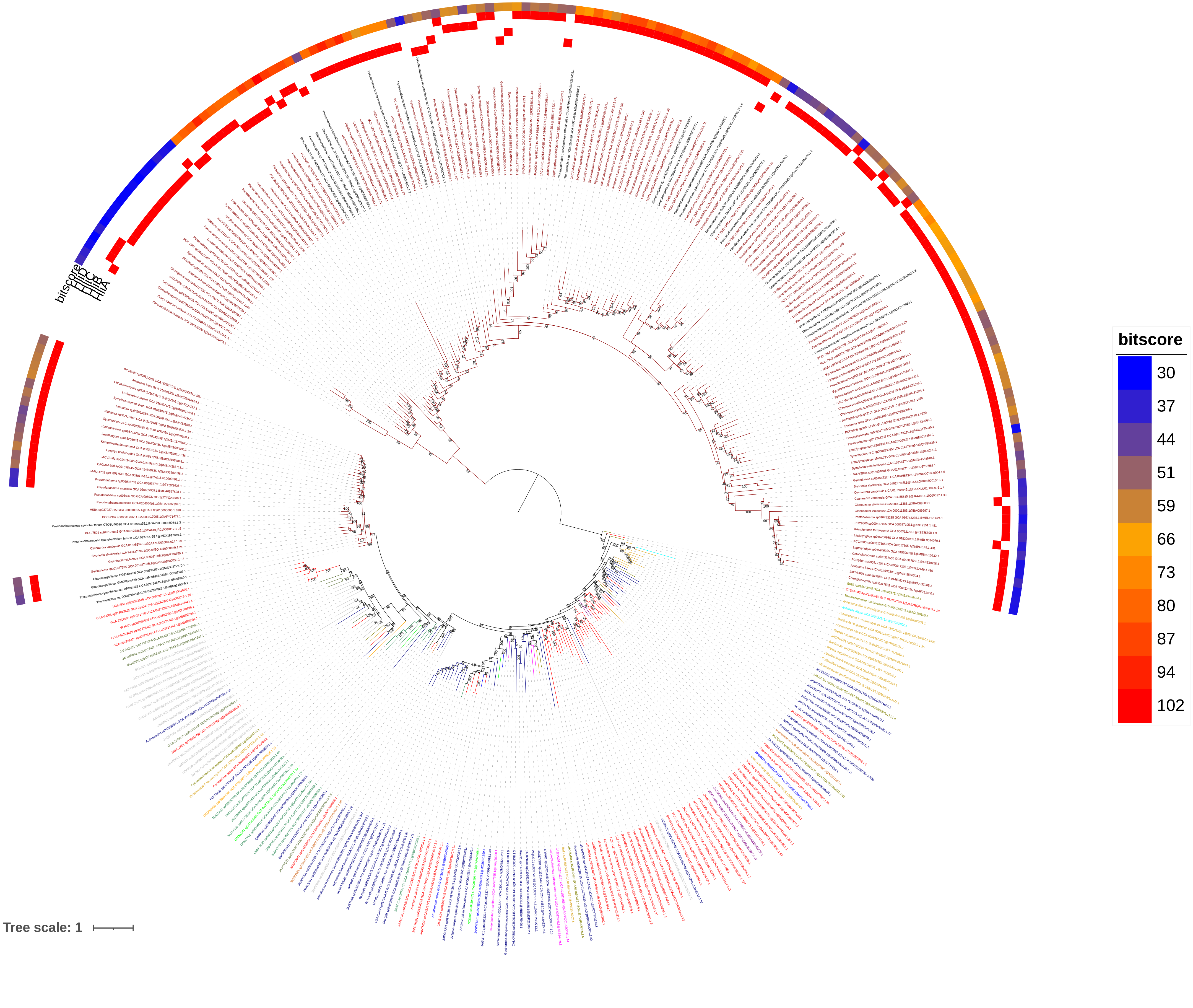

Fig. S56 - PAM71

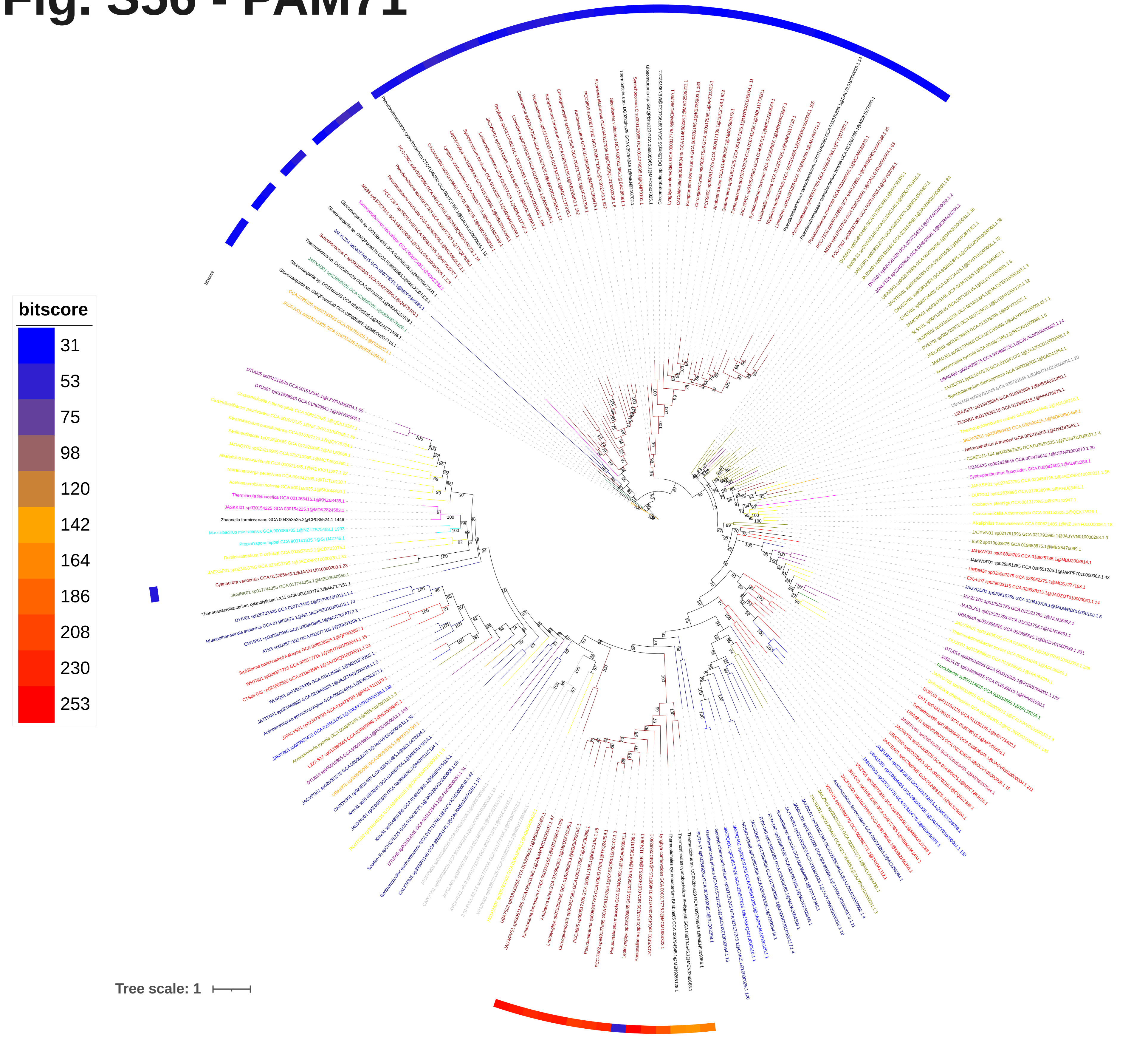

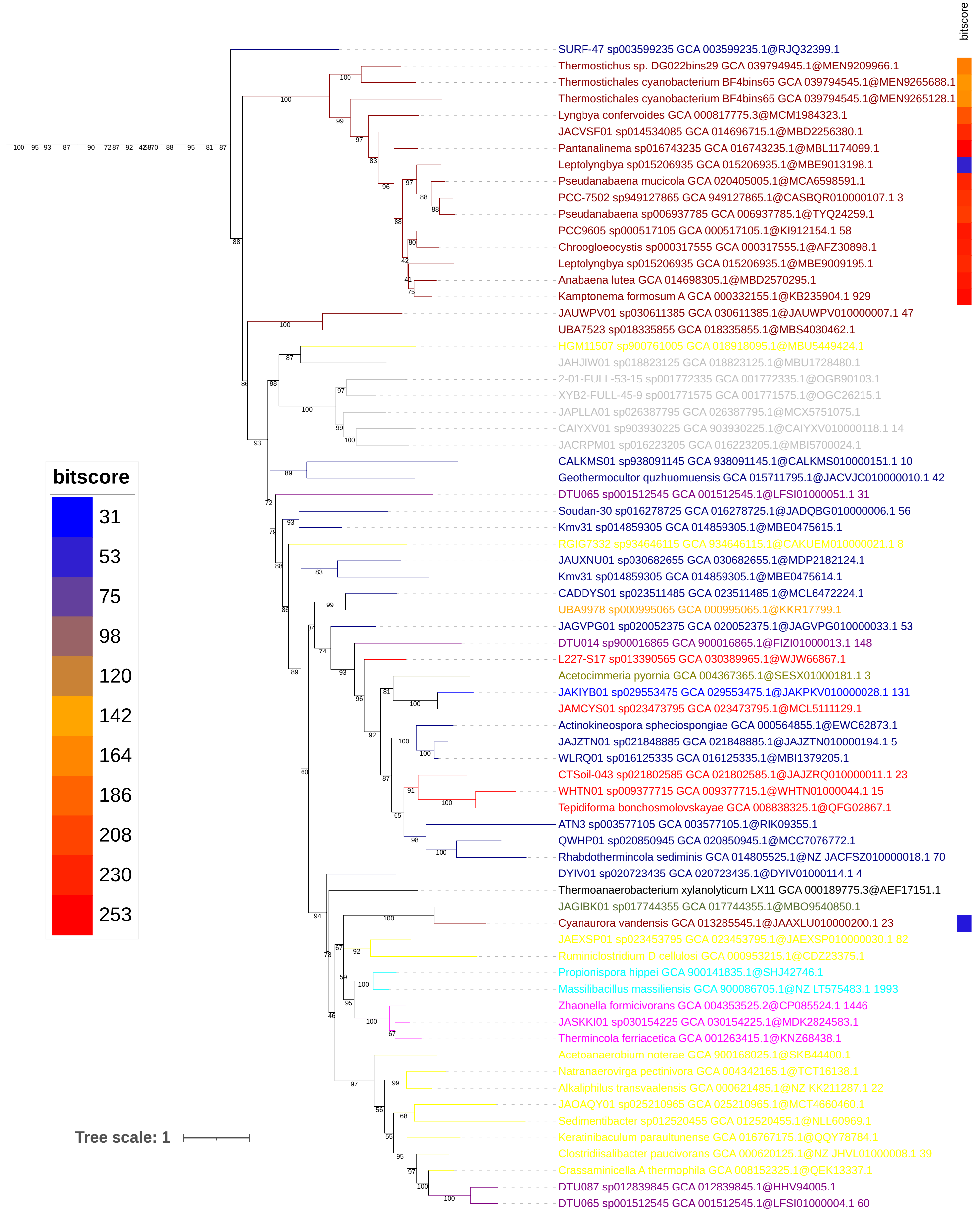

Fig. S57 - Pitt

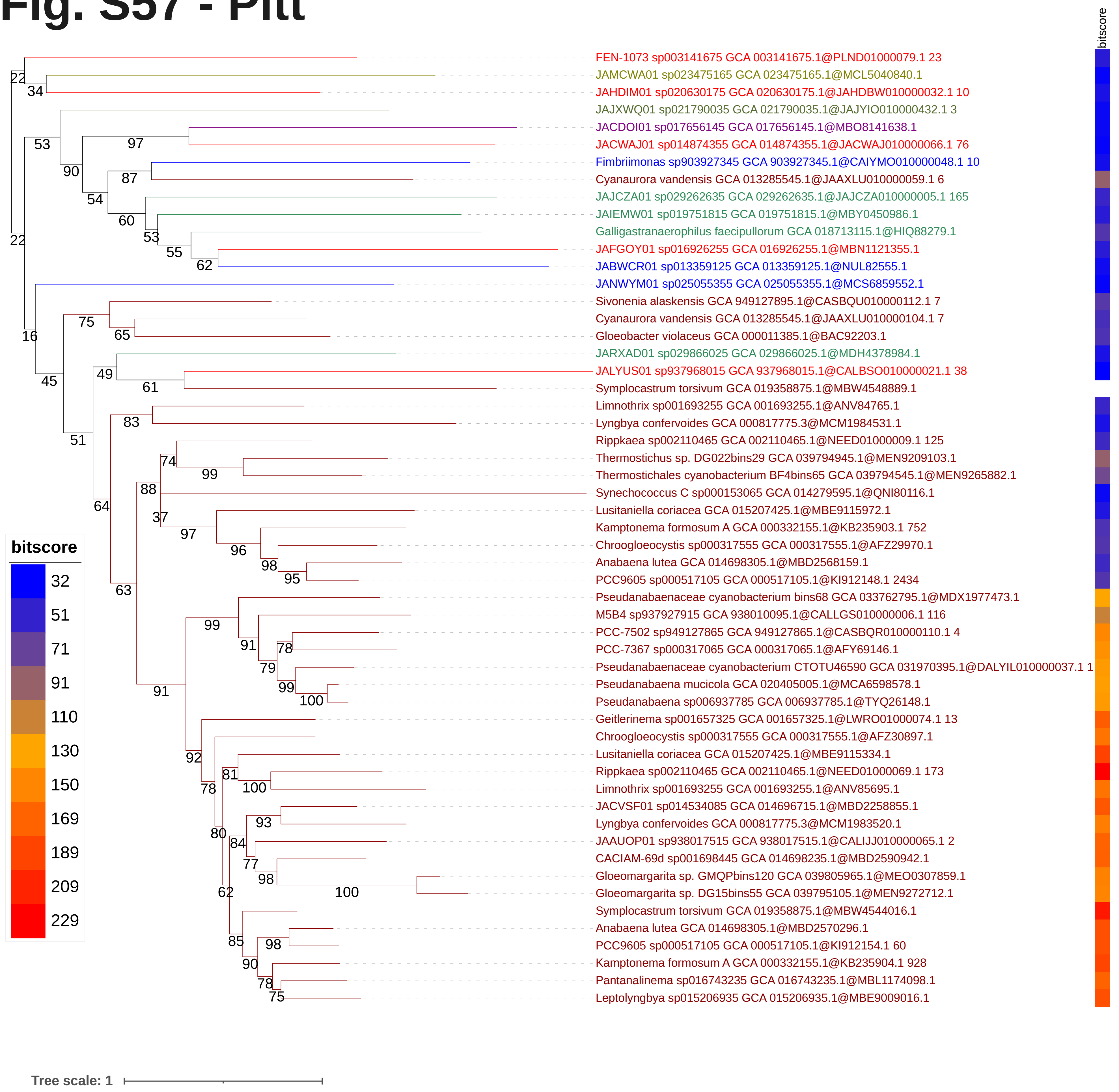

bitscore
FEN-1073 sp003141675 GCA 003141675.1@PLND01000079.1 23
JAMCWA01 sp023475165 GCA 023475165.1@MCL5040840.1
JAHDIM01 sp020630175 GCA 020630175.1@JAHDBW010000032.1 10
JAJXWQ01 sp021790035 GCA 021790035.1@JAJYIO010000432.1 3
JACDOI01 sp017656145 GCA 017656145.1@MBO8141638.1
JACWAJ01 sp014874355 GCA 014874355.1@JACWAJ010000066.1 76
Fimbriimonas sp903927345 GCA 903927345.1@CAIYMO010000048.1 10
Cyanaurora vandensis GCA 013285545.1@JAAXLU010000059.1 6
JAJCZA01 sp029262635 GCA 029262635.1@JAJCZA010000005.1 165
JAIEMW01 sp019751815 GCA 019751815.1@MBY0450986.1
Galligastranaerophilus faecipullorum GCA 018713115.1@HIQ88279.1
JAFGOY01 sp016926255 GCA 016926255.1@MBN1121355.1
JABWCR01 sp013359125 GCA 013359125.1@NUL82555.1
JANWYM01 sp025055355 GCA 025055355.1@MCS6859552.1
Sivonenia alaskensis GCA 949127895.1@CASBQU010000112.1 7
Cyanaurora vandensis GCA 013285545.1@JAAXLU010000104.1 7
Gloeobacter violaceus GCA 000011385.1@BAC92203.1
JARXAD01 sp029866025 GCA 029866025.1@MDH4378984.1
JALYUS01 sp937968015 GCA 937968015.1@CALBSO010000021.1 38
Symplocastrum torsivum GCA 019358875.1@MBW4548889.1
Limnothrix sp001693255 GCA 001693255.1@ANV84765.1
Lyngbya confervoides GCA 000817775.3@MCM1984531.1
Rippkaea sp002110465 GCA 002110465.1@NEED01000009.1 125
Thermostichus sp. DG022bins29 GCA 039794945.1@MEN9209103.1
Thermostichales cyanobacterium BF4bins65 GCA 039794545.1@MEN9265882.1
Synechococcus C sp000153065 GCA 014279595.1@QNI80116.1
Lusitaniella coriacea GCA 015207425.1@MBE9115972.1
Kamptonema formosum A GCA 000332155.1@KB235903.1 752
Chroogloeocystis sp000317555 GCA 000317555.1@AFZ29970.1
Anabaena lutea GCA 014698305.1@MBD2568159.1
PCC9605 sp000517105 GCA 000517105.1@KI912148.1 2434
Pseudanabaenaceae cyanobacterium bins68 GCA 033762795.1@MDX1977473.1
M5B4 sp937927915 GCA 938010095.1@CALLGS010000006.1 116
PCC-7502 sp949127865 GCA 949127865.1@CASBQR010000110.1 4
PCC-7367 sp000317065 GCA 000317065.1@AFY69146.1
Pseudanabaenaceae cyanobacterium CTOTU46590 GCA 031970395.1@DALYIL010000037.1 1
Pseudanabaena mucicola GCA 020405005.1@MCA6598578.1
Pseudanabaena sp006937785 GCA 006937785.1@TYQ26148.1
Geitlerinema sp001657325 GCA 001657325.1@LWRO01000074.1 13
Chroogloeocystis sp000317555 GCA 000317555.1@AFZ30897.1
Lusitaniella coriacea GCA 015207425.1@MBE9115334.1
Rippkaea sp002110465 GCA 002110465.1@NEED01000069.1 173
Limnothrix sp001693255 GCA 001693255.1@ANV85695.1
JACVSF01 sp014534085 GCA 014696715.1@MBD2258855.1
Lyngbya confervoides GCA 000817775.3@MCM1983520.1
JAAUOP01 sp938017515 GCA 938017515.1@CALIJJ010000065.1 2
CACIAM-69d sp001698445 GCA 014698235.1@MBD2590942.1
Gloeomargarita sp. GMQPbins120 GCA 039805965.1@MEO0307859.1
Gloeomargarita sp. DG15bins55 GCA 039795105.1@MEN9272712.1
Symplocastrum torsivum GCA 019358875.1@MBW4544016.1
Anabaena lutea GCA 014698305.1@MBD2570296.1
PCC9605 sp000517105 GCA 000517105.1@KI912154.1 60
Kamptonema formosum A GCA 000332155.1@KB235904.1 928
Pantanalinema sp016743235 GCA 016743235.1@MBL1174098.1
Leptolyngbya sp015206935 GCA 015206935.1@MBE9009016.1
bitscore
32
51
71
91
110
130
150
169
189
209
229
Tree scale: 1

Fig. S58 - PratA

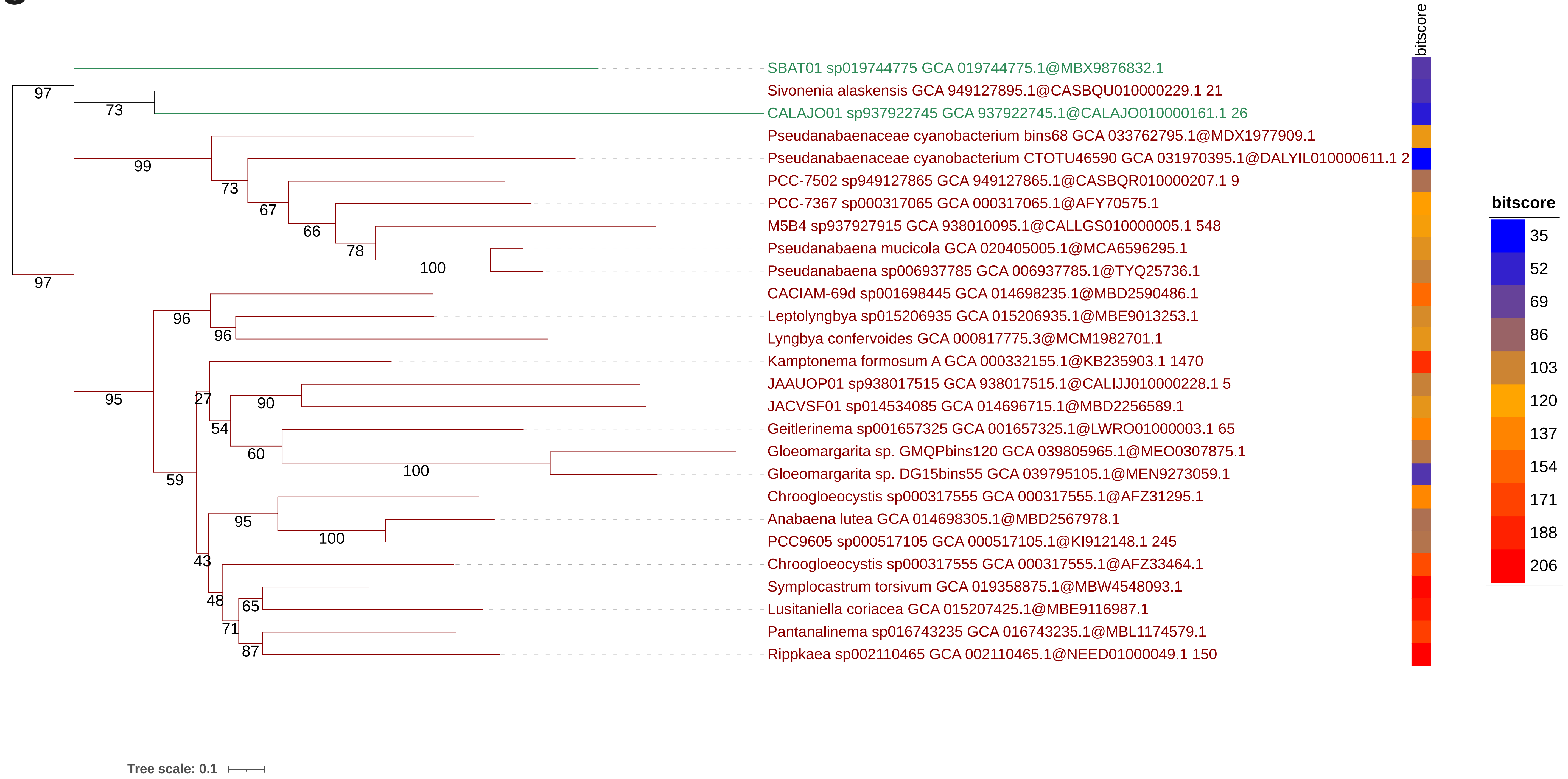

Fig. S59 - Psb27

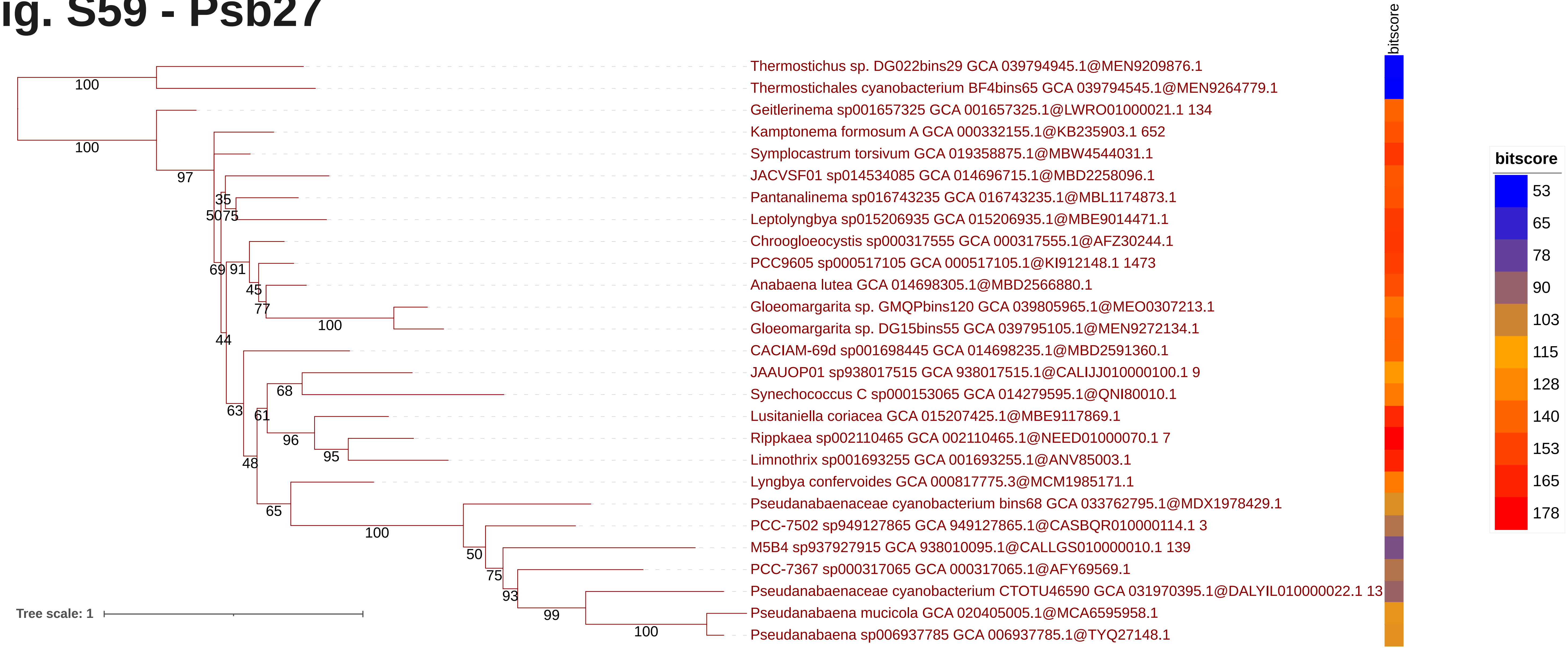

Fig. S60 - Psb28

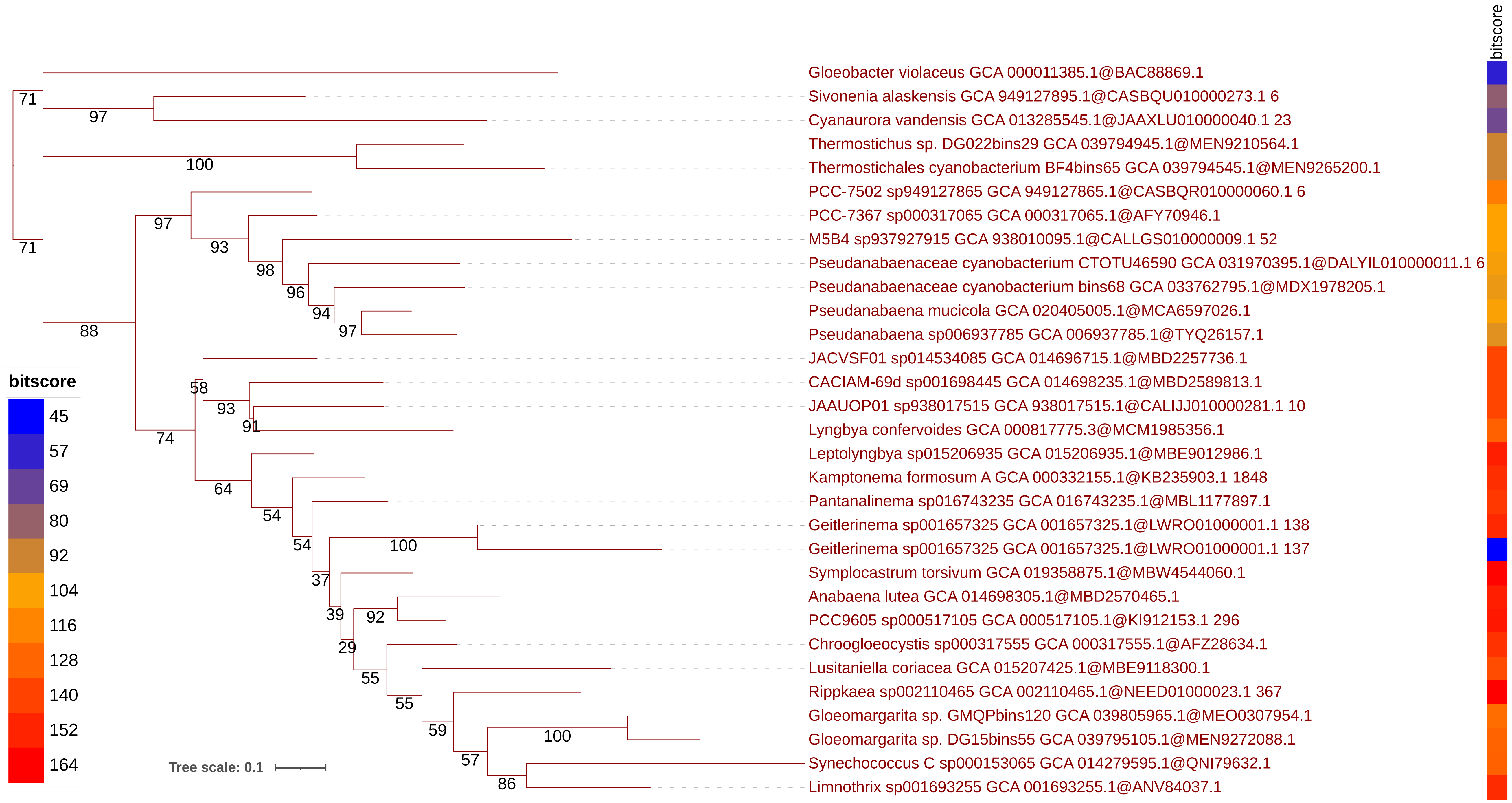

Fig. S61 - Psb29

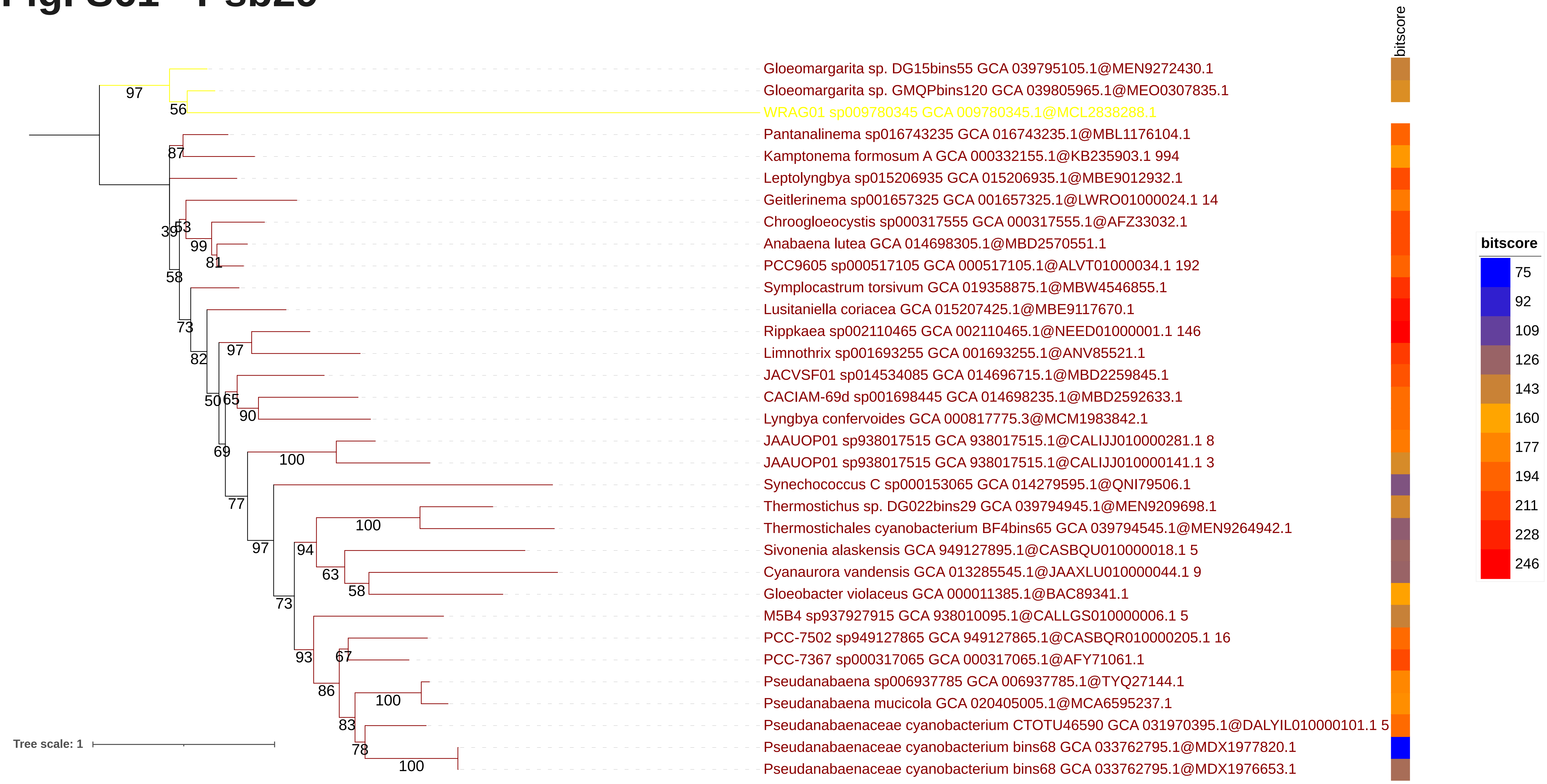

Fig. S62 - Psb32

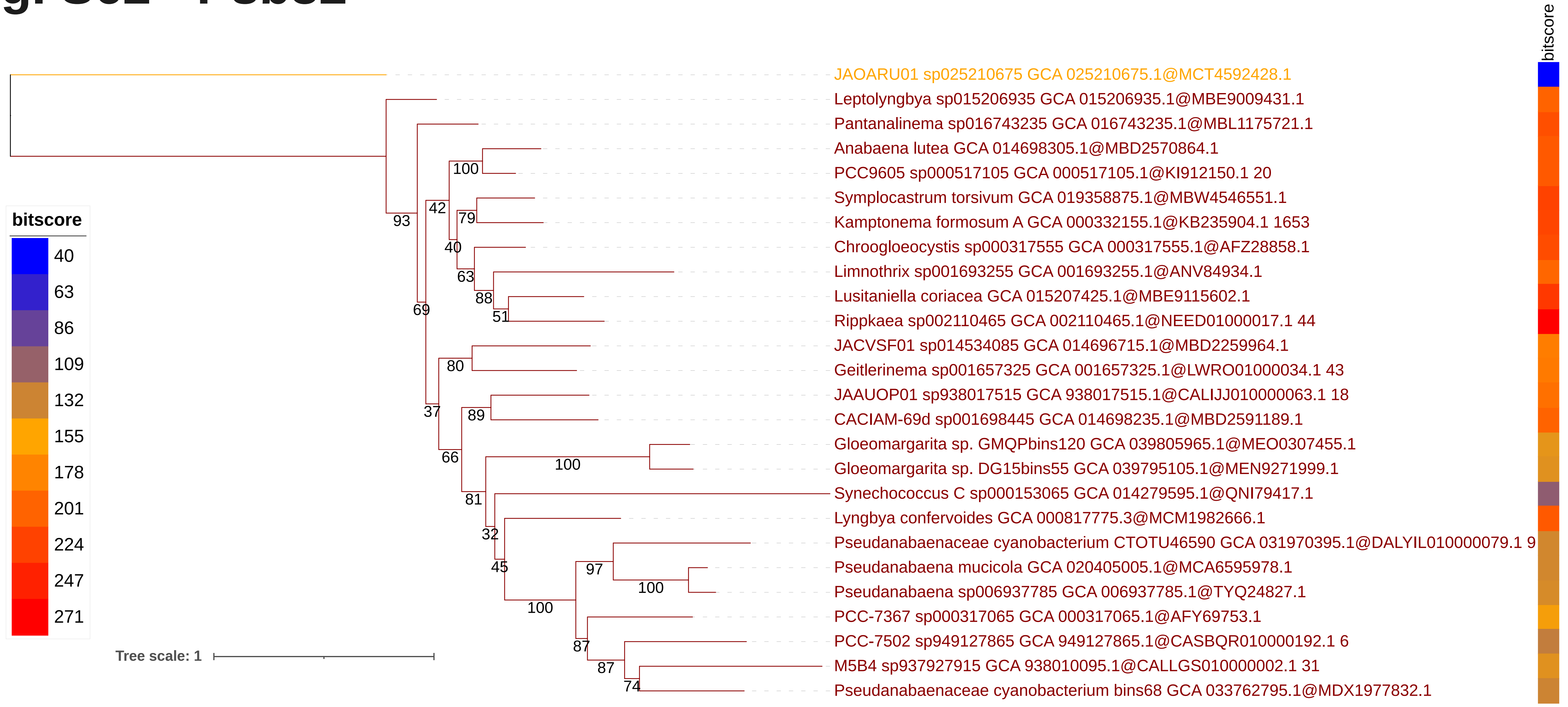

Fig. S63 - Psb34

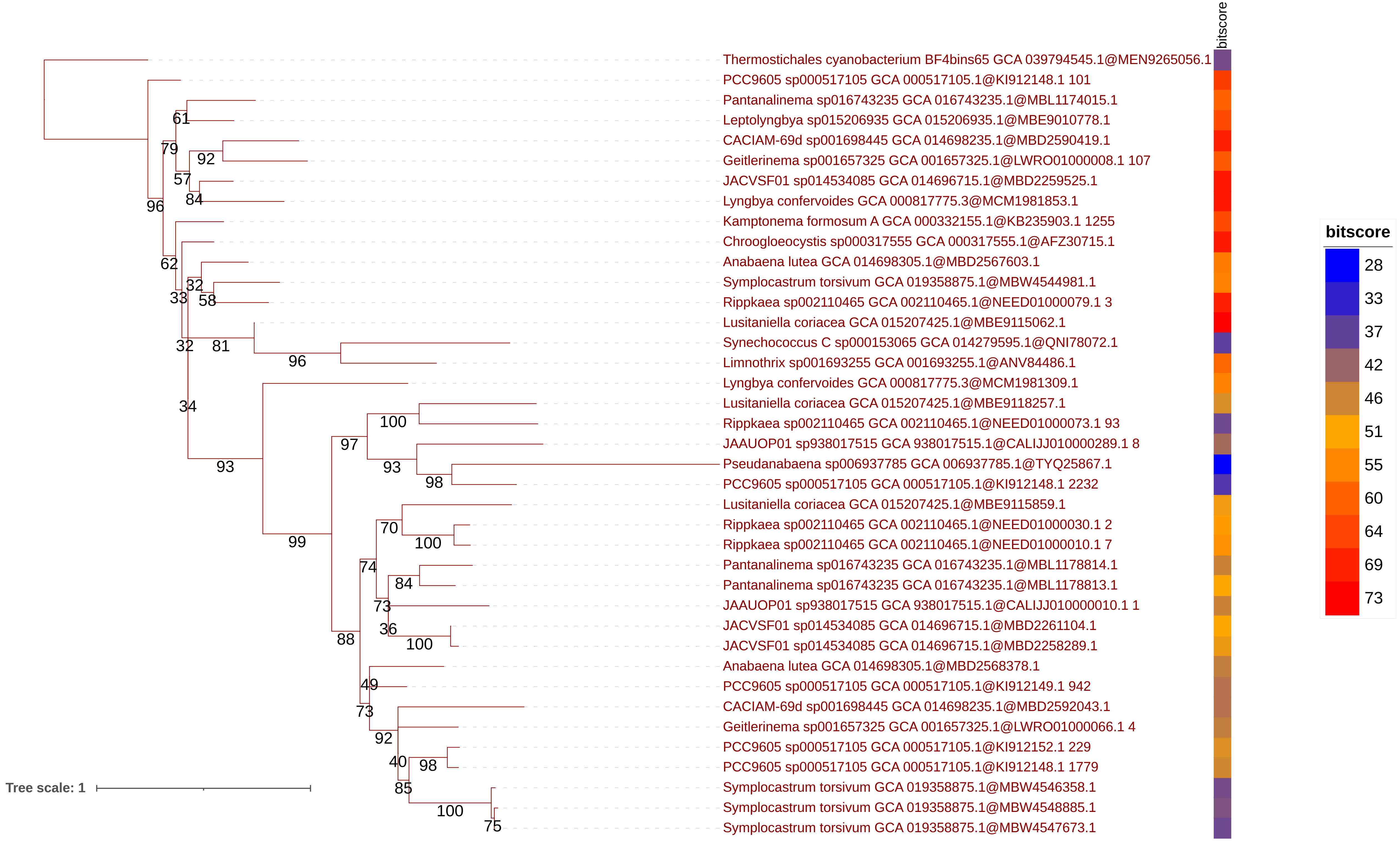

Fig. S64 - Psb35

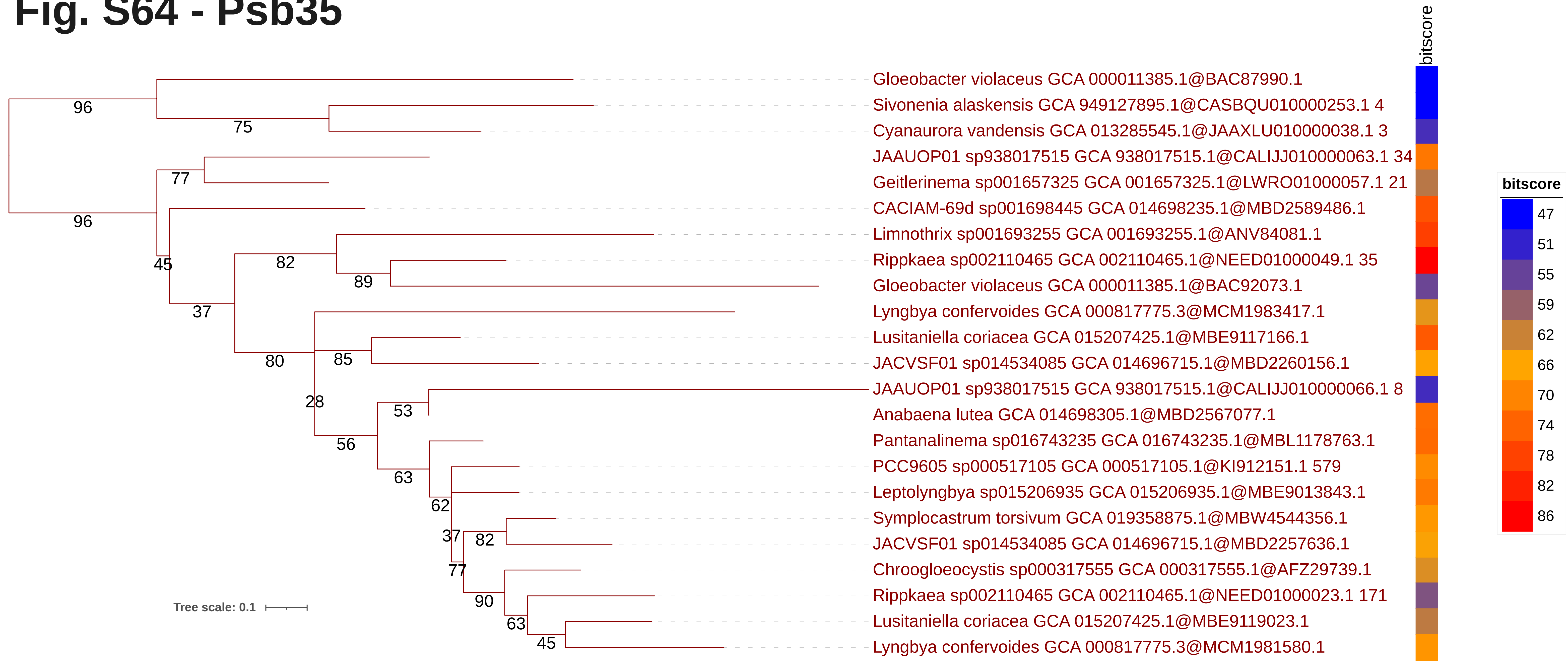

Fig. S65 - PsbN

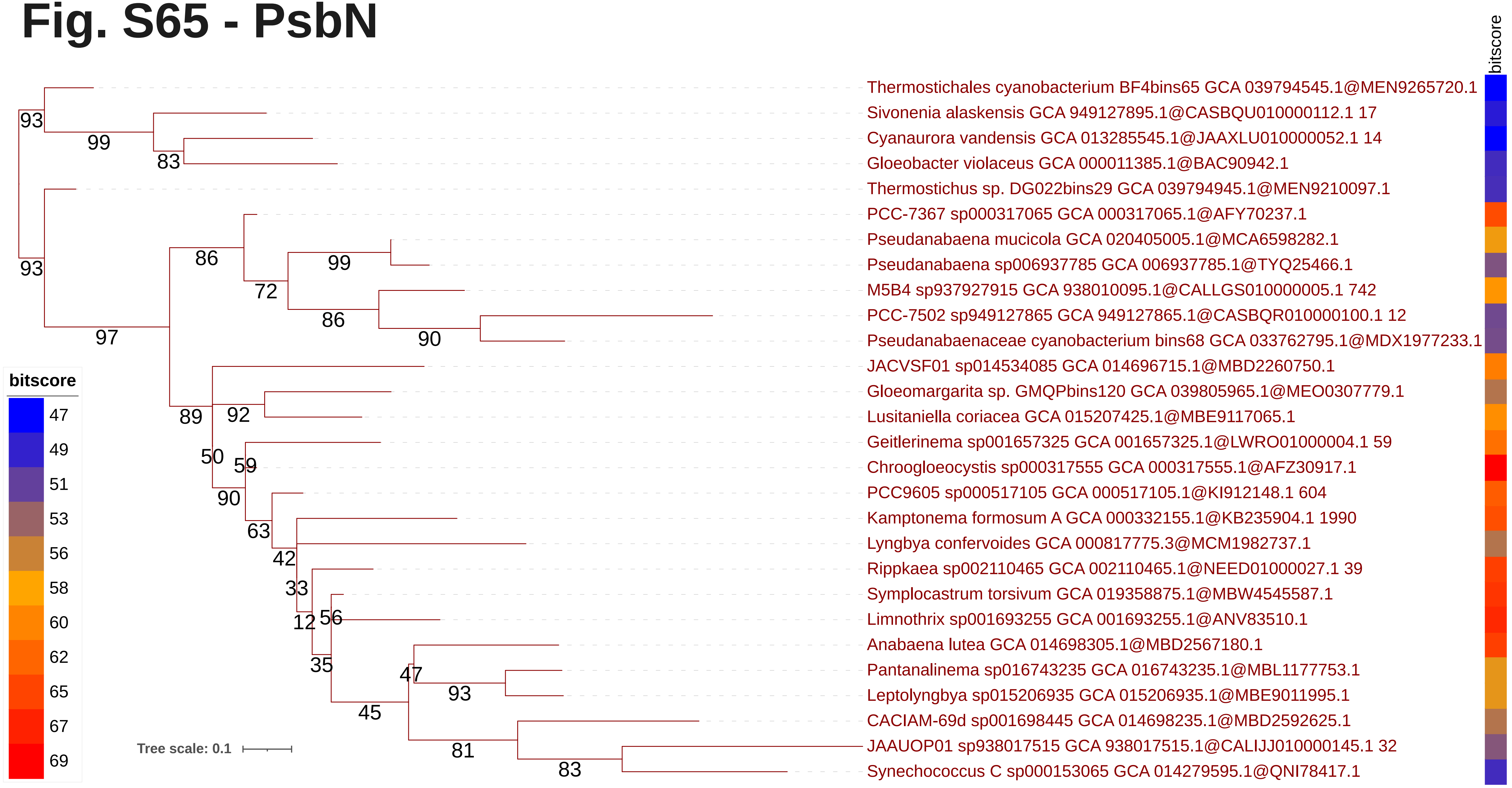
bitscore
Thermostichales cyanobacterium BF4bins65 GCA 039794545.1@MEN9265720.1
Sivonenia alaskensis GCA 949127895.1@CASBQU010000112.1 17
Cyanaurora vandensis GCA 013285545.1@JAAXLU010000052.1 14
Gloeobacter violaceus GCA 000011385.1@BAC90942.1
Thermostichus sp. DG022bins29 GCA 039794945.1@MEN9210097.1
PCC-7367 sp000317065 GCA 000317065.1@AFY70237.1
Pseudanabaena mucicola GCA 020405005.1@MCA6598282.1
Pseudanabaena sp006937785 GCA 006937785.1@TYQ25466.1
M5B4 sp937927915 GCA 938010095.1@CALLGS010000005.1 742
PCC-7502 sp949127865 GCA 949127865.1@CASBQR010000100.1 12
Pseudanabaenaceae cyanobacterium bins68 GCA 033762795.1@MDX1977233.1
JACVSF01 sp014534085 GCA 014696715.1@MBD2260750.1
Gloeomargarita sp. GMQPbins120 GCA 039805965.1@MEO0307779.1
Lusitaniella coriacea GCA 015207425.1@MBE9117065.1
Geitlerinema sp001657325 GCA 001657325.1@LWRO01000004.1 59
Chroogloeocystis sp000317555 GCA 000317555.1@AFZ30917.1
PCC9605 sp000517105 GCA 000517105.1@KI912148.1 604
Kamptonema formosum A GCA 000332155.1@KB235904.1 1990
Lyngbya confervoides GCA 000817775.3@MCM1982737.1
Rippkaea sp002110465 GCA 002110465.1@NEED01000027.1 39
Symplocastrum torsivum GCA 019358875.1@MBW4545587.1
Limnothrix sp001693255 GCA 001693255.1@ANV83510.1
Anabaena lutea GCA 014698305.1@MBD2567180.1
Pantanalinema sp016743235 GCA 016743235.1@MBL1177753.1
Leptolyngbya sp015206935 GCA 015206935.1@MBE9011995.1
CACIAM-69d sp001698445 GCA 014698235.1@MBD2592625.1
JAAUOP01 sp938017515 GCA 938017515.1@CALIJJ010000145.1 32
Synechococcus C sp000153065 GCA 014279595.1@QNI78417.1
93
99
83
93
86
99
72
86
90
97
89
92
50
59
90
63
42
33
12
56
35
47
93
45
81
83
bitscore
47
49
51
53
56
58
60
62
65
67
69
Tree scale: 0.1

Fig. S66 - RubA

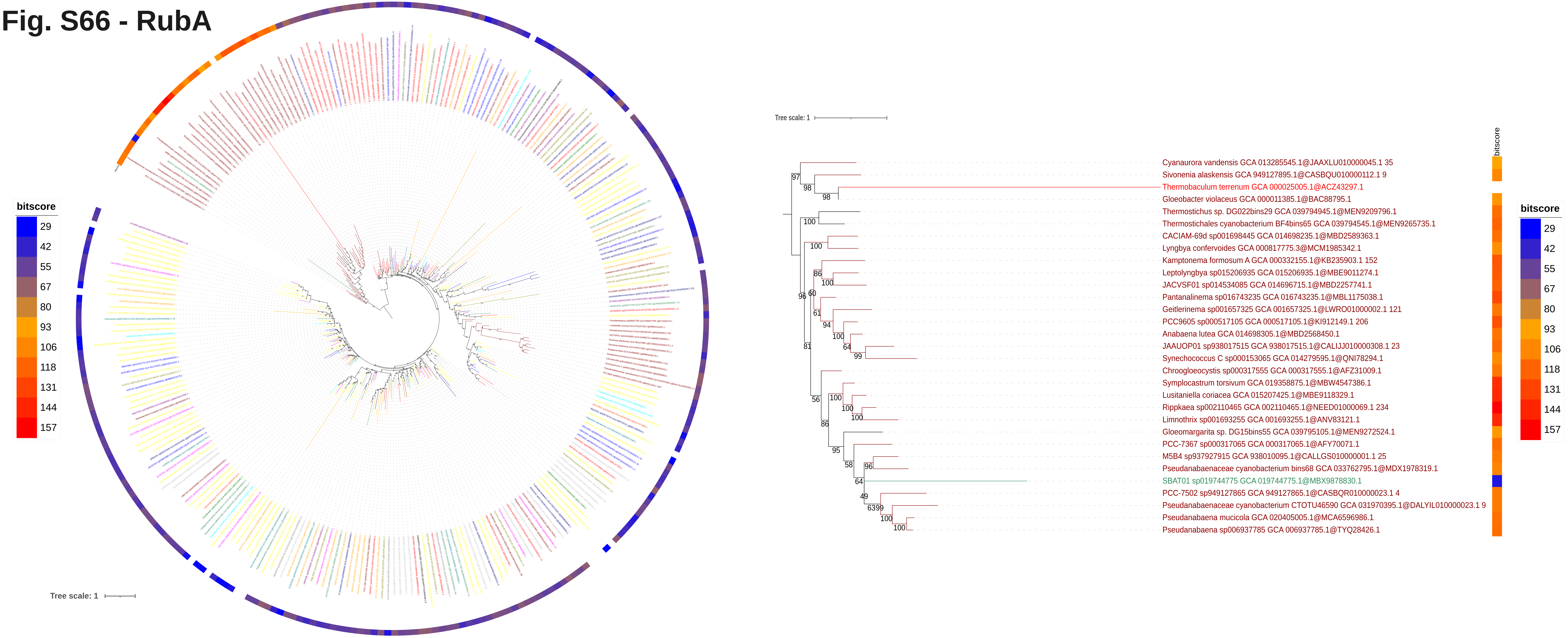

bitscore
29
42
55
67
80
93
106
118
131
144
157
Tree scale: 1
Tree scale: 1
bitscore
Cyanaurora vandensis GCA 013285545.1@JAAXLU010000045.1 35
Sivonenia alaskensis GCA 949127895.1@CASBQU010000112.1 9
Thermobaculum terrenum GCA 000025005.1@ACZ43297.1
Gloeobacter violaceus GCA 000011385.1@BAC88795.1
Thermostichus sp. DG022bins29 GCA 039794945.1@MEN9209796.1
Thermostichales cyanobacterium BF4bins65 GCA 039794545.1@MEN9265735.1
CACIAM-69d sp001698445 GCA 014698235.1@MBD2589363.1
Lyngbya confervoides GCA 000817775.3@MCM1985342.1
Kamptonema formosum A GCA 000332155.1@KB235903.1 152
Leptolyngbya sp015206935 GCA 015206935.1@MBE9011274.1
JACVSF01 sp014534085 GCA 014696715.1@MBD2257741.1
Pantanalinema sp016743235 GCA 016743235.1@MBL1175038.1
Geitlerinema sp001657325 GCA 001657325.1@LWRO01000002.1 121
PCC9605 sp000517105 GCA 000517105.1@KI912149.1 206
Anabaena lutea GCA 014698305.1@MBD2568450.1
JAAUOP01 sp938017515 GCA 938017515.1@CALIJJ010000308.1 23
Synechococcus C sp000153065 GCA 014279595.1@QNI78294.1
Chroogloeocystis sp000317555 GCA 000317555.1@AFZ31009.1
Symplocastrum torsivum GCA 019358875.1@MBW4547386.1
Lusitaniella coriacea GCA 015207425.1@MBE9118329.1
Rippkaea sp002110465 GCA 002110465.1@NEED01000069.1 234
Limnothrix sp001693255 GCA 001693255.1@ANV83121.1
Gloeomargarita sp. DG15bins55 GCA 039795105.1@MEN9272524.1
PCC-7367 sp000317065 GCA 000317065.1@AFY70071.1
M5B4 sp937927915 GCA 938010095.1@CALLGS010000001.1 25
Pseudanabaenaceae cyanobacterium bins68 GCA 033762795.1@MDX1978319.1
SBAT01 sp019744775 GCA 019744775.1@MBX9878830.1
PCC-7502 sp949127865 GCA 949127865.1@CASBQR010000023.1 4
Pseudanabaenaceae cyanobacterium CTOTU46590 GCA 031970395.1@DALYIL010000023.1 9
Pseudanabaena mucicola GCA 020405005.1@MCA6596986.1
Pseudanabaena sp006937785 GCA 006937785.1@TYQ28426.1
bitscore
29
42
55
67
80
93
106
118
131
144
157

Fig. S67 - SecD

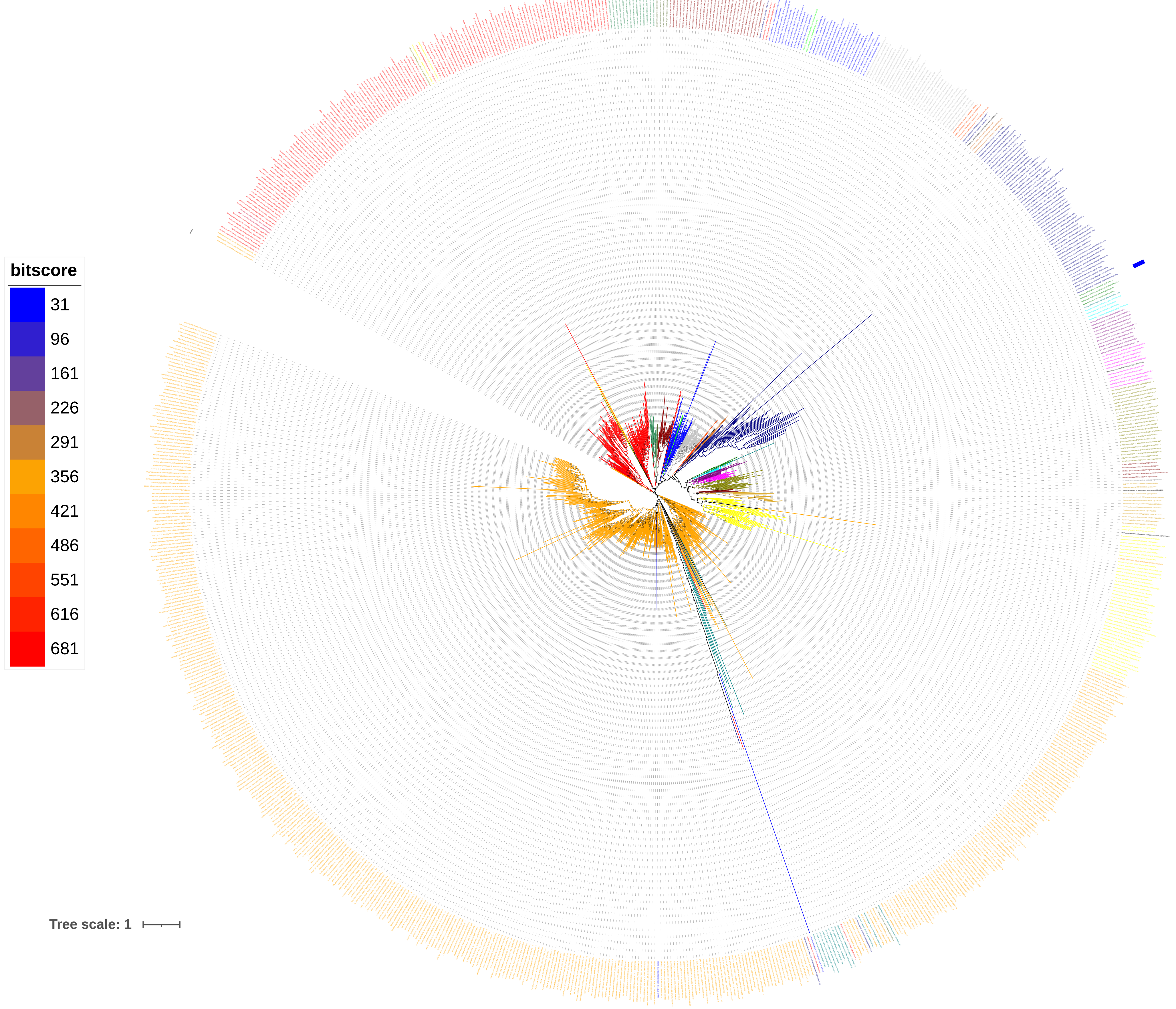
bitscore
31
96
161
226
291
356
421
486
551
616
681
Tree scale: 1

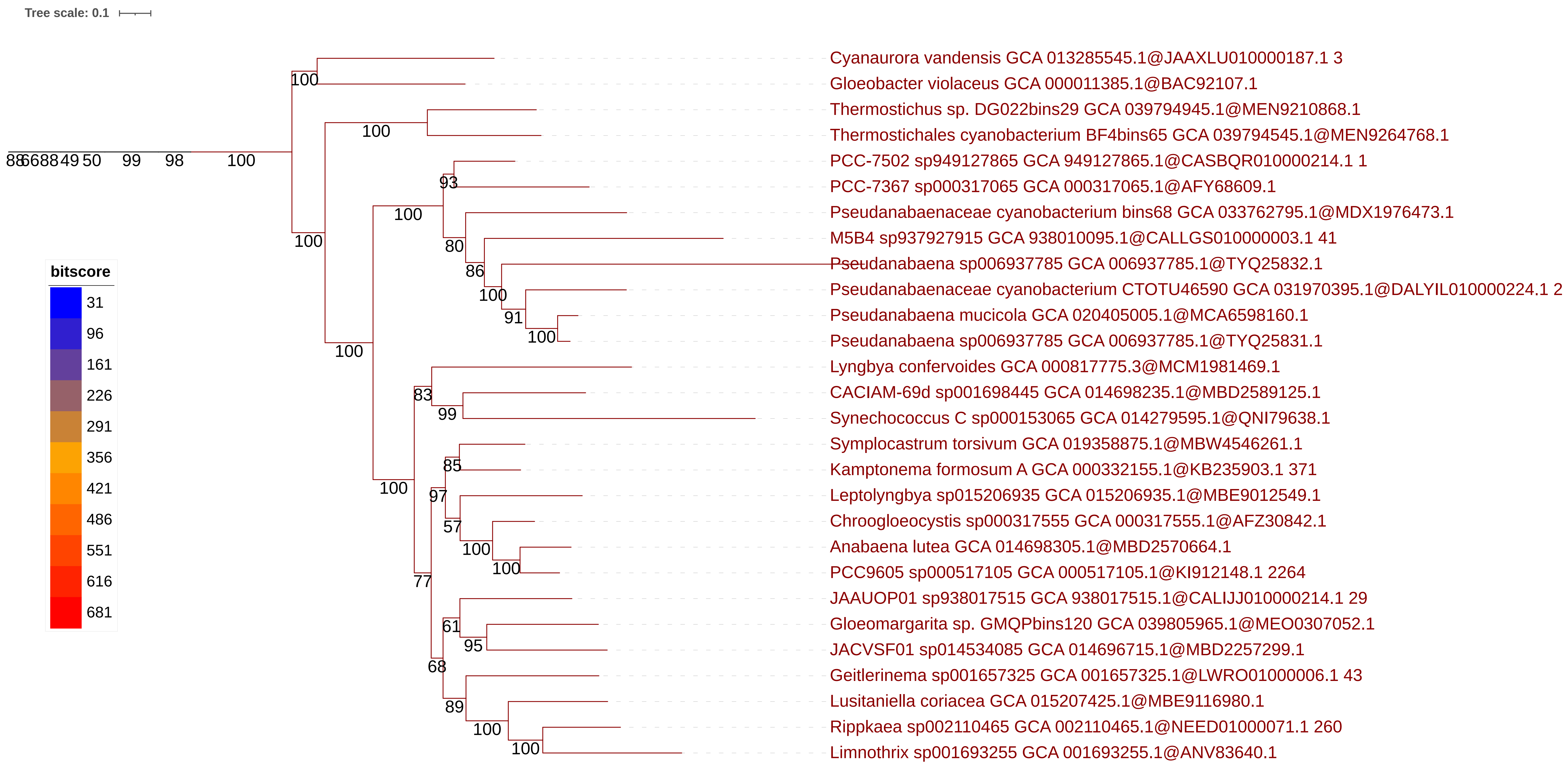
Tree scale: 0.1
bitscore
31
96
161
226
291
356
421
486
551
616
681
Cyanaurora vandensis GCA 013285545.1@JAAXLU010000187.1 3
Gloeobacter violaceus GCA 000011385.1@BAC92107.1
Thermostichus sp. DG022bins29 GCA 039794945.1@MEN9210868.1
Thermostichales cyanobacterium BF4bins65 GCA 039794545.1@MEN9264768.1
PCC-7502 sp949127865 GCA 949127865.1@CASBQR010000214.1 1
PCC-7367 sp000317065 GCA 000317065.1@AFY68609.1
Pseudanabaenaceae cyanobacterium bins68 GCA 033762795.1@MDX1976473.1
M5B4 sp937927915 GCA 938010095.1@CALLGS010000003.1 41
Pseudanabaena sp006937785 GCA 006937785.1@TYQ25832.1
Pseudanabaenaceae cyanobacterium CTOTU46590 GCA 031970395.1@DALYIL010000224.1 2
Pseudanabaena mucicola GCA 020405005.1@MCA6598160.1
Pseudanabaena sp006937785 GCA 006937785.1@TYQ25831.1
Lyngbya confervoides GCA 000817775.3@MCM1981469.1
CACIAM-69d sp001698445 GCA 014698235.1@MBD2589125.1
Synechococcus C sp000153065 GCA 014279595.1@QNI79638.1
Symplocastrum torsivum GCA 019358875.1@MBW4546261.1
Kamptonema formosum A GCA 000332155.1@KB235903.1 371
Leptolyngbya sp015206935 GCA 015206935.1@MBE9012549.1
Chroogloeocystis sp000317555 GCA 000317555.1@AFZ30842.1
Anabaena lutea GCA 014698305.1@MBD2570664.1
PCC9605 sp000517105 GCA 000517105.1@KI912148.1 2264
JAAUOP01 sp938017515 GCA 938017515.1@CALIJJ010000214.1 29
Gloeomargarita sp. GMQPbins120 GCA 039805965.1@MEO0307052.1
JACVSF01 sp014534085 GCA 014696715.1@MBD2257299.1
Geitlerinema sp001657325 GCA 001657325.1@LWRO01000006.1 43
Lusitaniella coriacea GCA 015207425.1@MBE9116980.1
Rippkaea sp002110465 GCA 002110465.1@NEED01000071.1 260
Limnothrix sp001693255 GCA 001693255.1@ANV83640.1

Fig. S68 - SecE

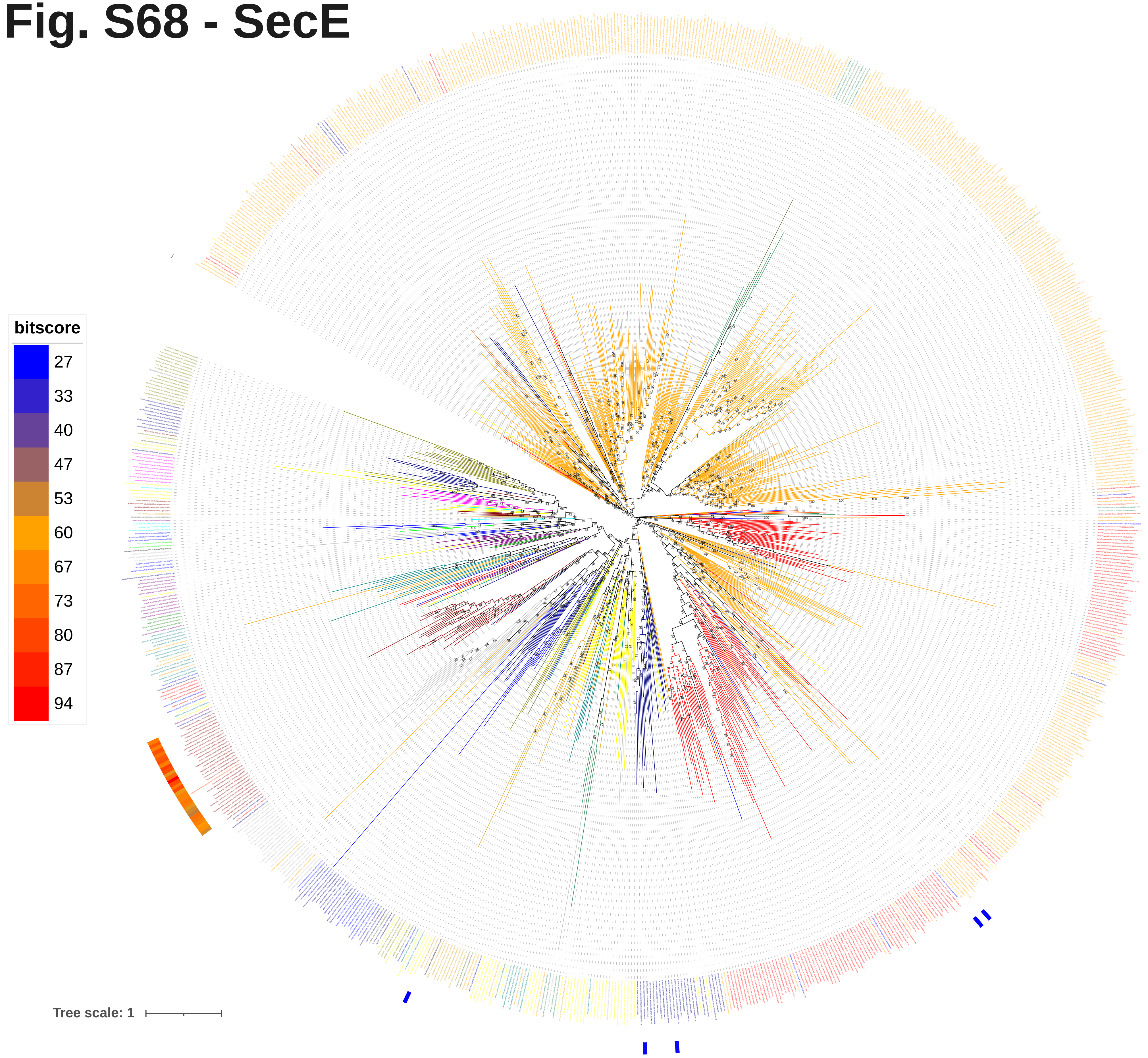
bitscore
27
33
40
47
53
60
67
73
80
87
94
Tree scale: 1

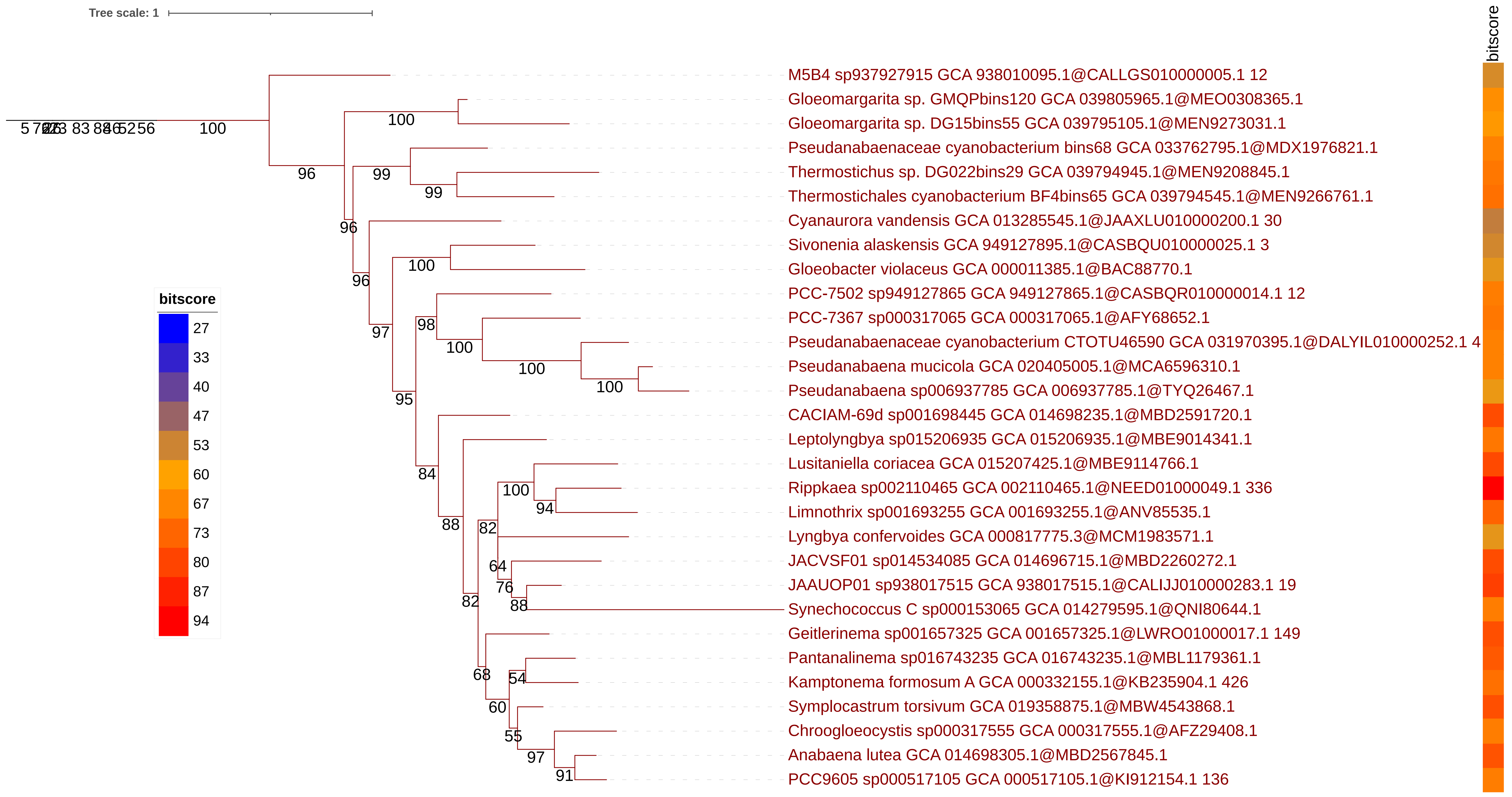
Tree scale: 1
bitscore
27
33
40
47
53
60
67
73
80
87
94
M5B4 sp937927915 GCA 938010095.1@CALLGS010000005.1 12
Gloeomargarita sp. GMQPbins120 GCA 039805965.1@MEO0308365.1
Gloeomargarita sp. DG15bins55 GCA 039795105.1@MEN9273031.1
Pseudanabaenaceae cyanobacterium bins68 GCA 033762795.1@MDX1976821.1
Thermostichus sp. DG022bins29 GCA 039794945.1@MEN9208845.1
Thermostichales cyanobacterium BF4bins65 GCA 039794545.1@MEN9266761.1
Cyanaurora vandensis GCA 013285545.1@JAAXLU010000200.1 30
Sivonenia alaskensis GCA 949127895.1@CASBQU010000025.1 3
Gloeobacter violaceus GCA 000011385.1@BAC88770.1
PCC-7502 sp949127865 GCA 949127865.1@CASBQR010000014.1 12
PCC-7367 sp000317065 GCA 000317065.1@AFY68652.1
Pseudanabaenaceae cyanobacterium CTOTU46590 GCA 031970395.1@DALYIL010000252.1 4
Pseudanabaena mucicola GCA 020405005.1@MCA6596310.1
Pseudanabaena sp006937785 GCA 006937785.1@TYQ26467.1
CACIAM-69d sp001698445 GCA 014698235.1@MBD2591720.1
Leptolyngbya sp015206935 GCA 015206935.1@MBE9014341.1
Lusitaniella coriacea GCA 015207425.1@MBE9114766.1
Rippkaea sp002110465 GCA 002110465.1@NEED01000049.1 336
Limnothrix sp001693255 GCA 001693255.1@ANV85535.1
Lyngbya confervoides GCA 000817775.3@MCM1983571.1
JACVSF01 sp014534085 GCA 014696715.1@MBD2260272.1
JAAUOP01 sp938017515 GCA 938017515.1@CALIJJ010000283.1 19
Synechococcus C sp000153065 GCA 014279595.1@QNI80644.1
Geitlerinema sp001657325 GCA 001657325.1@LWRO01000017.1 149
Pantanalinema sp016743235 GCA 016743235.1@MBL1179361.1
Kamptonema formosum A GCA 000332155.1@KB235904.1 426
Symplocastrum torsivum GCA 019358875.1@MBW4543868.1
Chroogloeocystis sp000317555 GCA 000317555.1@AFZ29408.1
Anabaena lutea GCA 014698305.1@MBD2567845.1
PCC9605 sp000517105 GCA 000517105.1@KI912154.1 136

Fig. S69 - SecF

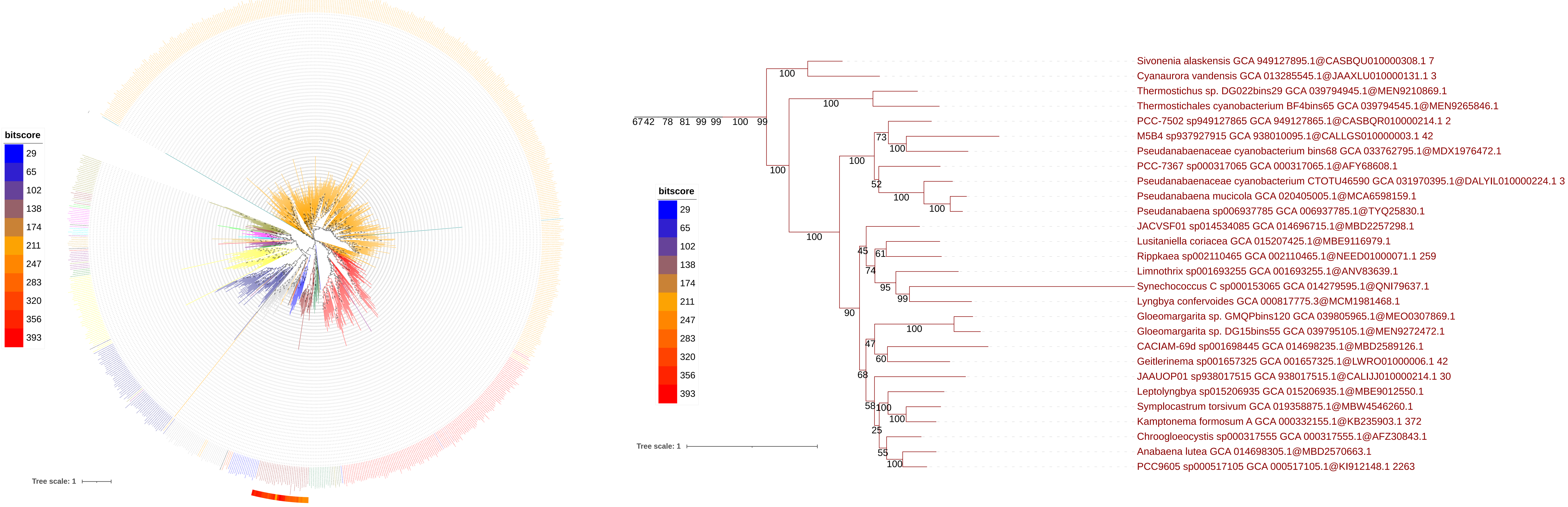
bitscore
29
65
102
138
174
211
247
283
320
356
393
Tree scale: 1
Sivonenia alaskensis GCA 949127895.1@CASBQU010000308.1 7
Cyanaurora vandensis GCA 013285545.1@JAAXLU010000131.1 3
Thermostichus sp. DG022bins29 GCA 039794945.1@MEN9210869.1
Thermostichales cyanobacterium BF4bins65 GCA 039794545.1@MEN9265846.1
PCC-7502 sp949127865 GCA 949127865.1@CASBQR010000214.1 2
M5B4 sp937927915 GCA 938010095.1@CALLGS010000003.1 42
Pseudanabaenaceae cyanobacterium bins68 GCA 033762795.1@MDX1976472.1
PCC-7367 sp000317065 GCA 000317065.1@AFY68608.1
Pseudanabaenaceae cyanobacterium CTOTU46590 GCA 031970395.1@DALYIL010000224.1 3
Pseudanabaena mucicola GCA 020405005.1@MCA6598159.1
Pseudanabaena sp006937785 GCA 006937785.1@TYQ25830.1
JACVSF01 sp014534085 GCA 014696715.1@MBD2257298.1
Lusitaniella coriacea GCA 015207425.1@MBE9116979.1
Rippkaea sp002110465 GCA 002110465.1@NEED01000071.1 259
Limnothrix sp001693255 GCA 001693255.1@ANV83639.1
Synechococcus C sp000153065 GCA 014279595.1@QNI79637.1
Lyngbya confervoides GCA 000817775.3@MCM1981468.1
Gloeomargarita sp. GMQPbins120 GCA 039805965.1@MEO0307869.1
Gloeomargarita sp. DG15bins55 GCA 039795105.1@MEN9272472.1
CACIAM-69d sp001698445 GCA 014698235.1@MBD2589126.1
Geitlerinema sp001657325 GCA 001657325.1@LWRO01000006.1 42
JAAUOP01 sp938017515 GCA 938017515.1@CALIJJ010000214.1 30
Leptolyngbya sp015206935 GCA 015206935.1@MBE9012550.1
Symplocastrum torsivum GCA 019358875.1@MBW4546260.1
Kamptonema formosum A GCA 000332155.1@KB235903.1 372
Chroogloeocystis sp000317555 GCA 000317555.1@AFZ30843.1
Anabaena lutea GCA 014698305.1@MBD2570663.1
PCC9605 sp000517105 GCA 000517105.1@KI912148.1 2263

Fig. S70 - SecG

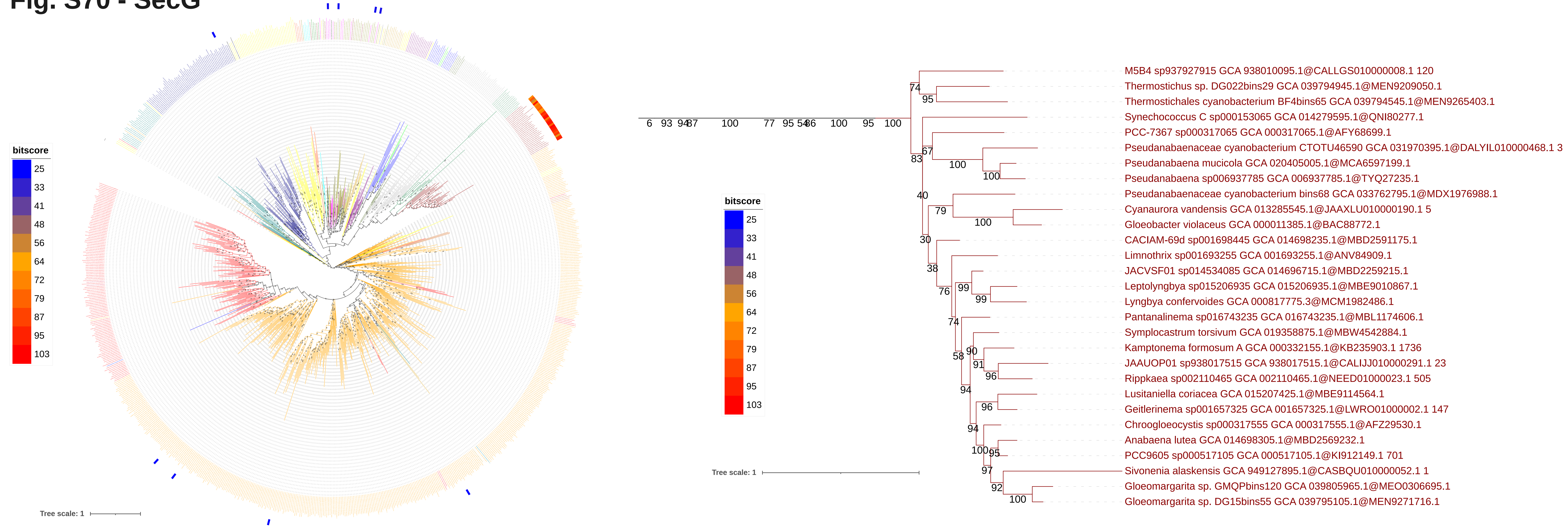
bitscore
25
33
41
48
56
64
72
79
87
95
103
Tree scale: 1
M5B4 sp937927915 GCA 938010095.1@CALLGS010000008.1 120
Thermostichus sp. DG022bins29 GCA 039794945.1@MEN9209050.1
Thermostichales cyanobacterium BF4bins65 GCA 039794545.1@MEN9265403.1
Synechococcus C sp000153065 GCA 014279595.1@QNI80277.1
PCC-7367 sp000317065 GCA 000317065.1@AFY68699.1
Pseudanabaenaceae cyanobacterium CTOTU46590 GCA 031970395.1@DALYIL010000468.1 3
Pseudanabaena mucicola GCA 020405005.1@MCA6597199.1
Pseudanabaena sp006937785 GCA 006937785.1@TYQ27235.1
Pseudanabaenaceae cyanobacterium bins68 GCA 033762795.1@MDX1976988.1
Cyanaurora vandensis GCA 013285545.1@JAAXLU010000190.1 5
Gloeobacter violaceus GCA 000011385.1@BAC88772.1
CACIAM-69d sp001698445 GCA 014698235.1@MBD2591175.1
Limnothrix sp001693255 GCA 001693255.1@ANV84909.1
JACVSF01 sp014534085 GCA 014696715.1@MBD2259215.1
Leptolyngbya sp015206935 GCA 015206935.1@MBE9010867.1
Lyngbya confervoides GCA 000817775.3@MCM1982486.1
Pantanalinema sp016743235 GCA 016743235.1@MBL1174606.1
Symplocastrum torsivum GCA 019358875.1@MBW4542884.1
Kamptonema formosum A GCA 000332155.1@KB235903.1 1736
JAAUOP01 sp938017515 GCA 938017515.1@CALIJJ010000291.1 23
Rippkaea sp002110465 GCA 002110465.1@NEED01000023.1 505
Lusitaniella coriacea GCA 015207425.1@MBE9114564.1
Geitlerinema sp001657325 GCA 001657325.1@LWRO01000002.1 147
Chroogloeocystis sp000317555 GCA 000317555.1@AFZ29530.1
Anabaena lutea GCA 014698305.1@MBD2569232.1
PCC9605 sp000517105 GCA 000517105.1@KI912149.1 701
Sivonenia alaskensis GCA 949127895.1@CASBQU010000052.1 1
Gloeomargarita sp. GMQPbins120 GCA 039805965.1@MEO0306695.1
Gloeomargarita sp. DG15bins55 GCA 039795105.1@MEN9271716.1
bitscore
25
33
41
48
56
64
72
79
87
95
103
Tree scale: 1

Fig. S71 - SecY

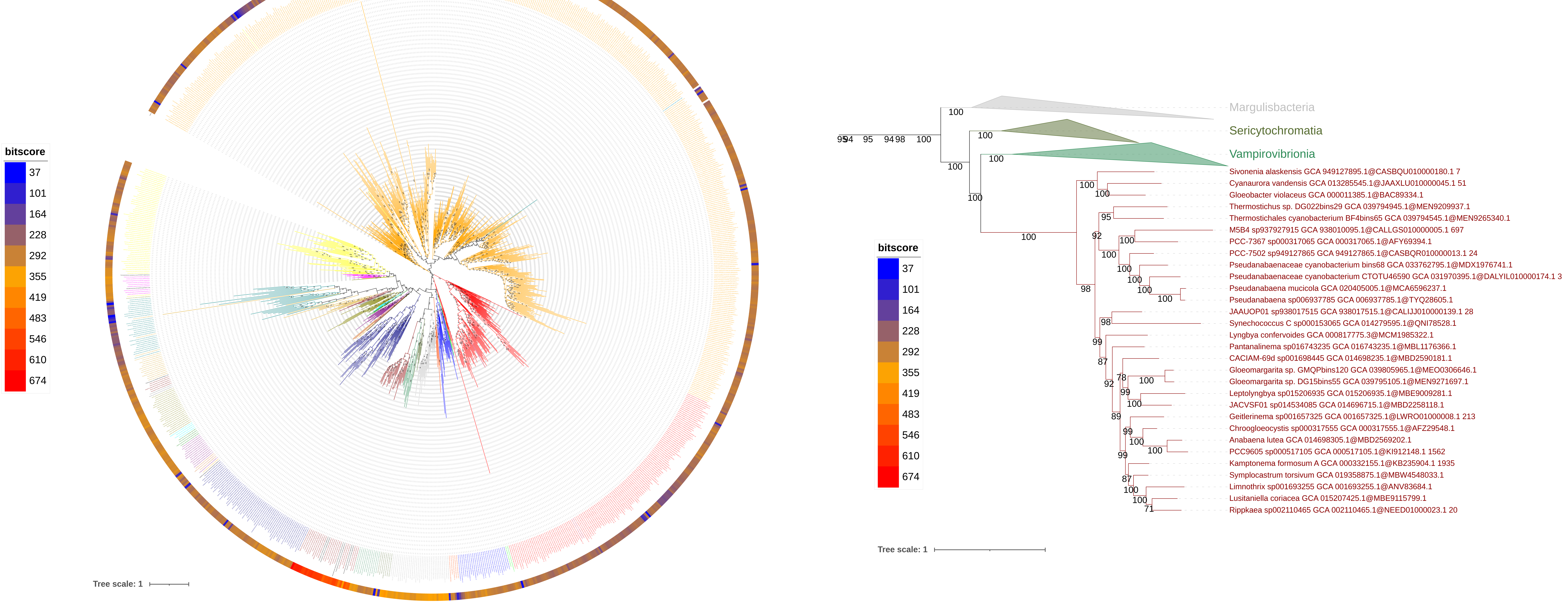
bitscore
37
101
164
228
292
355
419
483
546
610
674
Tree scale: 1
Margulisbacteria
Sericytochromatia
Vampirovibrionia
Sivonenia alaskensis GCA 949127895.1@CASBQU010000180.1 7
Cyanaurora vandensis GCA 013285545.1@JAAXLU010000045.1 51
Gloeobacter violaceus GCA 000011385.1@BAC89334.1
Thermostichus sp. DG022bins29 GCA 039794945.1@MEN9209937.1
Thermostichales cyanobacterium BF4bins65 GCA 039794545.1@MEN9265340.1
M5B4 sp937927915 GCA 938010095.1@CALLGS010000005.1 697
PCC-7367 sp000317065 GCA 000317065.1@AFY69394.1
PCC-7502 sp949127865 GCA 949127865.1@CASBQR010000013.1 24
Pseudanabaenaceae cyanobacterium bins68 GCA 033762795.1@MDX1976741.1
Pseudanabaenaceae cyanobacterium CTOTU46590 GCA 031970395.1@DALYIL010000174.1 3
Pseudanabaena mucicola GCA 020405005.1@MCA6596237.1
Pseudanabaena sp006937785 GCA 006937785.1@TYQ28605.1
JAAUOP01 sp938017515 GCA 938017515.1@CALIJJ010000139.1 28
Synechococcus C sp000153065 GCA 014279595.1@QNI78528.1
Lyngbya confervoides GCA 000817775.3@MCM1985322.1
Pantanalinema sp016743235 GCA 016743235.1@MBL1176366.1
CACIAM-69d sp001698445 GCA 014698235.1@MBD2590181.1
Gloeomargarita sp. GMQPbins120 GCA 039805965.1@MEO0306646.1
Gloeomargarita sp. DG15bins55 GCA 039795105.1@MEN9271697.1
Leptolyngbya sp015206935 GCA 015206935.1@MBE9009281.1
JACVSF01 sp014534085 GCA 014696715.1@MBD2258118.1
Geitlerinema sp001657325 GCA 001657325.1@LWRO01000008.1 213
Chroogloeocystis sp000317555 GCA 000317555.1@AFZ29548.1
Anabaena lutea GCA 014698305.1@MBD2569202.1
PCC9605 sp000517105 GCA 000517105.1@KI912148.1 1562
Kamptonema formosum A GCA 000332155.1@KB235904.1 1935
Symplocastrum torsivum GCA 019358875.1@MBW4548033.1
Limnothrix sp001693255 GCA 001693255.1@ANV83684.1
Lusitaniella coriacea GCA 015207425.1@MBE9115799.1
Rippkaea sp002110465 GCA 002110465.1@NEED01000023.1 20
Tree scale: 1

Fig. S72 - Sll0408 (TLP40)

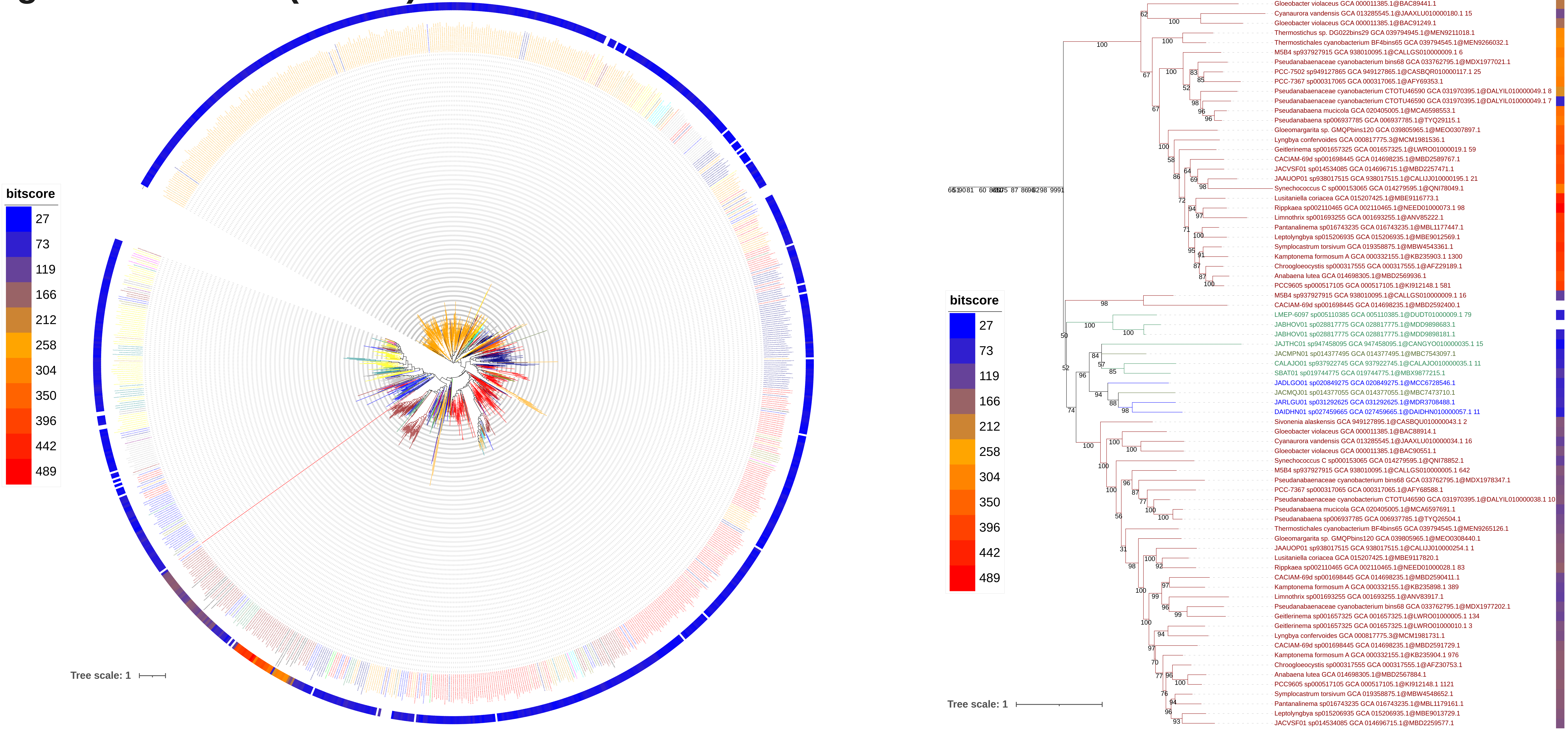

Fig. S73 - Sll0509

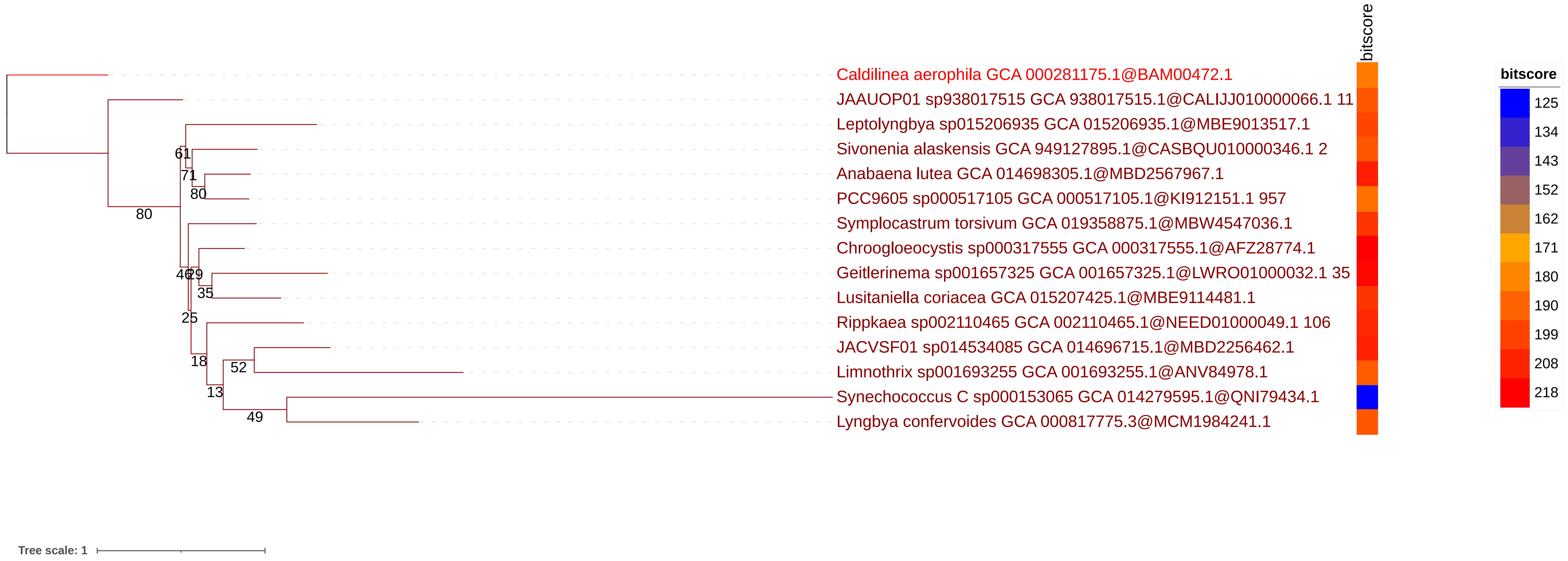

Fig. S74 - Sll0606

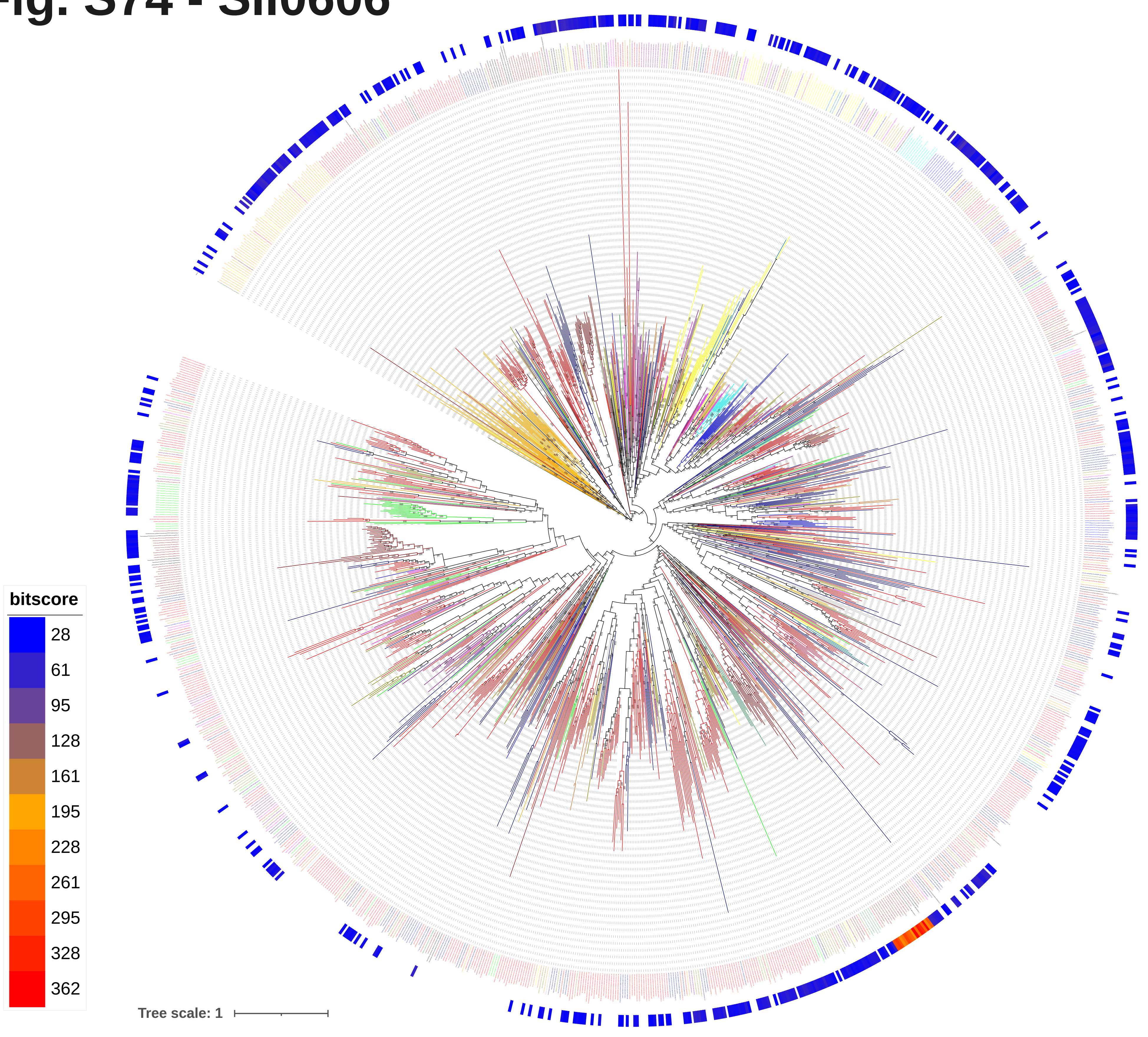

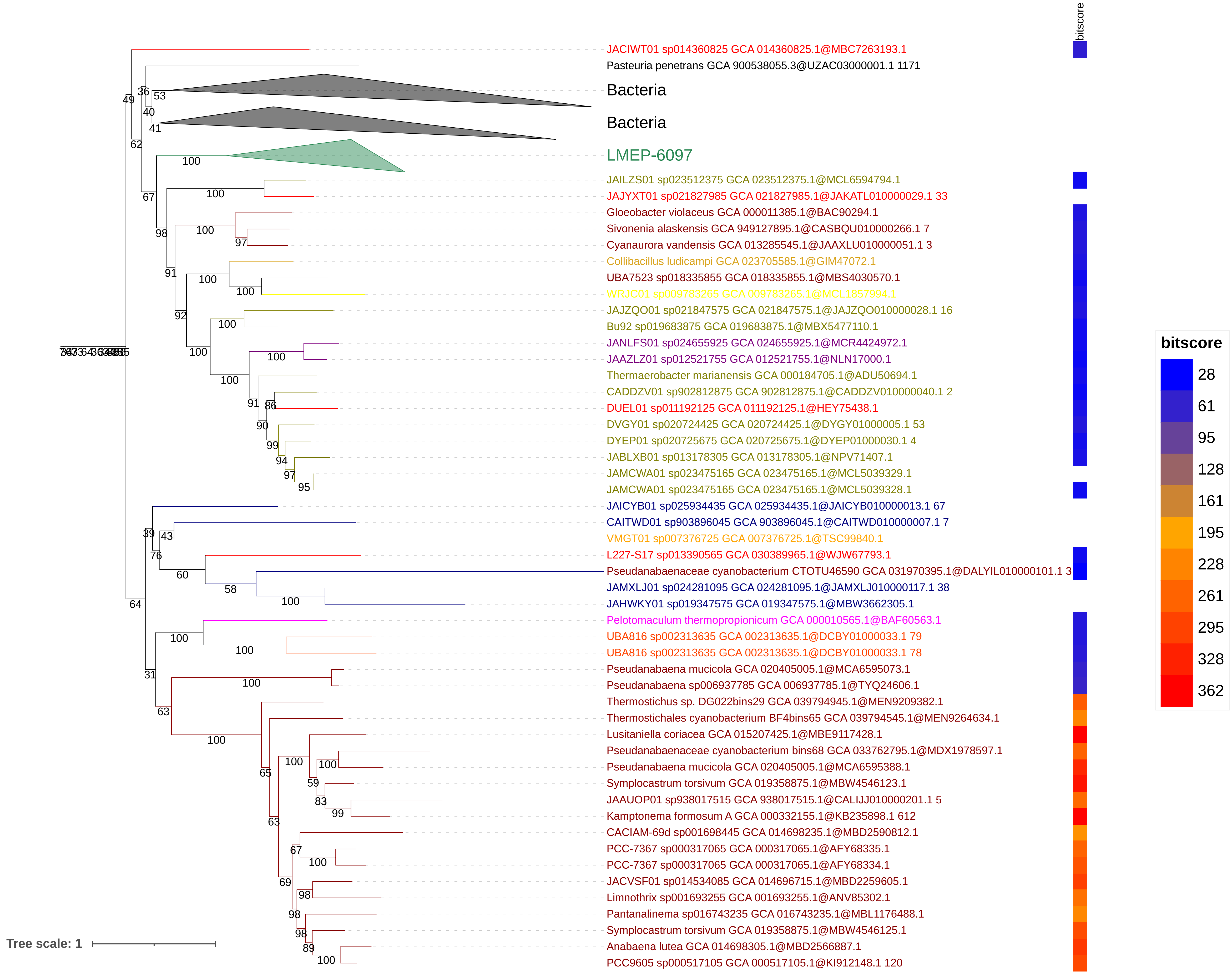

Fig. S75 - Sll0933 (Pam68)

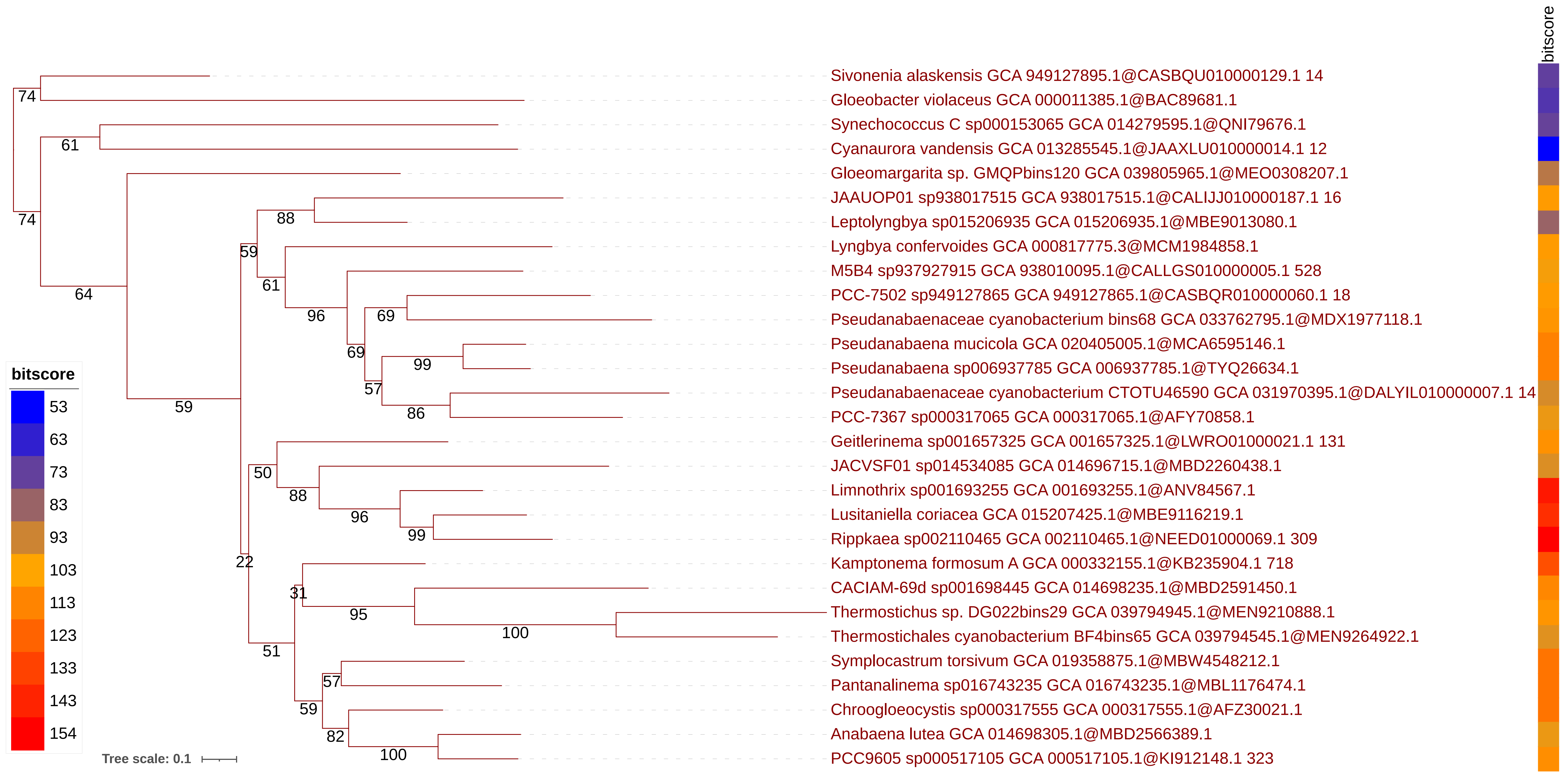

Fig. S76 - Sll1021

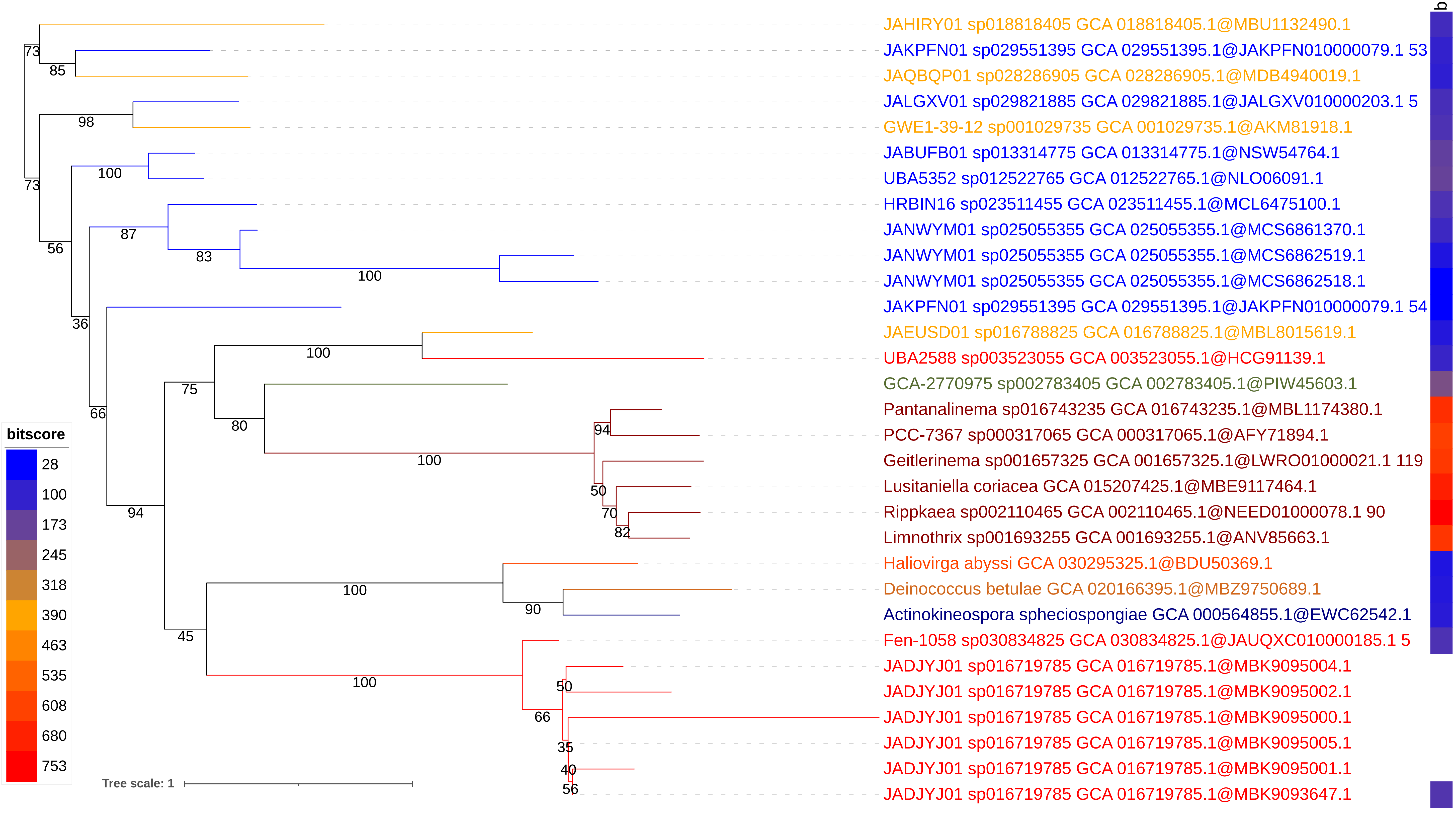

Fig. S77 - Slr0144

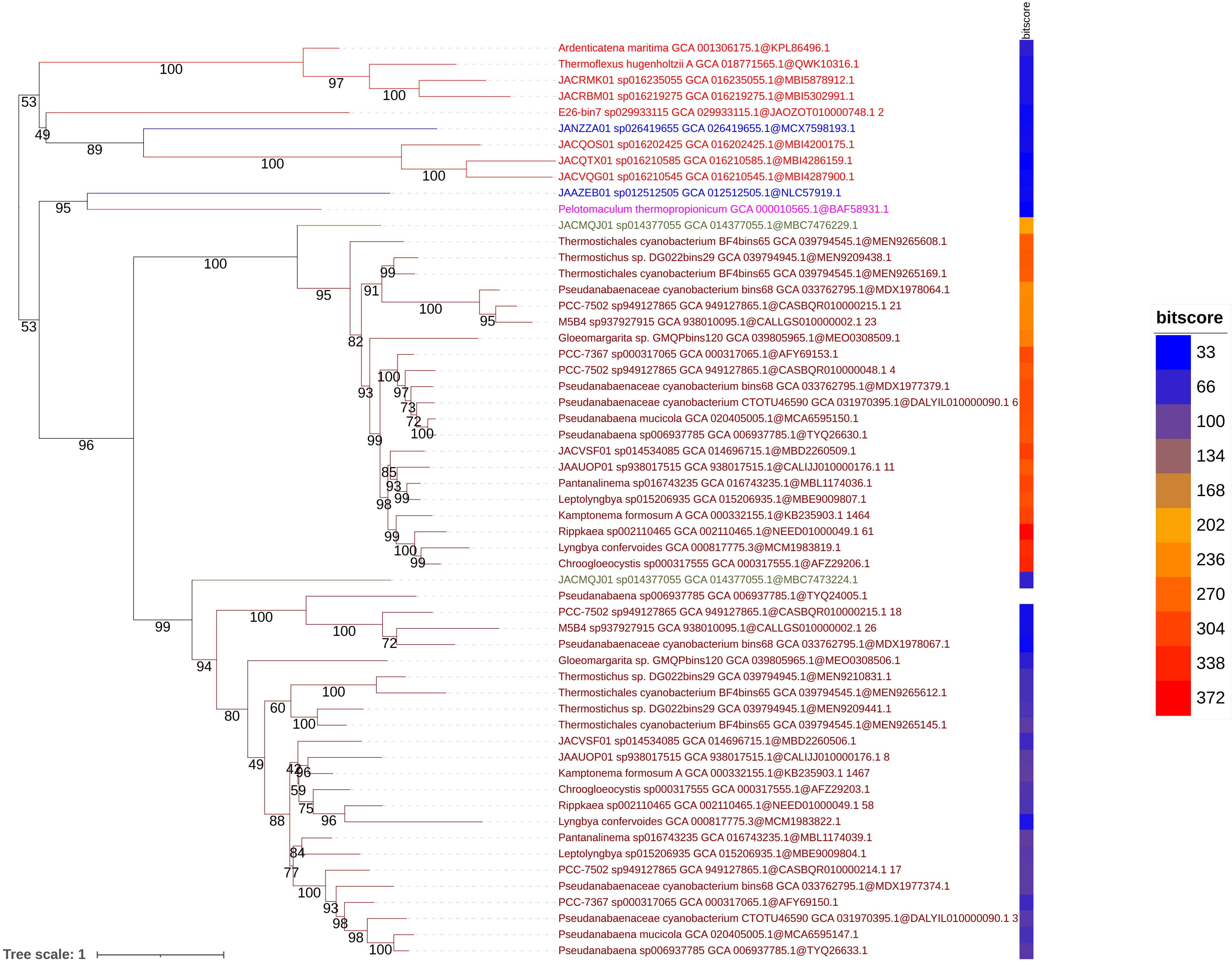

Fig. S78 - Slr0151

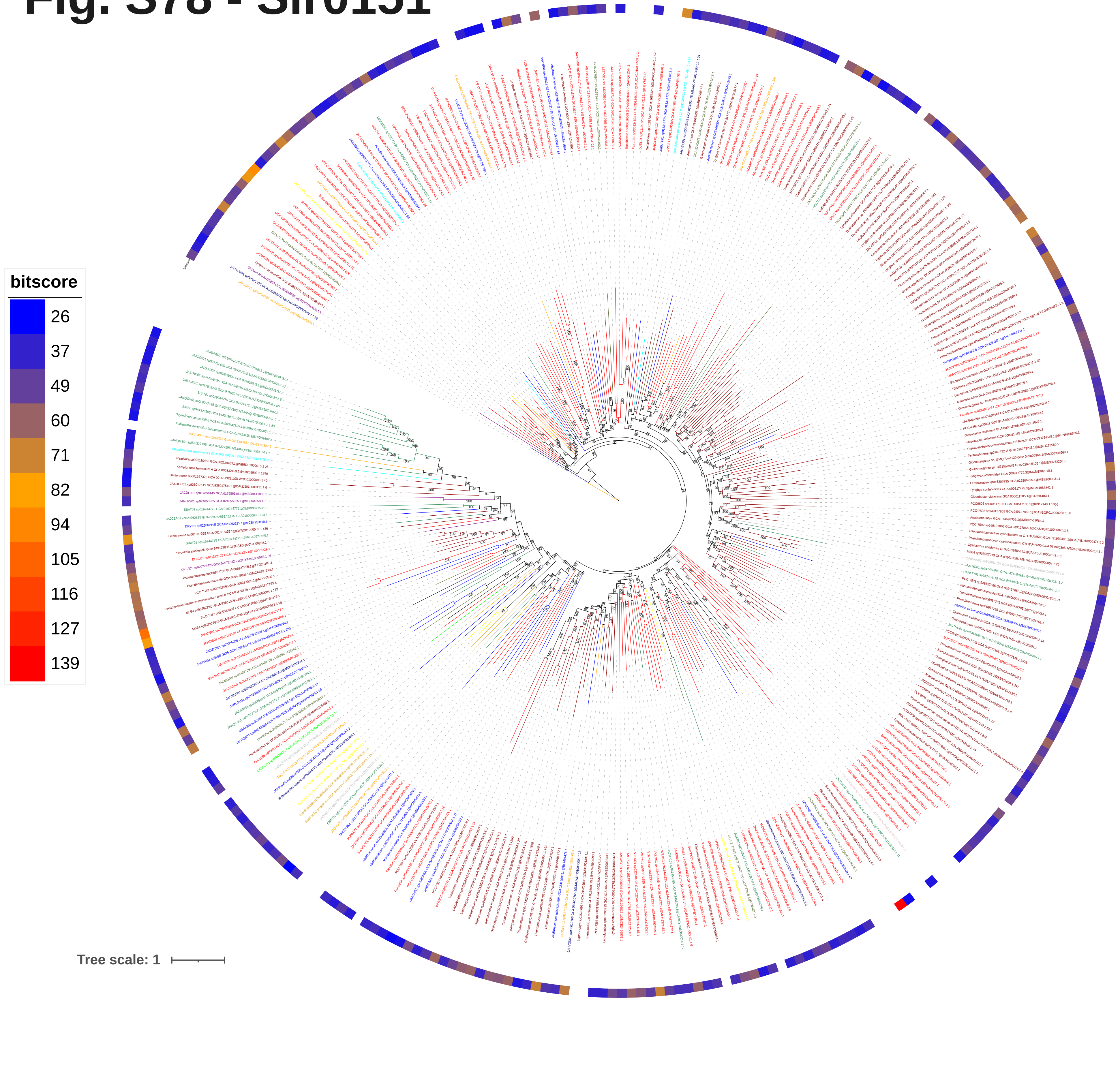

Fig. S79 - Slr0232

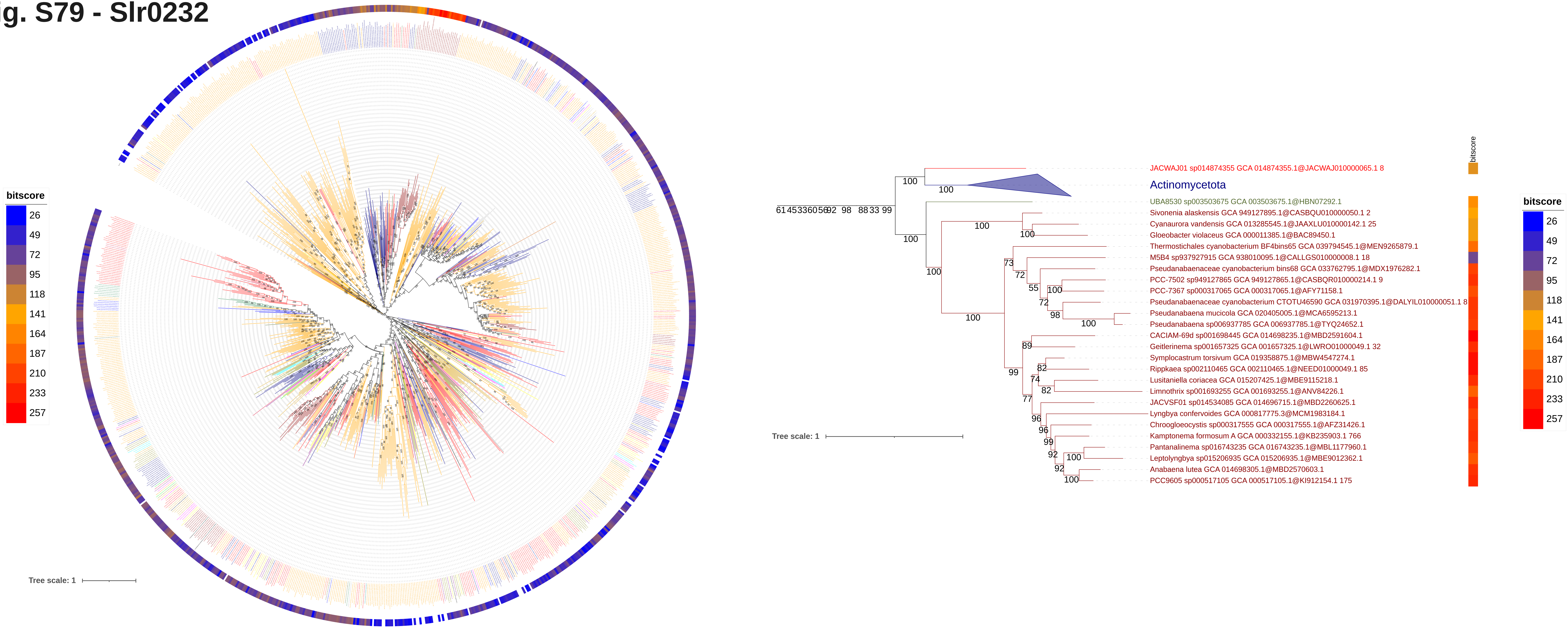

Fig. S80 - Slr0286

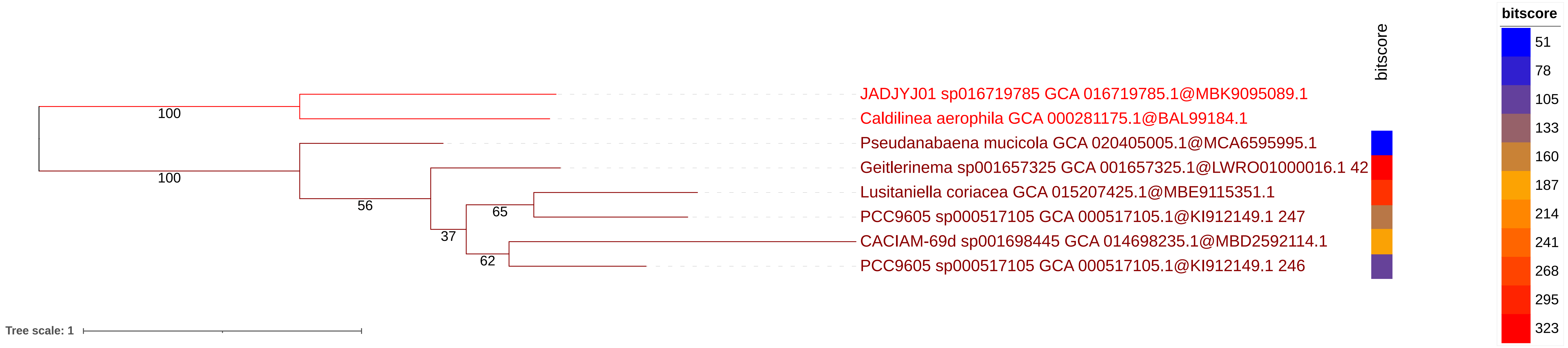

Fig. S81 - Slr0305

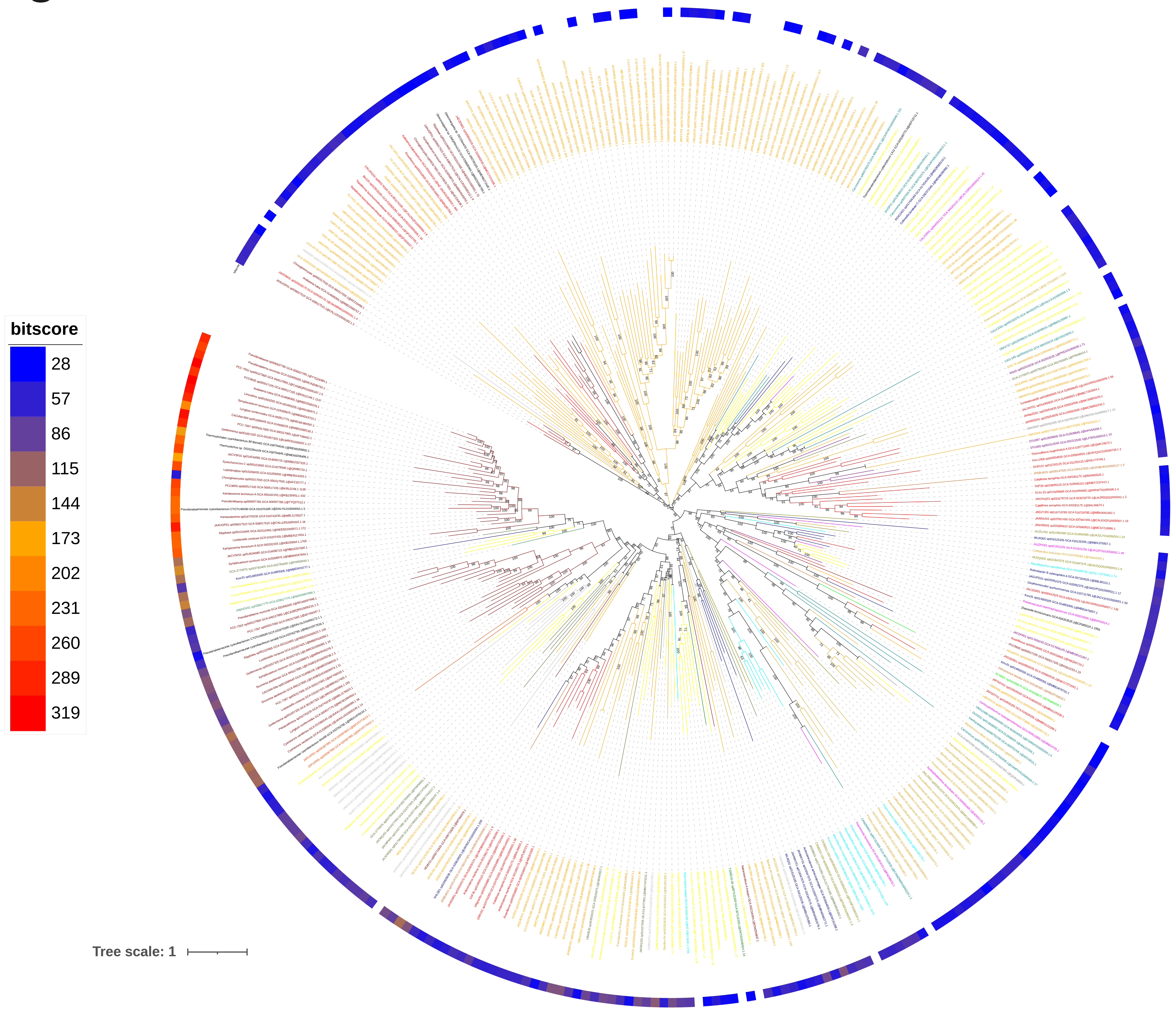

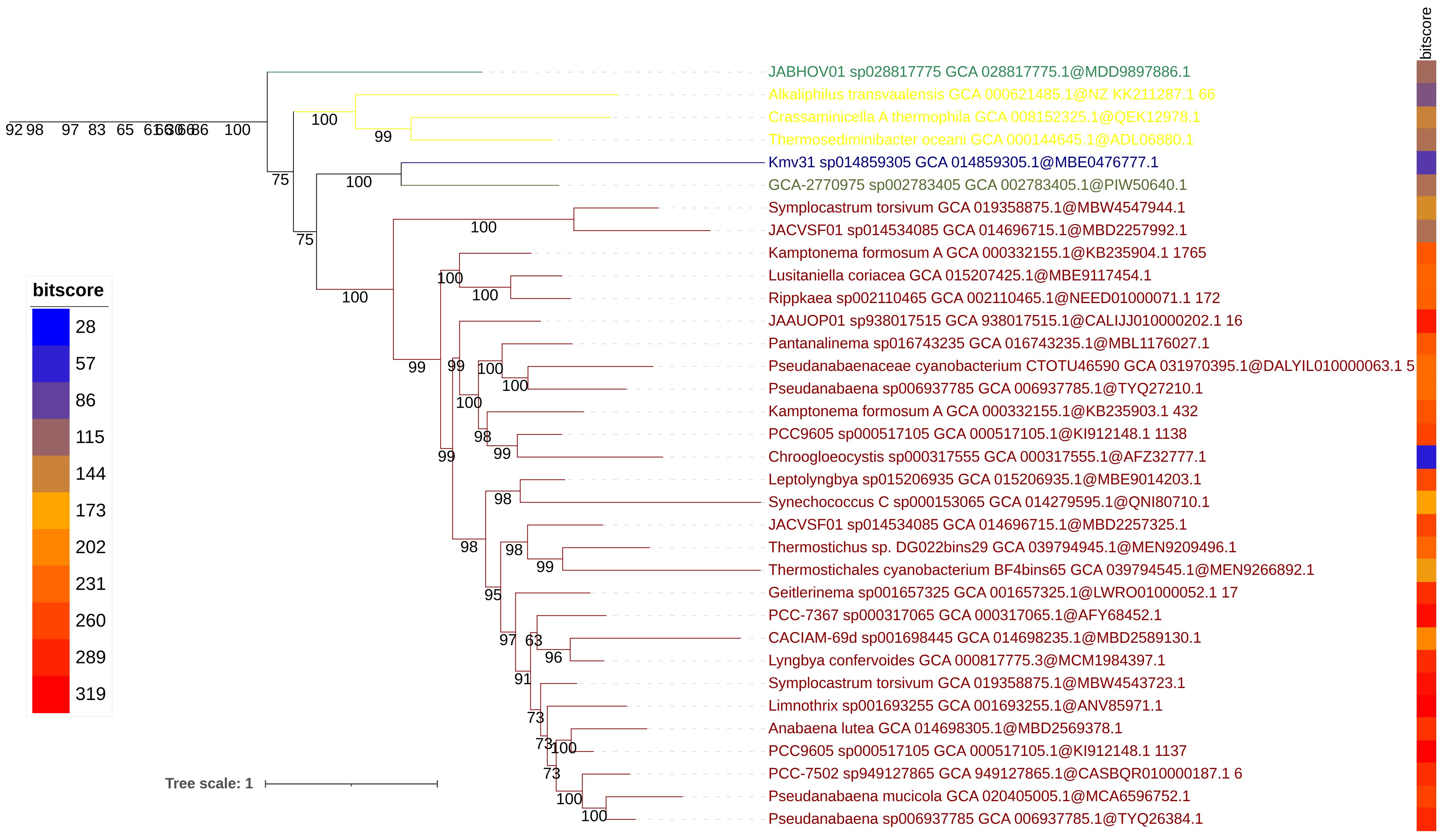

Fig. S82 - Slr0483

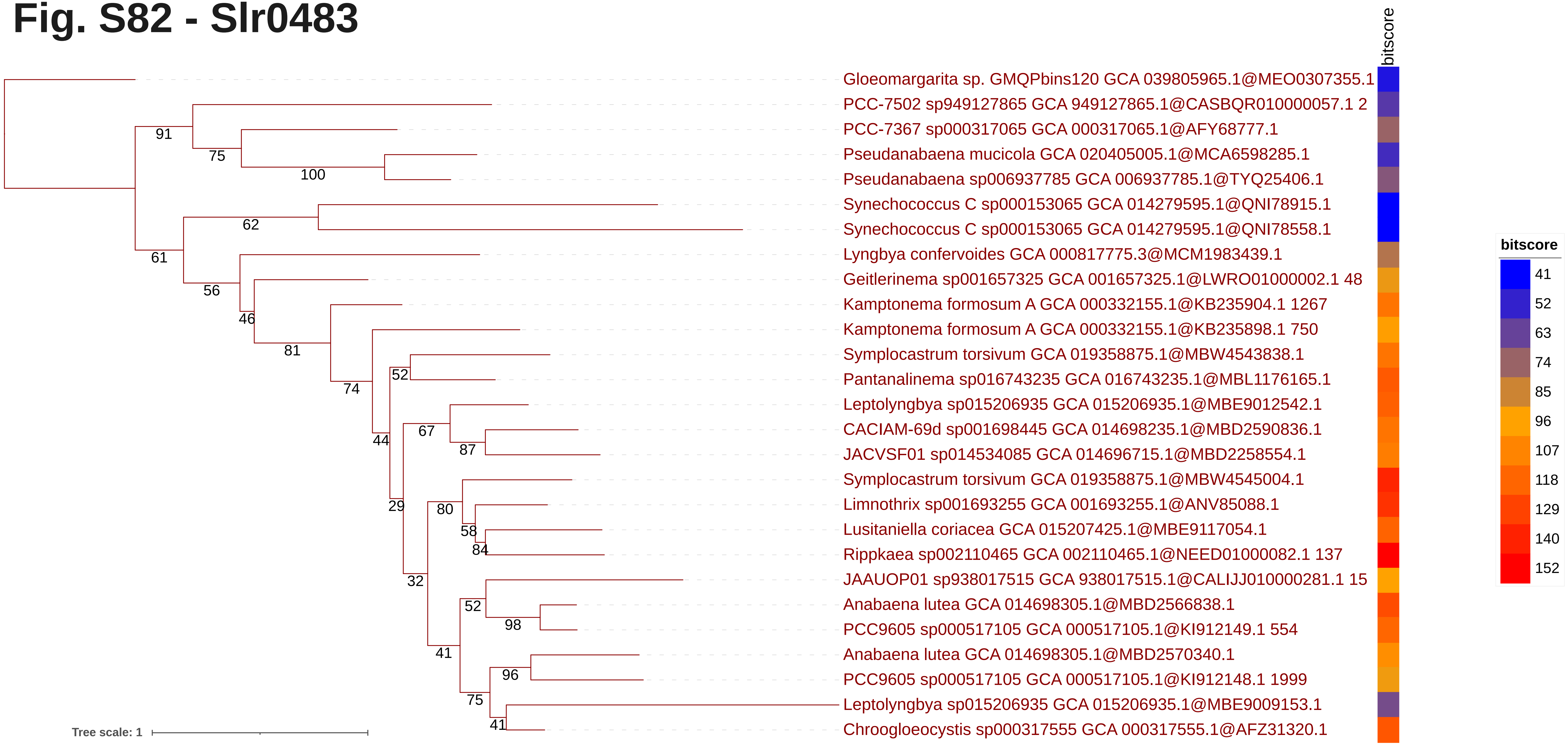

Fig. S83 - Slr0565

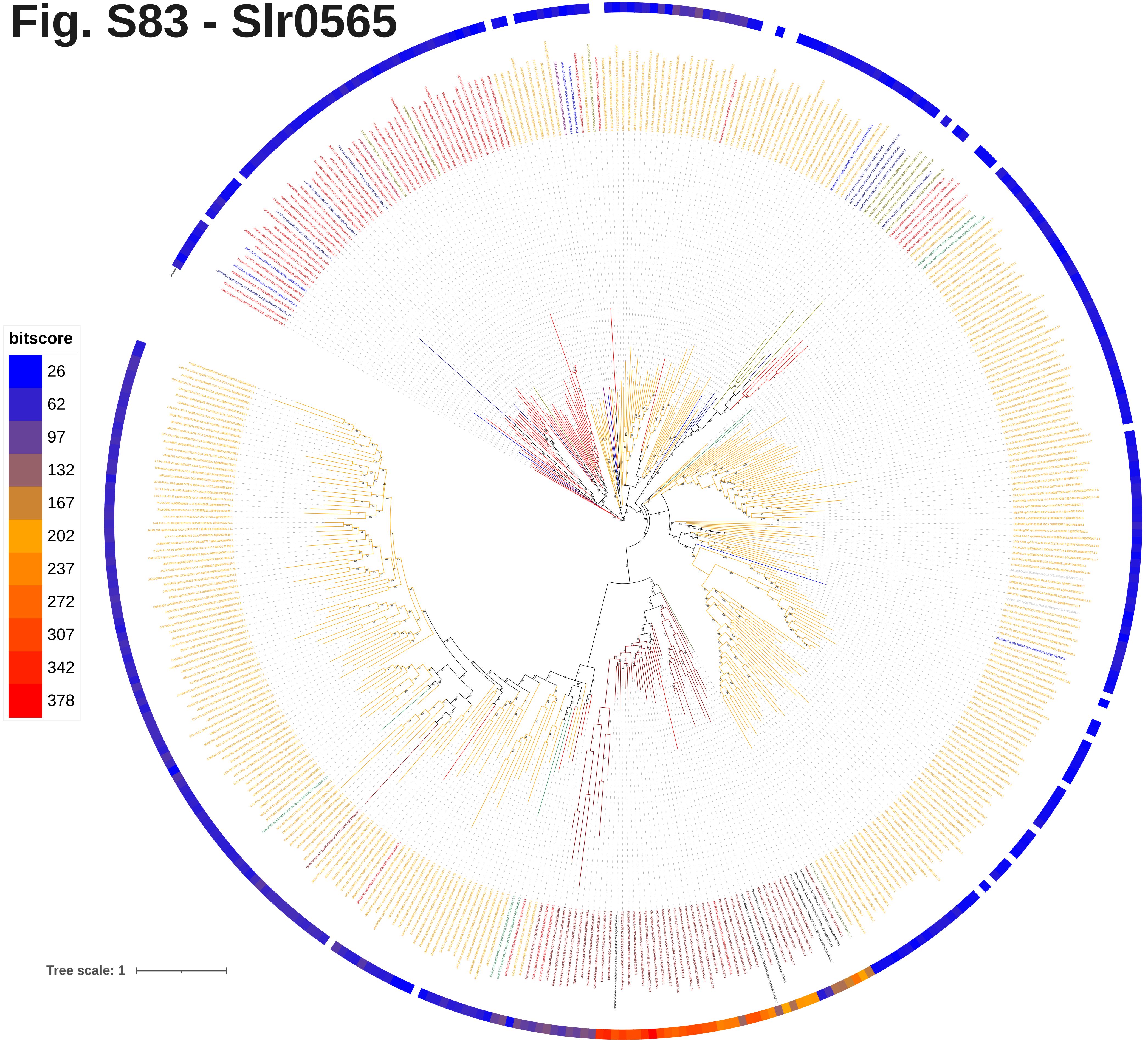

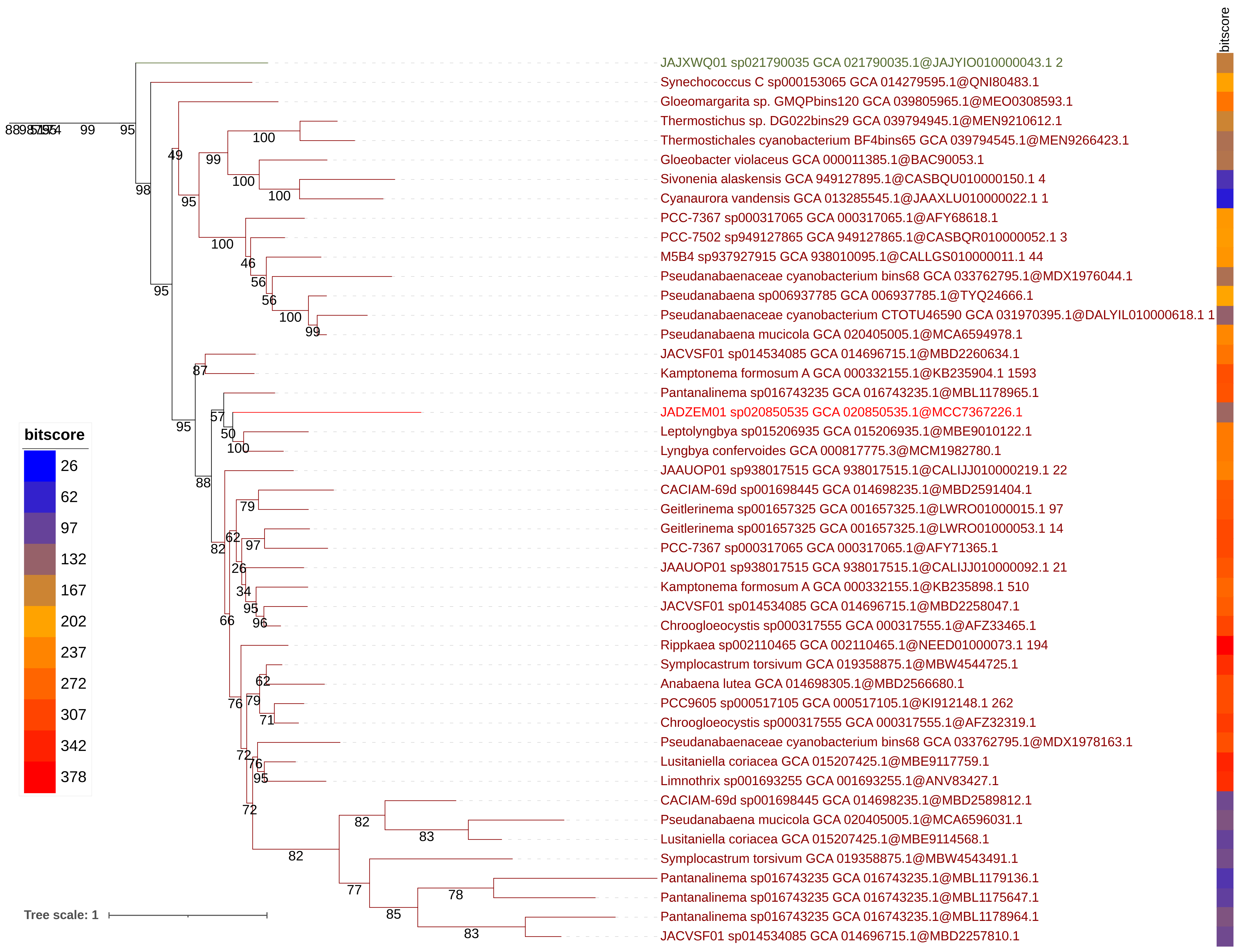

Fig. S84 - Slr0869

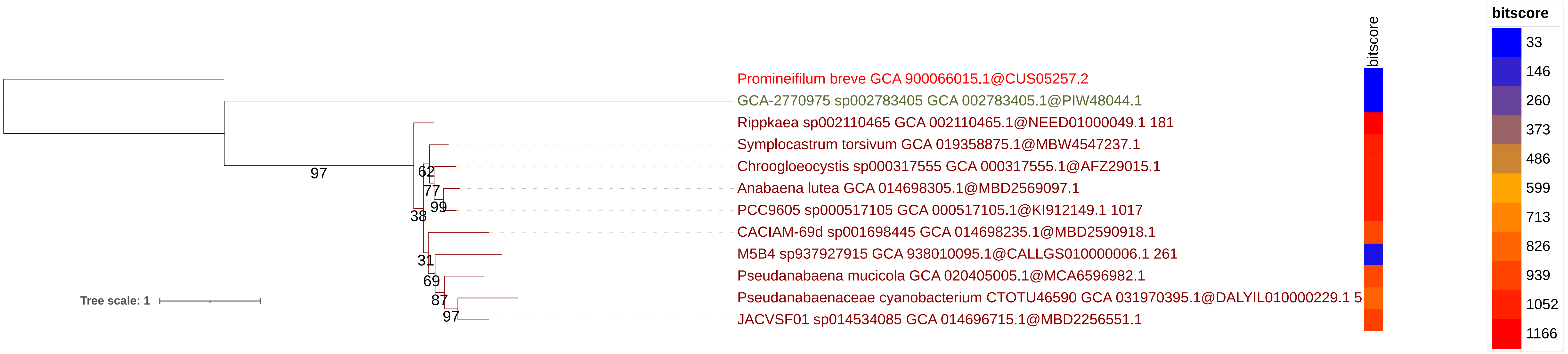

Fig. S85 - Slr1106

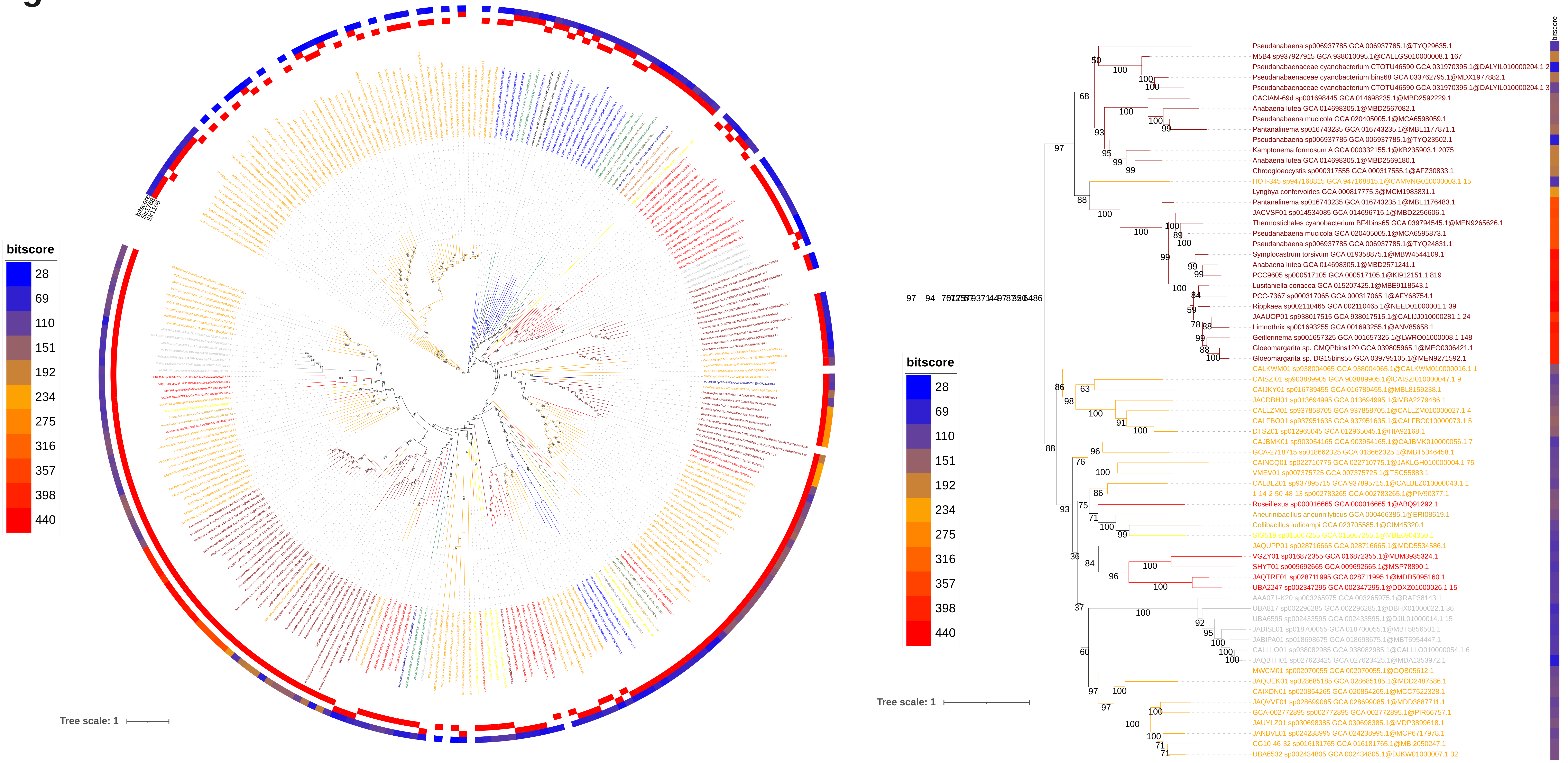

Fig. S86 - Slr1128 / Sll0815

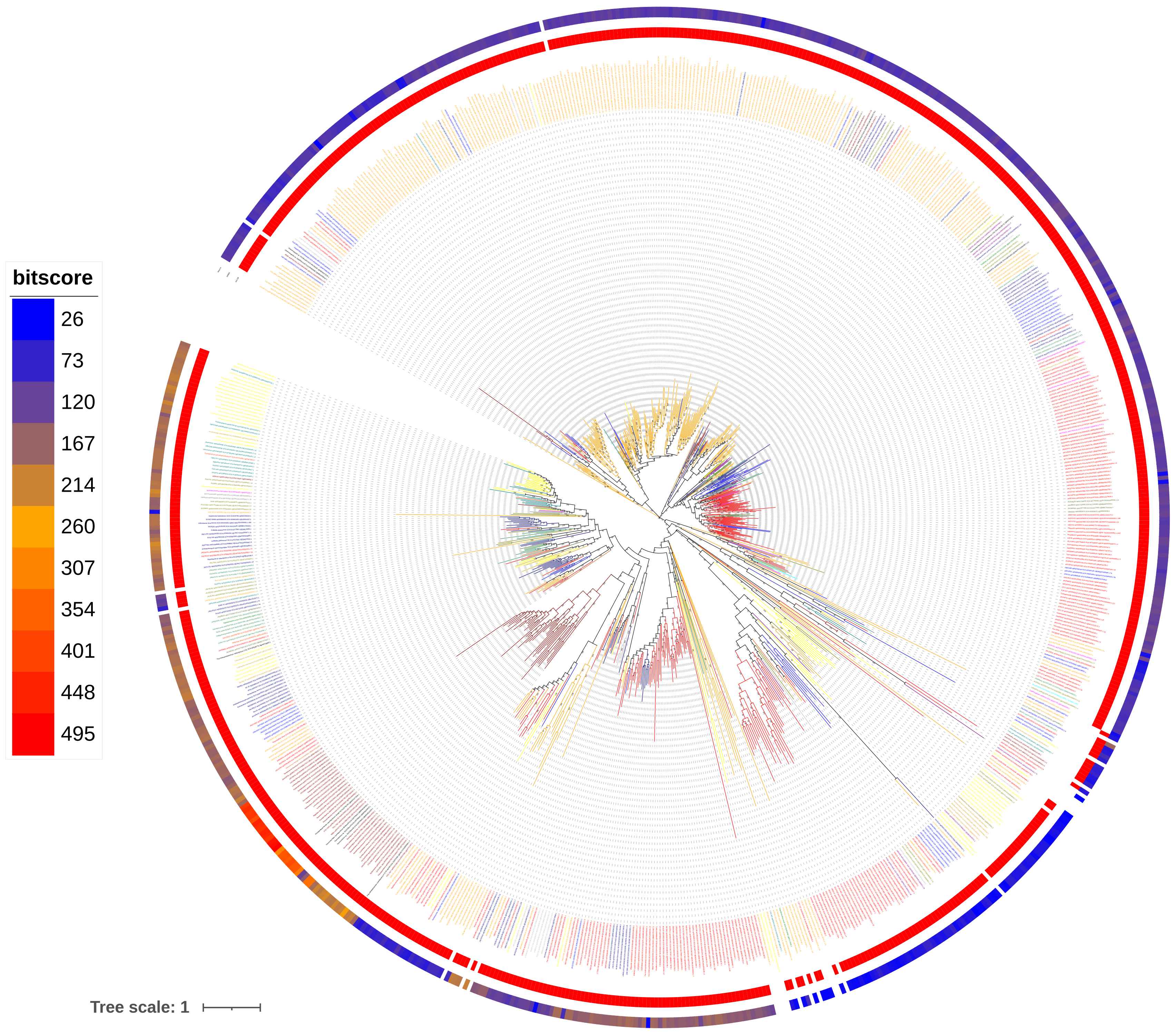

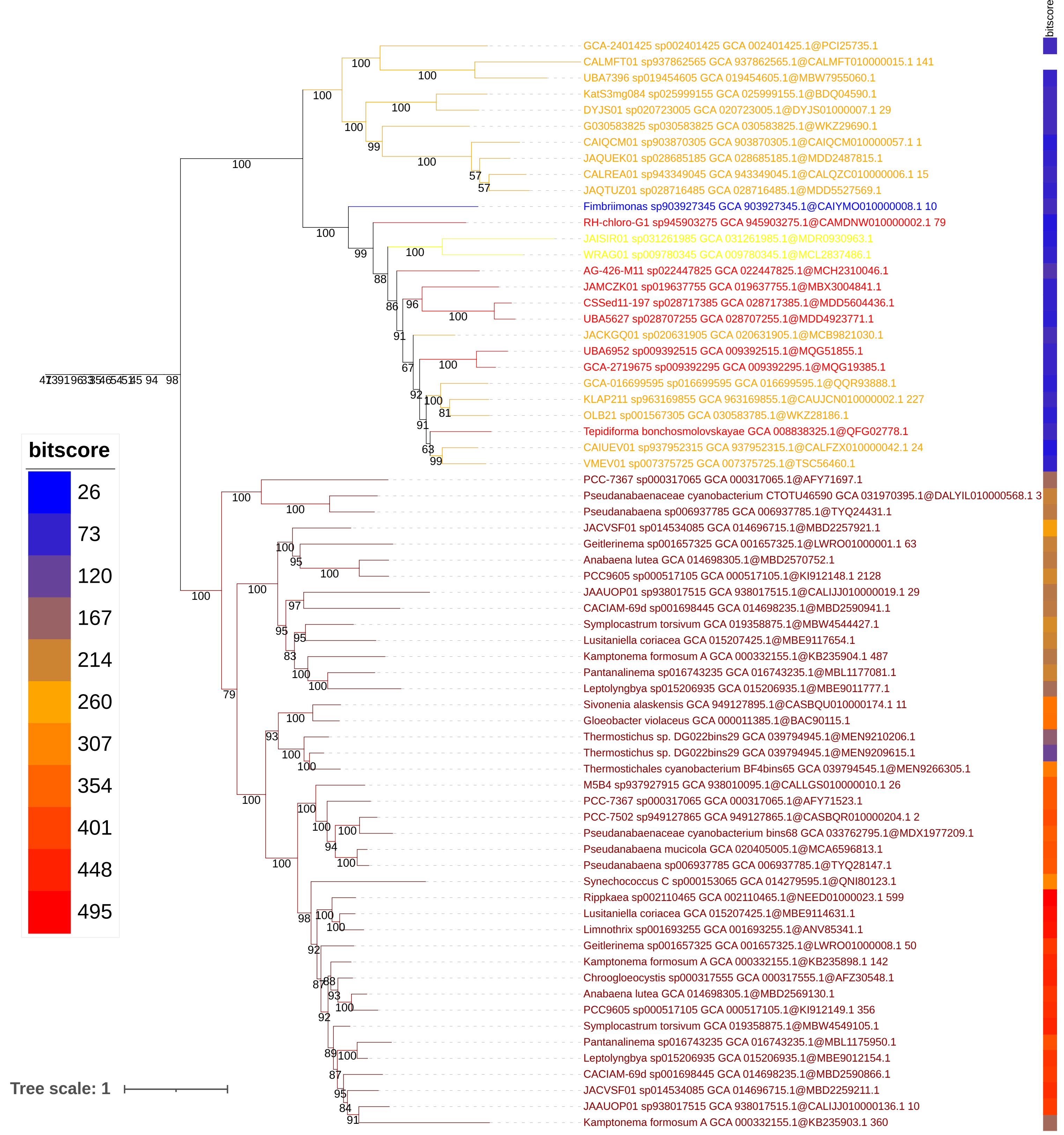

Fig. S87 - Slr1761

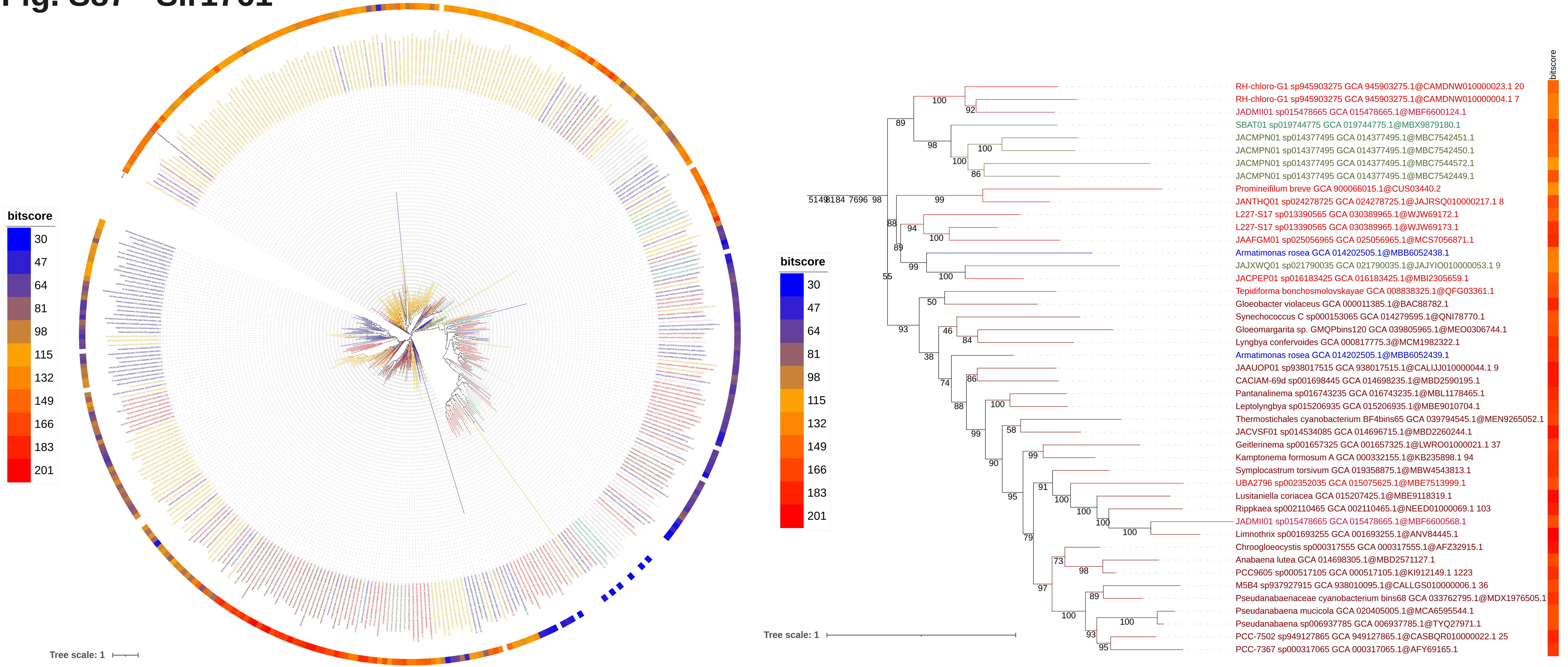

Fig. S88 - Slr1768

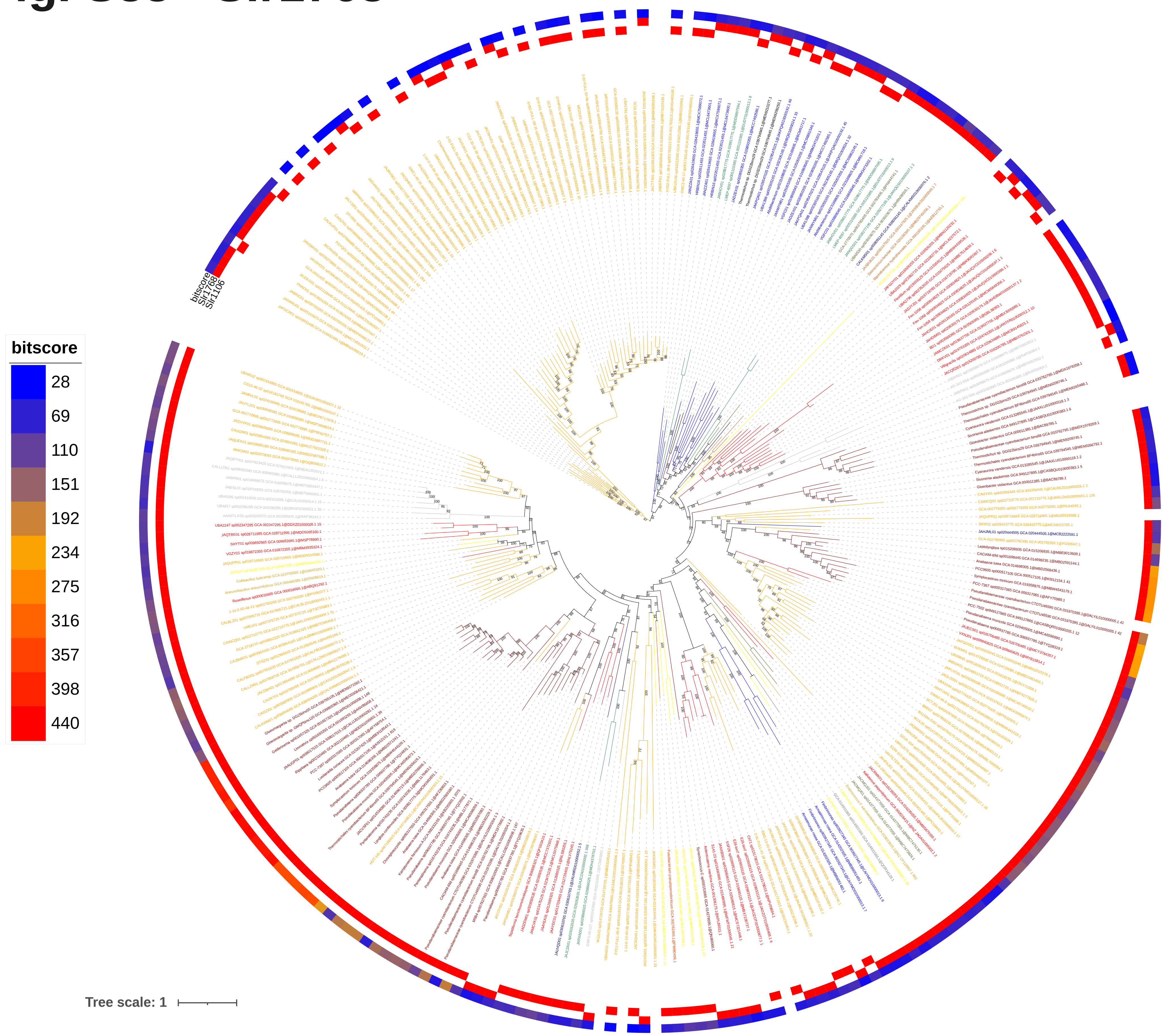

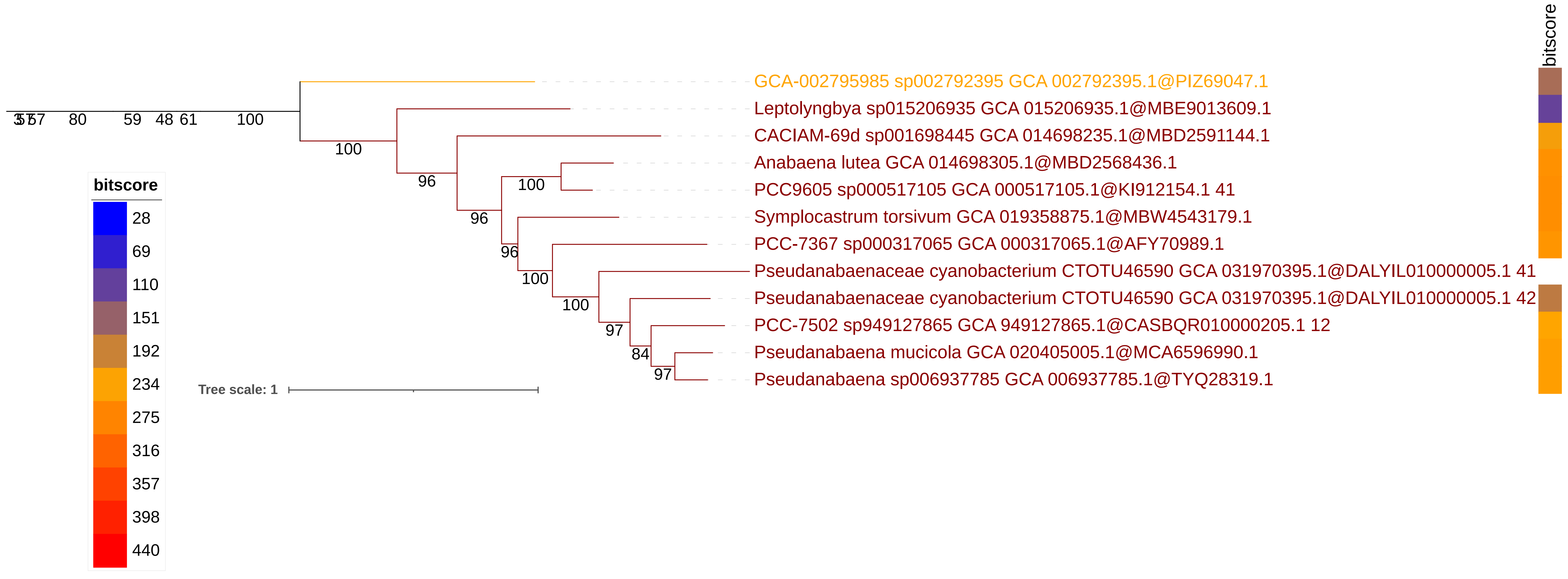

Fig. S89 - Slr2013

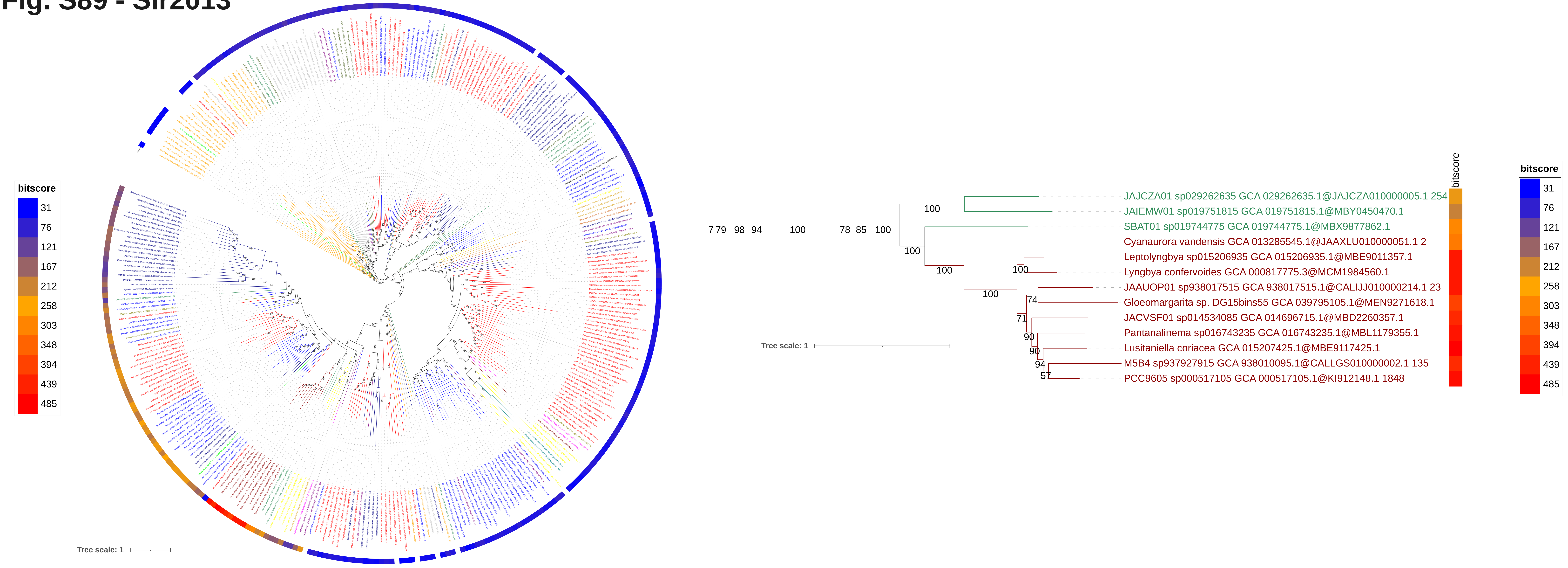

Fig. S90 - IM30 (VIPP1)

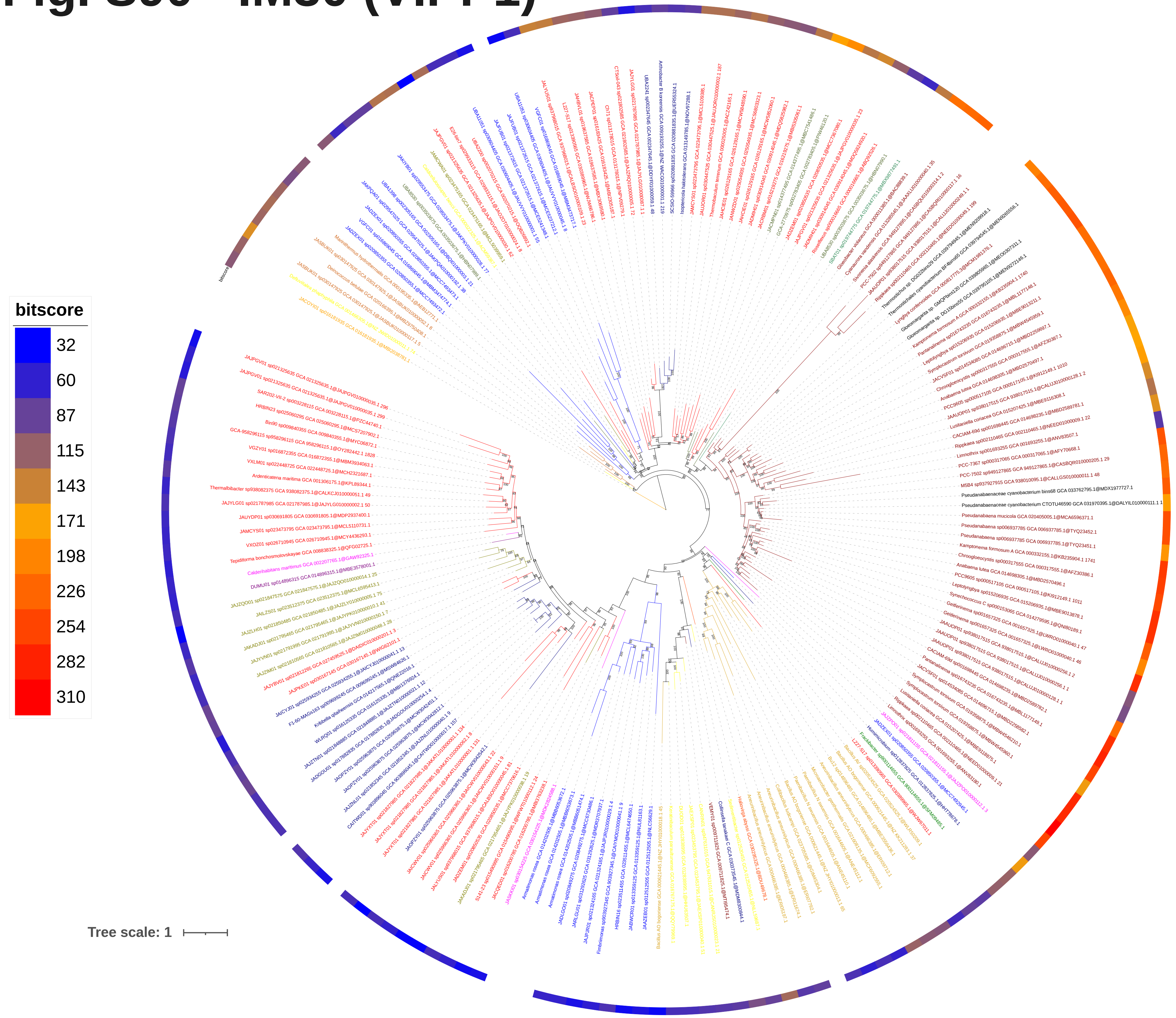

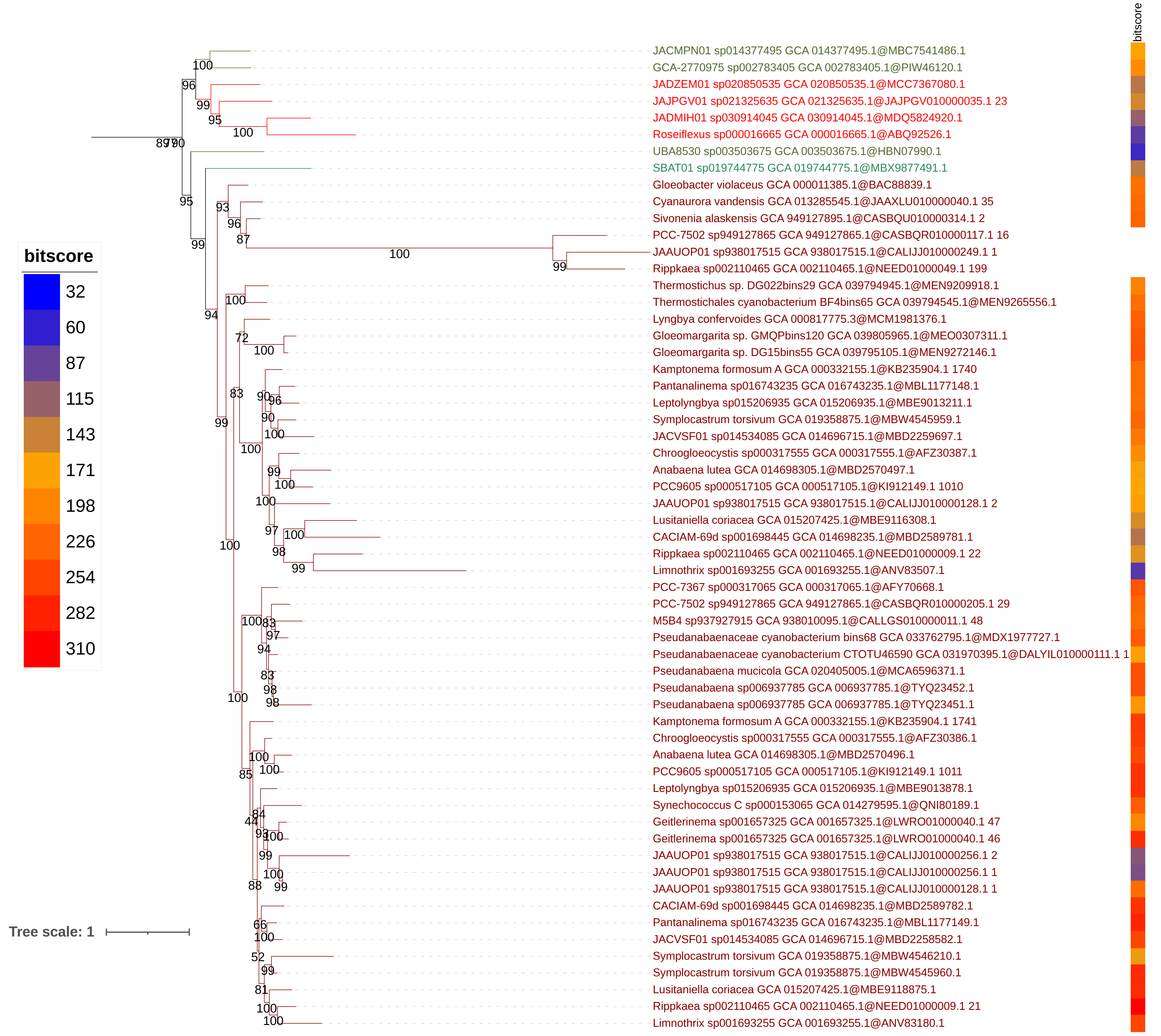

Fig. S91 - Ycf37

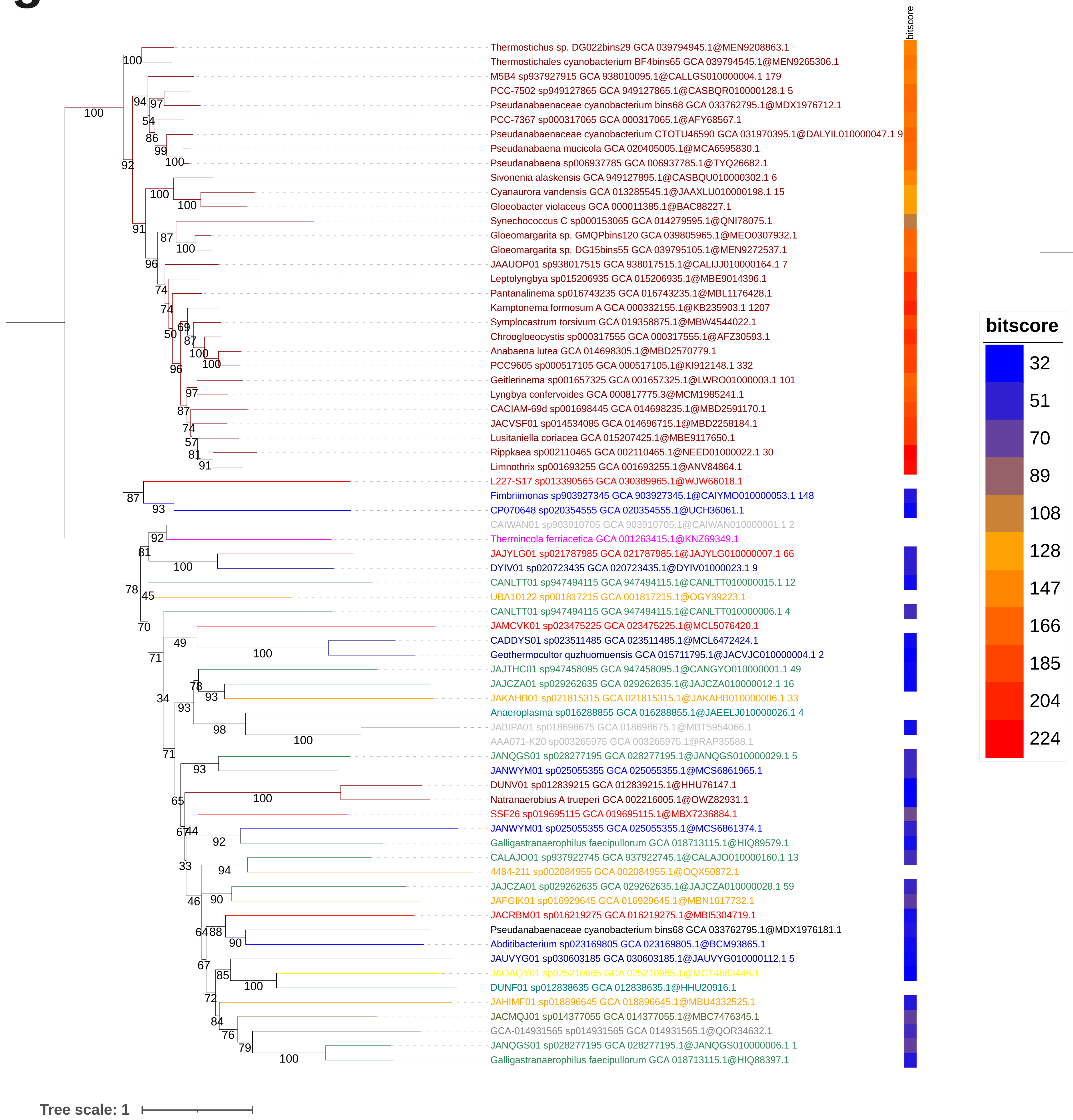

Fig. S92 - Ycf39

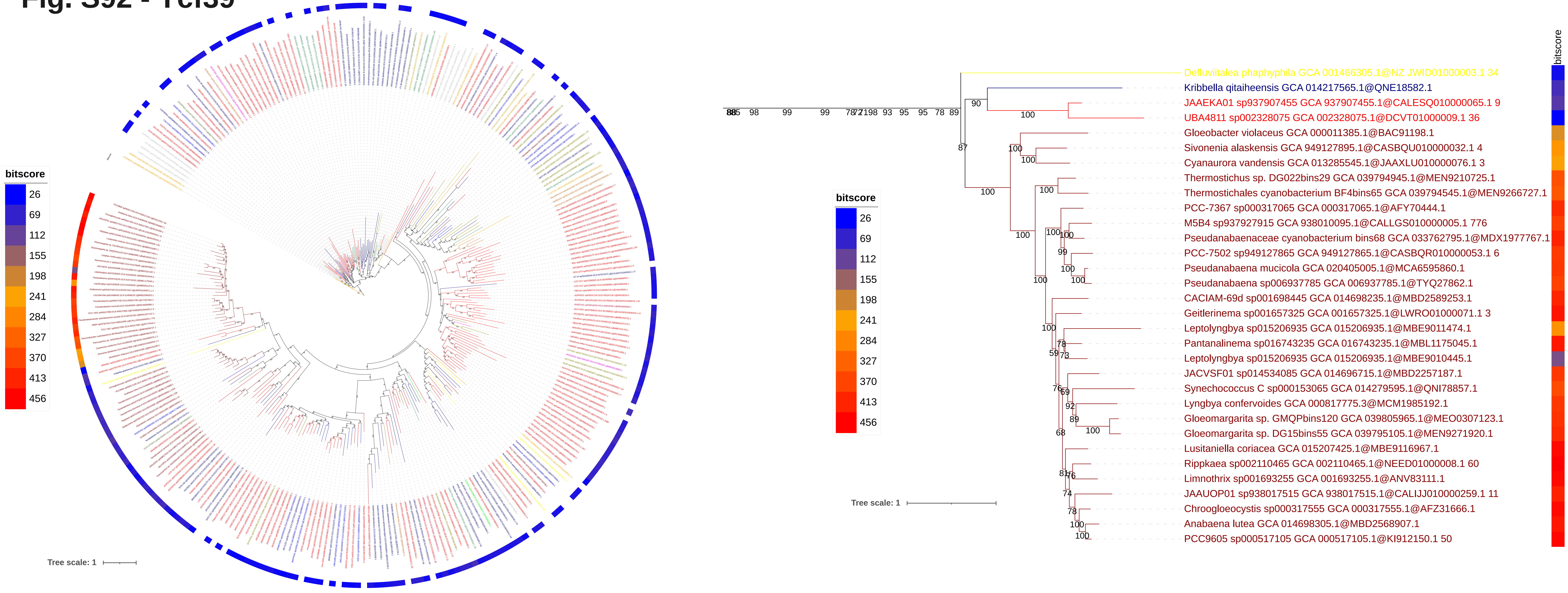

Fig. S93 - Ycf48

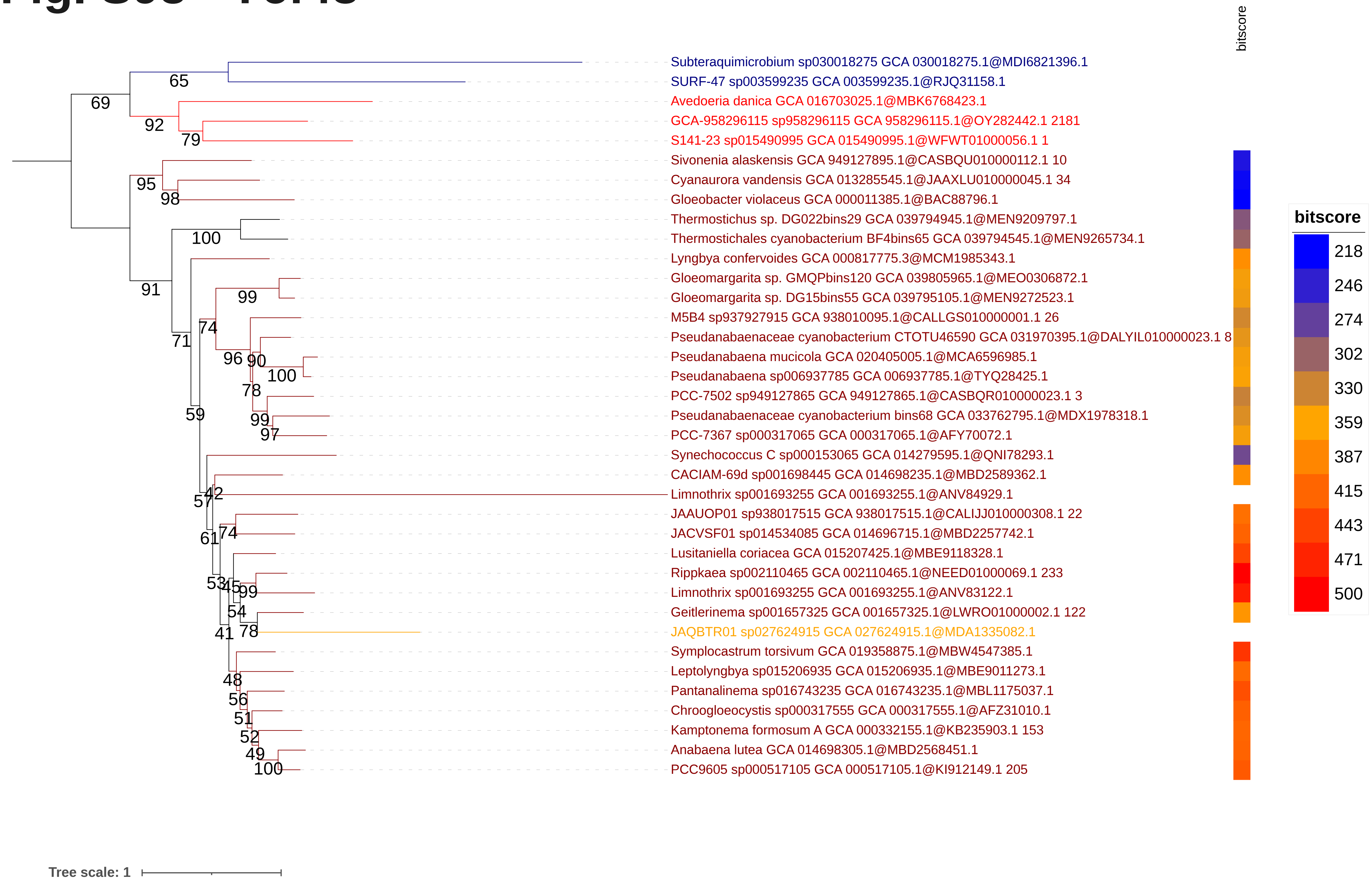

Fig. S94 - YidC

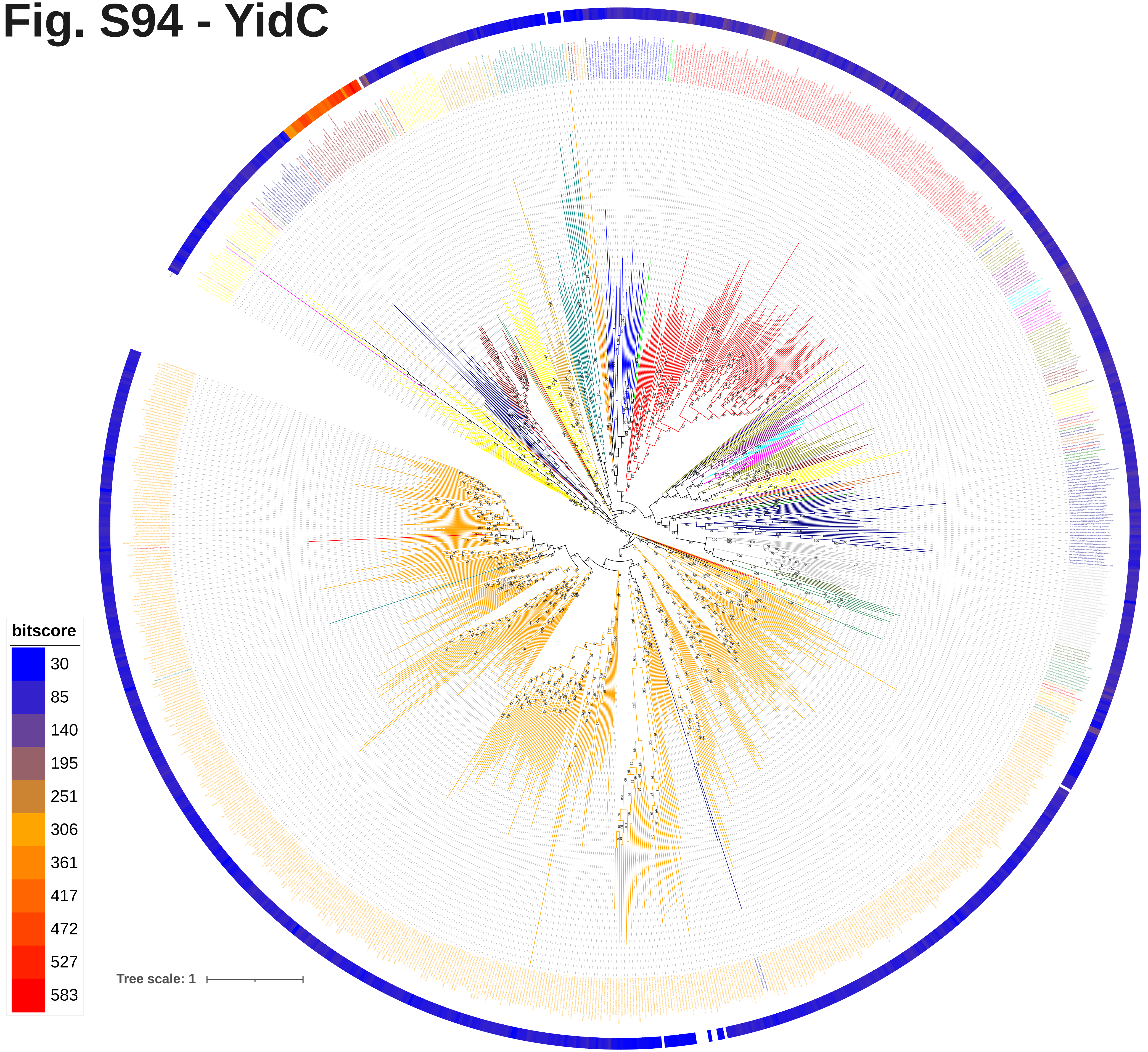
bitscore
30
85
140
195
251
306
361
417
472
527
583
Tree scale: 1

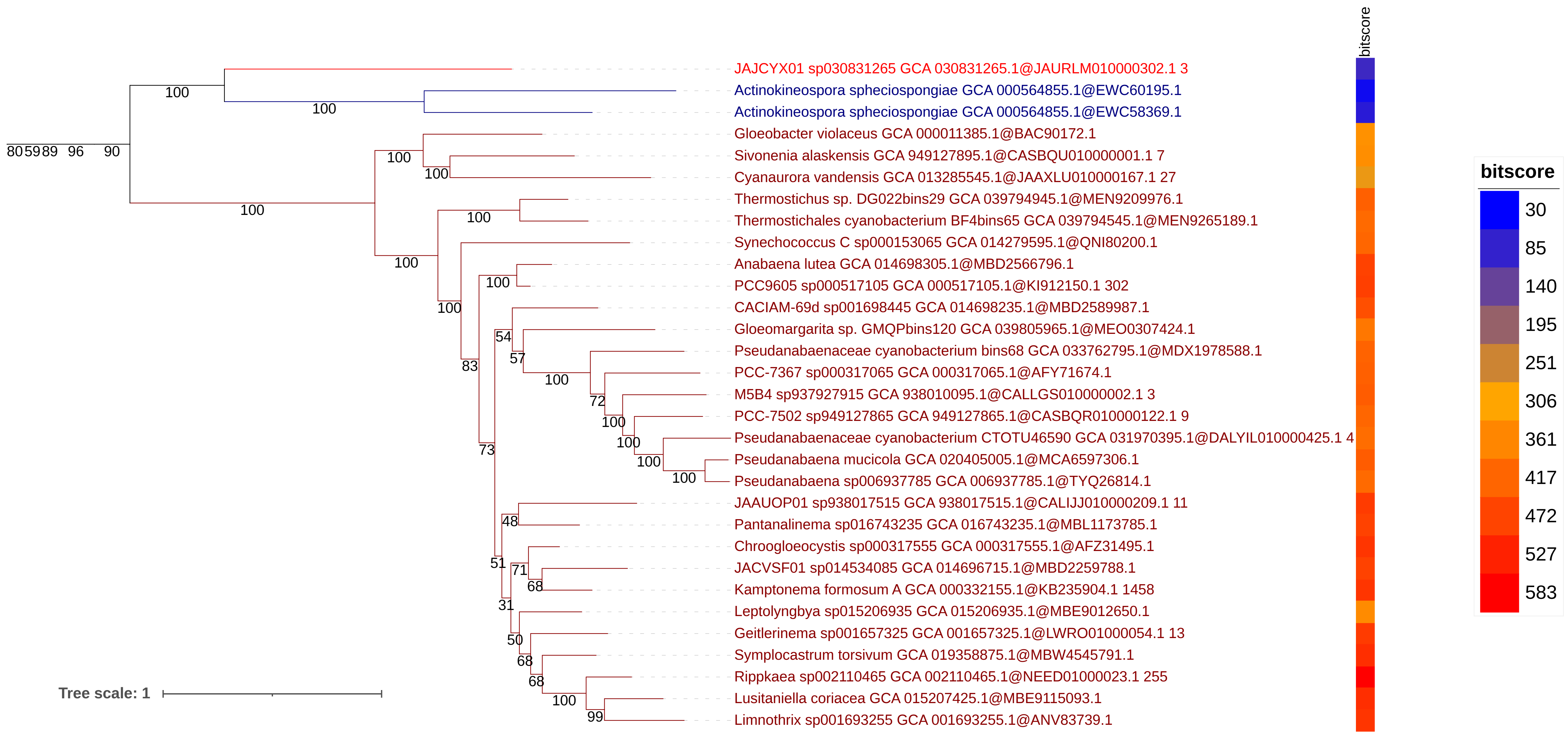
bitscore
JAJCYX01 sp030831265 GCA 030831265.1@JAURLM010000302.1 3
Actinokineospora spheciospongiae GCA 000564855.1@EWC60195.1
Actinokineospora spheciospongiae GCA 000564855.1@EWC58369.1
Gloeobacter violaceus GCA 000011385.1@BAC90172.1
Sivonenia alaskensis GCA 949127895.1@CASBQU010000001.1 7
Cyanaurora vandensis GCA 013285545.1@JAAXLU010000167.1 27
Thermostichus sp. DG022bins29 GCA 039794945.1@MEN9209976.1
Thermostichales cyanobacterium BF4bins65 GCA 039794545.1@MEN9265189.1
Synechococcus C sp000153065 GCA 014279595.1@QNI80200.1
Anabaena lutea GCA 014698305.1@MBD2566796.1
PCC9605 sp000517105 GCA 000517105.1@KI912150.1 302
CACIAM-69d sp001698445 GCA 014698235.1@MBD2589987.1
Gloeomargarita sp. GMQPbins120 GCA 039805965.1@MEO0307424.1
Pseudanabaenaceae cyanobacterium bins68 GCA 033762795.1@MDX1978588.1
PCC-7367 sp000317065 GCA 000317065.1@AFY71674.1
M5B4 sp937927915 GCA 938010095.1@CALLGS010000002.1 3
PCC-7502 sp949127865 GCA 949127865.1@CASBQR010000122.1 9
Pseudanabaenaceae cyanobacterium CTOTU46590 GCA 031970395.1@DALYIL010000425.1 4
Pseudanabaena mucicola GCA 020405005.1@MCA6597306.1
Pseudanabaena sp006937785 GCA 006937785.1@TYQ26814.1
JAAUOP01 sp938017515 GCA 938017515.1@CALIJJ010000209.1 11
Pantanalinema sp016743235 GCA 016743235.1@MBL1173785.1
Chroogloeocystis sp000317555 GCA 000317555.1@AFZ31495.1
JACVSF01 sp014534085 GCA 014696715.1@MBD2259788.1
Kamptonema formosum A GCA 000332155.1@KB235904.1 1458
Leptolyngbya sp015206935 GCA 015206935.1@MBE9012650.1
Geitlerinema sp001657325 GCA 001657325.1@LWRO01000054.1 13
Symplocastrum torsivum GCA 019358875.1@MBW4545791.1
Rippkaea sp002110465 GCA 002110465.1@NEED01000023.1 255
Lusitaniella coriacea GCA 015207425.1@MBE9115093.1
Limnothrix sp001693255 GCA 001693255.1@ANV83739.1
Tree scale: 1
bitscore
30
85
140
195
251
306
361
417
472
527
583

Fig. S95 - SecA

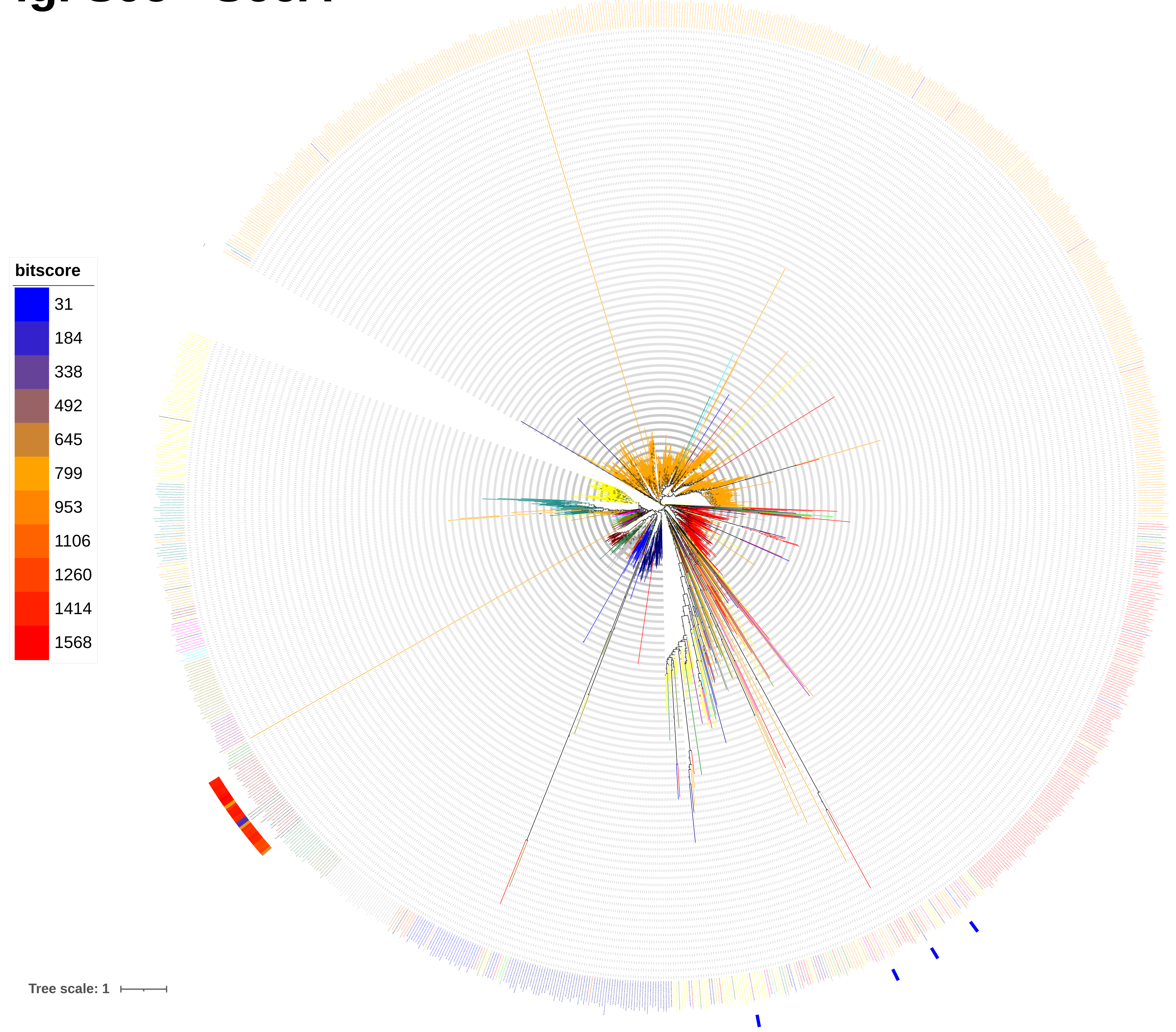
bitscore
31
184
338
492
645
799
953
1106
1260
1414
1568
Tree scale: 1

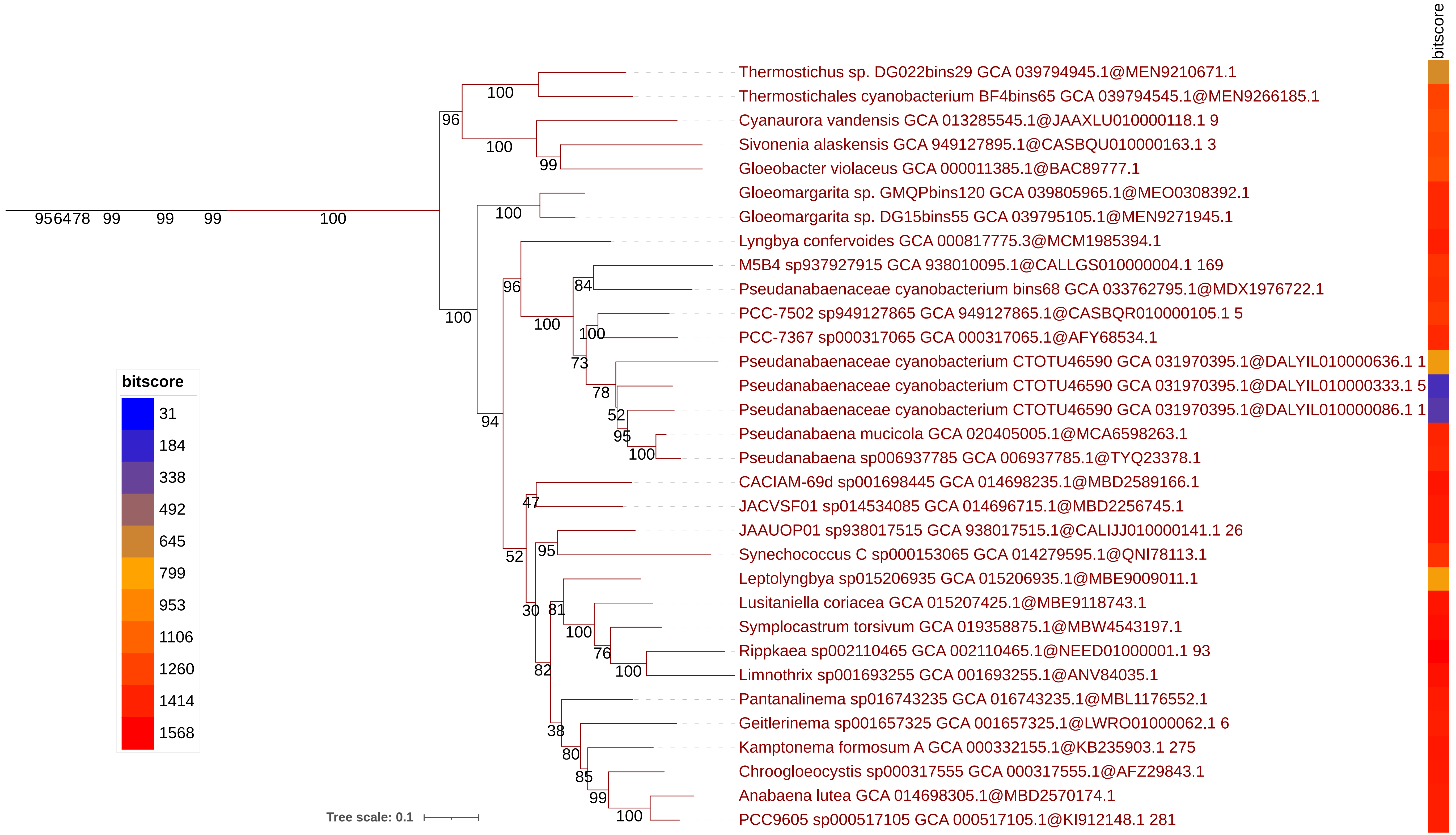
bitscore
31
184
338
492
645
799
953
1106
1260
1414
1568
Tree scale: 0.1
Thermostichus sp. DG022bins29 GCA 039794945.1@MEN9210671.1
Thermostichales cyanobacterium BF4bins65 GCA 039794545.1@MEN9266185.1
Cyanaurora vandensis GCA 013285545.1@JAAXLU010000118.1 9
Sivonenia alaskensis GCA 949127895.1@CASBQU010000163.1 3
Gloeobacter violaceus GCA 000011385.1@BAC89777.1
Gloeomargarita sp. GMQPbins120 GCA 039805965.1@MEO0308392.1
Gloeomargarita sp. DG15bins55 GCA 039795105.1@MEN9271945.1
Lyngbya confervoides GCA 000817775.3@MCM1985394.1
M5B4 sp937927915 GCA 938010095.1@CALLGS010000004.1 169
Pseudanabaenaceae cyanobacterium bins68 GCA 033762795.1@MDX1976722.1
PCC-7502 sp949127865 GCA 949127865.1@CASBQR010000105.1 5
PCC-7367 sp000317065 GCA 000317065.1@AFY68534.1
Pseudanabaenaceae cyanobacterium CTOTU46590 GCA 031970395.1@DALYIL010000636.1 1
Pseudanabaenaceae cyanobacterium CTOTU46590 GCA 031970395.1@DALYIL010000333.1 5
Pseudanabaenaceae cyanobacterium CTOTU46590 GCA 031970395.1@DALYIL010000086.1 1
Pseudanabaena mucicola GCA 020405005.1@MCA6598263.1
Pseudanabaena sp006937785 GCA 006937785.1@TYQ23378.1
CACIAM-69d sp001698445 GCA 014698235.1@MBD2589166.1
JACVSF01 sp014534085 GCA 014696715.1@MBD2256745.1
JAAUOP01 sp938017515 GCA 938017515.1@CALIJJ010000141.1 26
Synechococcus C sp000153065 GCA 014279595.1@QNI78113.1
Leptolyngbya sp015206935 GCA 015206935.1@MBE9009011.1
Lusitaniella coriacea GCA 015207425.1@MBE9118743.1
Symplocastrum torsivum GCA 019358875.1@MBW4543197.1
Rippkaea sp002110465 GCA 002110465.1@NEED01000001.1 93
Limnothrix sp001693255 GCA 001693255.1@ANV84035.1
Pantanalinema sp016743235 GCA 016743235.1@MBL1176552.1
Geitlerinema sp001657325 GCA 001657325.1@LWRO01000062.1 6
Kamptonema formosum A GCA 000332155.1@KB235903.1 275
Chroogloeocystis sp000317555 GCA 000317555.1@AFZ29843.1
Anabaena lutea GCA 014698305.1@MBD2570174.1
PCC9605 sp000517105 GCA 000517105.1@KI912148.1 281

Fig. S96 - ChlG

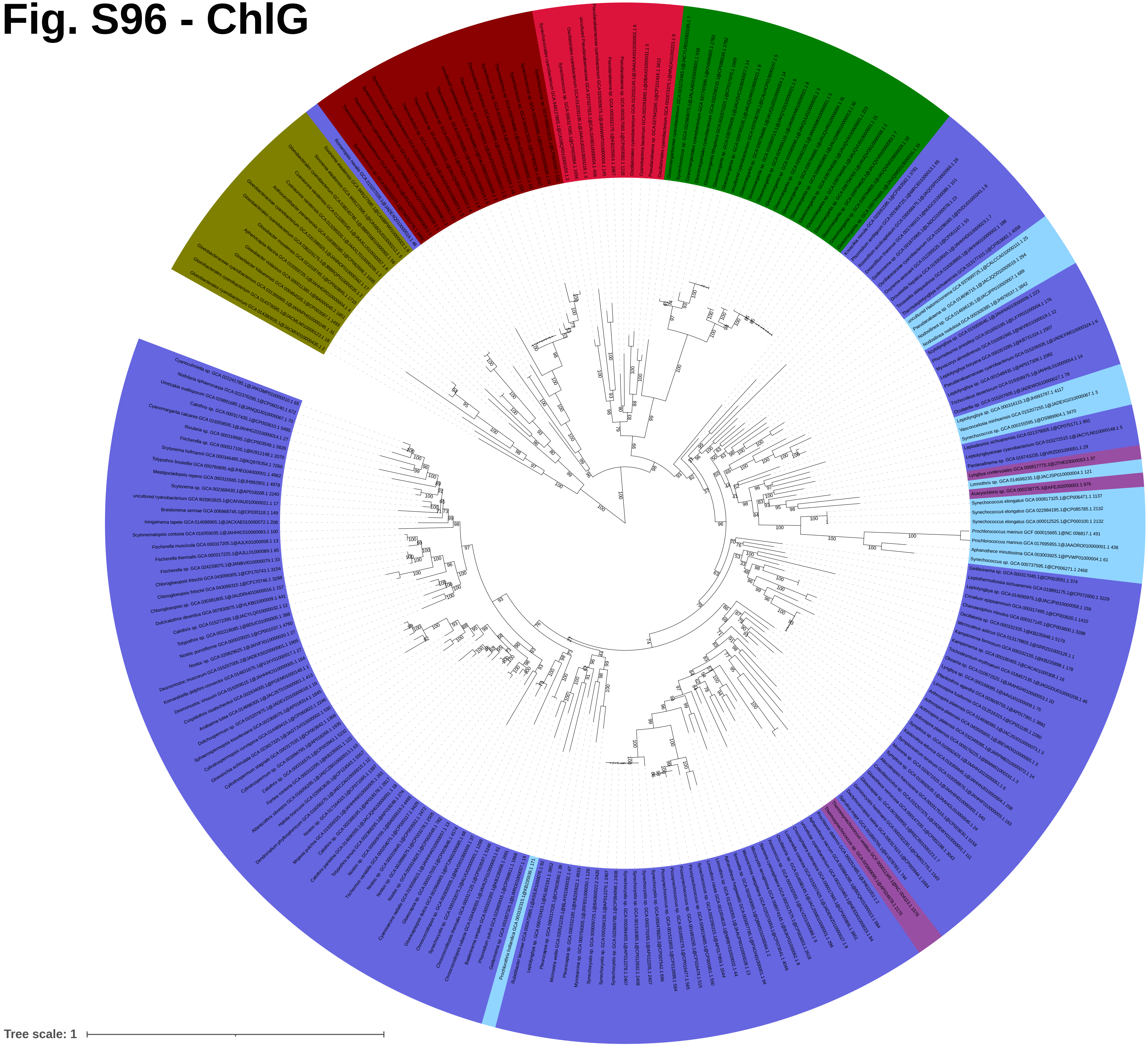

Fig. S97 - CtpA

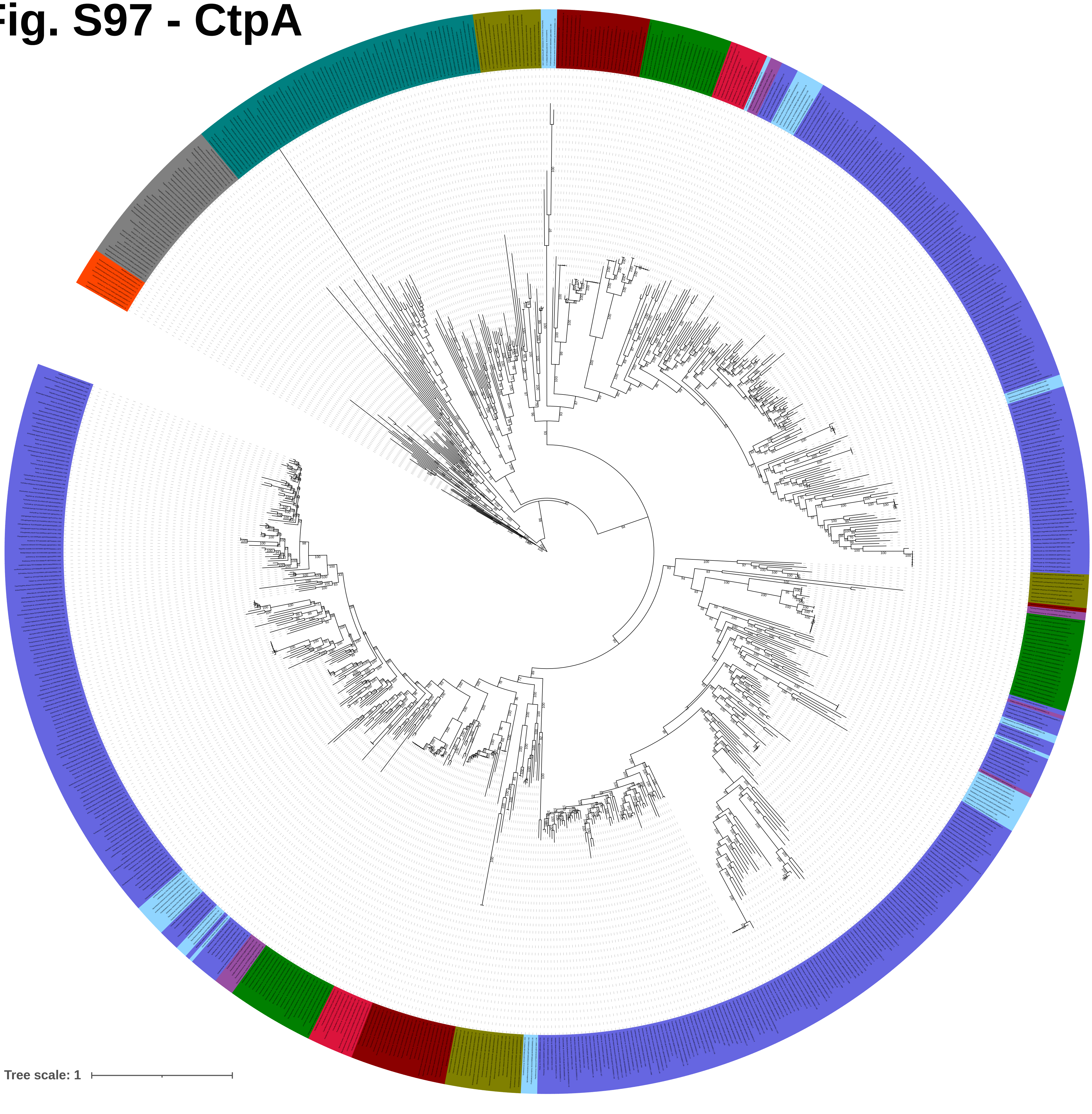

Fig. S98 - CyanoP (PsbP-like)

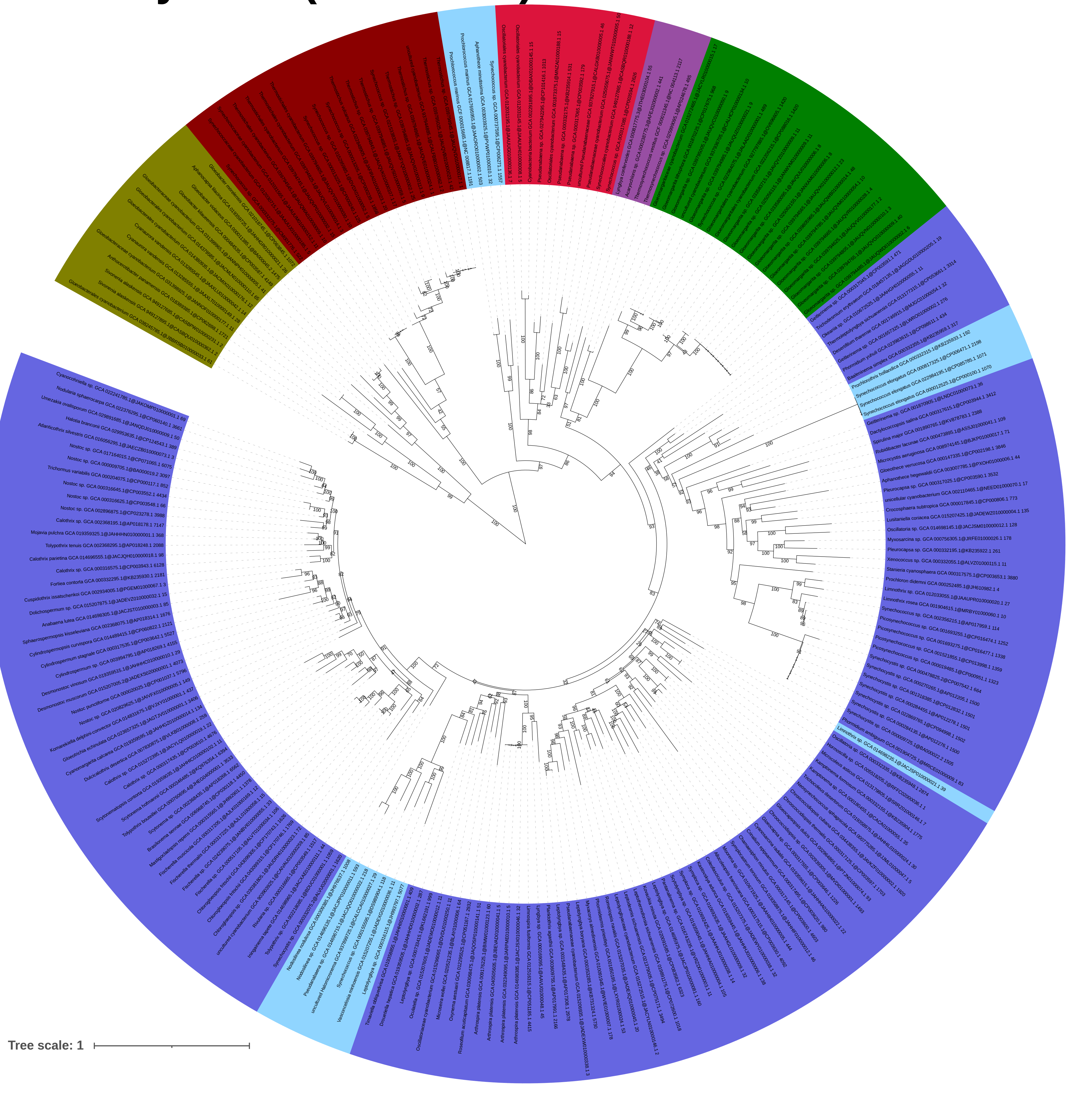

Fig. S99 - FtsZ

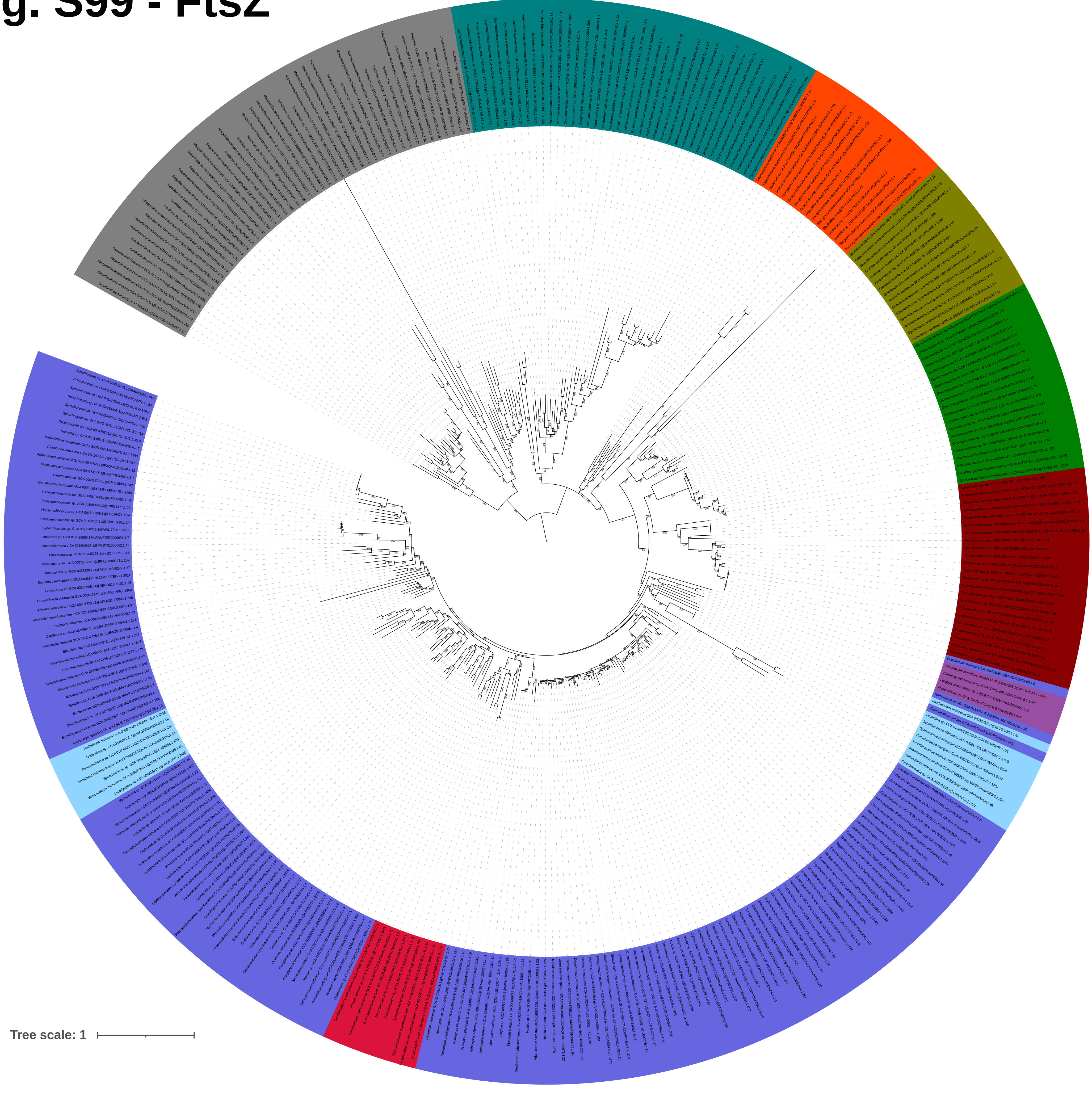

Fig. S100 - IM30 (VIPP1) / PspA

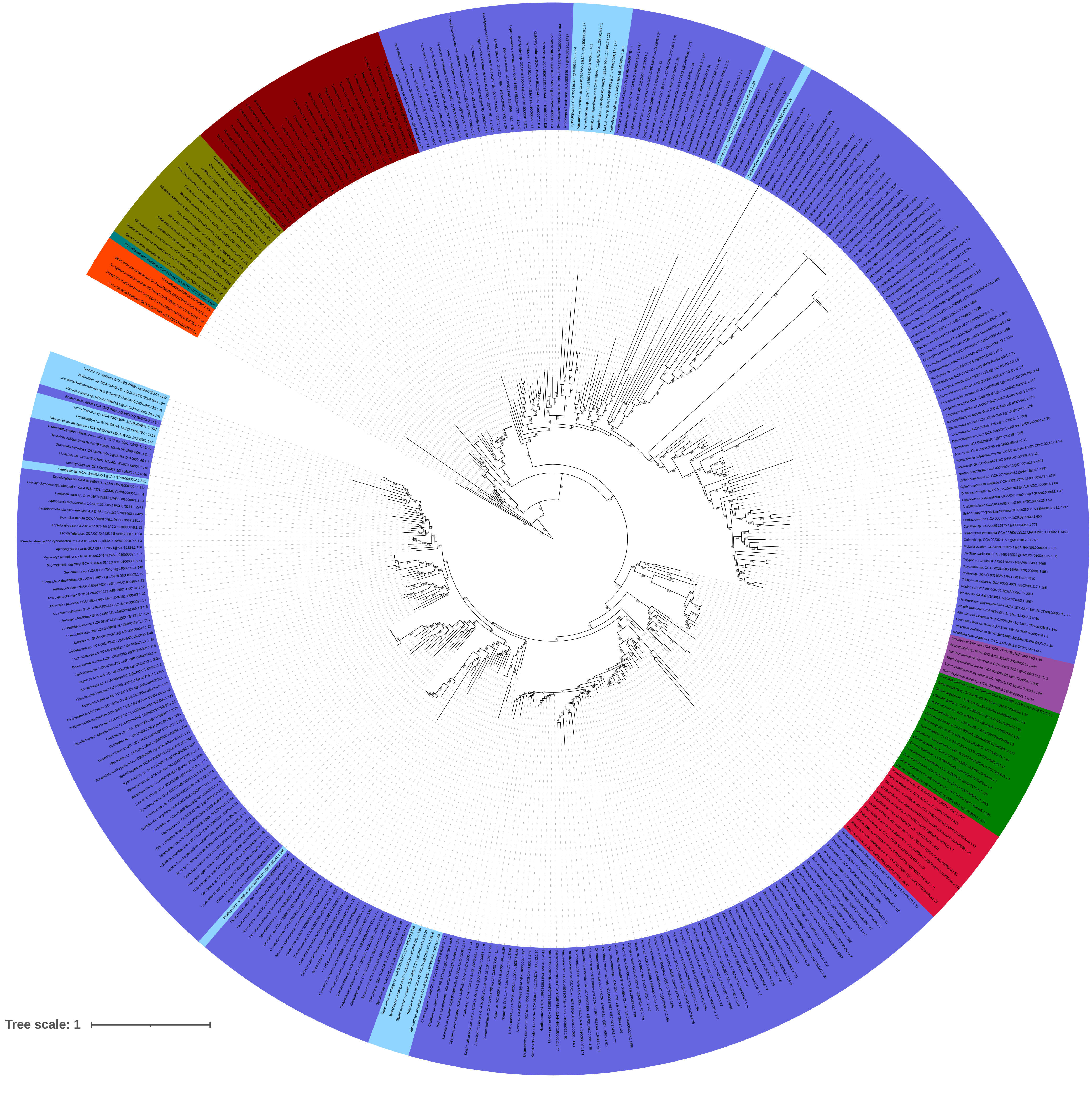

Fig. S101 - PAM71

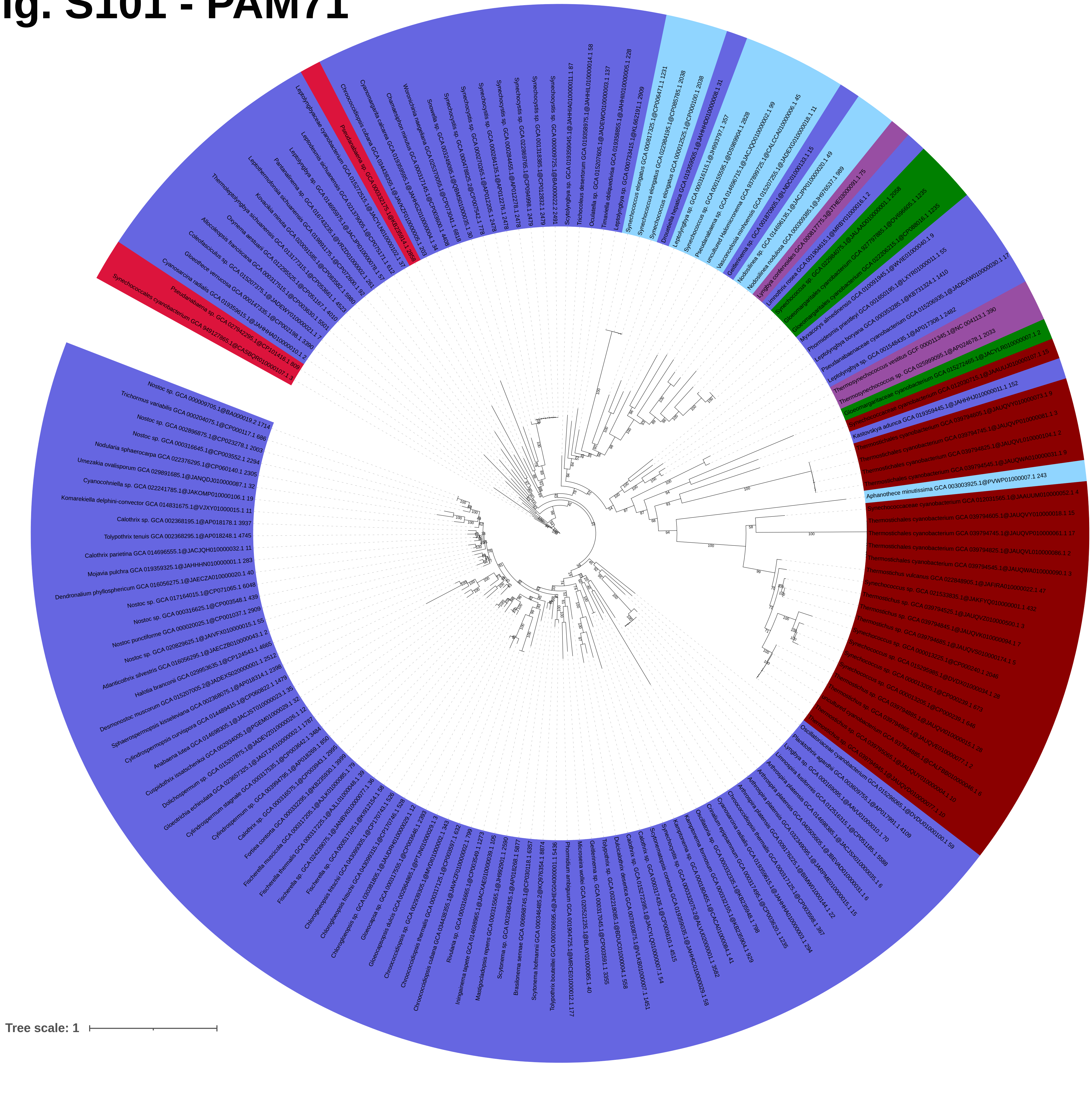

Fig. S102 - Pitt

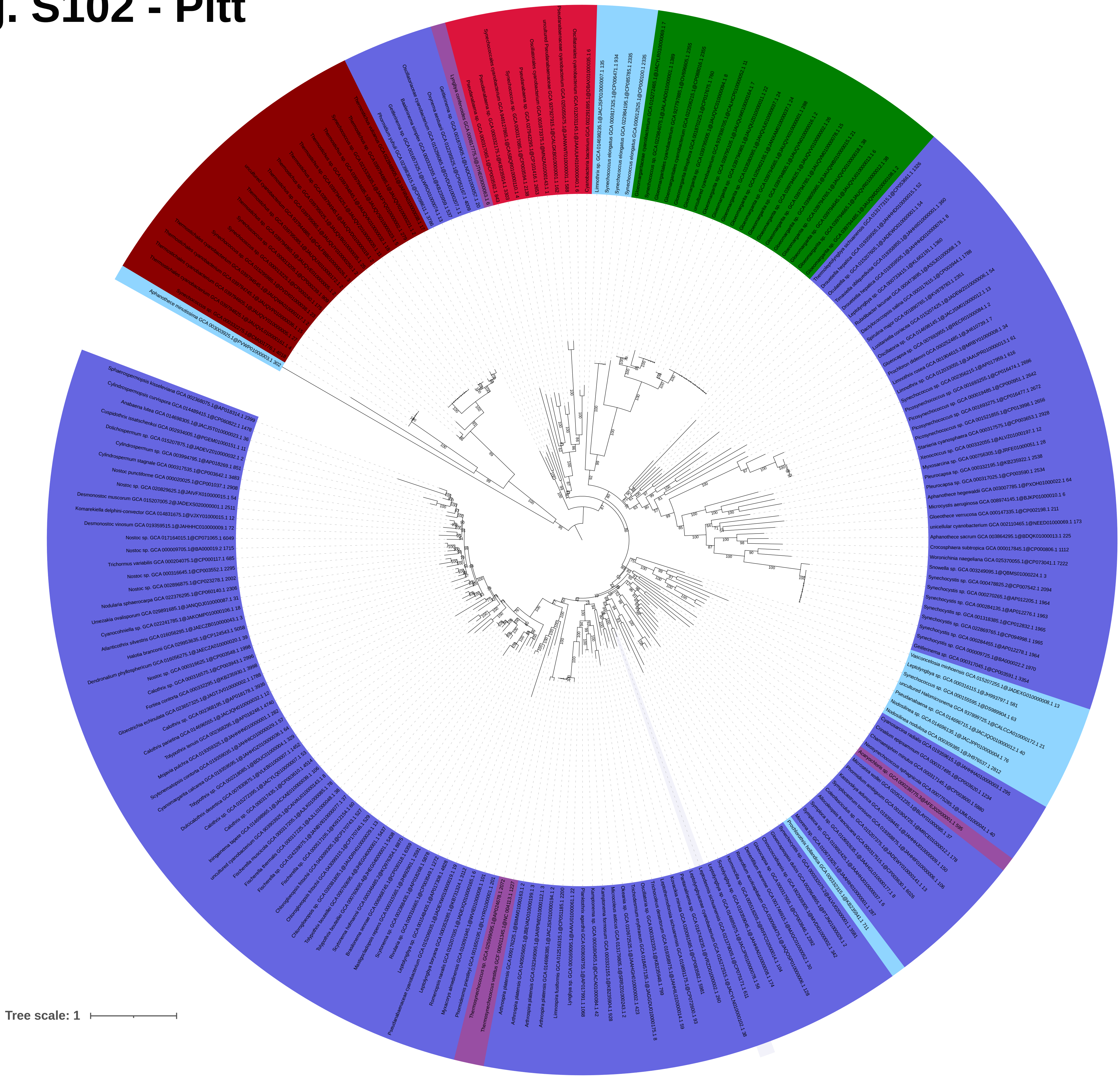

Fig. S103 - PratA

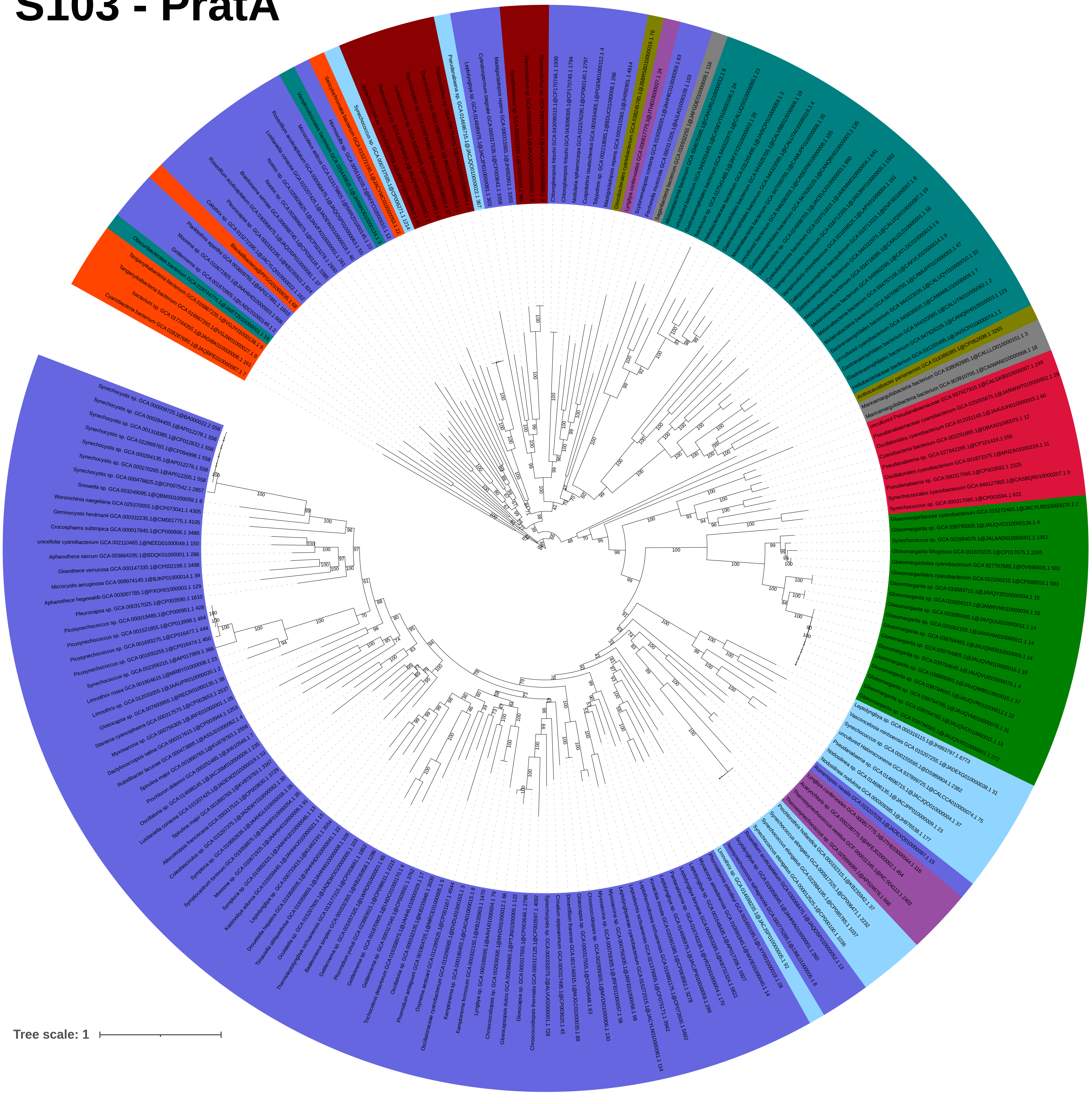

Fig. S104 - Psb27

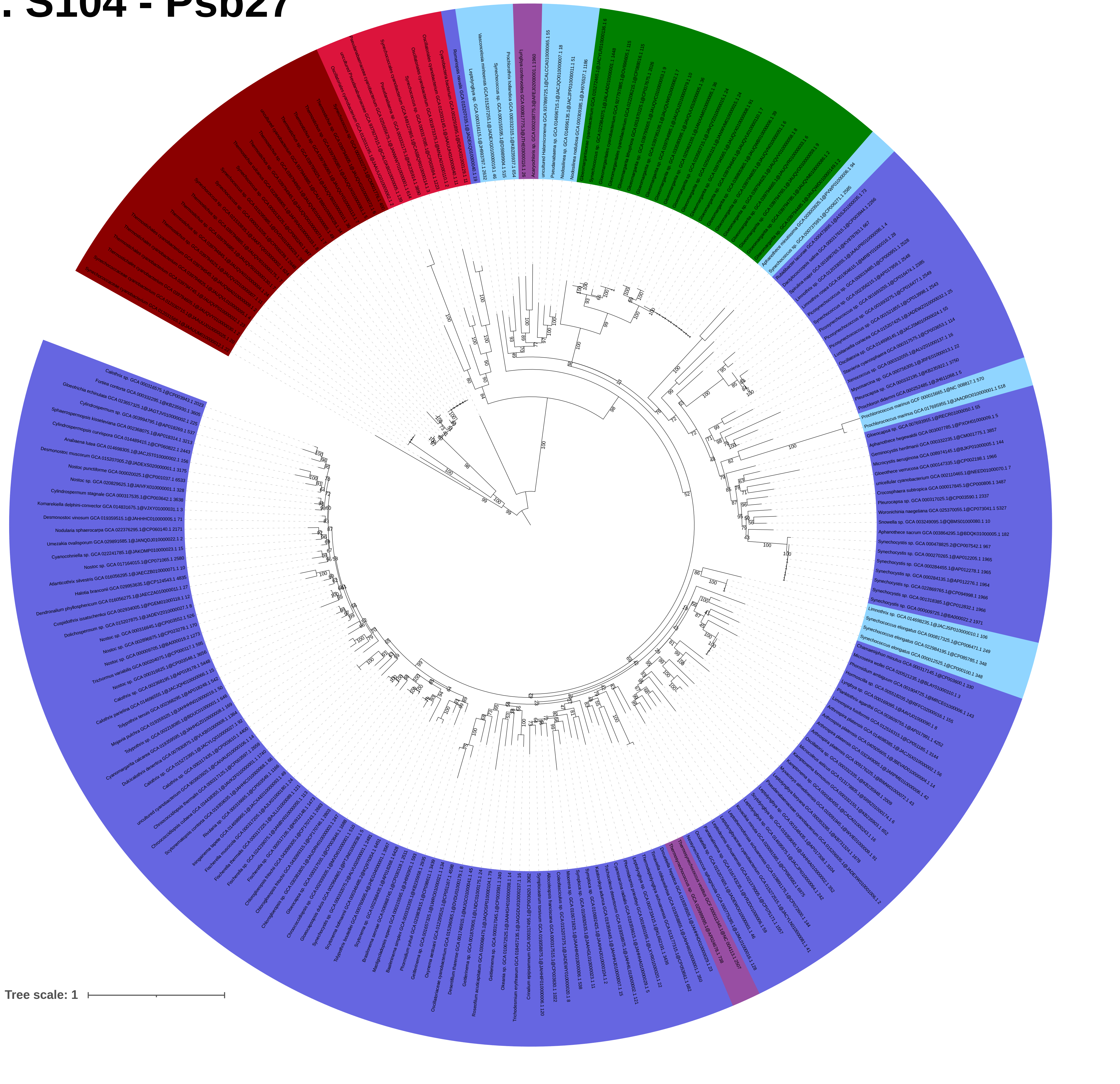

Fig. S105 - Psb28

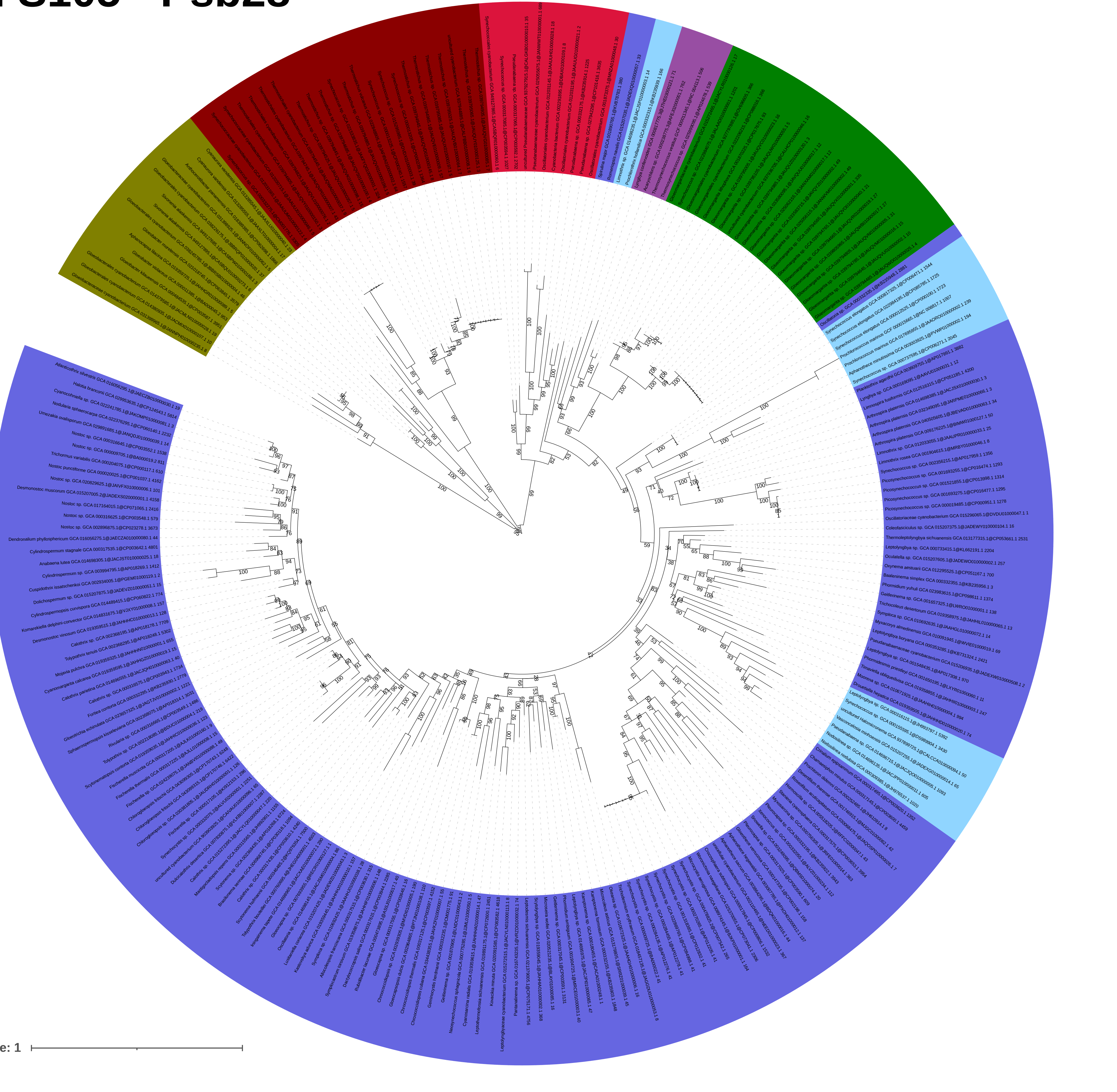

Fig. S106 - Psb29

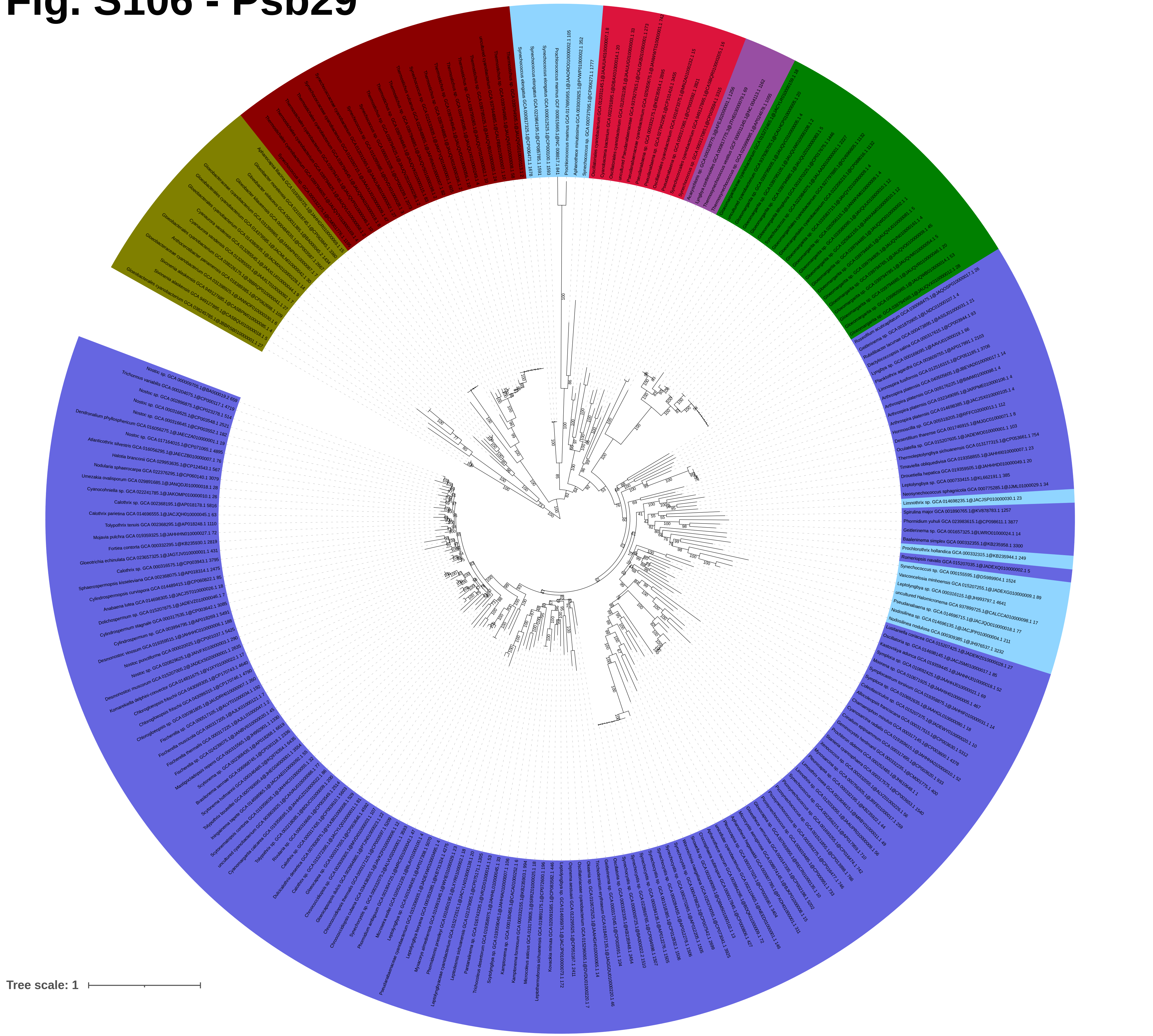

Fig. S107 - Psb32

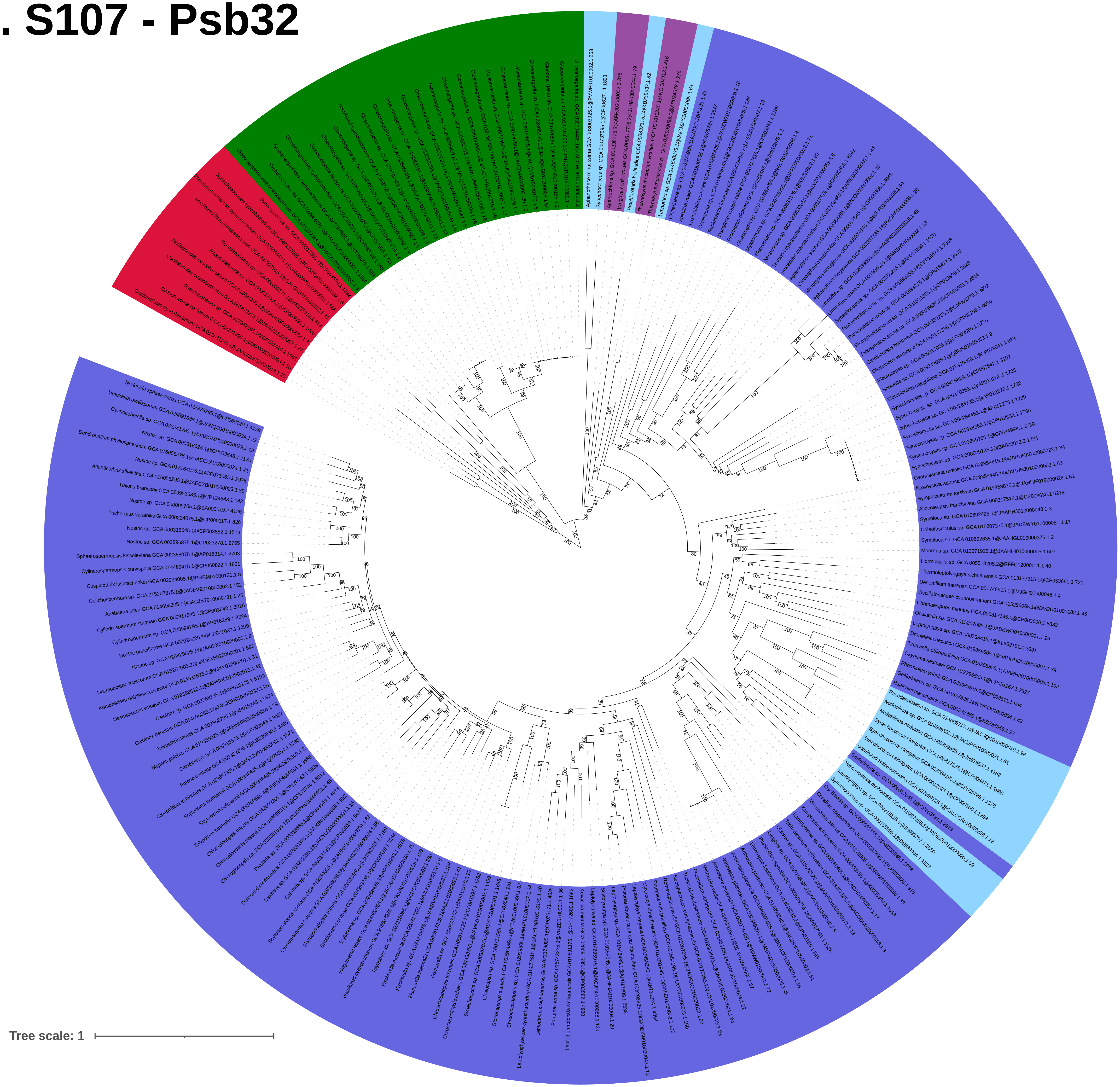

Fig. S108 - Psb34

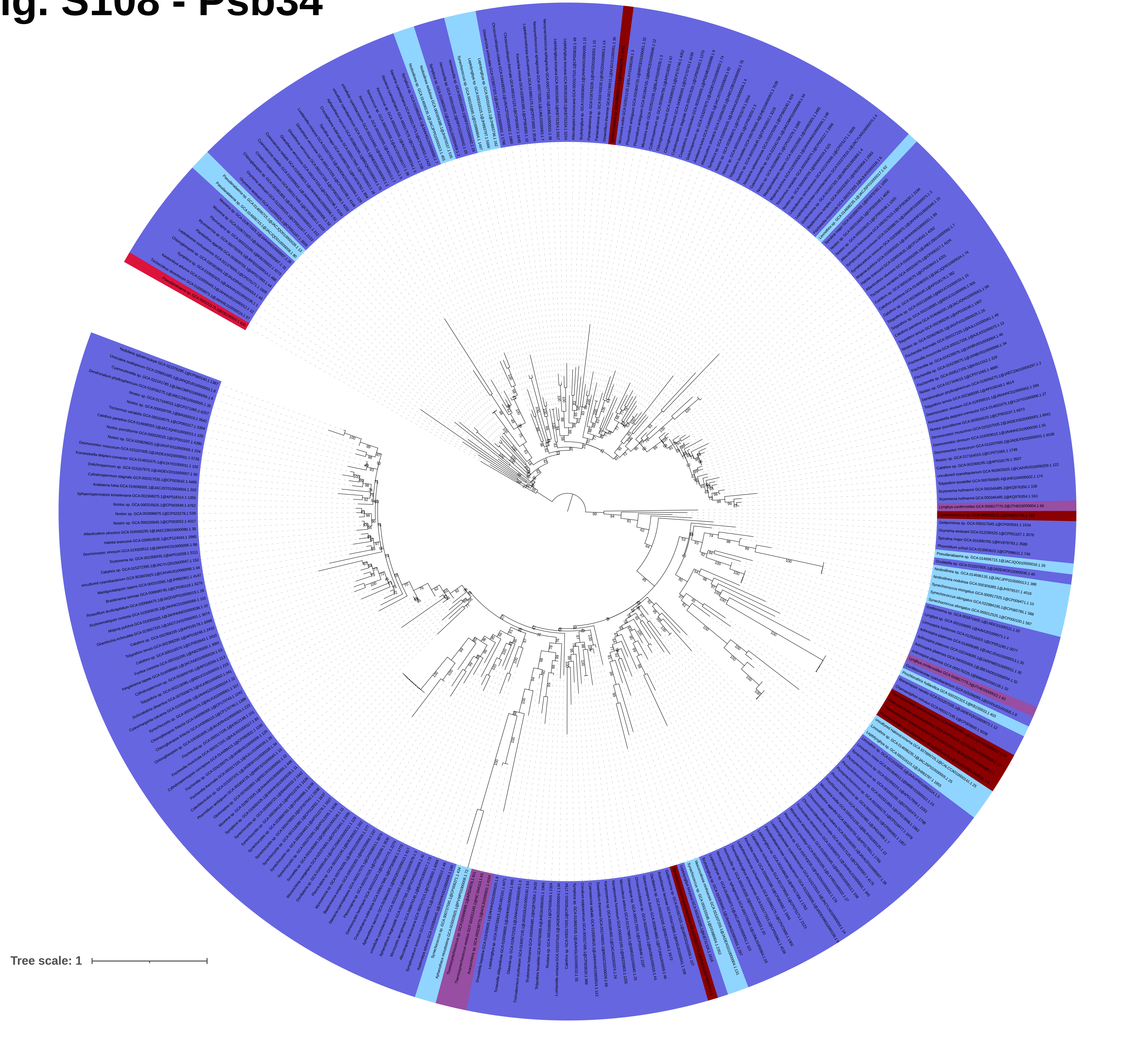

Fig. S109 - Psb35

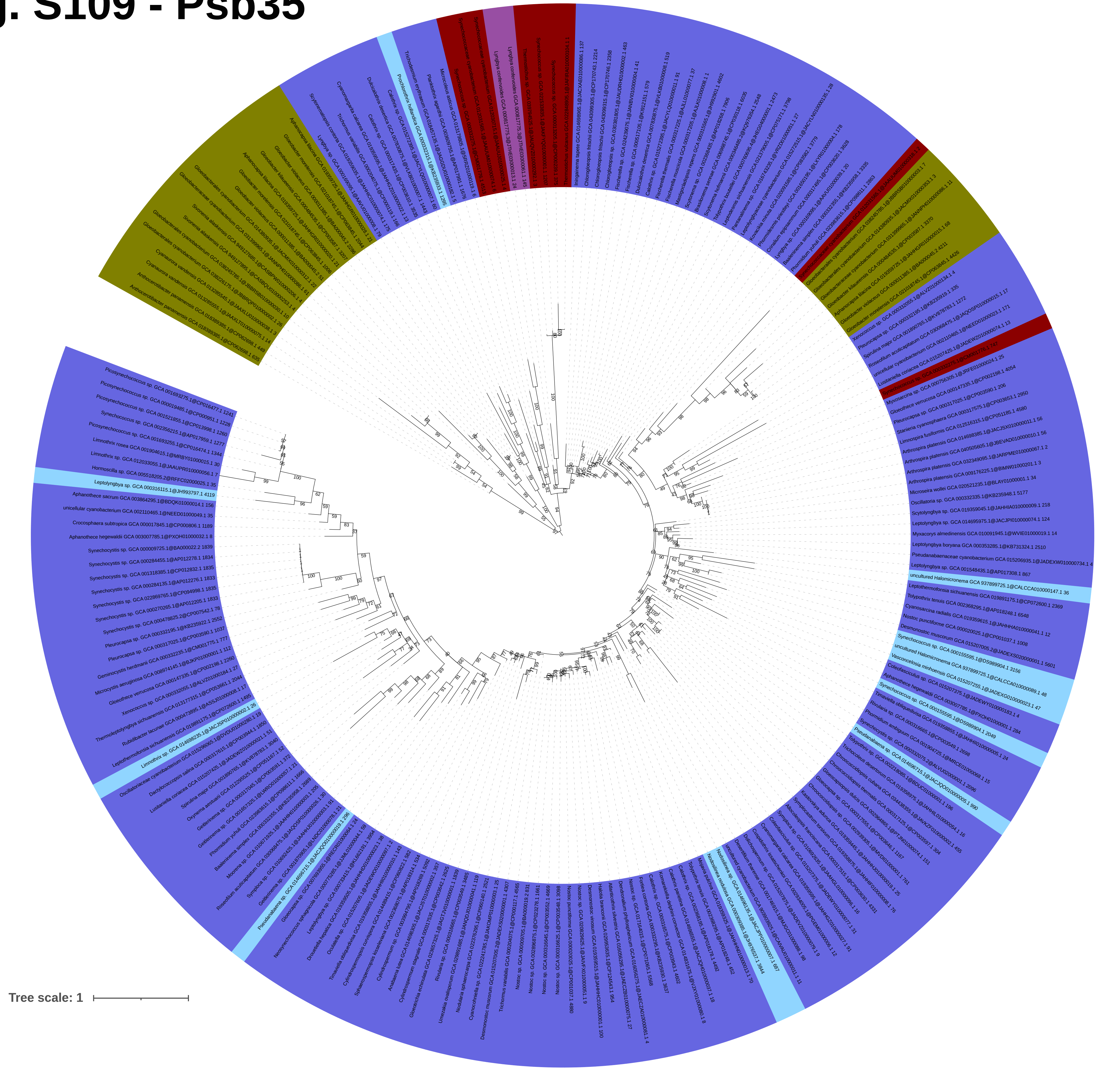

Fig. S110 - PsbN

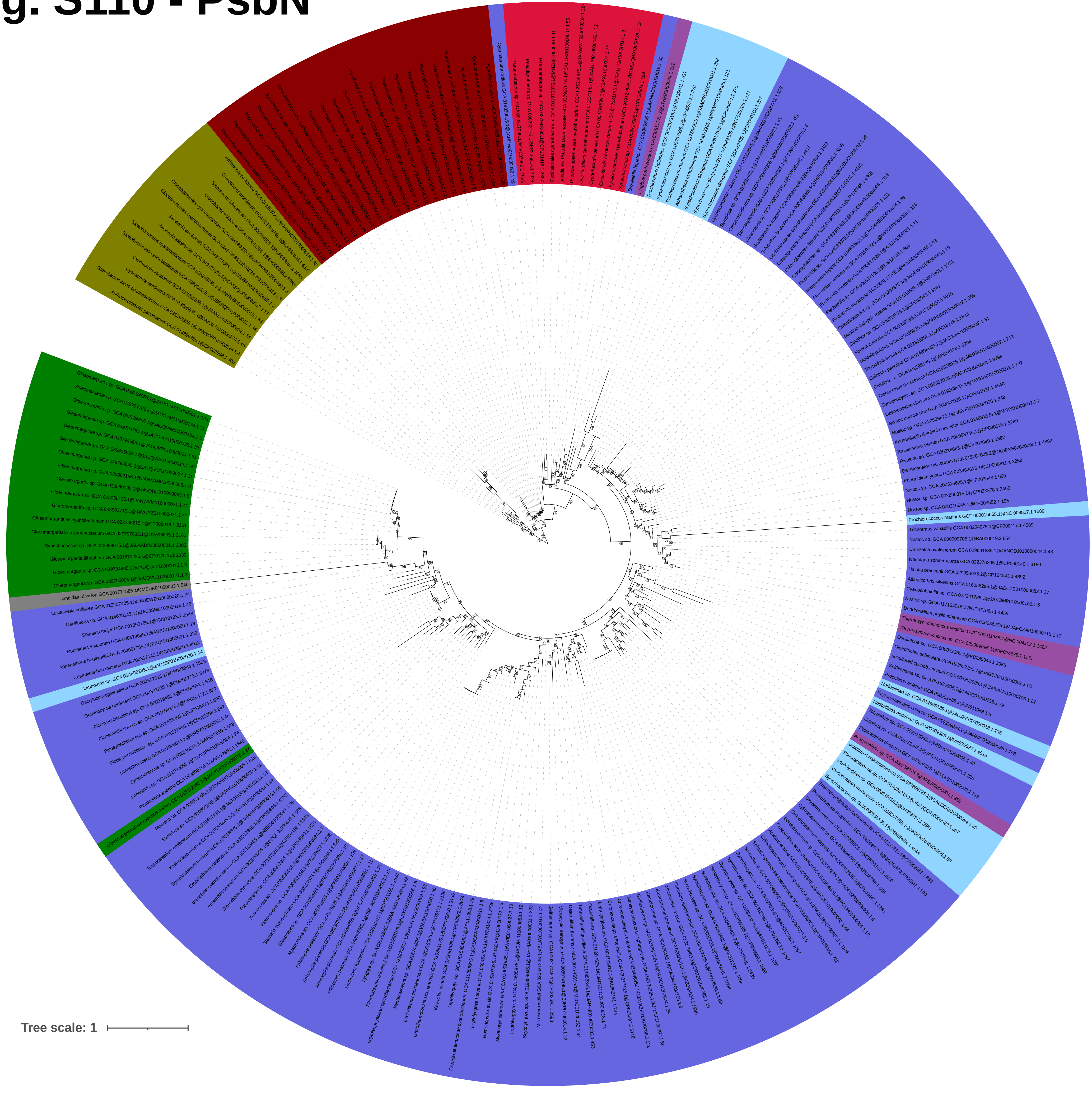

Fig. S111 - RubA

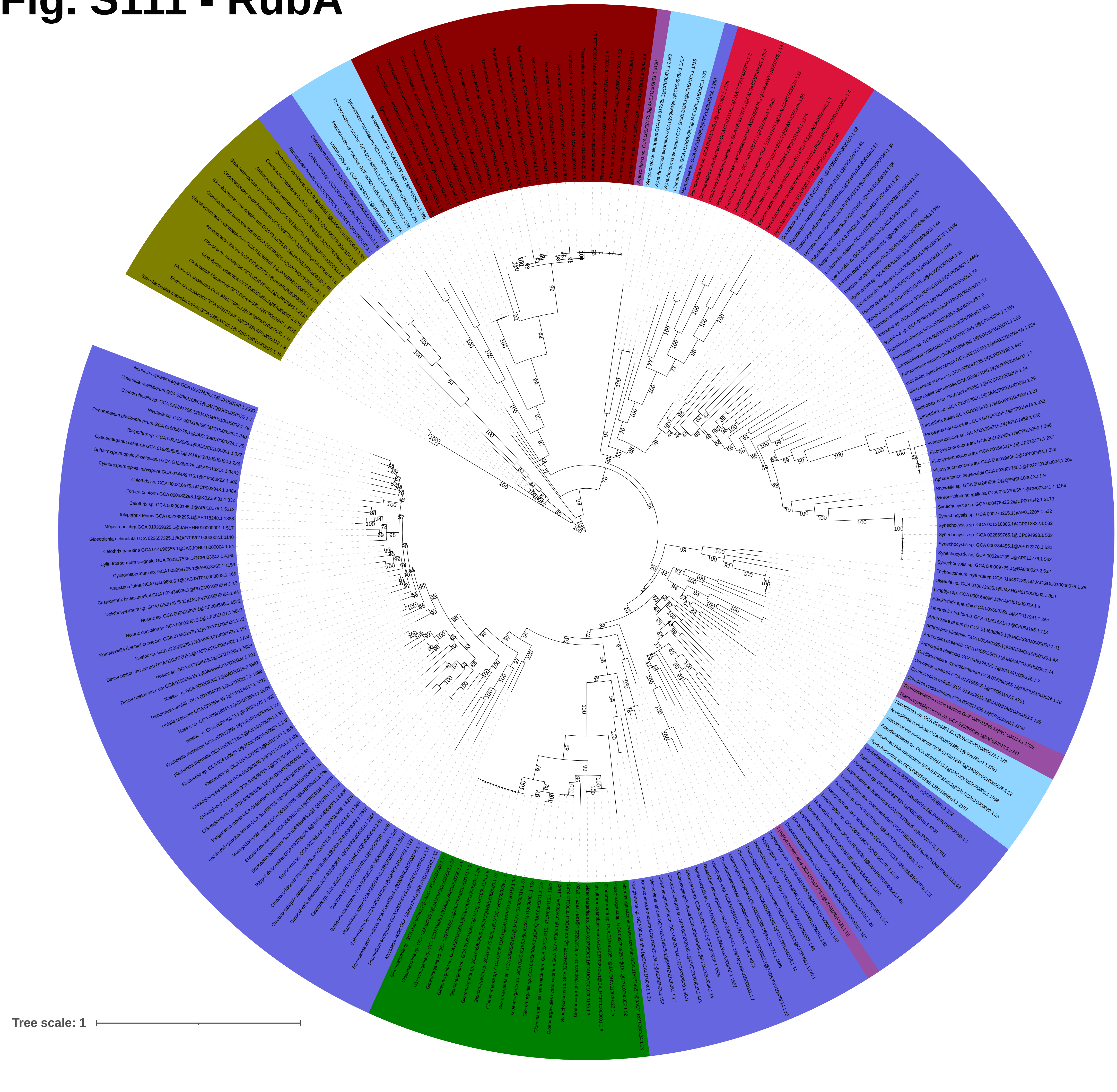

Fig. S112 - SecA

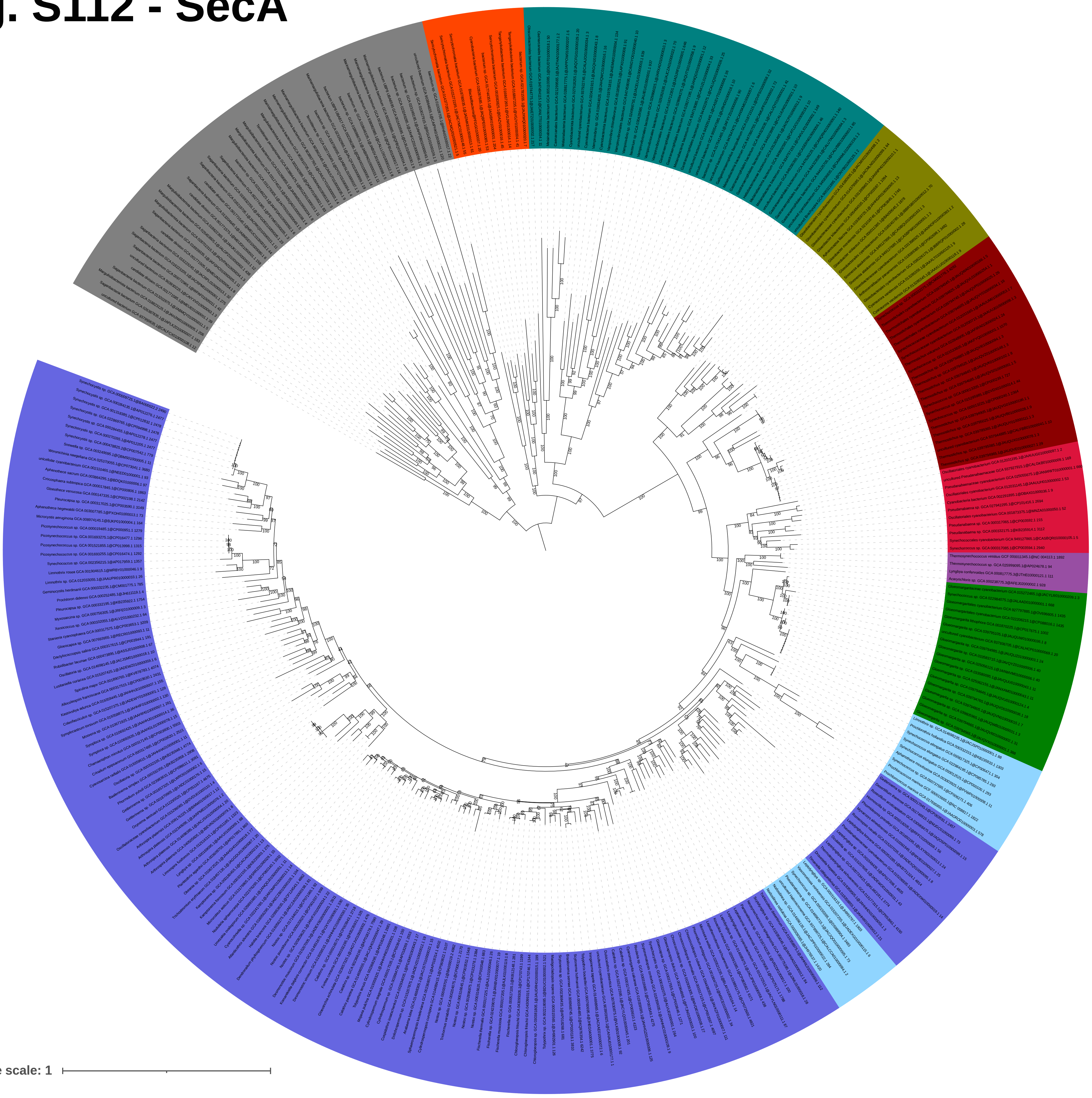

Fig. S113 - SecD

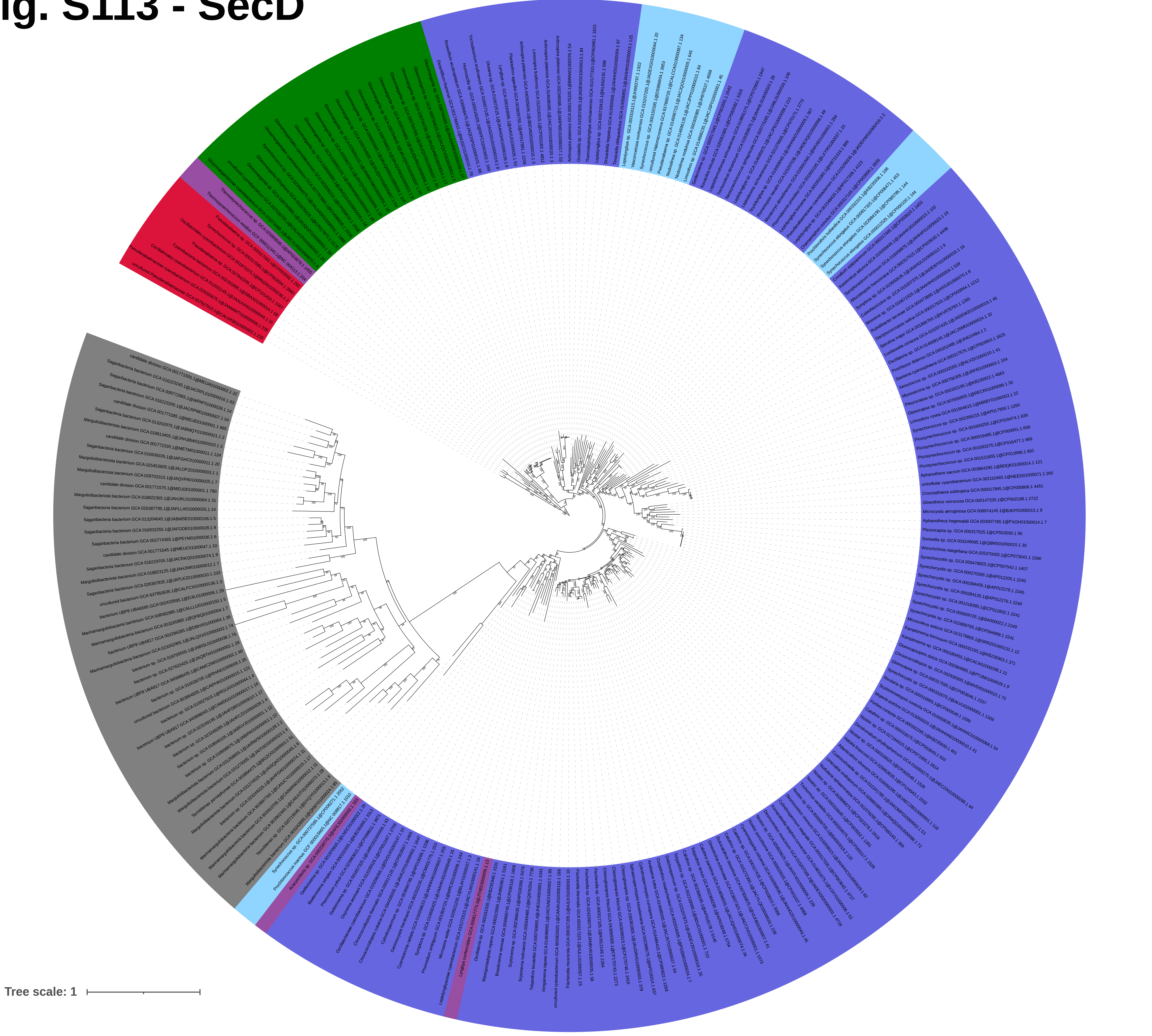

Fig. S114- SecE

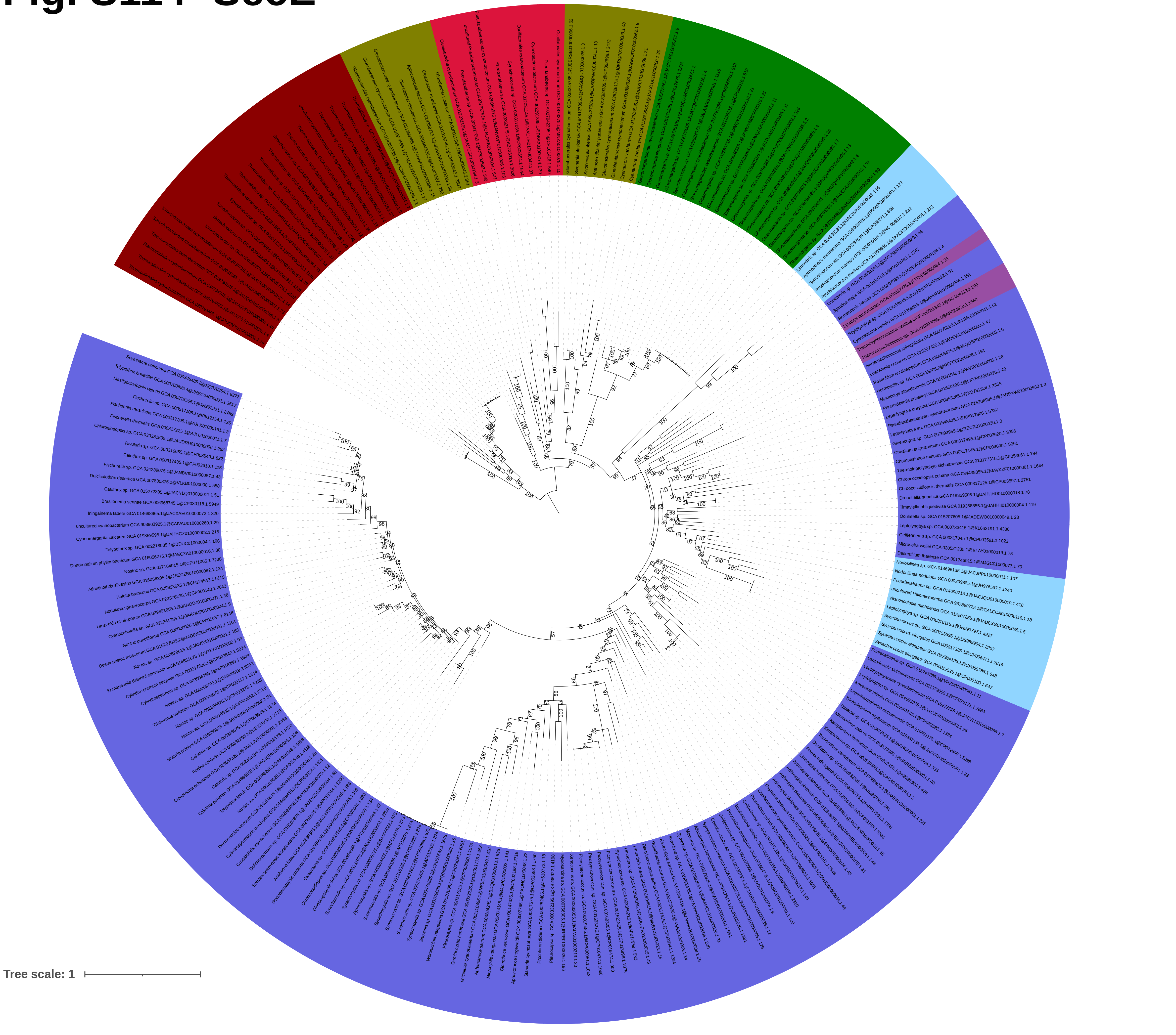

Fig. S115- SecF

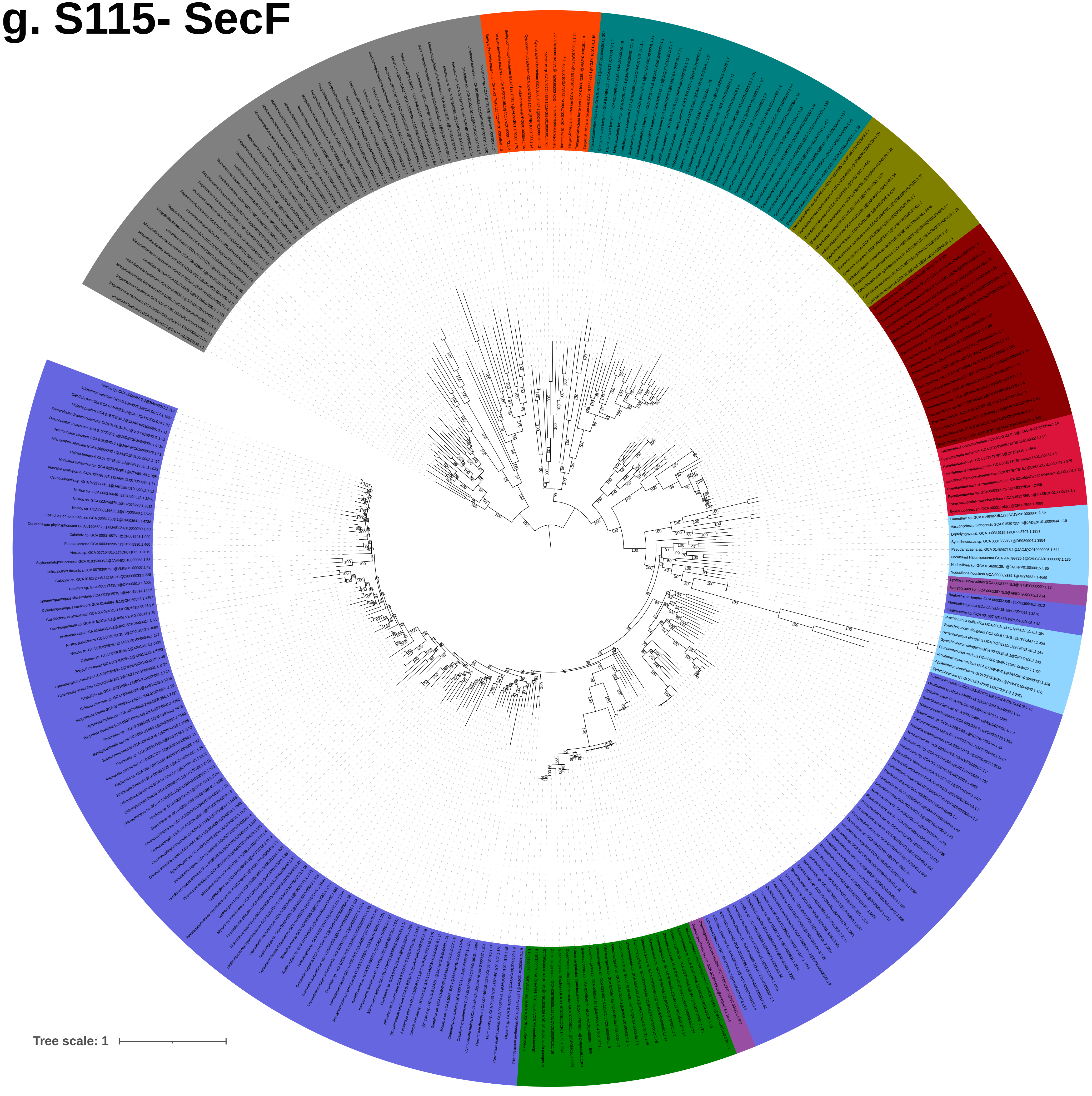

Fig. S116 - SecG

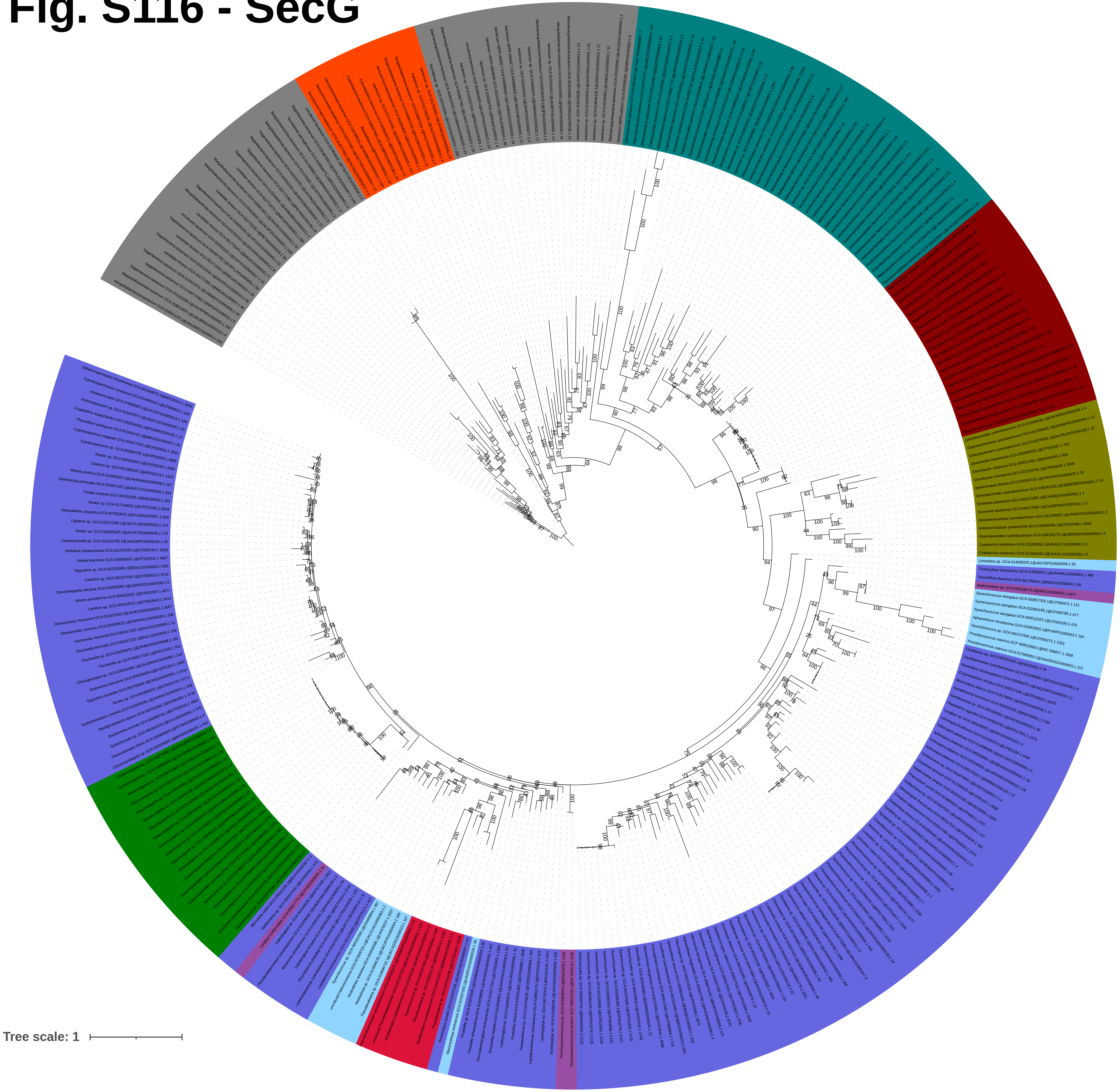

Fig. S117- SecY

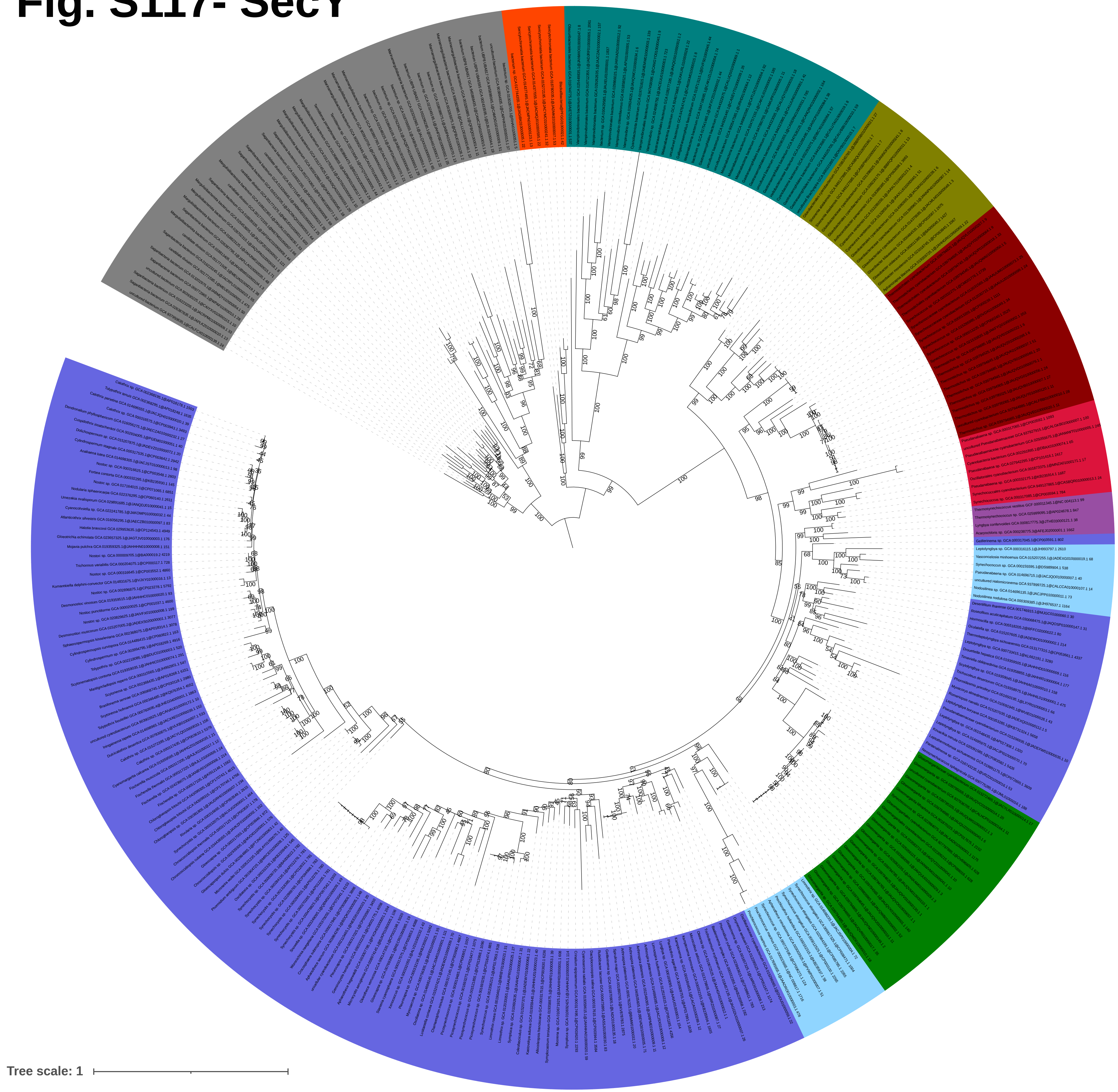

Fig. S118 - Sll0408 (TLP40)

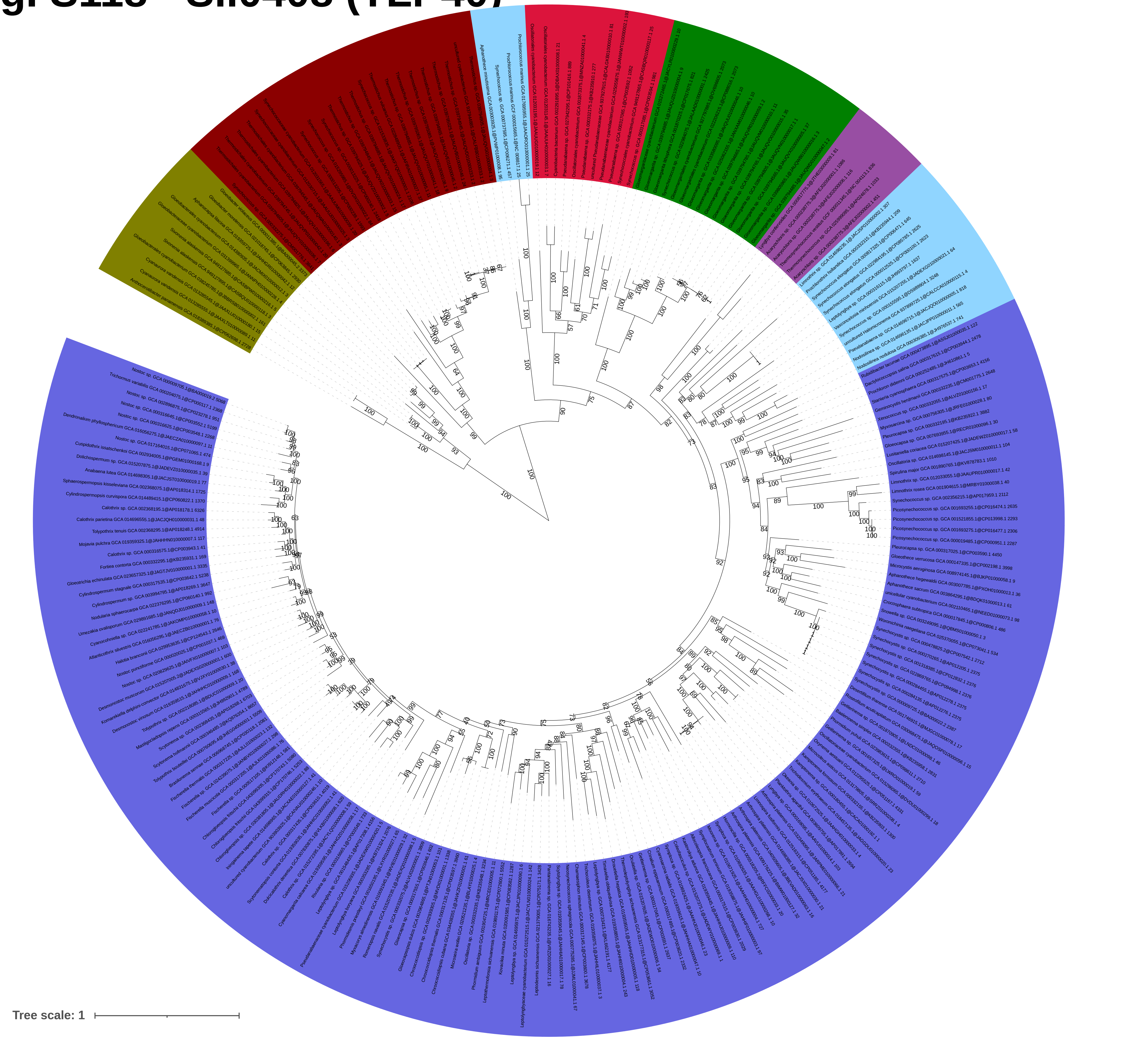

Fig. S119 - Sll0509

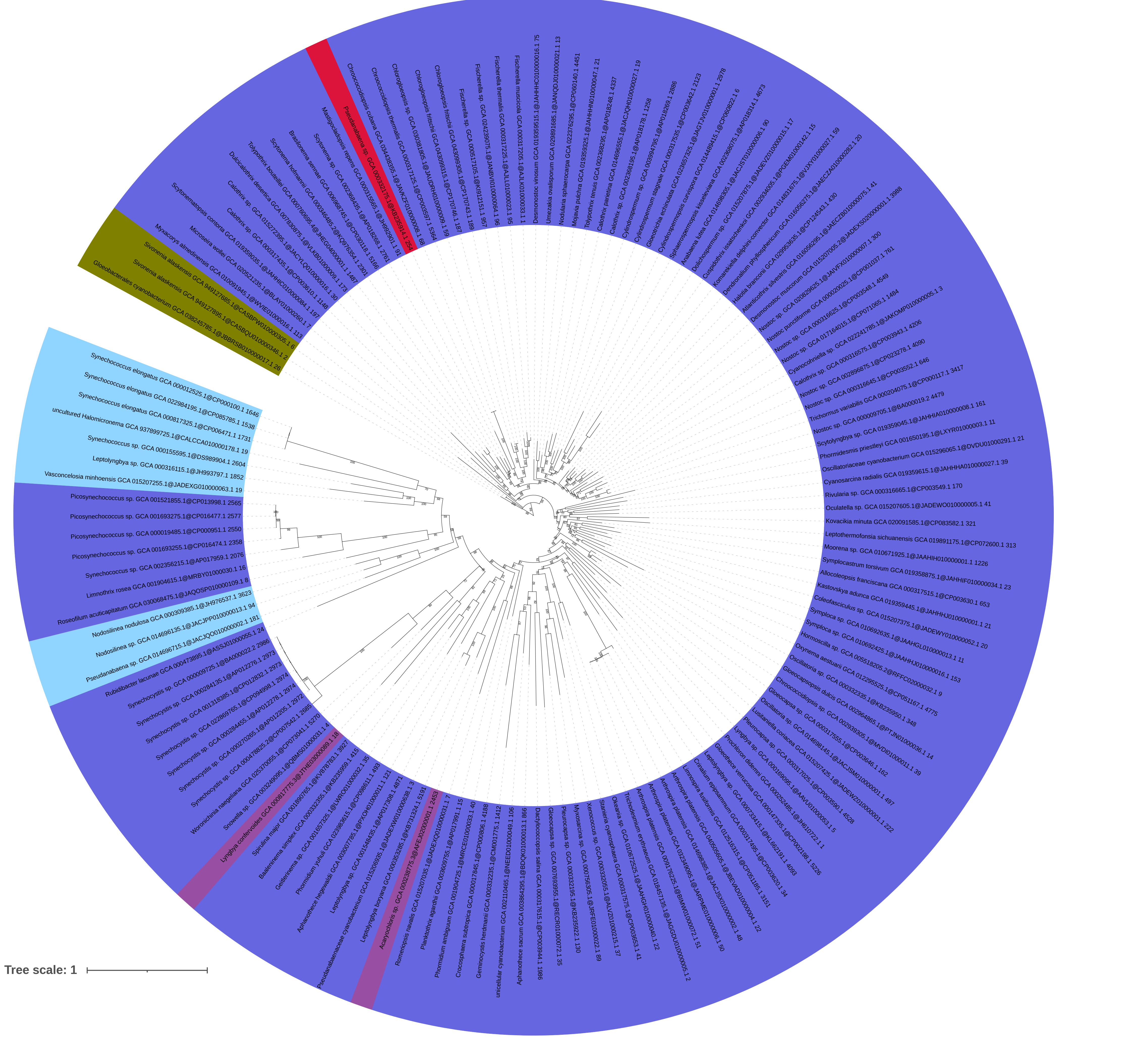

Fig. S120 - Sll0606

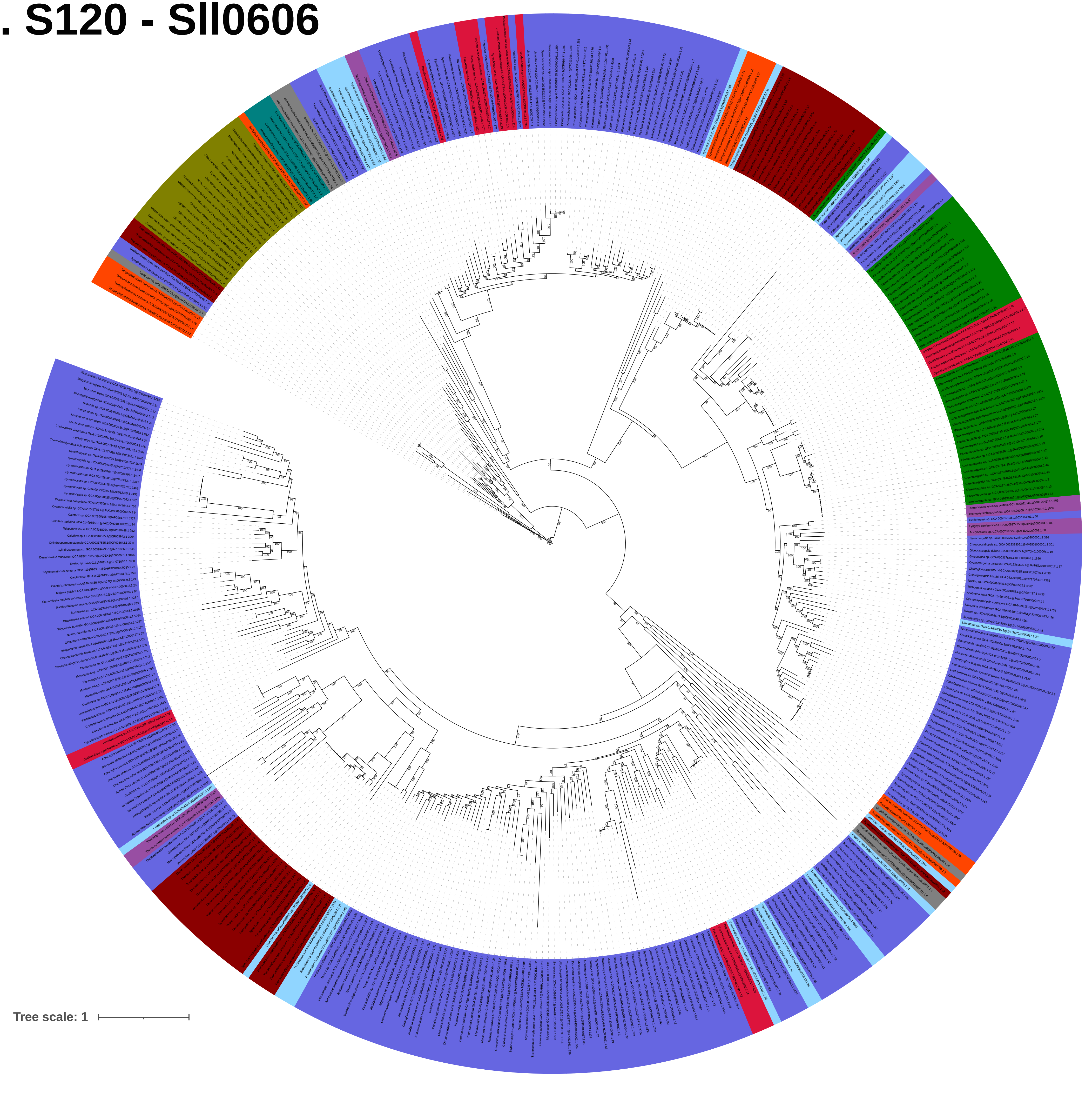

Fig. S121 - Sll0933 (Pam68)

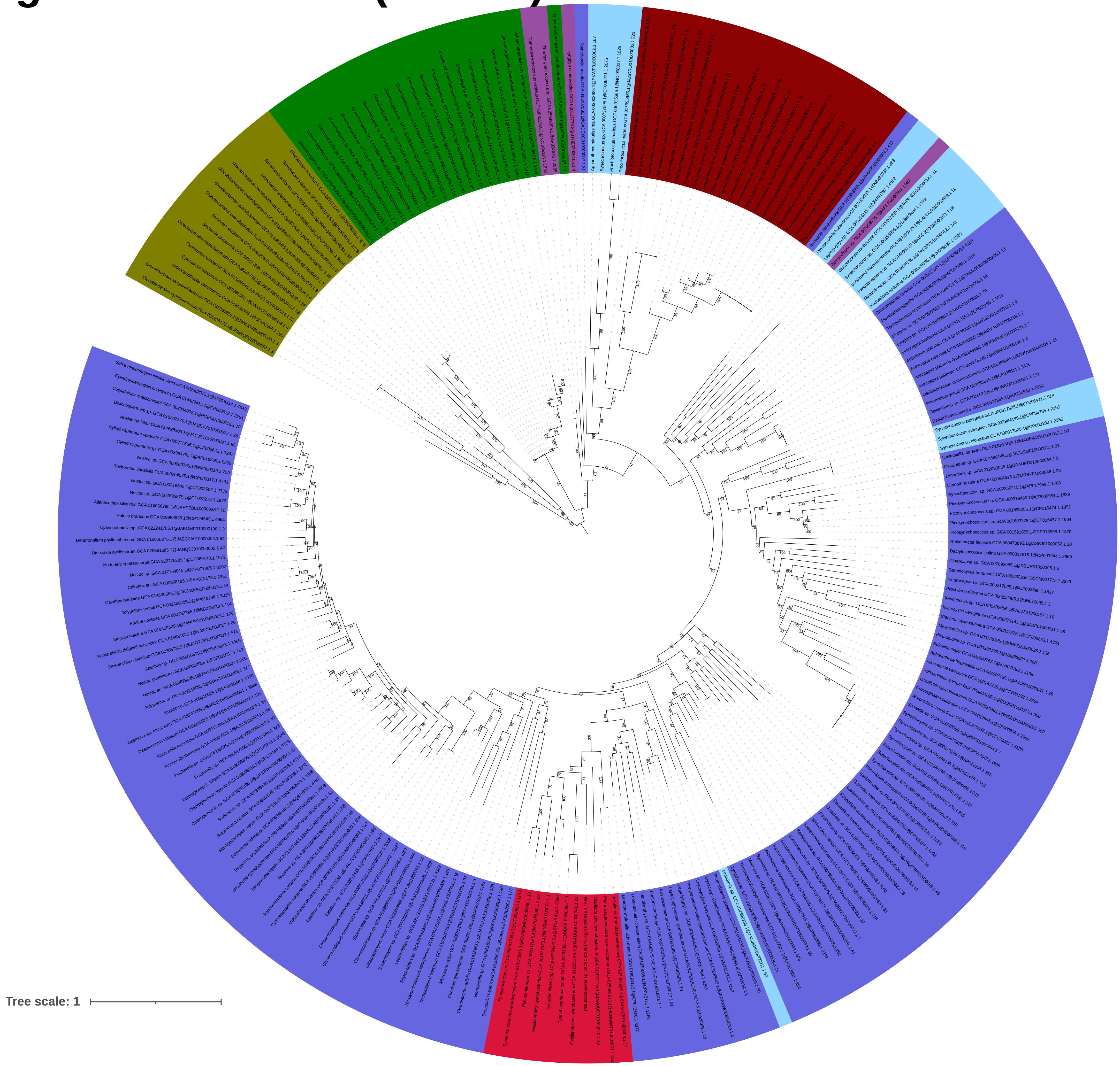

Fig. S122 - Sll1021

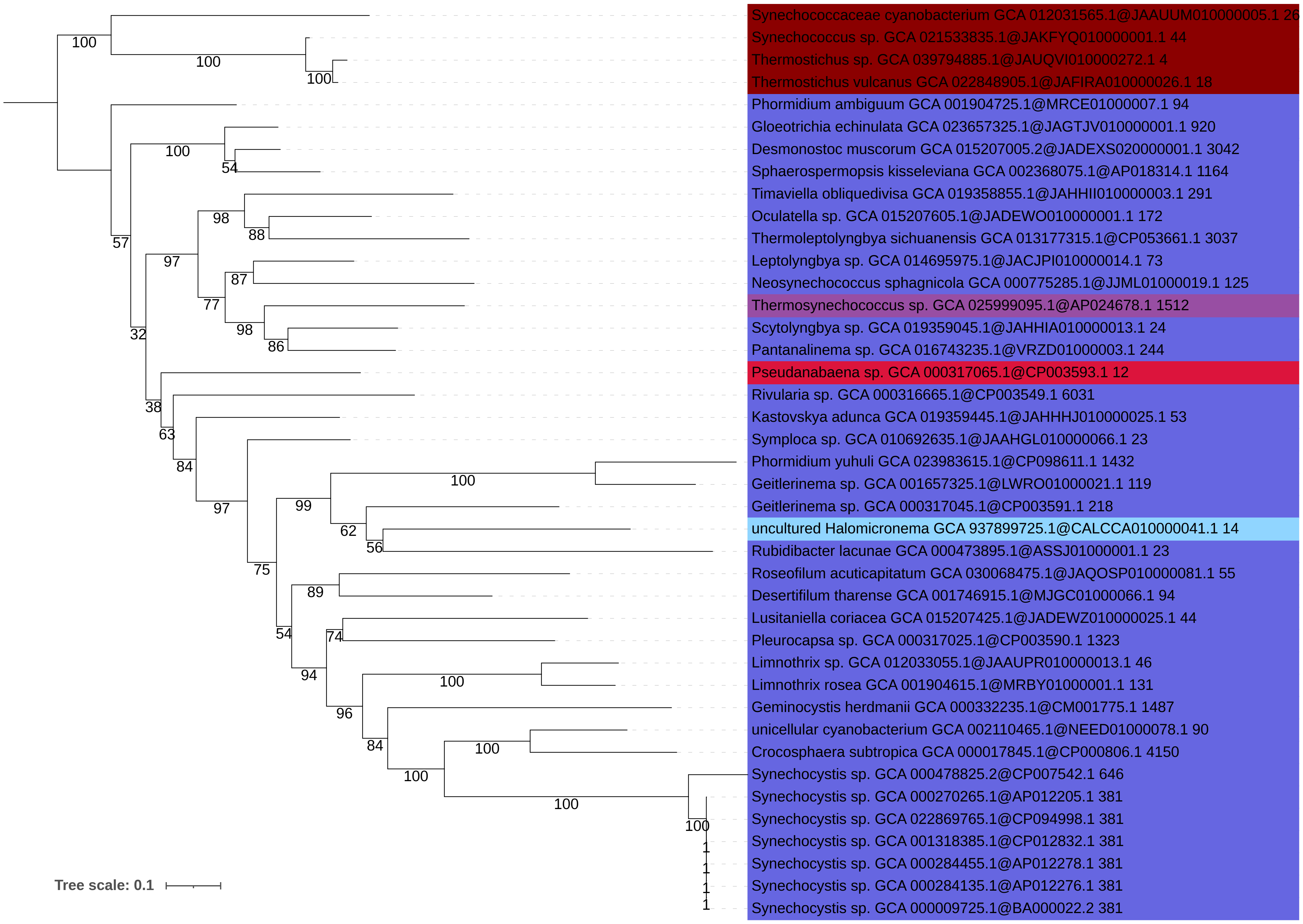

Fig. S123 - Slr0232

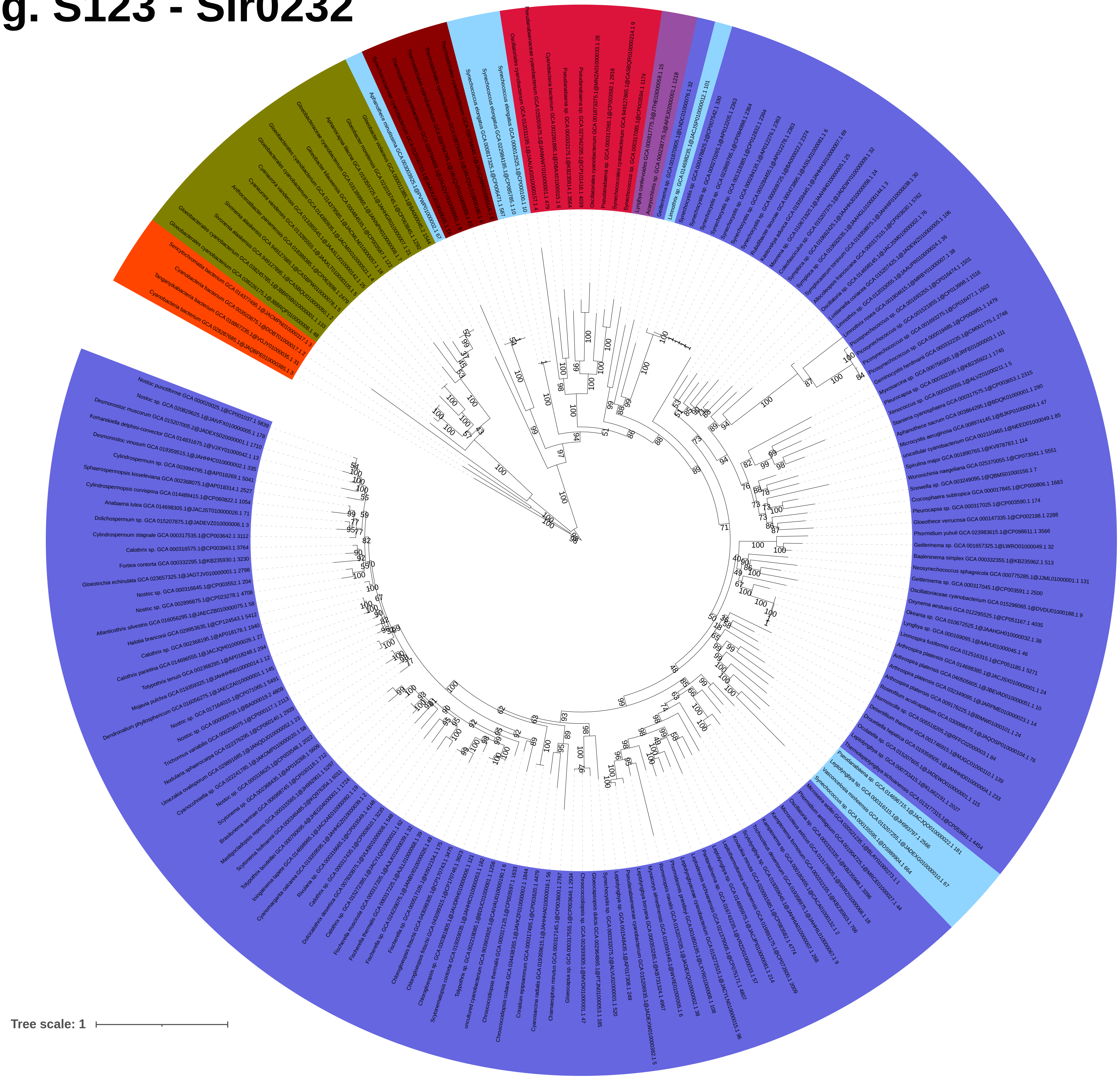

Fig. S124 - Slr0286

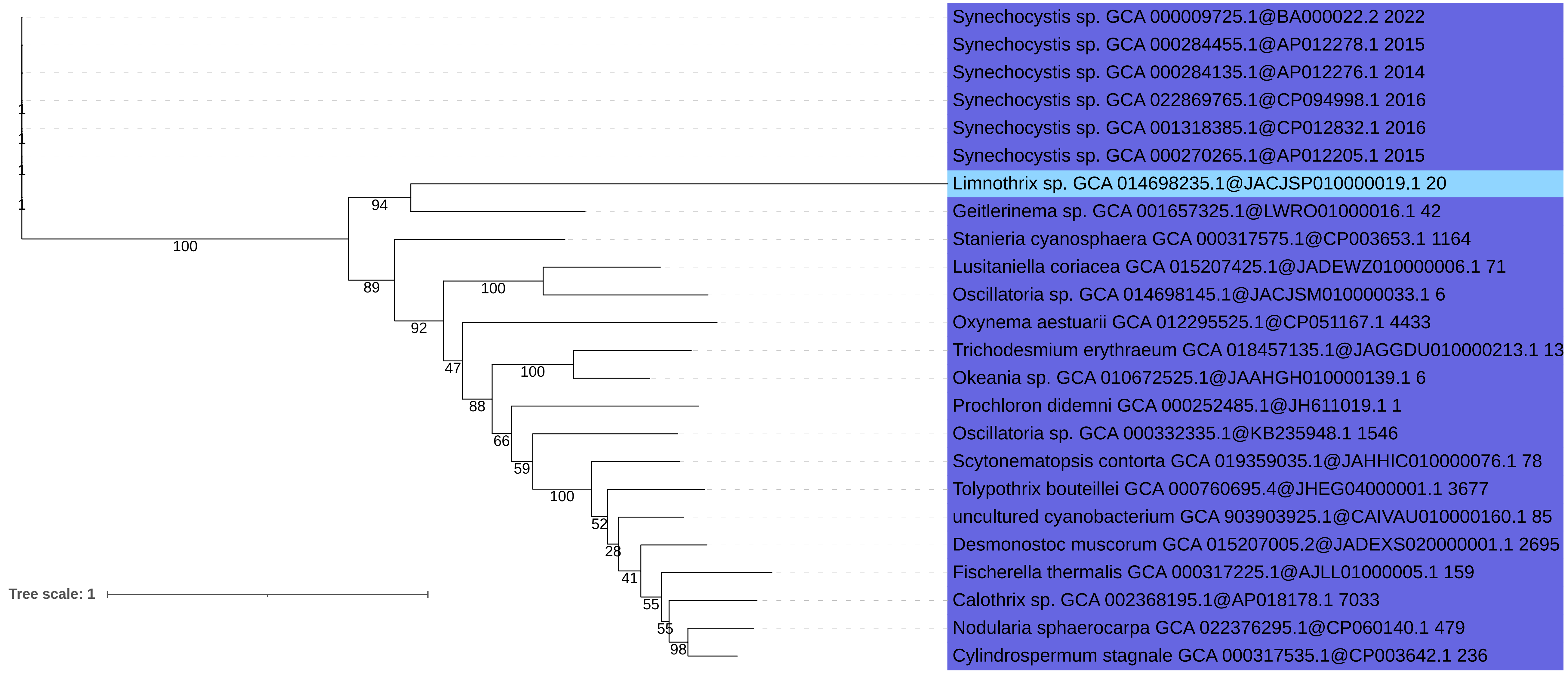

Fig. S125 - Slr0305

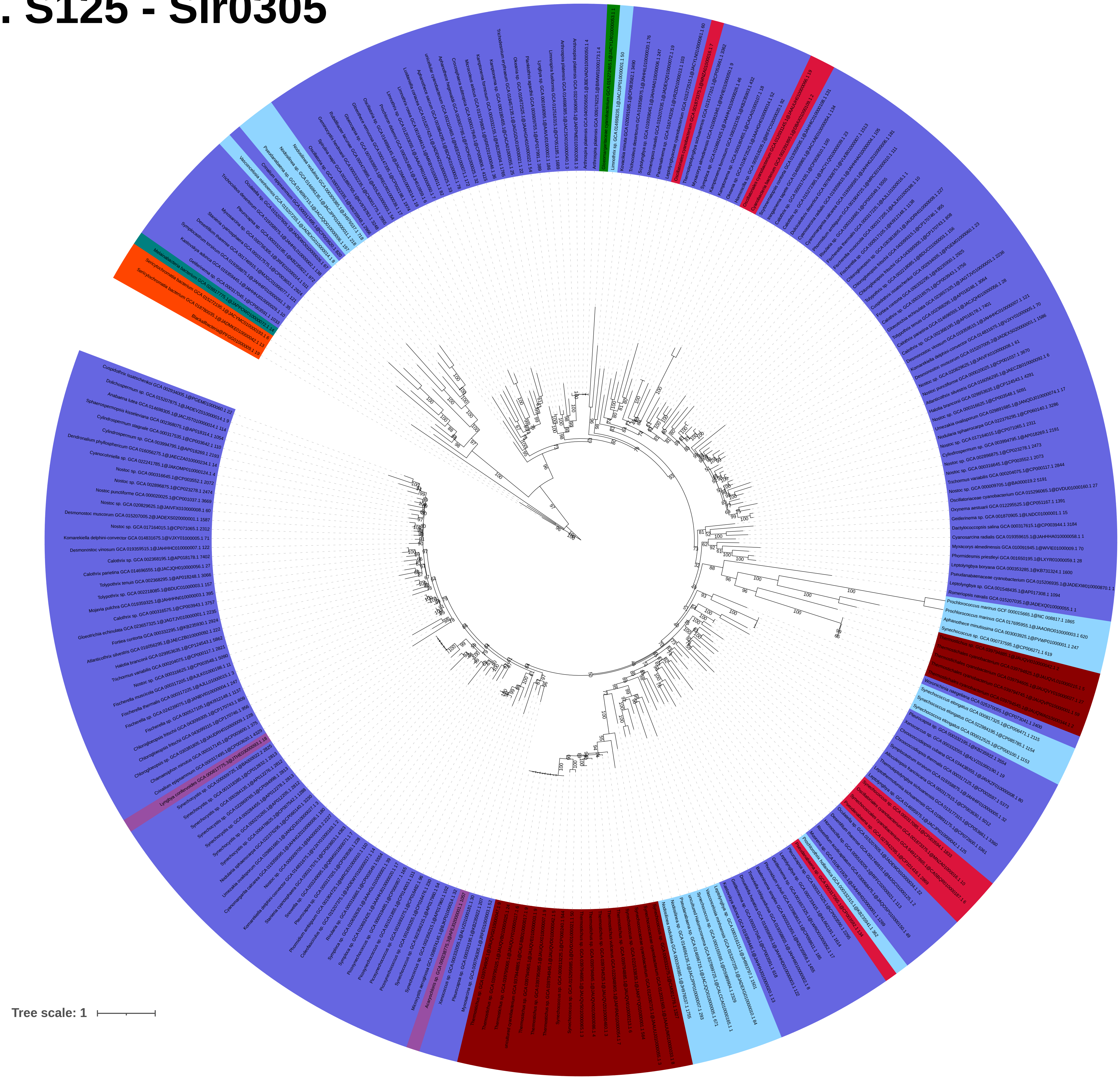

Fig. S126 - Slr0483

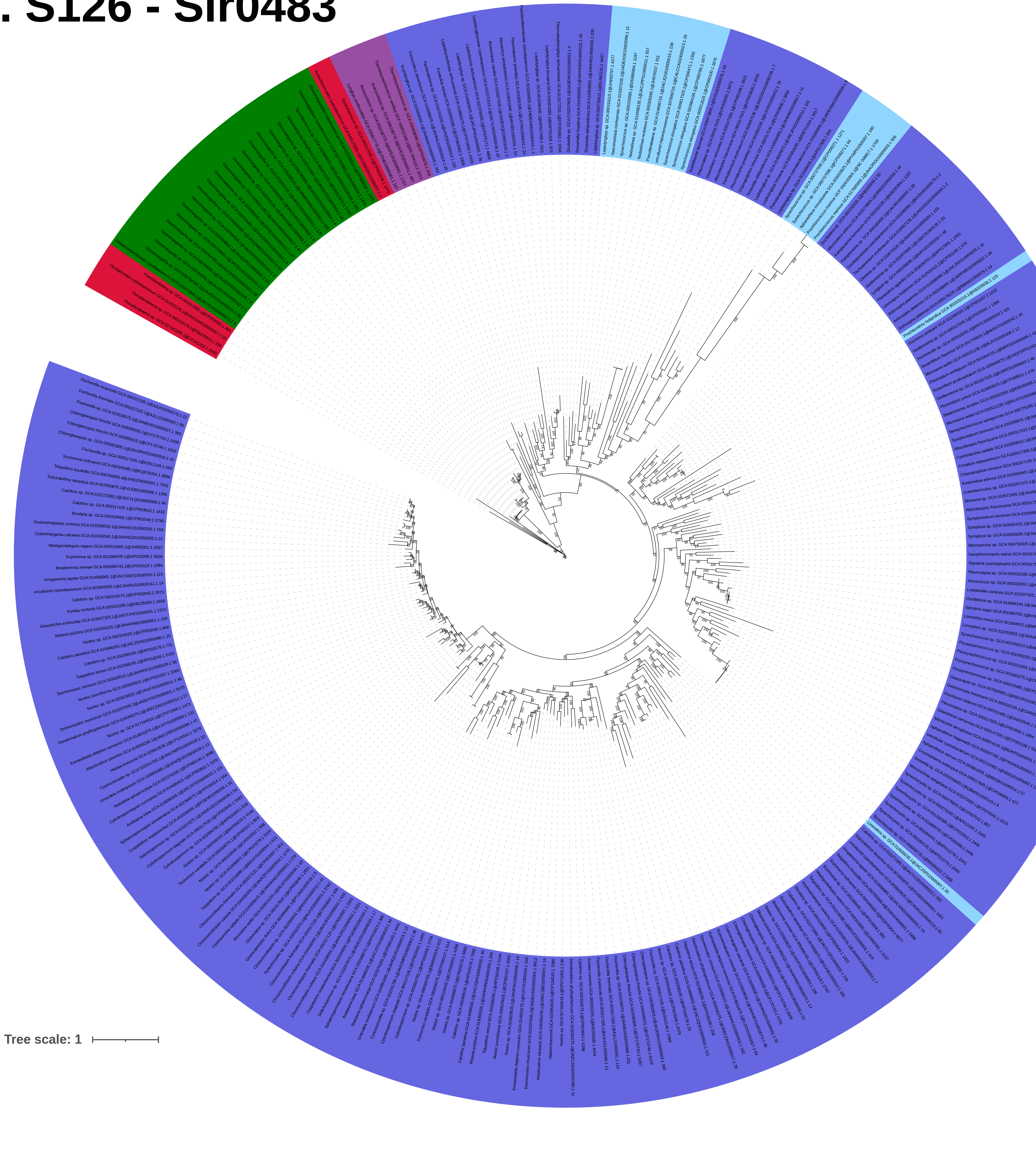

Fig. S127 - Slr0565

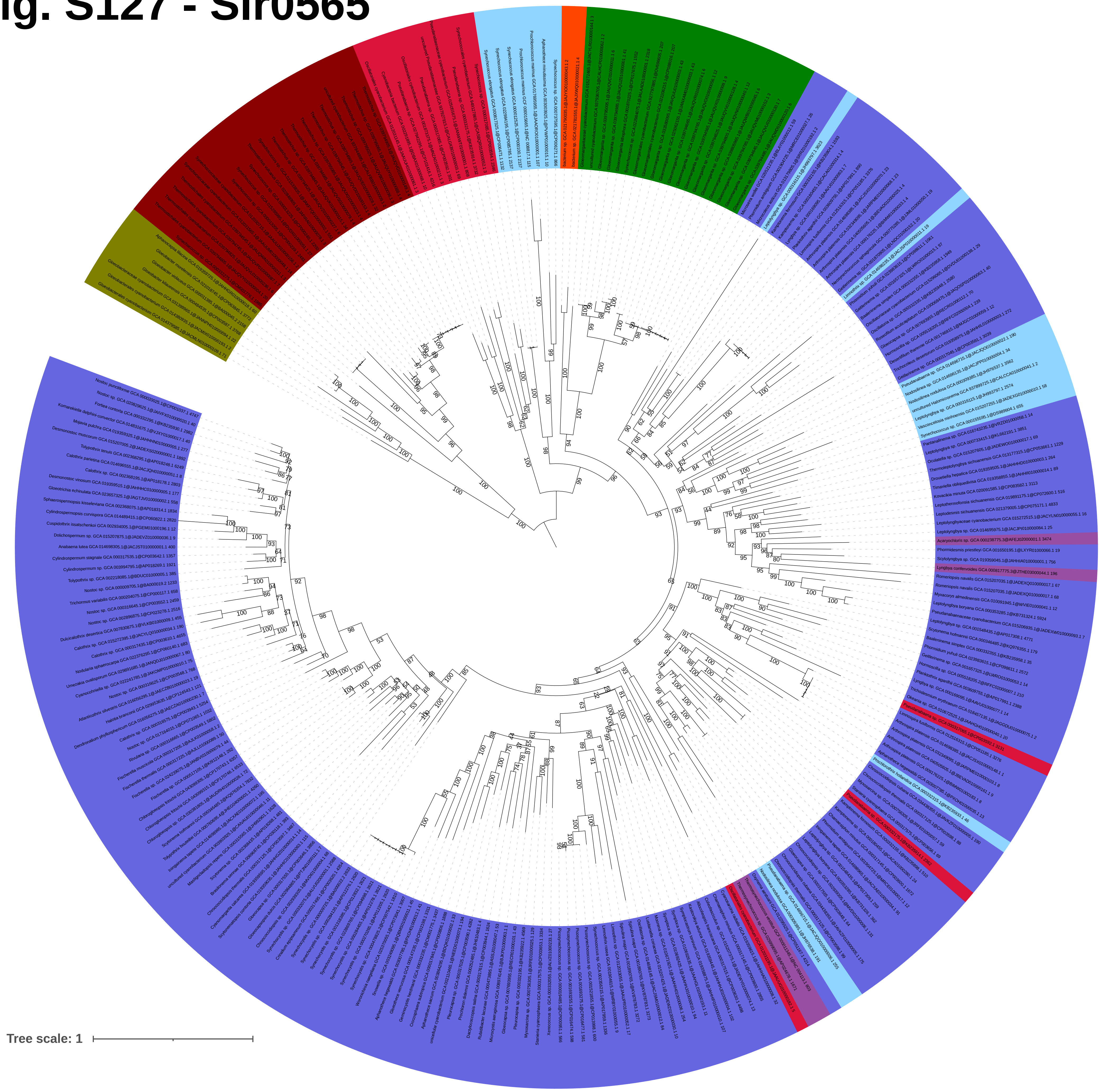

Fig. S128 - Slr0869

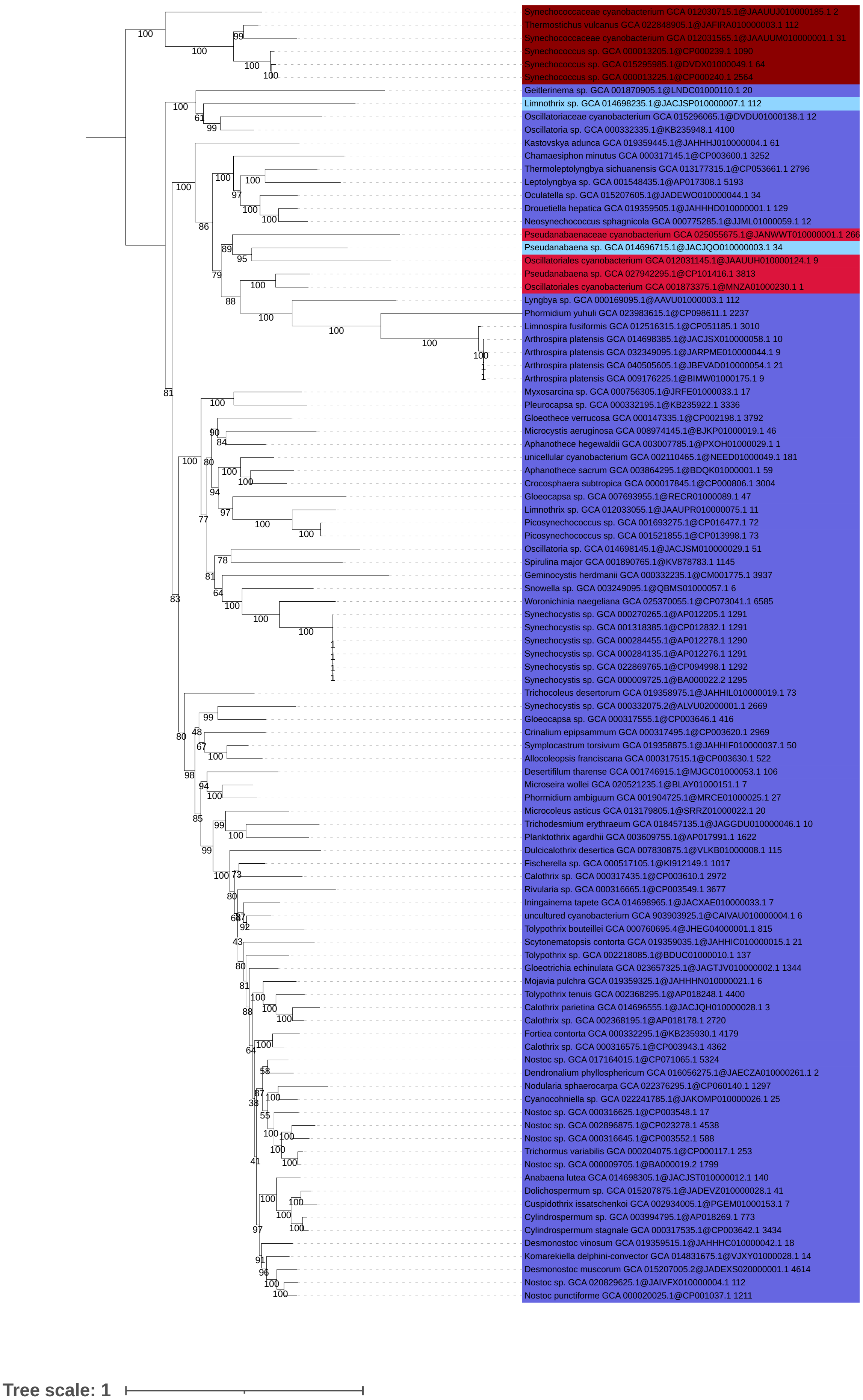

Fig. S129 - Slr1106

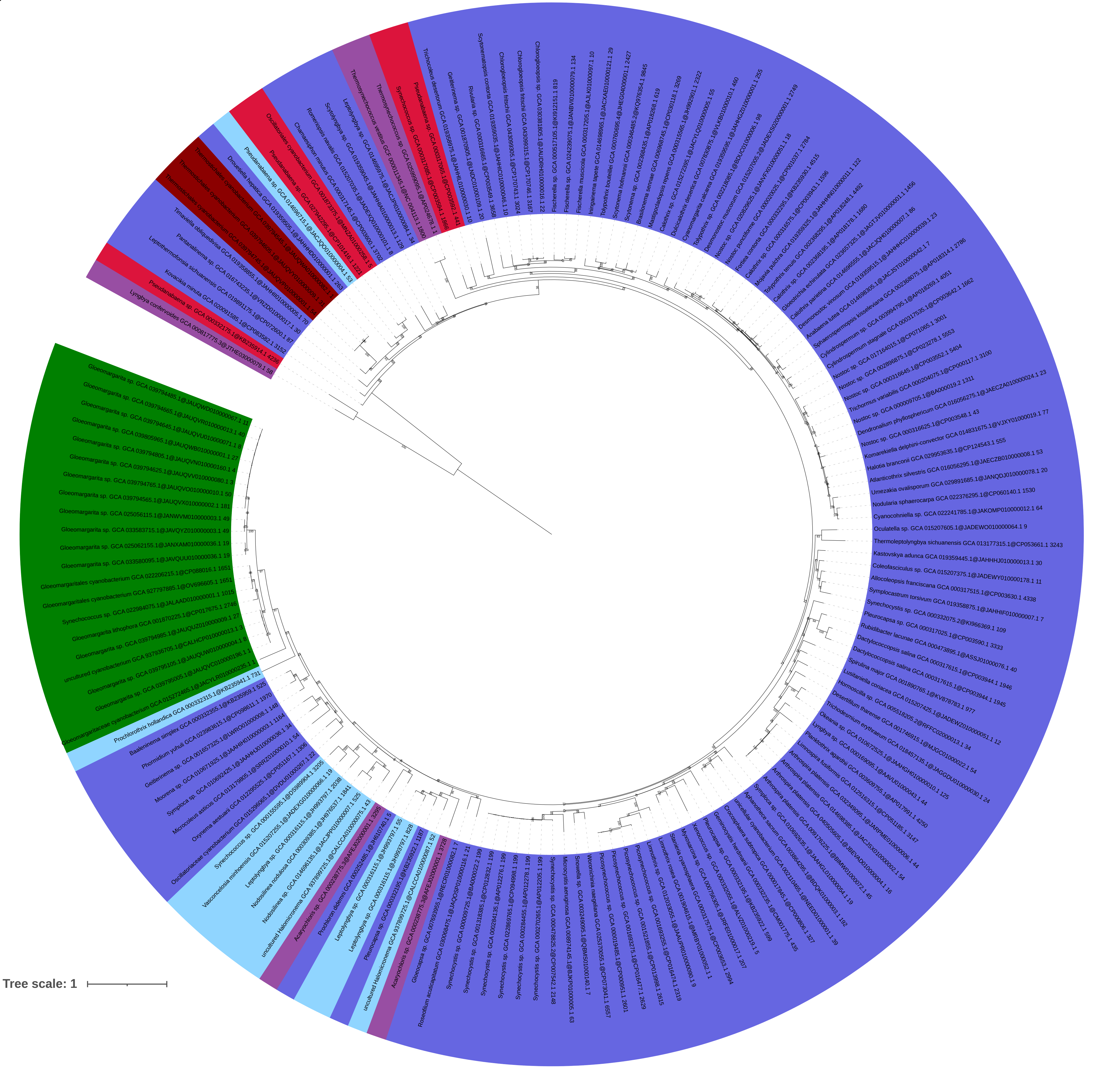

Fig. S130 - Slr1128

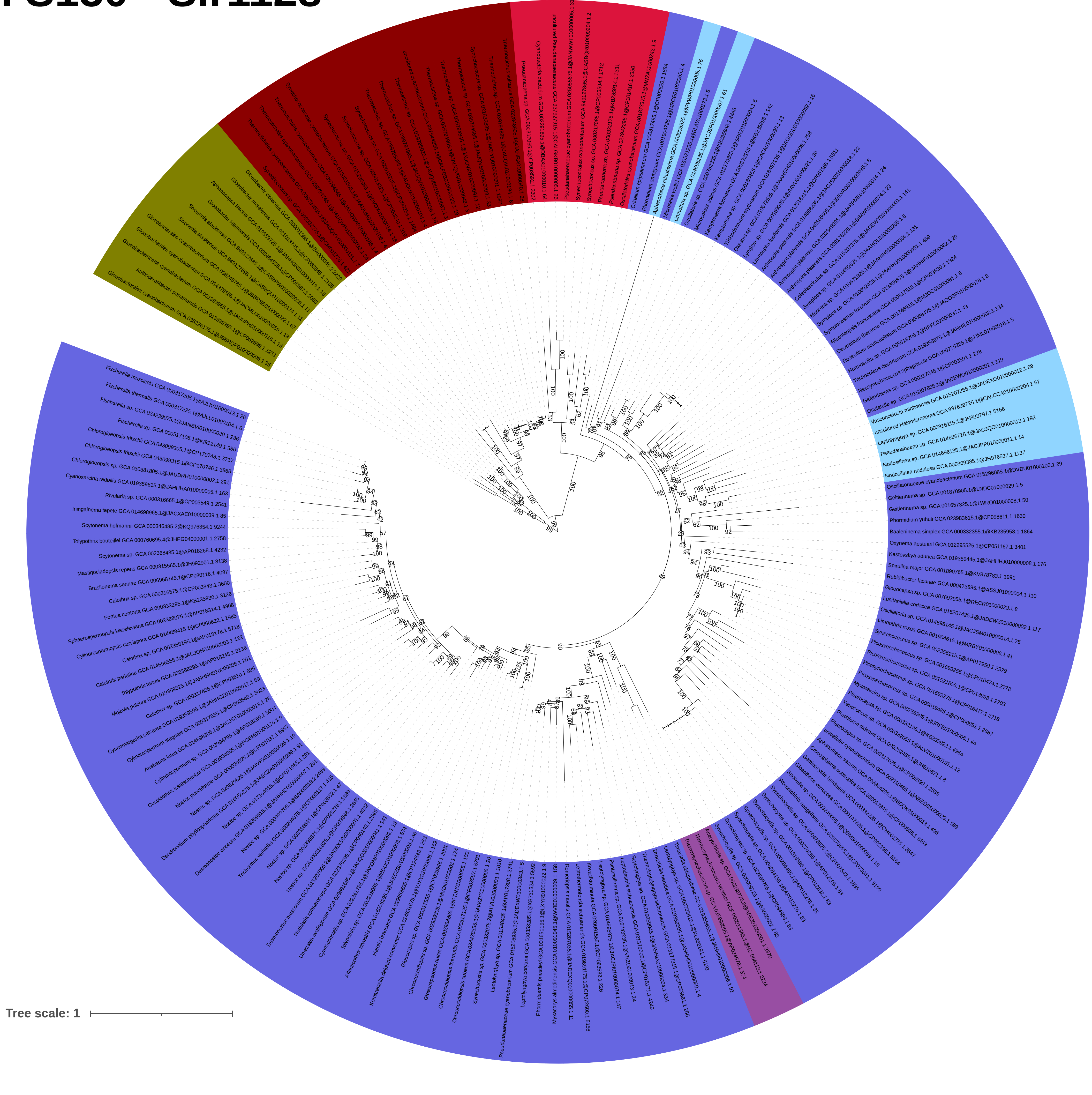

Fig. S131 - Slr1761

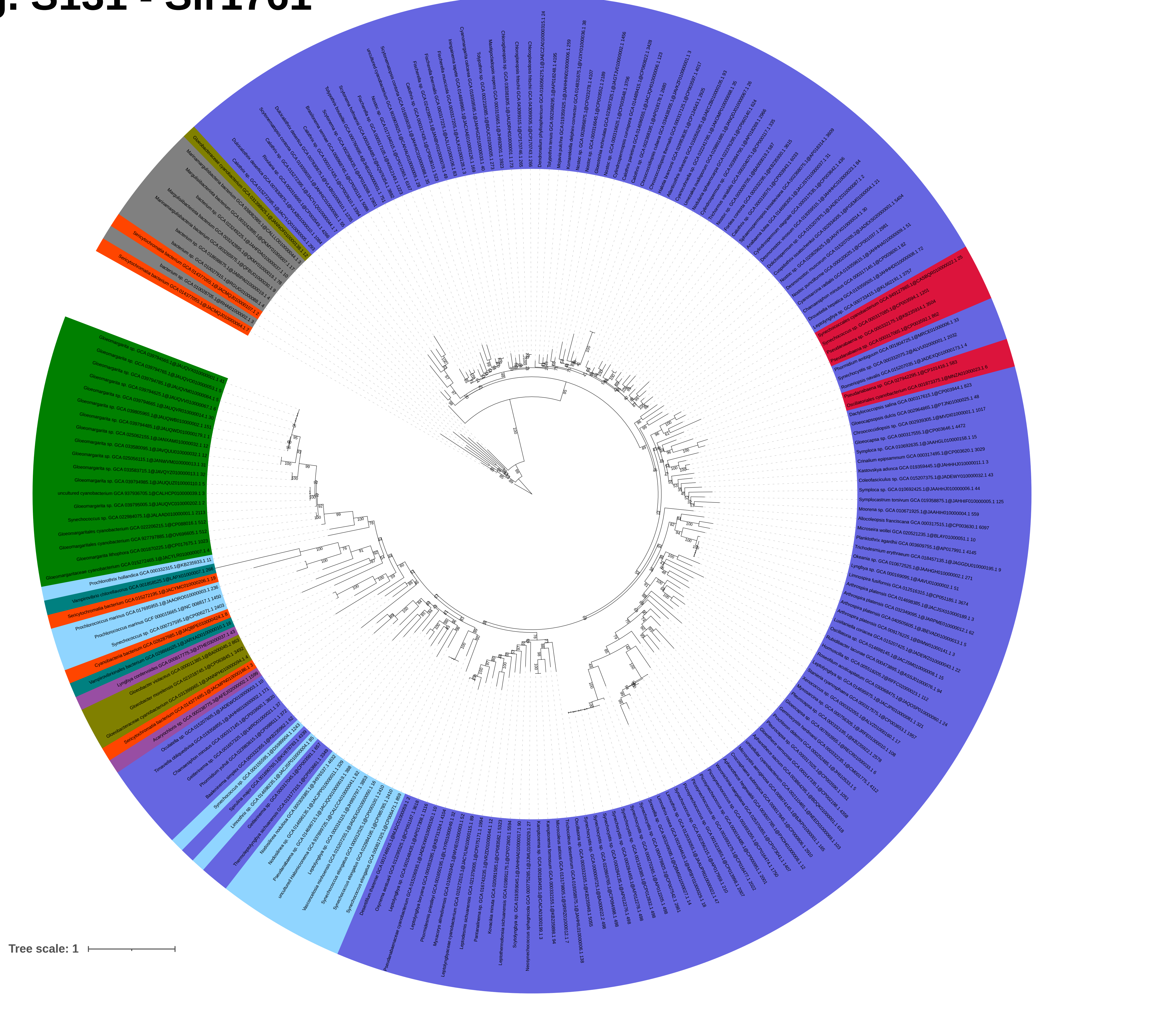

Fig. S132 - Slr1768

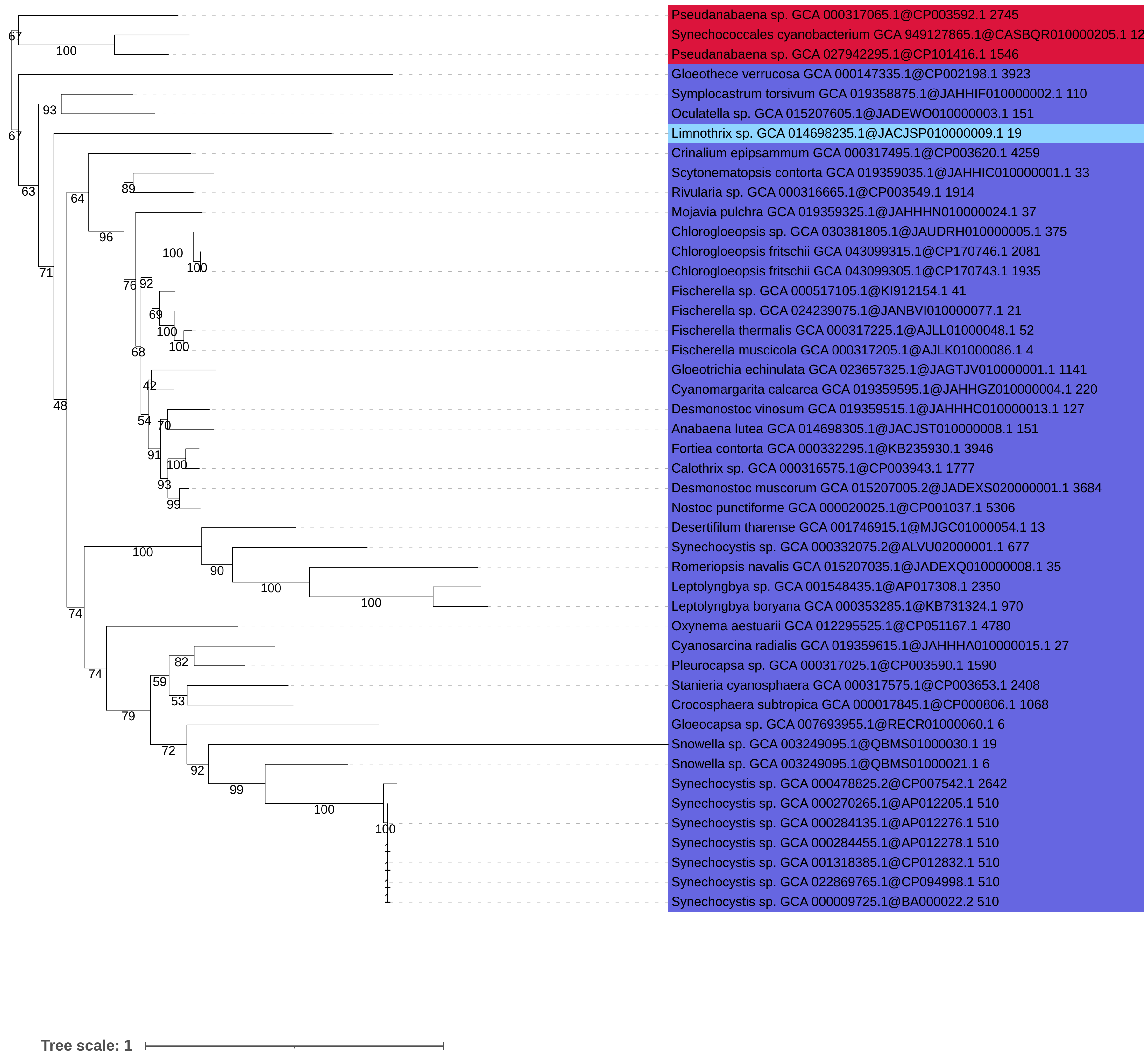

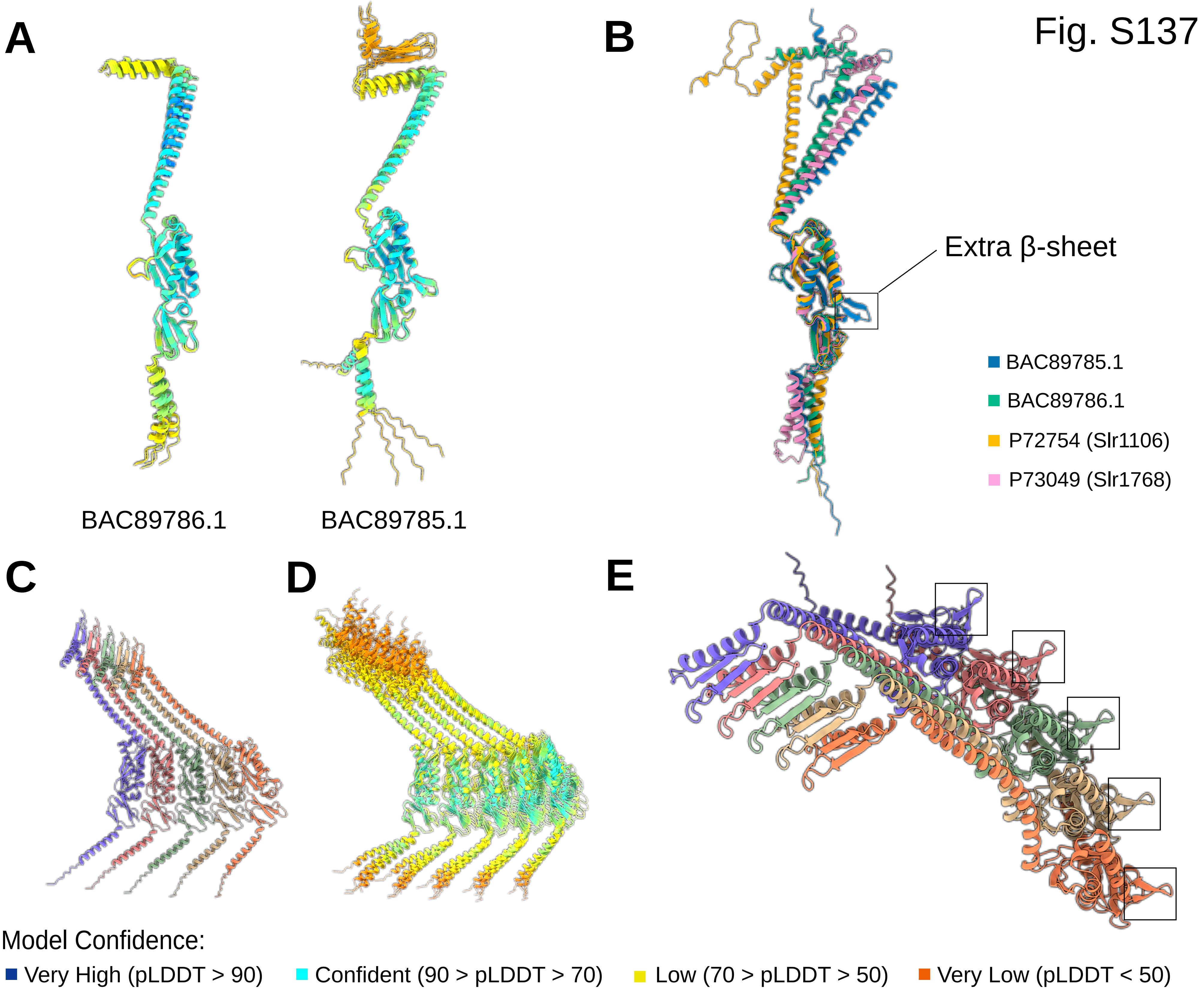
Fig. S137
A
B
BAC89786.1
BAC89785.1
Extra β-sheet
BAC89785.1
BAC89786.1
P72754 (Slr1106)
P73049 (Slr1768)
C
D
E
Model Confidence:
Very High (pLDDT > 90)
Confident (90 > pLDDT > 70)
Low (70 > pLDDT > 50)
Very Low (pLDDT < 50)

Fig. S138

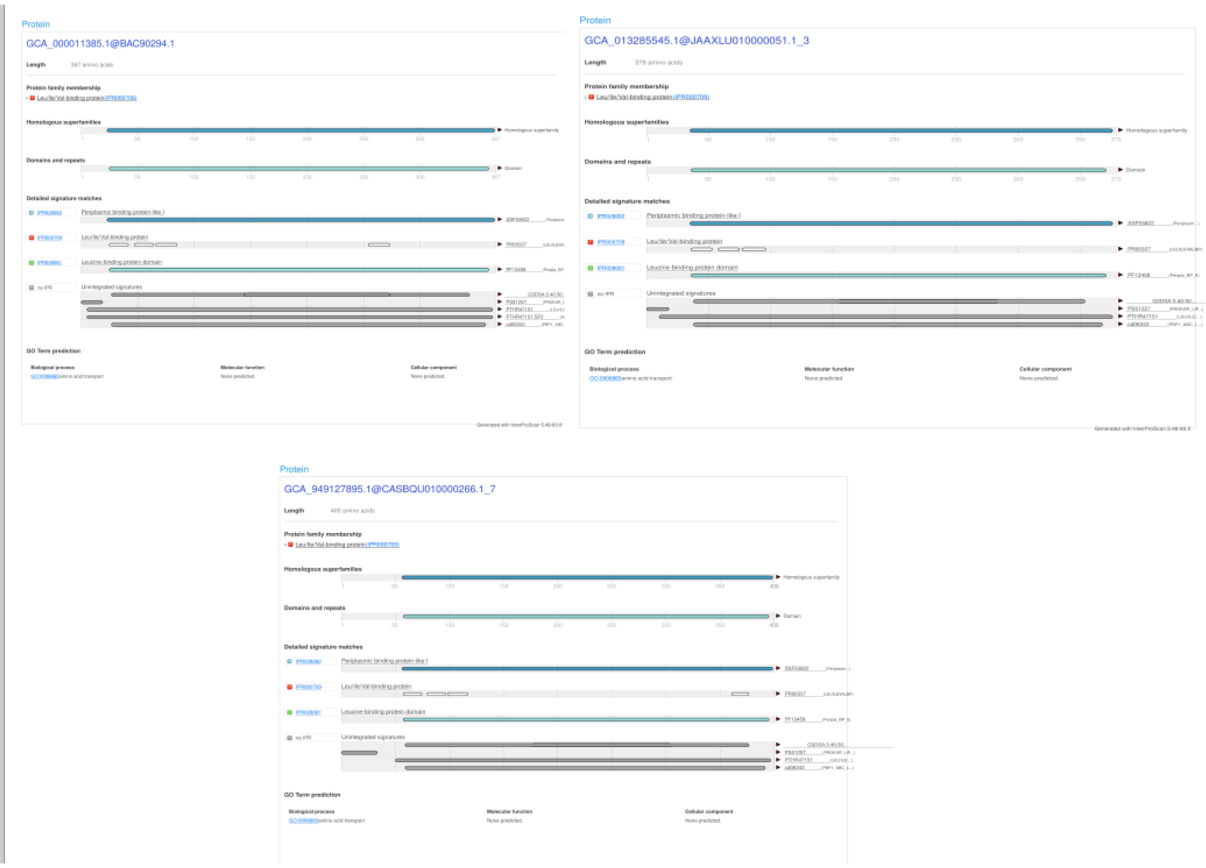

Fig. S139

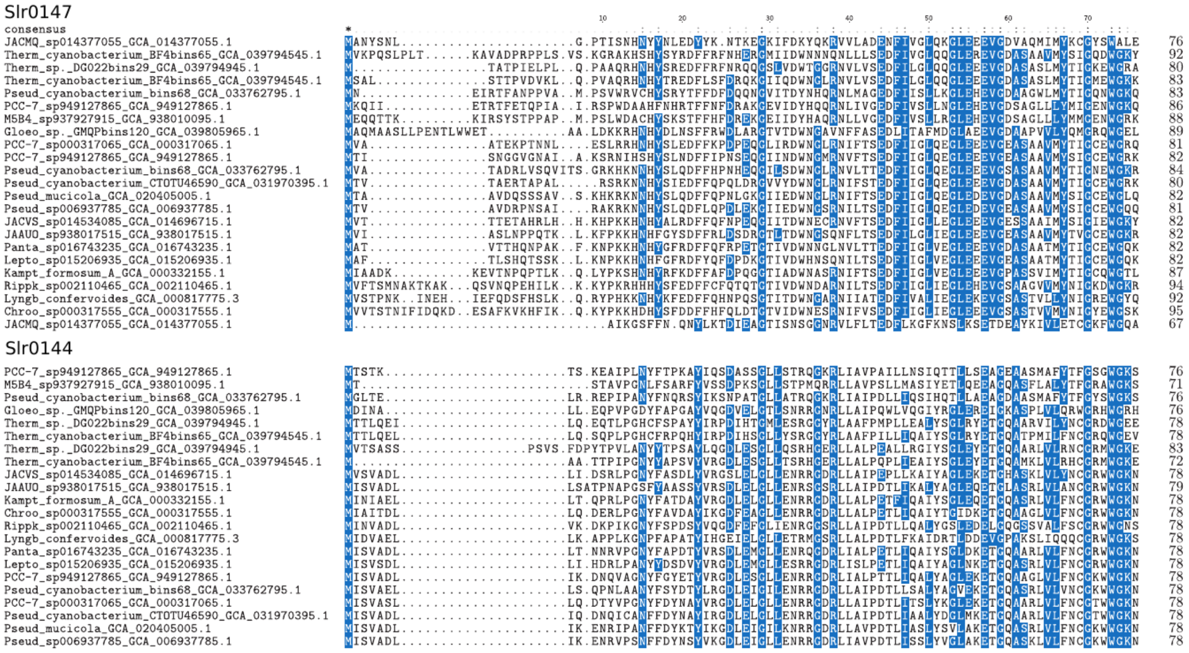

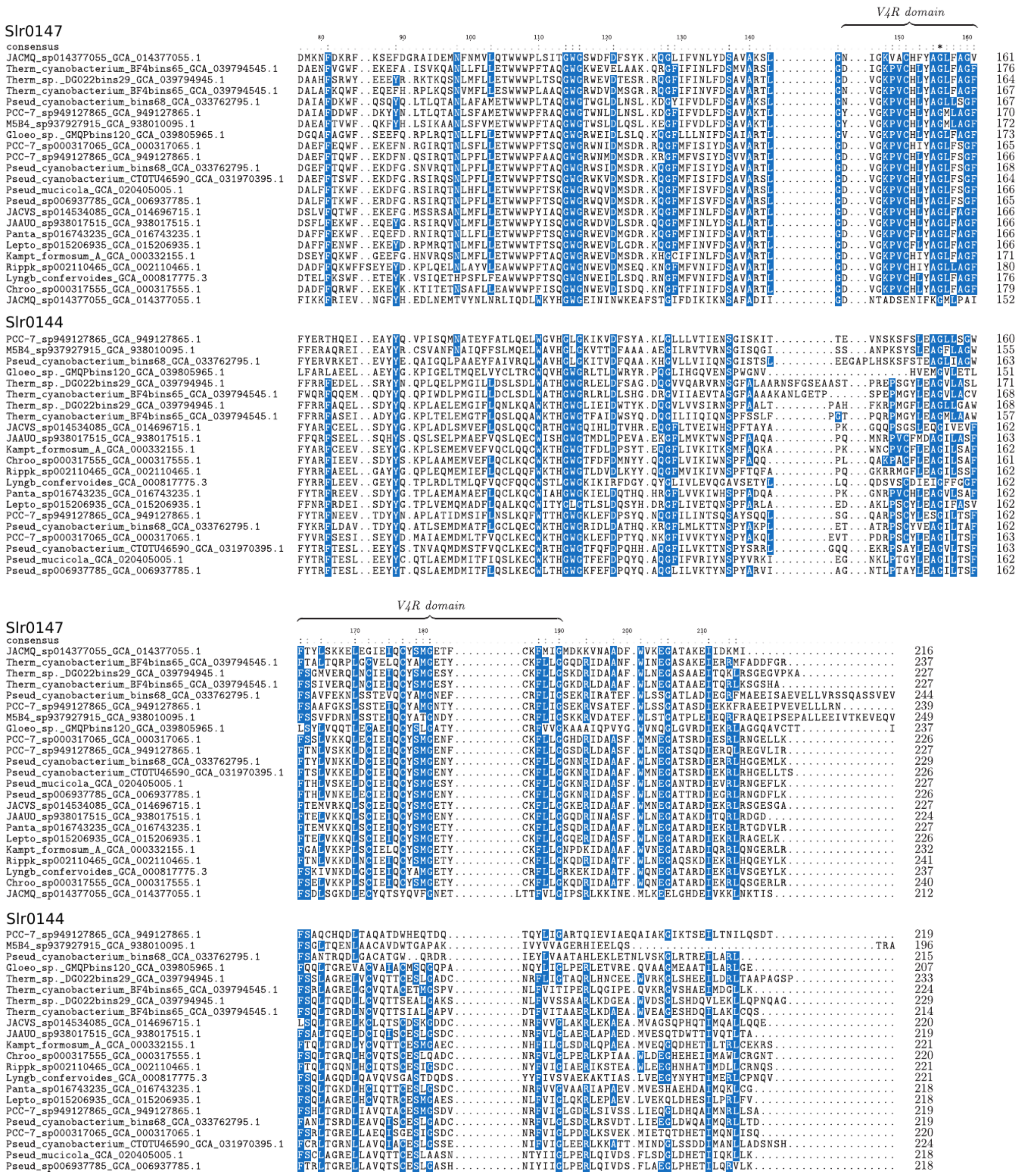

V4R domain
Slr0147
consensus
Slr0144
V4R domain
Slr0147
consensus
Slr0144

Fig. S140

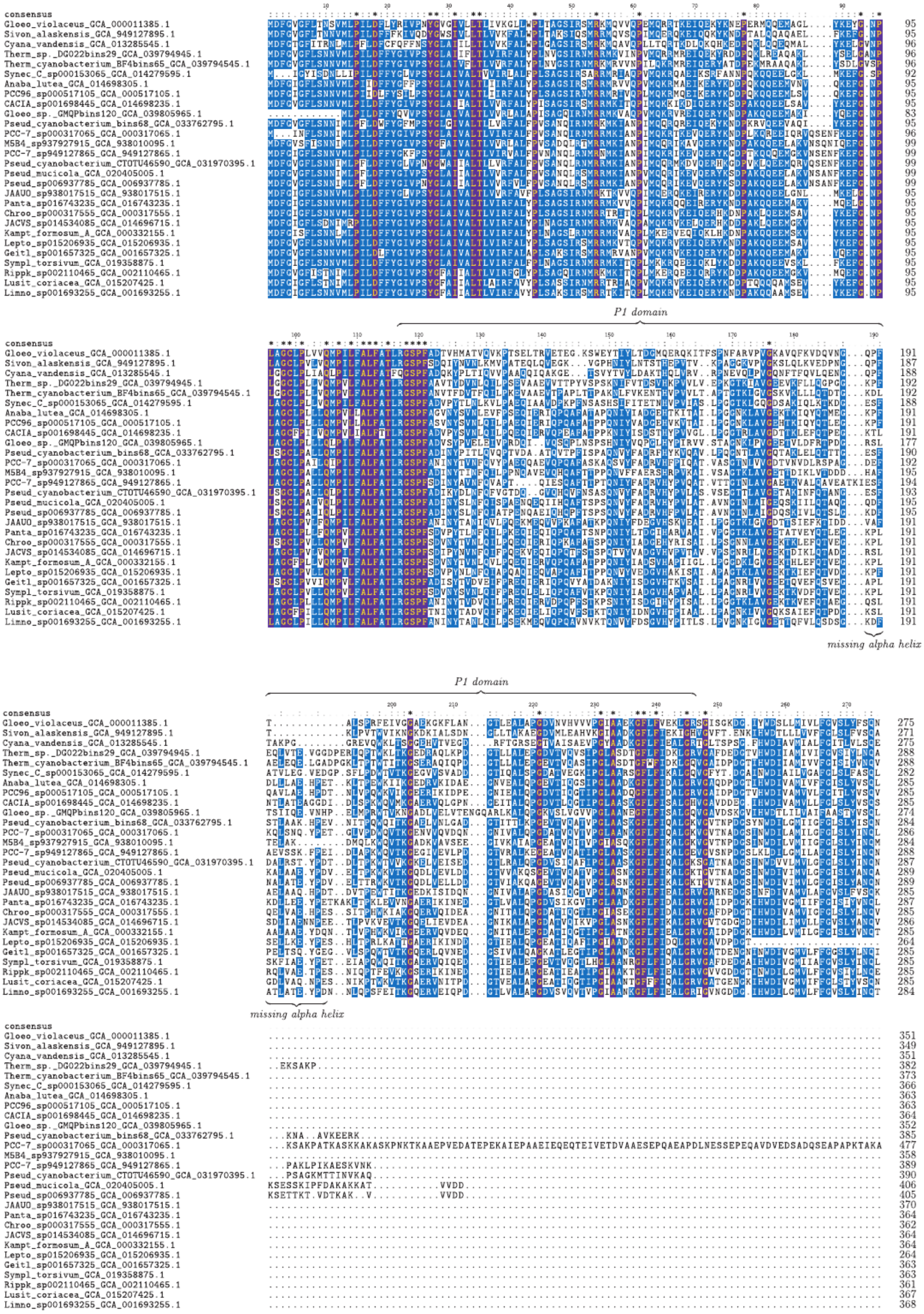

P1 domain
missing alpha helix
P1 domain
missing alpha helix

```
                                                       360
consensus                                              ........................:.............:::::....:::................................
Gloeo_violaceus_GCA_000011385.1                        ..................................TDKPLPFENK.QRRNKKKA.............................  369
Sivon_alaskensis_GCA_949127895.1                       ......................EAG.........TGMEIPFEGN.SRRARKTIDVQ..ATEKGDNSSSNRKQRKDRKKPD..  395
Cyana_vandensis_GCA_013285545.1                        ..................................PKPVLPFEKK.RRKKK................................  366
Therm_sp._DG022bins29_GCA_039794945.1                  .....................KAAGE........GRESLPFEP.......................................  396
Therm_cyanobacterium_BF4bins65_GCA_039794545.1         ..................................ERETLPFEN.......................................  382
Synec_C_sp000153065_GCA_014279595.1                    .......................GGN........PAGRLPFEPR.SK...................................  381
Anaba_lutea_GCA_014698305.1                            .......................SA.........EPKKLPFEPK.SSKKKATG..............................  383
PCC96_sp000517105_GCA_000517105.1                      ...................................QKTLPFEPK.SSKKKAAG..............................  380
CACIA_sp001698445_GCA_014698235.1                      ......................VDG.........ATGRLPFEPK.GTKASSPAKNQ..PSKGPGNKPSTNNNKQKGKS....  408
Gloeo_sp._GMQPbins120_GCA_039805965.1                  ...................................TETLPFERQ.RPKKKPS..............................  368
Pseud_cyanobacterium_bins68_GCA_033762795.1            ..................................AKVDIPFEPG.SAKKKKPQP.............................  404
PCC-7_sp000317065_GCA_000317065.1                      SKATKSAAPKPQKEQKEKPRKPAKGKKAKEEQSEPREPLPFEPGFGKKKKSKSS............................  531
M5B4_sp937927915_GCA_938010095.1                       ...................................KPALDFEPK.SKKKKQT..............................  374
PCC-7_sp949127865_GCA_949127865.1                      .......................NK.........DEVNLPFEPG.NSSKKKKS..............................  409
Pseud_cyanobacterium_CTOTU46590_GCA_031970395.1        .......................DT.........TKESLPFEPN.SKKKKKGSGENS..........................  414
Pseud_mucicola_GCA_020405005.1                         ....................KDKDGK......TPTKSALPFEPN.SNSSKKKKKS............................  434
Pseud_sp006937785_GCA_006937785.1                      ....................KDQDTK......SPTKSALPFEPN.SSNKKKKKGS............................  433
JAAUD_sp938017515_GCA_938017515.1                      ...................................NNRMAFEPK.NPKKEAAKAEAKETTKGGRKKPASKGGKKGKKSSKKS  416
Panta_sp016743235_GCA_016743235.1                      ......................ATG..........KTTLPFEPGNRTKKKAQS.............................  385
Chroo_sp000317555_GCA_000317555.1                      .......................KG..........RESLPFEPG.RSKKKASG.............................  381
JACVS_sp014534085_GCA_014696715.1                      .......................DG..........RQALPFEPGSRSKKKA...............................  382
Kampt_formosum_A_GCA_000332155.1                       .......................KG..........EDALPFEPG.RTKKKA...............................  381
Lepto_sp015206935_GCA_015206935.1                      ..................................................................................  264
Geitl_sp001657325_GCA_001657325.1                      .......................DG..........RQTLPFEPK.RSKKKAG..............................  381
Sympl_torsivum_GCA_019358875.1                         .......................SGE........TREALPFER..RSKKKASS.............................  383
Rippk_sp002110465_GCA_002110465.1                      ...................................QEALPFEKK.RSKKKEKTS............................  379
Lusit_coriacea_GCA_015207425.1                         .......................EG..........RDKLPFERK.RSKKKEKTSG...........................  388
Limno_sp001693255_GCA_001693255.1                      ........................G..........RDKLPFERK.GSKKKEKTSG...........................  388
```

Fig. S141

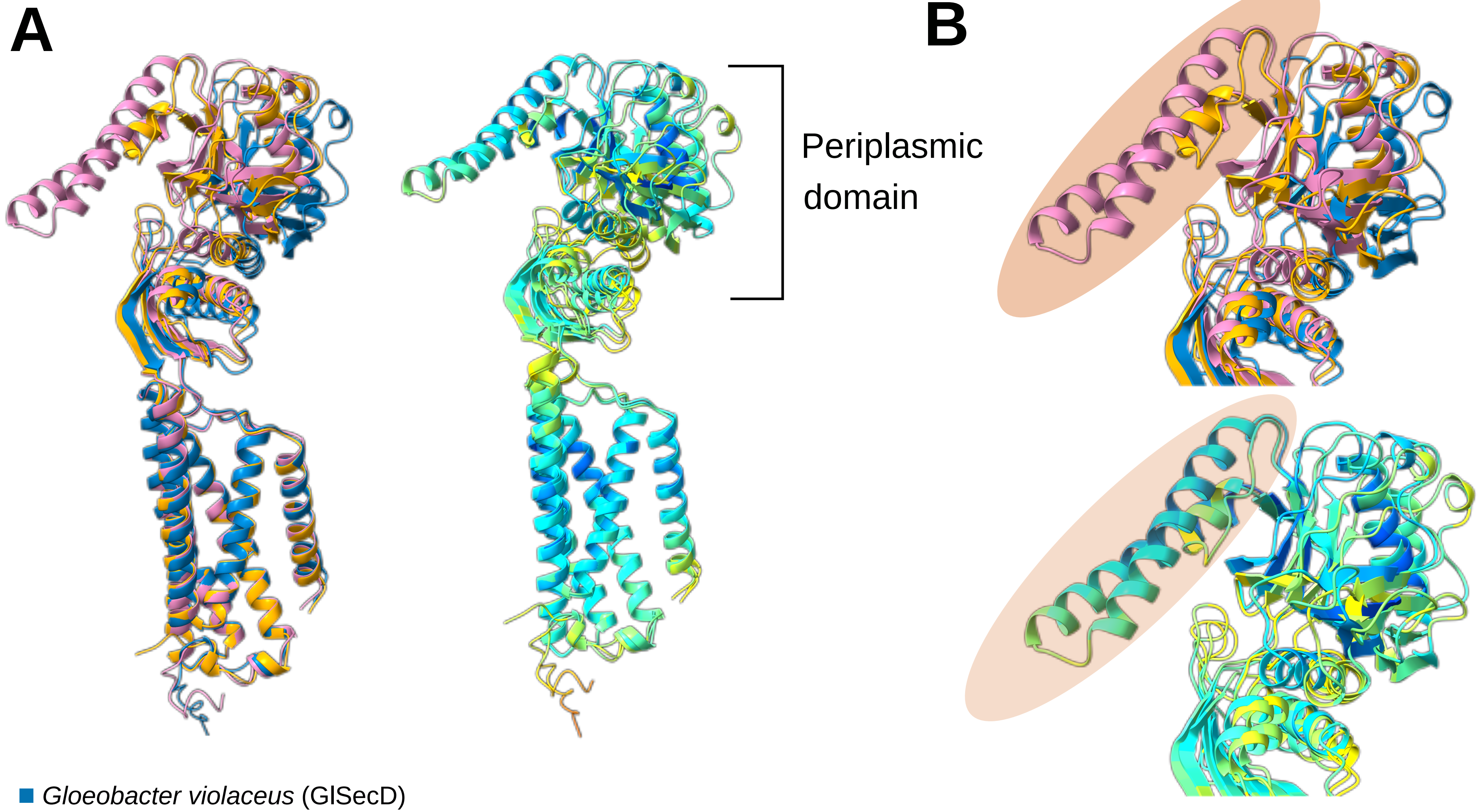

■ *Gloeobacter violaceus* (GlSecD)

■ Thermostichales cyanobacterium GCA_039794545.1 (TSecD)

■ *Synechocystis* sp. PCC6803 (SynSecD)

Model Confidence:

■ Very High (pLDDT > 90) ■ Confident (90 > pLDDT > 70) ■ Low (70 > pLDDT > 50) ■ Very Low (pLDDT < 50)

Fig. S142

■ *Gloeobacter violaceus* (GlSecF)

■ Thermostichales cyanobacterium GCA_039794545.1 (TSecF)

■ *Synechocystis* sp. PCC6803 (SynSecF)

B

A

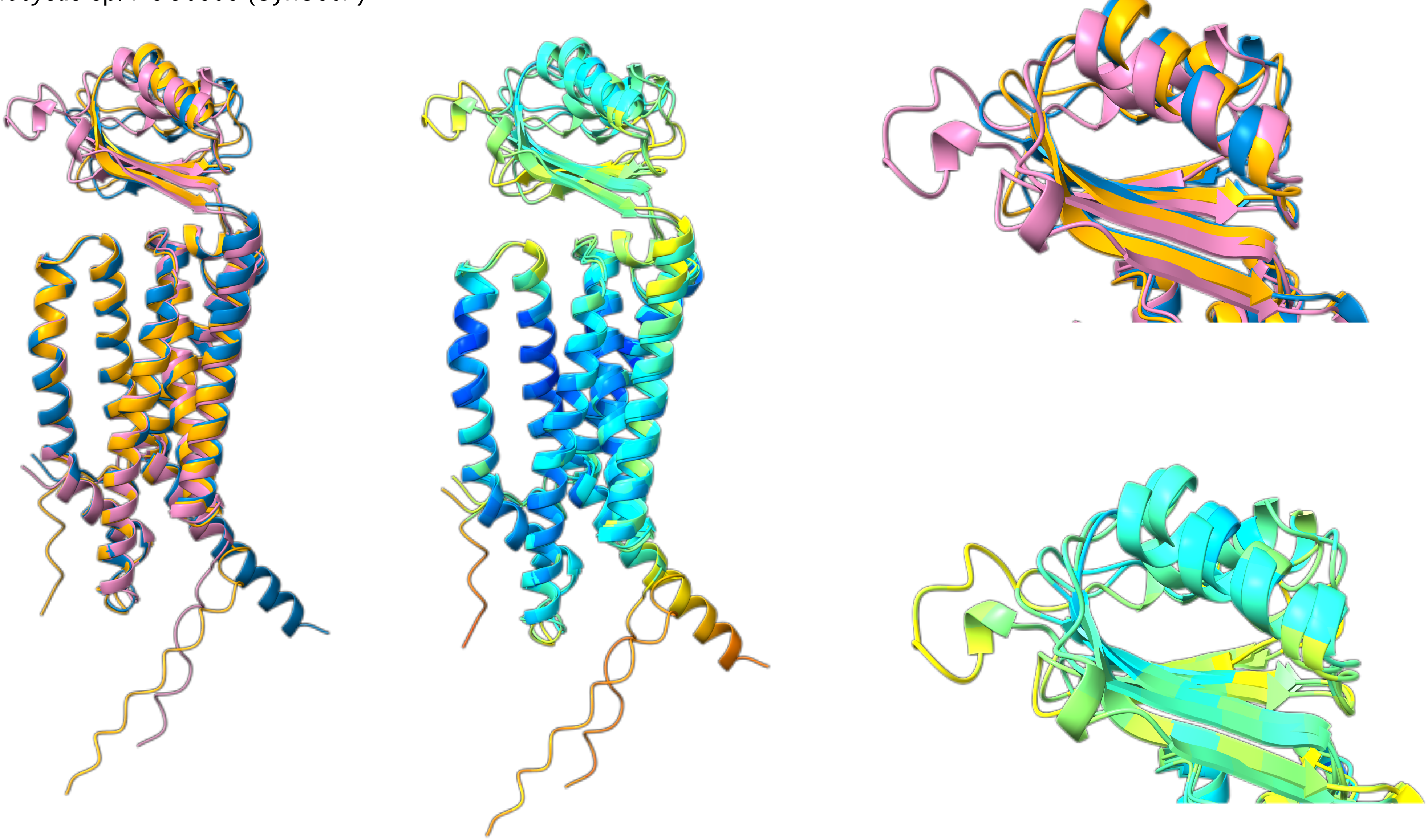

Model Confidence:

■ Very High (pLDDT > 90) ■ Confident (90 > pLDDT > 70) ■ Low (70 > pLDDT > 50) ■ Very Low (pLDDT < 50)

Fig. S143

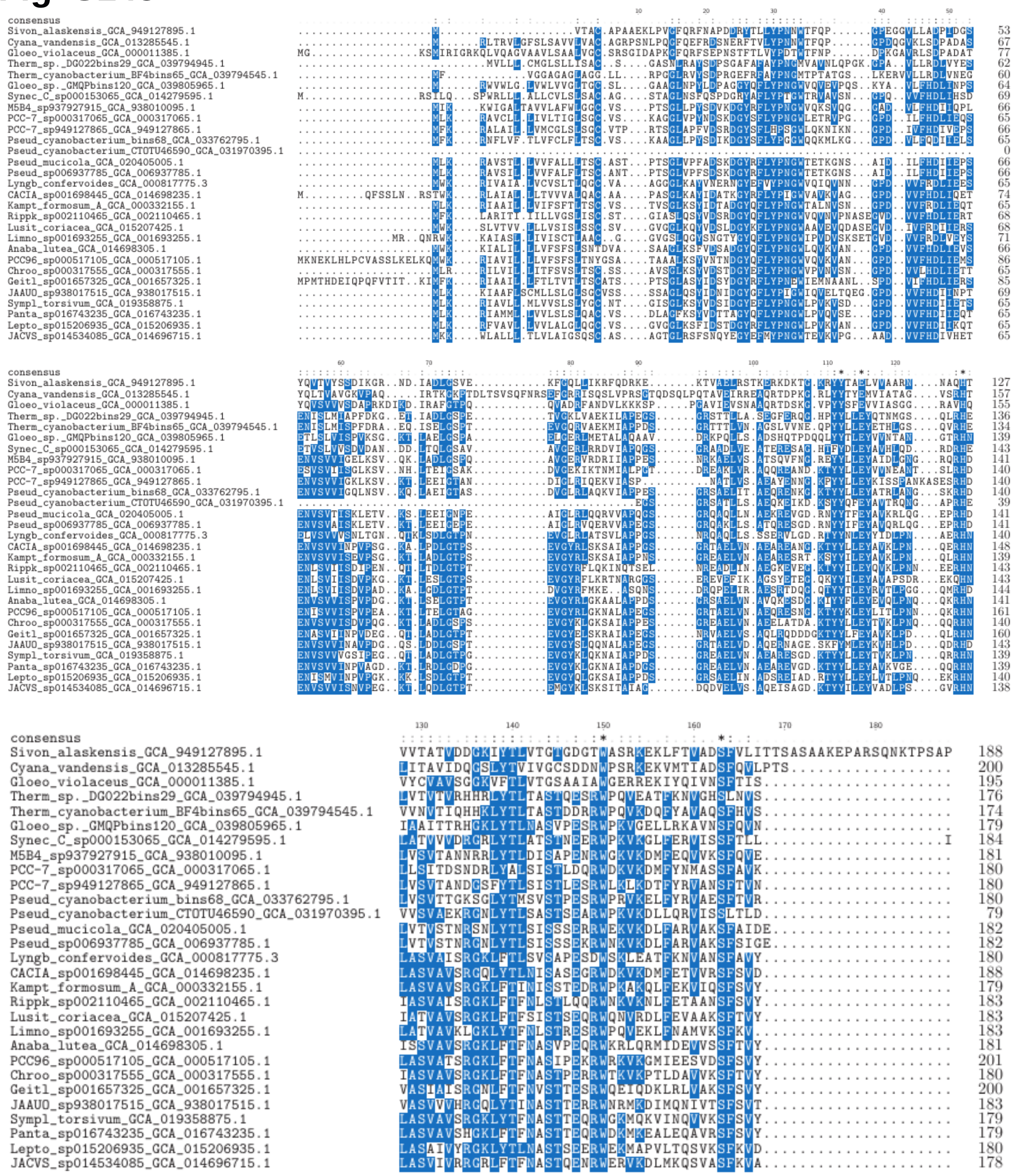

Fig. S144

A

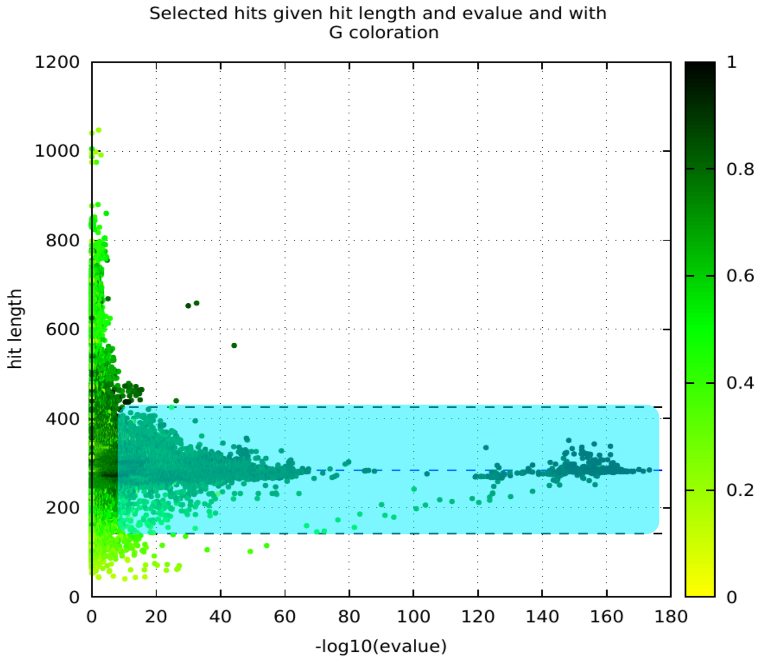

B

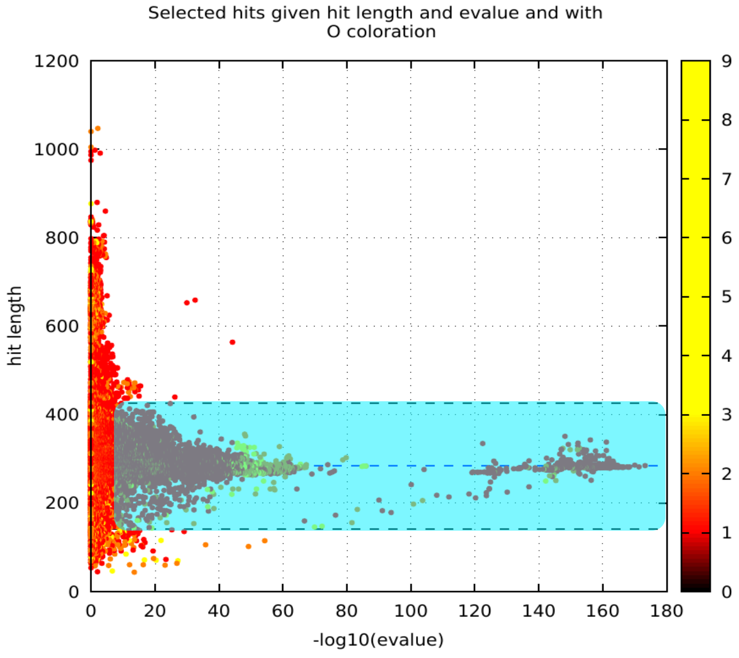

Fig. S145

A

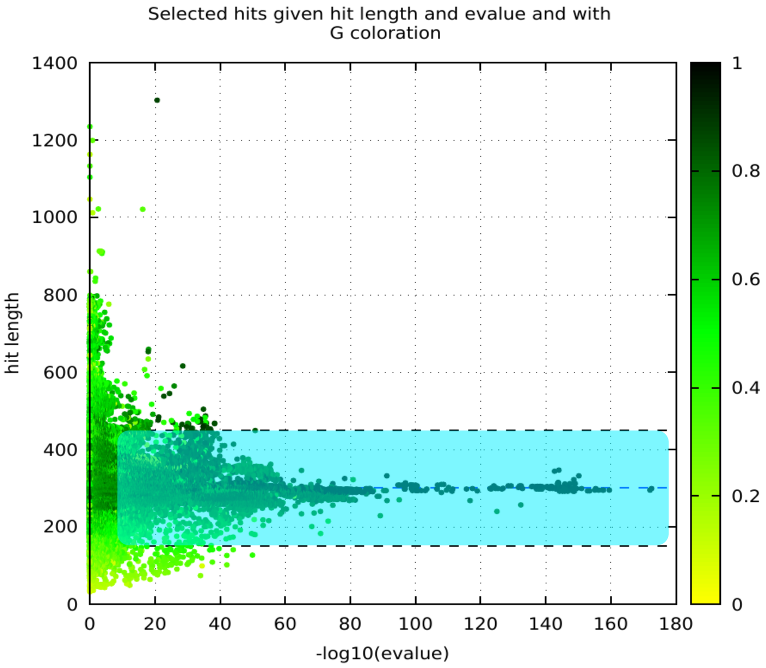

B

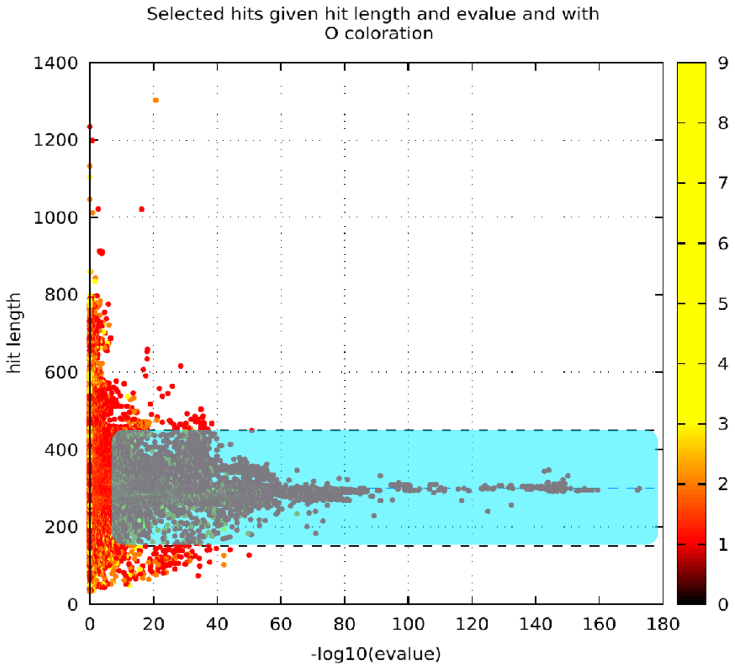

Fig. S146

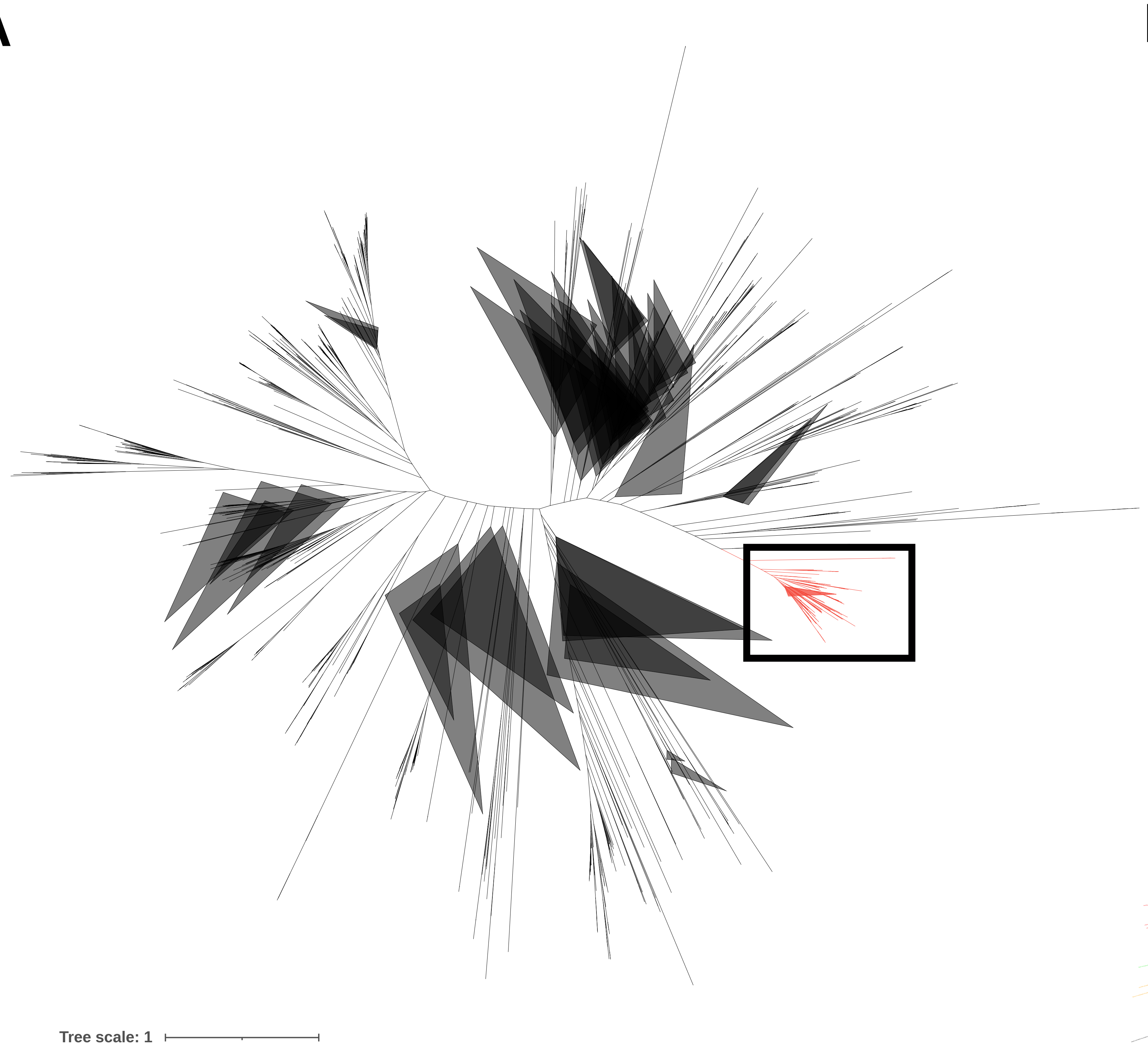
A
Tree scale: 1

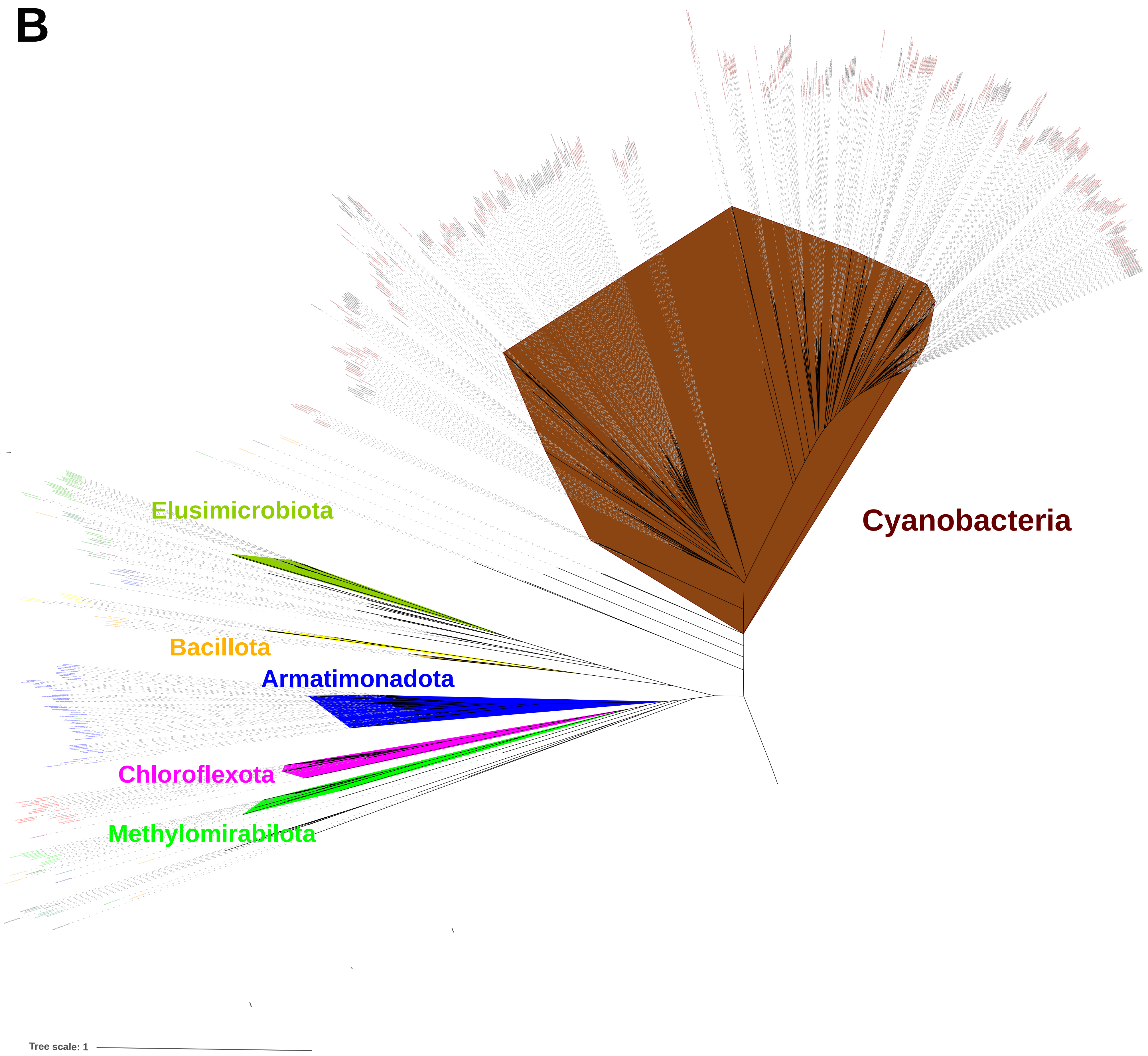
B
Elusimicrobiota
Bacillota
Armatimonadota
Chloroflexota
Methylomirabilota
Cyanobacteria
Tree scale: 1

Fig. S147

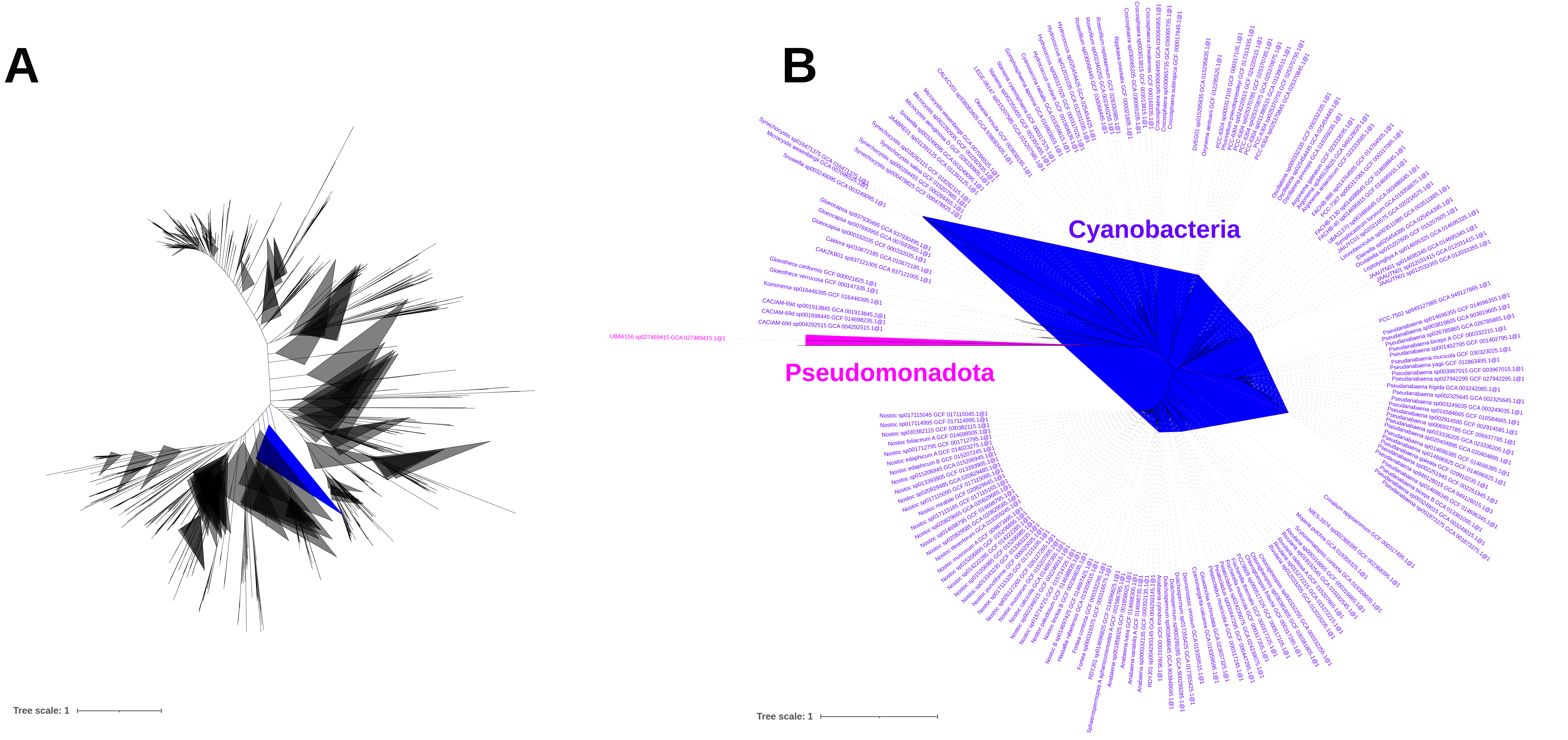

Supplement: Supplementary file 1 — Fig. S1 Phylogenomic tree based on ribosomal proteins. Fig. S2 Genome completeness by Terrabacteria phylum using CheckM2. Fig. S3 Genome contamination by Terrabacteria phylum using CheckM2. Fig. S4 Diversity curve generated using parnas v.0.1.6 for the pruning of the ribosomal tree. Figs. S5–S50 Boxplots based on the bitscore distributions of blastp results. Figs. S51–S95 Gene trees of each OG of interest. Figs. S96–S136 Gene trees based on the best hits of a HMM search on the Cyanobacterial dataset (343 Cyanobacteriota species). Fig. S137 Additional β‐sheet positioned outside of the ring structure formed during oligomerization in the proteins of the basal group I. Fig. S138 Interpro predictions of functional domains within the closest homologs of Sll0606 in Gloeobacterales. Fig. S139 Alignment of the cyanobacterial Slr0144 and Slr0147 sequences. Fig. S140 Alignment of the cyanobacterial YidC sequences. Fig. S141 AlphaFold3 predicted 3D structures of SecD from Gloeobacter violaceus, Thermostichales cyanobacterium GCA_039794545.1, Synechocystis sp. PCC 6803. Fig. S142 AlphaFold3 predicted 3D structures of SecF from Gloeobacter violaceus, Thermostichales cyanobacterium GCA_039794545.1, and Synechocystis sp. PCC 6803. Fig. S143 Alignment of the cyanobacterial CyanoP sequences. Fig. S144 Visualization of the selection of the sequences similar to Slr1106 with Ompa‐Pa. Fig. S145 Visualization of the selection of the sequences similar to Slr1768 with Ompa‐Pa. Fig. S146 Preliminary multigenic family tree of Slr1106. Fig. S147 Preliminary multigenic family tree of Slr1768. [file NPH-251-1329-s003.pdf]
