# Supplementary material for: Exploring thylakoid emergence: evolution of membrane biogenesis and photosystem II assembly in early‐diverging cyanobacteria
Source: New Phytol. 2026 Jun 5;251(3):1329–43. doi: 10.1111/nph.71284 (PMC13326532; doi:10.1111/nph.71284)
Supplement: Supplementary file 2 [file NPH-251-1329-s002.pdf]

New Phytologist Supporting Information
Article title: Exploring Thylakoid Emergence: Evolution of Membrane Biogenesis and Photosystem II assembly in early-diverging Cyanobacteria
Authors: Louise Hambücken, Denis Baurain, Luc Cornet
Article acceptance date: 27 April 2026

Supplementary Tables

Genome selection and phylogenomic species tree

Supplemental Table S1 (Excel sheets): Manual selection of the Terrabacteria representative species recovered from GTDB r220 (Parks et al., 2022). Only the 43,827 genomes passing the GUNC contamination filter were retained. Within each GTDB order, genomes were ranked based on the difference between CheckM2 completeness and contamination scores. For orders represented by a single genus, the two highest-quality genomes were selected, whereas for all other orders, the top-ranked genome from each genus was retained. This procedure yielded a total of 1,452 genomes, excluding members of the phylum Cyanobacteriota.

Supplemental Table S2 (Excel sheets): Selection of representative Cyanobacteriota genomes to complement the manual selection described in Table S1. One or two genomes were selected per genus, prioritizing those with >80% completeness and <10% contamination, as estimated using CheckM2. When multiple genomes met these criteria, the highest-quality representatives were retained for downstream analyses.

Supplemental Table S3 (Excel sheets): Sources of proteomes for the manually-selected accessions. Proteomes were obtained either from NCBI RefSeq/GenBank annotations or predicted de novo using Prodigal v2.6.3 (Hyatt et al., 2010).

Supplemental Table S4 (Excel sheets): Selection of 950 genomes based on manual filtering of Tables S1 and S2, combined with ribosomal phylogenetic tree pruning using PARNA (Markin et al., 2023). A ribosomal phylogeny was used to guide genome subsampling, enabling the selection of a reduced, non-redundant dataset that maximizes phylogenetic diversity. The resulting set of 950 genomes was retained for downstream analyses.

Orthologous gene tree inference

Supplemental Table S5 (This file): Description of selected assembly factors for this study. Proteins were compiled from multiple literature sources and comprise factors implicated in Photosystem II (PSII) assembly, covering both early and late stages of the assembly pathway.

Supplemental Table S6 (This file): Description of selected proteins involved in cyanobacterial membrane dynamics from (Siebenaller & Schneider, 2023). Proteins were compiled from Siebenaller and Schneider (2023) and include proteins mplicated in membrane remodeling and organization.

Supplemental Table S7 (This file): Summary of the BLASTp search and the phylogenetic trees. Asterisks in the *Gloeobacter violaceus* PCC 7421 ortholog column means that this gene is present in *Gloeobacterales* but not in *Gloeobacter violaceus* PCC 7421.

Supplemental Table S8 (This file): Identification of homologs in closely related taxa. Identification of homologs in closely related taxa (Vampirovibrioniae, Sericytochromatia, and Margulisbacteria) based on HMM searches (Finn et al., 2011) performed across representative genomes from the GTDB (Parks et al., 2022). Highlighted proteins correspond to those for which no clear homologs were detected in any of these three lineages.

Supplemental Table S5.

Category	Cyanobacterial gene of PSII assembly factors	Gene product name	Localization	Protein function	Target ligand or PSII subunits	Other interactions	Closest homolog in Arabidopsis thaliana	References
precursor of the D19 pt protein (pD1) insertion	slr1471	YidC*	Intrinsic, PDM, TM	Membrane insertase	pD1	Sec apparatus, ChlG-HliD complex, Ycf39, ribosome subunits	AT2G28800 (Alb3)	(Chidgey et al., 2014a; Gathmann, Sven et al., 2008; Proctor et al., 2018)
	sll1814	SecY*	Intrinsic, CM, TM	Protein translocase subunit of the SecYEG channel	pD1	Other proteins of the Sec apparatus, ribosome subunits, YidC	AT2G18710	(Frain et al., 2016)
	ssl3335	SecE	Intrinsic	Protein translocase subunit of the SecYEG channel	pD1		AT4G14870	(Frain et al., 2016)
	NON20_19535	SecG	Intrinsic	Protein translocase subunit of the SecYEG channel	pD1		-	(Frain et al., 2016)
	slr0774	SecD	Intrinsic	Protein translocase subunit interacting with SecYEG channel	pD1		-	(Frain et al., 2016)
	slr0775	SecF	Intrinsic	Protein translocase subunit interacting with SecYEG channel	pD1		-	(Frain et al., 2016)
	sll0616	SecA	Intrinsic, CM (cytosolic side), TM (cytosolic side)	Protein translocase subunit with ATPase activity interacting with SecYEG channel	pD1		AT4G01800, AT1G21650	(Frain et al., 2016)
Chlorophyll-binding molecule	slr0144	Slr0144	Intrinsic, TM	Putative chlorophyll-binding V4R domain	Chlorophyll (putative)	-	-	(Addlesee et al., 2000; Wegener et al., 2008)
Chlorophyll incorporation into pD1	slr0056	ChlG*	Intrinsic	Terminal enzyme of the Chl biosynthesis pathway	Chlorophyll	Ycf39, HliD	AT3G51820	(Proctor et al., 2018)
	slr0399	Ycf39*	Intrinsic	Incorporation of the chlorophyll into pD1	Chlorophyll precursors	HliD, pD1	AT4G35250 (HCF244)	(Chidgey et al., 2014b; Linhartová et al., 2014; Sachelaru et al., 2013; Wysocka, 2025)
	ssl1633	HliC	Intrinsic	Incorporation of the chlorophyll into pD1	pD1	HliD, Ycf39, YidC, ChlG, chlorophyll	CAB/HLIP/ELIP family	(Komenda & Sobotka, 2016)
	ssr1789	HliD*	Intrinsic	Incorporation of the	Ycf39	HliC, Ycf39, YidC, ChlG,		(Konert, 2022)

				chlorophyll into pD1		chlorophyll, β-carotene		
	slr1644	Pitt	Intrinsic, PDM, TM	Tetratricopeptide Repeat Protein Involved in Light-Dependent Chlorophyll Biosynthesis	POR	-	AT1G78915 (TPR1)	(Schottkowski et al., 2009)
**Manganese-preloading interaction with pD1**	slr2048	PratA*	Extrinsic, PDM, PP	Manganese pre-loading in pD1	pD1, Mn2+	CtpA	-	(Singh et al., 2004)
**Cleavage of the C-terminal extension of pD1**	slr0008	CtpA*	Extrinsic, PDM	Peptidase cleaving the C-terminal extension of pD1	pD1	Associated with the PDM region	AT4G17740	(Anbudurai et al., 1994; Karnauchov et al., 1997)
**D2 folding**	slr0286	Slr0286	Intrinsic, TM	Assembly and stability of the water-splitting system of photosystem II.	D2	-	-	(Kufryk & Vermaas, 2001)
	slr2013	Slr2013	Intrinsic	Folding of the D2 protein	D2	-	-	(Kufryk & Vermaas, 2003)
**RC complex formation**	slr2034	Ycf48*	Extrinsic, L, PDM, TM	Stabilization of newly synthesized pD1, involved in PSII damage repair	pD1, iD1	Sll0933	AT5G23120 (HCF136)	(Kiss et al., 2019; Komenda et al., 2008; Rengstl et al., 2013)
	slr0008	CtpA*	Extrinsic, PDM	Peptidase cleaving the C-terminal extension of iD1	iD1	Associated with the PDM region	AT4G17740	(Anbudurai et al., 1994; Karnauchov et al., 1997)
	sll0408	Sll0408	Extrinsic, L	Peptidyl-prolyl cis-trans isomerase	CP47	-	AT3G01480 (CYP38, TLP40)	(Fu et al., 2007; Sirpiö et al., 2008)
	D082_23770	RubA	Intrinsic, TM	Rubdredoxin-line protein required for D1 and D2 association	D1, RC47-PSI	RC47-PSI complex	AT1G54500 (RBD1)	(Kiss et al., 2019; Šesták, 2007; Shen et al., 2002)

**RC47 intermediate formation**	sll1398	Psb28*	Intrinsic, CM, TM	Biogenesis of certain Chl-binding proteins	CP47 antennae, CP47	-	AT4G28660 (PsbW)	(Bečková et al., 2017; Dobáková et al., 2009; Kashino et al., 2007; Sakata et al., 2013)
	sll0933	Sll0933*	Intrinsic, TM	RC47 and PSII core formation	CP43, CP47	Ycf48	AT4G19100 (Pam68)	(Armbruster et al., 2010; Bučinská et al., 2018; Rengstl et al., 2011, 2013)
	ssl2542	HliA	Intrinsic	Associated with later PSII assembly intermediate	CP47 antenna	HliC, chlorophyll, β-carotene	CAB/HLIP/ELI P family	(Akulinkina et al., 2015; Konert, 2022)
	ssr2595	HliB	Intrinsic		CP47 antenna	HliC, chlorophyll, β-carotene	-	(Akulinkina et al., 2015; Konert, 2022)
	ssl2148	Psb35	Putative intrinsic	Involved in in the life cycle of cyanobacterial Chl-binding proteins, especially CP47	CP47 antenna, PSI trimer	-	-	(Pascual-Aznar et al., 2021)
**PSII lacking manganese cluster formation**	sll0933	Sll0933*	Intrinsic, TM	RC47 and PSII core formation	CP47 antennae	Ycf48	AT4G19100 (Pam68)	(Armbruster et al., 2010; Rengstl et al., 2011, 2013)
	slr1645	Psb27*	Extrinsic, L	CP43 binding for assembly and Cluster oxygen-evolving complex assembly	CP43 antennae, D1	-	AT1G03600	(Komenda et al., 2012; Nowaczyk, 2014; Roose et al., 2007)
	sll0606	Sll0606*	Intrinsic, CM or extrinsic, PP	Hypothetical transport of hydrophobic components;Insertion of the CP43 module into RC47	CP43 antennae	-	-	(Komenda et al., 2008; Zhang et al., 2010)
	ssl1498	Psb34	Intrinsic	Lipoprotein involved in PSII late-assembly (competes with HliA/B for the same binding site)	CP47 assembled in PSII	-	-	(Rahimzadeh-Karvansara et al., 2022)
**photoactivation of the oxygen-evolving manganese cluster**	sll1418	CyanoP (PsbP-like)*	Extrinsic, Lumen	Assembly of PSII intermediate, putative role in OEC assembly	D1, oxygen-evolving cluster	Divalent ions	AT3G55330 (PPL1)	(Knoppová et al., 2016; Nowaczyk, 2014)
	slr0565	Slr0565	Extrinsic, L	Catalyze of disulfide bond formation in lumenal and lumen-exposed proteins	PsbO	-	AT4G35760 (LTO1)	(Karamoko et al., 2011)
**Supercompl**	slr1761	Slr1761	Extrinsic, L	Fkbp-type Peptidyl-	PSII	Trx	AT3G60307 (FKBP-20-2)	(Lima et al., 2006)

**ex assembly**				prolylcis-transisomerase involved in PSII supercomplex assembly				
**Protection from oxidative stress and photodamage**	sll1390	Psb32	Intrinsic, TM	PSII protection from photodamage and repair acceleration	PSII lacking manganese cluster	-	AT1G54780 (TLP18.3)	(Wegener et al., 2011)
**PSII repair**	sll1414	Psb29	Intrinsic, CM, TM	PSII repair (D1 degradation)	D1 ?	FtsH complex	AT2G20890 (THF1)	(Keren et al., 2005)
	slr0151	Slr0151	Intrinsic, TM	PSII repair under high-light conditions	CP43 antennae, D1	-	-	(Yang et al., 2014)
	smr0009	PsbN	Intrinsic, TM (stromal side)	Bitopic trans-membrane peptide required for PSII repair and PSII RC assembly	?	?	ATCG00700	(Plöchinger et al., 2016; Torabi et al., 2014)

Supplemental Table S6.

**Category**	**Cyanobacterial gene of PSII assembly factors**	**Eukaryotic homolog**	**Protein function**
DedA proteins	Slr0232	Tvp38	Vesicular trafficking, putative lipid scramblase
	Slr0305		
	Sll0509		
SPFH proteins	Slr1106	Prohibitin	Stabilization and dynamics of mitochondrial membrane, associated with lipid rafts
	Slr1768		
	Sll1021	Flotilin	Associated with lipid rafts
	Slr1128	Stomatin	Ion channel regulation, associated with lipid rafts
	Sll0815		

Cytoskeletal proteins	Sll1633 (FtsZ)	Tubulin	Microtubule formation
Membrane-bending proteins	Slr0483	Arabidopsis CURT1	Induction of curvature in TM
Dynamin-like proteins	Slr0869	Dynamin	Membrane fission/fusion
ESCRT-III proteins	Sll0617 (IM30/VIPP1)	ESCRT-III	Membrane fission
	Slr1188 (PspA)		Part of a membrane stress response system

Supplemental Table S7.

Gene name	Protein accession of the reference from NCBI	Orthogroup	*Gloeobacter violaceus* PCC 7421 (GCA_000011385.1) ortholog accession from NCBI	Bitscore	Phylogenetic tree of the corresponding orthogroup	Best-fit model for the orthogroup phylogenetic tree	Phylogenetic tree of the cyanobacterial sampling	Best-fit model fot eh phylogenetic tree of the cyanobacterial sampling
**Assembly factors of PSII**								
YidC	P74155.1	OG0000214	BAC90172.1	342	Fig. S94	LG+F+R10	Fig. S136	Q.YEAST+I+R7
SecY	P77964.1	OG0000240	BAC89334.1	527	Fig. S71	LG+F+R10	Fig. S117	LG+F+I+R7
SecE	BAA17421.1	OG0000328	BAC88770.1	57.8	Fig. S68	Q.PFAM+R8	Fig. S114	Q.INSECT+F+I+R5
SecG	BAA18612.1	OG0000335	BAC88772.1	73.6	Fig. S70	LG+F+R8	Fig. S116	Q.YEAST+F+R6
SecD	BAA10118.1	OG0000360	BAC92107.1	458	Fig. S67	LG+F+R10	Fig. S113	LG+F+I+R8
SecF	BAA10119.1	OG0000411	BAC92108.1	244	Fig. S69	LG+F+R10	Fig. S115	LG+F+I+R9
SecA	BAA10347.1	OG0000154	BAC89777.1	1229	Fig. S95	LG+R10	Fig. S112	Q.YEAST+R10
Slr0144	BAA18544.1	OG0003484	-	-	Fig. S77	LG+I+R5	Fig. S138	Q.PFAM+R6
ChlG	QWO79731.1	OG0002917	BAC89750.1	421	Fig. S51	LG+F+R5	Fig. S96	Q.PFAM+I+R7
Ycf39	P74429.1	OG0001181	BAC91198.1	214	Fig. S91	Q.PFAM+F+I+R9	Fig. S134	LG+I+R7
HliC	P73563.2	OG0001968,OG00008795,OG0021297	Unidentified	-	Fig. S55	Q.PFAM+R7	-	-
HliD	P72932.1	OG0001968	Unidentified	-	Fig. S55	Q.PFAM+R7	-	-
Pitt	BAA18461.1	OG0003587	BAC92203.1	61.6	Fig. S57	LG+F+I+R4	Fig. S102	LG+F+R7
PratA	BAA17116.1	OG0005899	-	-	Fig. S58	LG+F+R3	Fig. S103	LG+F+R6
CtpA	Q55669.1	OG0000126	BAC88008.1	363	Fig. S52	Q.PFAM+F+R10	Fig. S97	LG+F+R10

Slr0286	BAA18506.1	OG0013412	-	-	Fig. S80	Q.PFAM+G4	Fig. S124	JTT+F+I+G4
Slr2013	BAA17260.1	OG0001053	-*	-	Fig. S89	Q.PFAM+F+I+R9	Fig . S137	Q.PFAM+F+I+R7
Ycf48	BAA17091.1	OG0004644	BAC88796.1	218	Fig. S92	Q.PFAM+G4	Fig. S135	Q.YEAST+I+R7
Sll0408	AA10250.1	OG0000241	BAC89441.1	194	Fig. S72	WAG+R8	Fig. S118	Q.PFAM+I+R7
RubA	P73068.1	OG0001187	BAC88795.1	100	Fig. S66	WAG+I+R5	Fig. S111	Q.PFAM+I+R5
Psb28	Q55356.3	OG0005257	BAC88869.1	56.6	Fig. S60	LG+G4	Fig. S105	Q.YEAST+I+R6
Sll0933	BAA16881.1	OG0005529	BAC89681.1	70.5	Fig. S75	Q.PFAM+F+I+G4	Fig. S121	Q.PFAM+F+I+R7
HliA	P73183.1	OG0001968	Unidentified	-	Fig. S55	Q.PFAM+R7	-	-
HliB	P73429.1	OG0001968,OG0009313	Unidentified	-	Fig. S55	Q.PFAM+R7	-	-
Psb35	BAA18333.1	OG0006562	BAC87990.1	47.4	Fig. S64	Q.PFAM+G4	Fig. S109	Q.PFAM+I+R5
Psb27	QWO79325.1	OG0005894	-	-	Fig. S59	Q.PFAM+I+G4	Fig. S104	Q.PLANT+F+I+R6
Sll0606	BAA10364.1	OG0000139	(BAC90294.1)	50.4	Fig. S74	Q.PFAM+F+R10	Fig. S120	
Psb34	BAA18143.1	OG0004508	-	-	Fig. S63	LG+G4	Fig. S108	Q.PFAM+I+R5
CyanoP (PsbP-like)*	BAA18019.1	OG0005391	BAC89381.1	54.3	Fig. S53	Q.PFAM+G4	Fig. S98	Q.PFAM+R6
Slr0565	BAA10482.1	OG0001631,OG0001293	BAC90053.1	152	Fig. S83	WAG+F+R8	Fig. S127	LG+F+R7
Psb32	BAA18232.1	OG00006067	-	-	Fig. S62	Q.PFAM+I+G4	Fig. S107	LG+F+R5
Slr1761	BAL34568.1	OG0000856	BAC88782.1	183	Fig. S87	WAG+F+R8	Fig. S131	WAG+F+I+R6
Psb29	BAA18023.1	OG0005016	BAC89341.1	164	Fig. S61	Q.PFAM+I+G	Fig. S106	Q.PFAM+I+R7
Slr0151	BAA18551.1	OG0001093	-	-	Fig. S78	LG+F+R8	-	-
PsbN	P26286.1	OG0005699	BAC90942.1	50.1	Fig. S65	LG+I+G4	Fig. S110	MTZOA+I+G4
PAM71	P52876.1	OG0001451	-	-	Fig. S56	LG+F+R8	Fig. S101	LG+F+R5
**Proteins involved in membrane dynamics**								
Slr0232	BAA10237.1	OG0000227	BAC89450.1	137	Fig. S79	LG+F+R10	Fig. S123	Q.PLANT+F+I+R7
Slr0305	BAA10672.1	OG0000999	-	-	Fig. S81	LG+F+R7	Fig. S125	Q.PLANT+F+I+R7
Sll0509	BAL36946.1	OG0008882	-*	-	Fig. S73	Q.PFAM+I+G4	Fig. S119	Q.PFAM+F+I+R6
Slr1106	BFM39848.1	OG0001215	-	-	Fig. S85	Q.PFAM+F+R8	Fig. S129	Q.PLANT+F+R6
Slr1768	BAA17070.1	OG0001215	-	-	Fig. S88	Q.PFAM+F+R8	Fig. S132	Q.PLANT+F+G4
Sll1021	BAA16946.1	OG0005308	-	-	Fig. S76	LG+F+G4	Fig. S122	Q.INSECT+F+R5
Slr1128	P72655.1	OG0000457	BAC90115.1	338	Fig. S86	LG+R10	Fig. S130	Q.PLANT+I+R7

Sll0815	BAA18117.1	OG0000457	-	-	Fig. S86	LG+R10	-	-
Sll1633 (FtsZ)	P73456.1	OG0000182	BAC88239.1	469	Fig. S54	Q.YEAST+R10	Fig. S99	Q.INSECT+I+R9
Slr0483	BAA10316.1	OG0005888	-	-	Fig. S82	LG+F+I+G4	Fig. S126	Q.PLANT+F+R7
Slr0869	BAA17817.1	OG0010292	-	-	Fig. S84	LG+F+I+R3	Fig. S128	Q.PLANT+F+I+R7
Sll0617 (IM30/VIPP1)	Q55707.1	OG0001588	BAC88839.1	217	Fig. S90	Q.YEAST+F+R7	Fig. S100	Q.INSECT+F+G4
Slr1188 (PspA)	AGF53385.1	OG0001588	-	-	Fig. S90	Q.YEAST+F+R7	Fig. S100	Q.INSECT+F+G4

Supplemental Table S8.

**Protein**	**Vampirovibrioniae**	**Sericytochromatia**	**Marguslibacteria**
**PSII assembly factors**			
YidC	Yes	Yes	Yes
SecY	Yes	Yes	Yes
SecE	Yes	Yes	Yes
SecG	Yes	Yes	Yes
SecD	Yes	Yes	Yes
SecF	Yes	Yes	Yes
SecA	Yes	Yes	Yes
Slr0144	No	Yes	No
**ChlG**	No	No	No
**Ycf39**	No	No	No
**Pitt**	No	No	No
PratA	Yes	Yes	No
**CtpA**	No	No	No
Slr2013	Yes	No	No
**Slr0286**	No	No	No
**Ycf48**	No	No	No
**Sll0408**	No	No	No
**RubA**	No	No	No
**Psb28**	No	No	No
**Sll0933**	No	No	No
**Psb35**	No	No	No
**Psb27**	No	No	No
**Slll0606**	No	No	No
**Psb34**	No	No	No
Slr0565	Yes	Yes	Yes
Slr1761	Yes	Yes	Yes

**Psb32**	No	No	No
**Psb29**	No	No	No
**PsbN**	No	No	No
**Membrane dynamics**			
**Slr0509**	No	No	No
Slr0232	No	Yes	No
Slr0305	Yes	Yes	No
Sll1021	No	Yes	No
**Slr1106**	No	No	No
**Slr1128**	No	No	No
**Slr1768**	No	No	No
FtsZ	Yes	Yes	Yes
**Slr0483**	No	No	No
**Slr0869**	No	No	No
**IM30 (VIPP1)**	No	No	No
PspA	Yes	Yes	No
**Manganese homeostasis**			
**CyanoP**	No	No	No
PAM71	No	Yes	Yes

References

Addlesee, H. A., Fiedor, L., & Hunter, C. N. (2000). Physical Mapping of *bchG* , *orf427* , and *orf177* in the Photosynthesis Gene Cluster of *Rhodobacter sphaeroides*: Functional Assignment of the Bacteriochlorophyll Synthetase Gene. *Journal of Bacteriology*, *182*(11), 3175–3182. https://doi.org/10.1128/JB.182.11.3175-3182.2000

Akulinkina, D. V., Bolychevtseva, Yu. V., Elanskaya, I. V., Karapetyan, N. V., & Yurina, N. P. (2015). Association of high light-inducible HliA/HliB stress proteins with photosystem 1 trimers and monomers of the cyanobacterium Synechocystis PCC 6803. *Biochemistry (Moscow)*, *80*(10), 1254–1261. https://doi.org/10.1134/s0006297915100053

Anbudurai, P. R., Mor, T. S., Ohad, I., Shestakov, S. V., & Pakrasi, H. B. (1994). The ctpA gene encodes the C-terminal processing protease for the D1 protein of the photosystem II reaction center complex. *Proceedings of the National Academy of Sciences*, *91*(17), 8082–8086. https://doi.org/10.1073/pnas.91.17.8082

Armbruster, U., Zühlke, J., Rengstl, B., Kreller, R., Makarenko, E., Rühle, T., Schünemann, D., Jahns, P., Weisshaar, B., Nickelsen, J., & Leister, D. (2010). The *Arabidopsis* Thylakoid Protein PAM68 Is Required for Efficient D1 Biogenesis and Photosystem II Assembly. *The Plant Cell*, *22*(10), 3439–3460. https://doi.org/10.1105/tpc.110.077453

Bečková, M., Gardian, Z., Yu, J., Konik, P., Nixon, P. J., & Komenda, J. (2017). Association of Psb28 and Psb27 Proteins with PSII-PSI Supercomplexes upon Exposure of Synechocystis sp. PCC 6803 to High Light. *Molecular Plant*, *10*(1), 62–72. https://doi.org/10.1016/j.molp.2016.08.001

Bučinská, L., Kiss, E., Konik, P., Knoppova, J., Komenda, J., & Sobotka, R. (2018). The ribosome-bound protein Pam68 promotes insertion of chlorophyll into the CP47 subunit of Photosystem II. *Plant Physiology*, pp.00061.2018. https://doi.org/10.1104/pp.18.00061

Chidgey, J. W., Linhartová, M., Komenda, J., Jackson, P. J., Dickman, M. J., Canniffe, D. P., Koník, P., Pilný, J., Hunter, C. N., & Sobotka, R. (2014a). A Cyanobacterial Chlorophyll Synthase-HliD

Complex Associates with the Ycf39 Protein and the YidC/Alb3 Insertase. *The Plant Cell*, *26*(3), 1267–1279. https://doi.org/10.1105/tpc.114.124495

Chidgey, J. W., Linhartová, M., Komenda, J., Jackson, P. J., Dickman, M. J., Canniffe, D. P., Koník, P., Pilný, J., Hunter, C. N., & Sobotka, R. (2014b). A Cyanobacterial Chlorophyll Synthase-HliD Complex Associates with the Ycf39 Protein and the YidC/Alb3 Insertase. *The Plant Cell*, *26*(3), 1267–1279. https://doi.org/10.1105/tpc.114.124495

Dobáková, M., Sobotka, R., Tichý, M., & Komenda, J. (2009). Psb28 Protein Is Involved in the Biogenesis of the Photosystem II Inner Antenna CP47 (PsbB) in the Cyanobacterium *Synechocystis* sp. PCC 6803. *Plant Physiology*, *149*(2), 1076–1086. https://doi.org/10.1104/pp.108.130039

Finn, R. D., Clements, J., & Eddy, S. R. (2011). HMMER web server: Interactive sequence similarity searching. *Nucleic Acids Research*, *39*(suppl), W29–W37. https://doi.org/10.1093/nar/gkr367

Frain, K. M., Gangl, D., Jones, A., Zedler, J. A. Z., & Robinson, C. (2016). Protein translocation and thylakoid biogenesis in cyanobacteria. *Biochimica et Biophysica Acta (BBA) - Bioenergetics*, *1857*(3), 266–273. https://doi.org/10.1016/j.bbabio.2015.08.010

Fu, A., He, Z., Cho, H. S., Lima, A., Buchanan, B. B., & Luan, S. (2007). A chloroplast cyclophilin functions in the assembly and maintenance of photosystem II in *Arabidopsis thaliana*. *Proceedings of the National Academy of Sciences*, *104*(40), 15947–15952. https://doi.org/10.1073/pnas.0707851104

Gathmann, Sven, Eva Rupprecht, Uwe Kahmann, & Dirk Schneider. (2008). A Conserved Structure and Function of the YidC Homologous Protein Slr1471 from Synechocystis sp. PCC 6803. *Journal of Microbiology and Biotechnology*, *18*(6), 1090–1094.

Karamoko, M., Cline, S., Redding, K., Ruiz, N., & Hamel, P. P. (2011). Lumen Thiol Oxidoreductase1, a Disulfide Bond-Forming Catalyst, Is Required for the Assembly of Photosystem II in*Arabidopsis*. *The Plant Cell*, *23*(12), 4462–4475. https://doi.org/10.1105/tpc.111.089680

Karnauchov, I., Herrmann, R. G., Pakrasi, H. B., & Klösgen, R. B. (1997). Transport of CtpA Protein from the Cyanobacterium *Synechocystis* 6803 Across the Thylakoid Membrane in Chloroplasts.

European Journal of Biochemistry, *249*(2), 497–504. https://doi.org/10.1111/j.1432-1033.1997.t01-1-00497.x

Kashino, Y., Takahashi, T., Inoue-Kashino, N., Ban, A., Ikeda, Y., Satoh, K., & Sugiura, M. (2007). Ycf12 is a core subunit in the photosystem II complex. *Biochimica et Biophysica Acta (BBA) - Bioenergetics*, *1767*(11), 1269–1275. https://doi.org/10.1016/j.bbabio.2007.08.008

Keren, N., Ohkawa, H., Welsh, E. A., Liberton, M., & Pakrasi, H. B. (2005). Psb29, a Conserved 22-kD Protein, Functions in the Biogenesis of Photosystem II Complexes in *Synechocystis* and *Arabidopsis*. *The Plant Cell*, *17*(10), 2768–2781. https://doi.org/10.1105/tpc.105.035048

Kiss, É., Knoppová, J., Aznar, G. P., Pilný, J., Yu, J., Halada, P., Nixon, P. J., Sobotka, R., & Komenda, J. (2019). A Photosynthesis-Specific Rubredoxin-Like Protein Is Required for Efficient Association of the D1 and D2 Proteins during the Initial Steps of Photosystem II Assembly. *The Plant Cell*, *31*(9), 2241–2258. https://doi.org/10.1105/tpc.19.00155

Knoppová, J., Yu, J., Konik, P., Nixon, P. J., & Komenda, J. (2016). CyanoP is Involved in the Early Steps of Photosystem II Assembly in the Cyanobacterium*Synechocystis*sp. PCC 6803. *Plant and Cell Physiology*, *57*(9), 1921–1931. https://doi.org/10.1093/pcp/pcw115

Komenda, J., Knoppová, J., Kopečná, J., Sobotka, R., Halada, P., Yu, J., Nickelsen, J., Boehm, M., & Nixon, P. J. (2012). The Psb27 Assembly Factor Binds to the CP43 Complex of Photosystem II in the Cyanobacterium *Synechocystis* sp. PCC 6803. *Plant Physiology*, *158*(1), 476–486. https://doi.org/10.1104/pp.111.184184

Komenda, J., Nickelsen, J., Tichý, M., Prášil, O., Eichacker, L. A., & Nixon, P. J. (2008). The Cyanobacterial Homologue of HCF136/YCF48 Is a Component of an Early Photosystem II Assembly Complex and Is Important for Both the Efficient Assembly and Repair of Photosystem II in Synechocystis sp. PCC 6803. *Journal of Biological Chemistry*, *283*(33), 22390–22399. https://doi.org/10.1074/jbc.m801917200

Komenda, J., & Sobotka, R. (2016). Cyanobacterial high-light-inducible proteins—Protectors of chlorophyll–protein synthesis and assembly. *Biochimica et Biophysica Acta (BBA) - Bioenergetics*, *1857*(3), 288–295. https://doi.org/10.1016/j.bbabio.2015.08.011

Konert, M. M. (2022). High-light-inducible proteins HliA and HliB: pigment binding and protein–protein interactions. *Photosynthesis Research*, *152*, 317–332.

Kufryk, G. I., & Vermaas, W. F. J. (2001). A Novel Protein Involved in the Functional Assembly of the Oxygen-Evolving Complex of Photosystem II in *Synechocystis* sp. PCC 6803. *Biochemistry*, *40*(31), 9247–9255. https://doi.org/10.1021/bi0026526

Kufryk, G. I., & Vermaas, W. F. J. (2003). Slr2013 Is a Novel Protein Regulating Functional Assembly of Photosystem II in *Synechocystis* sp. Strain PCC 6803. *Journal of Bacteriology*, *185*(22), 6615–6623. https://doi.org/10.1128/jb.185.22.6615-6623.2003

Lima, A., Lima, S., Wong, J. H., Phillips, R. S., Buchanan, B. B., & Luan, S. (2006). A redox-active FKBP-type immunophilin functions in accumulation of the photosystem II supercomplex in *Arabidopsis thaliana*. *Proceedings of the National Academy of Sciences*, *103*(33), 12631–12636. https://doi.org/10.1073/pnas.0605452103

Linhartová, M., Bučinská, L., Halada, P., Ječmen, T., Šetlík, J., Komenda, J., & Sobotka, R. (2014). Accumulation of the Type IV prepilin triggers degradation of SecY and YidC and inhibits synthesis of Photosystem II proteins in the cyanobacterium *Synechocystis* PCC 6803. *Molecular Microbiology*, *93*(6), 1207–1223. https://doi.org/10.1111/mmi.12730

Markin, A., Wagle, S., Grover, S., Vincent Baker, A. L., Eulenstein, O., & Anderson, T. K. (2023). PARNAS: Objectively Selecting the Most Representative Taxa on a Phylogeny. *Systematic Biology*, *72*(5), 1052–1063. https://doi.org/10.1093/sysbio/syad028

Nowaczyk, M. M. (2014). Localization of the CyanoP binding site on photosystem II by surface plasmon resonance spectroscopy. *Frontiers in Plant Science*, *5*(595).

Parks, D. H., Chuvochina, M., Rinke, C., Mussig, A. J., Chaumeil, P.-A., & Hugenholtz, P. (2022). GTDB: An ongoing census of bacterial and archaeal diversity through a phylogenetically consistent, rank normalized and complete genome-based taxonomy. *Nucleic Acids Research*, *50*(D1), D785–D794. https://doi.org/10.1093/nar/gkab776

Pascual-Aznar, G., Konert, G., Bečkov�, M., Kotabov�, E., Gardian, Z., Knoppov�, J., Bučinsk�, L., Kaňa, R., Sobotka, R., & Komenda, J. (2021). Psb35 Protein Stabilizes the CP47 Assembly Module and Associated High-Light Inducible Proteins during the Biogenesis of Photosystem II

in the Cyanobacterium*Synechocystis*sp. PCC6803. *Plant and Cell Physiology*, *62*(1), 178–190. https://doi.org/10.1093/pcp/pcaa148

Plöchinger, M., Schwenkert, S., Von Sydow, L., Schröder, W. P., & Meurer, J. (2016). Functional Update of the Auxiliary Proteins PsbW, PsbY, HCF136, PsbN, TerC and ALB3 in Maintenance and Assembly of PSII. *Frontiers in Plant Science*, *7*. https://doi.org/10.3389/fpls.2016.00423

Proctor, M. S., Chidgey, J. W., Shukla, M. K., Jackson, P. J., Sobotka, R., Hunter, C. N., & Hitchcock, A. (2018). Plant and algal chlorophyll synthases function in *Synechocystis* and interact with the YidC/Alb3 membrane insertase. *FEBS Letters*, *592*(18), 3062–3073. https://doi.org/10.1002/1873-3468.13222

Rahimzadeh-Karvansara, P., Pascual-Aznar, G., Bečková, M., & Komenda, J. (2022). Psb34 protein modulates binding of high-light-inducible proteins to CP47-containing photosystem II assembly intermediates in the cyanobacterium Synechocystis sp. PCC 6803. *Photosynthesis Research*, *152*(3), 333–346. https://doi.org/10.1007/s11120-022-00908-9

Rengstl, B., Knoppová, J., Komenda, J., & Nickelsen, J. (2013). Characterization of a Synechocystis double mutant lacking the photosystem II assembly factors YCF48 and Sll0933. *Planta*, *237*(2), 471–480. https://doi.org/10.1007/s00425-012-1720-0

Rengstl, B., Oster, U., Stengel, A., & Nickelsen, J. (2011). An Intermediate Membrane Subfraction in Cyanobacteria Is Involved in an Assembly Network for Photosystem II Biogenesis. *Journal of Biological Chemistry*, *286*(24), 21944–21951. https://doi.org/10.1074/jbc.m111.237867

Roose, J. L., Kashino, Y., & Pakrasi, H. B. (2007). The PsbQ protein defines cyanobacterial Photosystem II complexes with highest activity and stability. *Proceedings of the National Academy of Sciences*, *104*(7), 2548–2553. https://doi.org/10.1073/pnas.0609337104

Sachelaru, I., Petriman, N. A., Kudva, R., Kuhn, P., Welte, T., Knapp, B., Drepper, F., Warscheid, B., & Koch, H.-G. (2013). YidC Occupies the Lateral Gate of the SecYEG Translocon and Is Sequentially Displaced by a Nascent Membrane Protein. *Journal of Biological Chemistry*, *288*(23), 16295–16307. https://doi.org/10.1074/jbc.m112.446583

Sakata, S., Mizusawa, N., Kubota-Kawai, H., Sakurai, I., & Wada, H. (2013). Psb28 is involved in recovery of photosystem II at high temperature in Synechocystis sp. PCC 6803. *Biochimica et*

Biophysica Acta (BBA) - Bioenergetics, *1827*(1), 50–59. https://doi.org/10.1016/j.bbabio.2012.10.004

Schottkowski, M., Ratke, J., Oster, U., Nowaczyk, M., & Nickelsen, J. (2009). Pitt, a Novel Tetratricopeptide Repeat Protein Involved in Light-Dependent Chlorophyll Biosynthesis and Thylakoid Membrane Biogenesis in Synechocystis sp. PCC 6803. *Molecular Plant*, *2*(6), 1289–1297. https://doi.org/10.1093/mp/ssp075

Šesták, Z. (2007). Golbeck, J.H. (ed.): Photosystem I. The Light-Driven Plastocyanin:Ferredoxin Oxidoreductase. *Photosynthetica*, *45*(4), 488–488. https://doi.org/10.1007/s11099-007-0083-4

Shen, G., Antonkine, M. L., Van Der Est, A., Vassiliev, I. R., Brettel, K., Bittl, R., Zech, S. G., Zhao, J., Stehlik, D., Bryant, D. A., & Golbeck, J. H. (2002). Assembly of Photosystem I. *Journal of Biological Chemistry*, *277*(23), 20355–20366. https://doi.org/10.1074/jbc.m201104200

Siebenaller, C., & Schneider, D. (2023). Cyanobacterial membrane dynamics in the light of eukaryotic principles. *Bioscience Reports*, *43*(2), BSR20221269. https://doi.org/10.1042/BSR20221269

Singh, A. K., Li, H., & Sherman, L. A. (2004). Microarray analysis and redox control of gene expression in the cyanobacterium *Synechocystis* sp. PCC 6803. *Physiologia Plantarum*, *120*(1), 27–35. https://doi.org/10.1111/j.0031-9317.2004.0232.x

Sirpiö, S., Khrouchtchova, A., Allahverdiyeva, Y., Hansson, M., Fristedt, R., Vener, A. V., Scheller, H. V., Jensen, P. E., Haldrup, A., & Aro, E. (2008). AtCYP38 ensures early biogenesis, correct assembly and sustenance of photosystem II. *The Plant Journal*, *55*(4), 639–651. https://doi.org/10.1111/j.1365-313x.2008.03532.x

Torabi, S., Umate, P., Manavski, N., Plöchinger, M., Kleinknecht, L., Bogireddi, H., Herrmann, R. G., Wanner, G., Schröder, W. P., & Meurer, J. (2014). PsbN Is Required for Assembly of the Photosystem II Reaction Center in *Nicotiana tabacum*. *The Plant Cell*, *26*(3), 1183–1199. https://doi.org/10.1105/tpc.113.120444

Wegener, K. M., Bennewitz, S., Oelmüller, R., & Pakrasi, H. B. (2011). The Psb32 Protein Aids in Repairing Photodamaged Photosystem II in the Cyanobacterium Synechocystis 6803. *Molecular Plant*, *4*(6), 1052–1061. https://doi.org/10.1093/mp/ssr044

Wegener, K. M., Welsh, E. A., Thornton, L. E., Keren, N., Jacobs, J. M., Hixson, K. K., Monroe, M. E., Camp, D. G., Smith, R. D., & Pakrasi, H. B. (2008). High Sensitivity Proteomics Assisted Discovery of a Novel Operon Involved in the Assembly of Photosystem II, a Membrane Protein Complex. *Journal of Biological Chemistry*, *283*(41), 27829–27837. https://doi.org/10.1074/jbc.m803918200

Wysocka, A. (2025). High-light-inducible proteins control associations between chlorophyll synthase and the Photosystem II biogenesis factor Ycf39. *Plant Physiology*, *198*, kiaf213.

Yang, H., Liao, L., Bo, T., Zhao, L., Sun, X., Lu, X., Norling, B., & Huang, F. (2014). Slr0151 in *Synechocystis* sp. PCC 6803 is required for efficient repair of photosystem II under high-light condition. *Journal of Integrative Plant Biology*, *56*(12), 1136–1150. https://doi.org/10.1111/jipb.12275

Zhang, S., Frankel, L. K., & Bricker, T. M. (2010). The Sll0606 Protein Is Required for Photosystem II Assembly/Stability in the Cyanobacterium Synechocystis sp. PCC 6803. *Journal of Biological Chemistry*, *285*(42), 32047–32054. https://doi.org/10.1074/jbc.m110.166983
